# Mapping the contact surfaces in the Lamin A:AIMP3 complex by hydrogen/deuterium exchange FT-ICR mass spectrometry

**DOI:** 10.1371/journal.pone.0181869

**Published:** 2017-08-10

**Authors:** Yeqing Tao, Pengfei Fang, Sunghoon Kim, Min Guo, Nicolas L. Young, Alan G. Marshall

**Affiliations:** 1 Department of Chemistry and Biochemistry, Florida State University, Tallahassee, Florida, United States of America; 2 Department of Cancer Biology, The Scripps Research Institute, Scripps Florida, Jupiter, Florida, United States of America; 3 Medicinal Bioconvergence Research Center, College of Pharmacy, Seoul National University, Seoul, South Korea; 4 Verna & Marrs McLean Department of Biochemistry & Molecular Biology, Baylor College of Medicine, Houston, Texas, United States of America; 5 Ion Cyclotron Resonance Program, National High Magnetic Field Laboratory, Florida State University, Tallahassee, Florida, United States of America; Pacific Northwest National Laboratory, UNITED STATES

## Abstract

Aminoacyl-tRNA synthetases-interacting multifunctional protein3 (AIMP3/p18) is involved in the macromolecular tRNA synthetase complex via its interaction with several aminoacyl-tRNA synthetases. Recent reports reveal a novel function of AIMP3 as a tumor suppressor by accelerating cellular senescence and causing defects in nuclear morphology. AIMP3 specifically mediates degradation of mature Lamin A (LmnA), a major component of the nuclear envelope matrix; however, the mechanism of how AIMP3 interacts with LmnA is unclear. Here we report solution-phase hydrogen/deuterium exchange (HDX) for AIMP3, LmnA, and AIMP3 in association with the LmnA C-terminus. Reversed-phase LC coupled with LTQ 14.5 T Fourier transform ion cyclotron resonance mass spectrometry (FT-ICR MS) results in high mass accuracy and resolving power for comparing the D-uptake profiles for AIMP3, LmnA, and their complex. The results show that the AIMP3-LmnA interaction involves one of the two putative binding sites and an adjacent novel interface on AIMP3. LmnA binds AIMP3 via its extreme C-terminus. Together these findings provide a structural insight for understanding the interaction between AIMP3 and LmnA in AIMP3 degradation.

## Introduction

Aminoacyl-tRNA synthetases (AARSs) are vital for gene translation. They catalyze the attachment of specific amino acids to their cognate tRNAs as building blocks of protein synthesis. About half of the cytoplasmic AARSs reside in the multi-tRNA synthetase complex (MSC). MSC is composed of nine distinctive AARSs and three aminoacyl-tRNA synthetases-interacting multifunctional proteins (AIMP1, 2, 3 or MSC p43, p38, p18) [1]. The non-enzymatic factors, AIMPs, serve as molecular scaffolds that bind AARS components in MSC for protein translation activity but also allow them to be released for non-translational functions [2], in diverse biological processes [3]. Among them, AIMP3, normally known to bind methionyl-tRNA synthetase (MRS) and the dual glutaminyl-tRNA and prolyl-tRNA synthetase (EPRS), is translocated to the nucleus to activate p53 in response to DNA damage or oncogenic stress [4],[5]. AIMP3 is also a potent tumor suppressor by causing accelerated cellular senescence [6]. Overexpressing AIMP3 in transgenic mice causes a progeroid phenotype. Cells overexpressing AIMP3 exhibit accelerated senescence and defects in nuclear morphology [7], due to the enhanced degradation of mature Lamin A protein (LmnA).

LmnA is a major component of the matrix underlying the inner nuclear membrane. Mutations in LmnA have been associated with progeria, such as the Hutchinson-Gilford progeria syndrome (HGPS) [8,9]. In the Lamin family, LmnA and LmnC are both encoded by the same gene, LMNA, but their mRNAs are alternatively spliced [10,11]. Compared to LmnC, LmnA has a unique C-terminal region generated from maturation of LmnA, involving removal of 17 residues at the C-terminus of prelamin A/C. Progerin, the common mutant of LmnA that causes the HGPS, lacks 50 residues in the C-terminal region, preventing it from maturation [12–14]. It is interesting to note that overexpression of AIMP3 leads to degradation of LmnA without affecting LmnC, prelamin A, or progerin, and AIMP3 specifically binds LmnA [7].

Furthermore, AIMP3 mediates LmnA degradation by recruiting the ubiquitin ligase, Siah1, to promote LmnA ubiquitination [15,16]. *In vitro* pull-down assay confirms the direct interaction between AIMP3 and Siah1 [7]. Suppressed expression of AIMP3 reduces the amount of LmnA co-immunoprecipitated with Siah1, indicating that AIMP3 is capable of binding both LmnA and Siah1 simultaneously, and possibly mediating their interaction. Full understanding of the binding between AIMP3 and LmnA would potentially pave the way for discovering a working model for AIMP3-dependent LmnA degradation. Hence, investigating the underlying mechanism of AIMP3 interaction is the focus of this study. AIMP3-LmnA interaction takes place in the nucleus and is thus exclusive of its original scaffold function in the cytosolic MSC. Our previous X-ray crystallography results reveal two putative binding sites of AIMP3. One includes residues Arg^50^, Thr^68^, Lys^75^, Ala^91^, Gln^94^, Gln^95^, Glu^98^, and Asp^119^ (interface I), and the other consists of residues Glu^125^, Val^128^, Tyr^129^, Tyr^133^, Leu^162^, Arg^166^, and Phe^186^ (interface II) [17]. These interfaces are responsible for binding to MRS and EPRS in the MSC [18], and could hint AIMP3 binding to LmnA.

In addition to X-ray crystallography, other approaches for study of protein-ligand and protein-protein interaction include: cryo-electron microscopy [19], nuclear magnetic resonance (NMR) [20], electron microscopy [21,22], small angle X-ray scattering [23], hydrogen-deuterium exchange [24,25], chimeric molecule analysis [26], mutagenesis, and chemical cross-linking [27]. Although X-ray crystallography remains the most prominent and reliable method, it is not readily applicable for the AIMP3-LmnA complex, due to the highly extended and dynamic structure of LmnA (only a partial crystal structure is available for the LmnA) [28]. Similarly, NMR-based HDX is typically restricted to relatively small proteins (<30 kDa), making it a less favorable tool for studying protein complexes.

To investigate the role that AIMP3 plays in LmnA degradation, we report solution-phase hydrogen/deuterium exchange (HDX) monitored by high resolution Fourier transform ion cyclotron resonance mass spectrometry (FT-ICR MS) for AIMP3 and AIMP3 in complex with the LmnA C-terminus. Recent improvements in HDX analysis include automation [29,30], faster chromatographic separation [31,32], more efficient protein digestion [33–35], and enhanced data analysis software. The improved HDX-MS methodology has achieved successful epitope mapping [36,37], and subunit contacts in protein complexes up to 7.7 MDa [38].

With our hybrid linear ion trap 14.5 T FT-ICR instrument, we achieved 100% sequence coverage for peptides common to free and bound AIMP3. The key structural features of AIMP3 revealed by HDX are consistent with the crystal structure. Our HDX data identify regions showing significant decreases in D-uptake, suggesting that AIMP3 binds LmnA through the interacting surface consisting of both putative (Interface I) and novel binding sites. HDX results for LmnA reveal that the C-terminal 7 residues are critical for its binding to AIMP3.

## Materials and methods

### Expression and purification of His-Tev-AIMP3 and His-Strep-TrxA-LmnA

Full-length AIMP3 was constructed in vector pET28a with a His-Tev-tag fused to its N-terminus. The protein was expressed in BL21 (DE3) strain with 0.2 mM isopropyl β-D-1-thiogalactopyranoside for 20 h at 16°C. The cell pellet (from 4 liters) was lysed in a buffer containing 500 mM NaCl, 20 mM Tris-HCl at pH 8.0, and 25 mM imidazole, loaded onto a Ni-HiTrap column and washed with the same lysis buffer. Protein was eluted with a buffer containing 500 mM NaCl, 20 mM Tris-HCl at pH 8.0, and 250 mM imidazole. After elution, the protein was concentrated to ~15 mg/mL and passed through a desalting column in a buffer containing 150 mM NaCl and 20 mM Hepes-Na at pH 7.5 before further use. The C-terminal part of LmnA protein (567–646) was constructed in vector pET28a with a His-Strep-TrxA-tag fused to its N-terminus, expressed, and purified similarly.

### Hydrogen/deuterium exchange automation

HDX experiments were automated with an HTC PAL autosampler (Eksigent Technologies, Dublin, CA). The event sequence of experiments was optimized by an algorithm (HDX integrator) [39] that interlaces short HDX reaction periods during the longer reaction periods, so that the entire HDX experiment can be completed in the shortest possible time without overlap of LC injections. The HDX integrator enables modification of all experimental parameters, such as HDX reaction period, number of replicates, sample consumption, D_2_O volume, quench and digestion periods and volumes, and LC fraction collection. With these user-defined parameters, HDX integrator calculates the event sequence for the autosampler and compiles it into a command list that is readable and executable by the HTC PAL autosampler.

### Hydrogen/deuterium exchange

Hydrogen/deuterium exchange (HDX) was performed as previously described [40]. HDX samples were prepared in 5 μL volume at 40 μM (His-Tev-AIMP3, His-Strep-TrxA-LmnA, and AIMP3:LmnA complex (AIMP3 and LmnA were mixed in 1:1 ratio to form the complex.) in 20 mM Hepes, 150 mM NaCl at pH 7.5. H/D exchange was initiated when this stock was diluted to 45 μL 20 mM Hepes, 150 mM NaCl in D_2_O (99.8 atom %) at pH 7.5. For the blank control, the sample was diluted in 20 mM HEPES, 150 mM NaCl in H_2_O at pH 7.5. For the zero-time control, HDX initiation and quench are performed simultaneously by adding quench buffer to D_2_O followed by sample addition. Triplicate data points were taken after 0, 0.5, 1, 2, 4, 8, 15, 30, 60, 120, and 240 min incubation at 0.4°C followed by quenching by addition of 25 μL of 200 mM tris(2-carboxyethyl)phosphine (TCEP), 6 M urea in 1.0% formic acid, and digestion with 25 μL of 40% (v/v) saturated protease type XIII (Sigma Aldrich, St Louis, MO) in 1.0% formic acid to yield a final pH of 2.3. Digestion proceeded for 3 min at 0.4°C before injection for LC-MS analysis. All HDX experiments and HPLC separation were conducted at 1°C, maintained by a MéCour Temperature Control cooling chamber (MéCour Temperature Control, LLC Groveland, MA).

### On-line LC-ESI FT-ICR MS

After proteolysis, the AIMP3 peptide (with and without LmnA) separation and desalting were performed over a Pro-Zap Expedite MS C_18_ column (1.5 μm particle size, 500 Å pore size, 2.1 x 10 mm^2^; Grace Davidson, Deerfield, IL) [41], with a Jasco high performance liquid chromatography/supercritical fluid chromatography (HPLC/SFC) system triggered by the HTC PAL autosampler (Eksigent Technologies). Both urea and TCEP are necessary for better proteolysis results, but are MS-incompatible. A high concentration of salt in the electrospray not only suppresses analyte signal but also accumulates in the ion source over time, causing a progressive decrease in ion signal. A divert valve was therefore employed to remove the salt during a 0.75 min washing period on the HPLC with solvent A at a flow rate of 300 μL/min before the gradient. Peptides were then eluted over 2 min with a gradient from 2 to 95% Solvent B (Solvent A: acetonitrile/H_2_O/formic acid (4.5:95:0.5) and Buffer B: acetonitrile/H_2_O/formic acid (95:4.5:0.5)). A post-column splitter reduces the LC flow rate by 1:1,000 for efficient electrospray ionization (ESI). To compensate for the extra 0.75 min during which additional back-exchange takes place, the gradient was shortened from 2.5 min to 2 min relative to our previously reported HDX procedure (S1 Fig) [24]. With the increased dynamic range as a result of replacing the LTQ front-end with a Velos Pro, the shorter gradient did not reduce the number of peptides identified. In fact, 258 peptides were identified from digestion of equine heart myoglobin, a 25% increase compared to our previous result.

After ionization by ESI at 3.8 kV, the sample was directed into a custom-built hybrid Velos Pro 14.5 T FT-ICR mass spectrometer (Thermo Fisher, San Jose, CA) [42]. Approximately 350 mass spectra were collected from *m/z* 210–1300 over a period of 6.5 min, at high mass resolving power (m/Δm_50%_ = 200,000 at *m/z* 400, in which Δm_50%_ is the peak full width at half-maximum peak height). External ion accumulation [43] was performed in the linear ion trap with a target ion population of 3 million charges for each FT-ICR measurement. Velos-accumulated ions were transferred (~1 ms transfer period[44] through three octopole ion guides (2.2 MHz, 250 V_p–p_) to a capacitively coupled[45] closed cylindrical ICR cell (55 mm i.d.) [46] for analysis. The ion external accumulation period was typically less than 50 ms during peptide elution, and the FT-ICR time-domain signal acquisition period was 767 ms (leading to an overall duty cycle of 1 Hz per acquisition). Automatic gain control [47] and high magnetic field [48] provided excellent external calibration [49,50] mass accuracy, resulting in rms mass error typically less than 500 ppb.

### Data analysis

Data were collected with Xcalibur software (Thermo Fisher Scientific) and analyzed by a custom analysis package [29,51]. Briefly, the software performs three major functions. The first is a “digest tool”, in which a peptide list is compiled by matching the isotopic envelopes identified from the blank control (no HDX) against those simulated for all possible peptide ions generated from the protein sequence. This process eliminates misidentified peptides by allowing a user-defined mass error tolerance, and applying a fitting algorithm that rejects the isotopic envelopes that do not match the simulated isotopic distribution. Next, the software picks all peaks above a user-specified signal-to-noise ratio threshold from all acquisitions with eluted peptides for all H/D exchange periods, and organizes them into a text file. Finally, the peptide list from the first step is compared to the peak list. The cumulative peak list is searched for peaks within each peptide’s *m/z* "sub-window", based on its charge state, number of exchangeable hydrogens, and peptide elemental composition. For each exchange period for each peptide, an averaged mass is calculated for all peaks for ions from the deuterated peptide within that “sub-window”.

After peptide masses were determined for the free and complexed proteins, the measured deuterium uptake percentage at each time point was calculated by dividing the measured deuteration level at each time point by the calculated maximum uptake, D_max_ (an *n*-amino acid-long peptide can take up to *n-1* backbone amide deuteriums in the absence of prolines). The average relative deuterium uptake difference (ARDD) for each exchange period is then calculated from the following equation:

$$ARDD=\sum_{i}\frac{Complexed(t_{i})-Free(t_{i})}{Complexed(t_{i})}$$ (1)

in which Complexed(*t*_*i*_) is the deuterium uptake for AIMP32 complexed with LmnA after a specified exchange period (*t*_*i*_) and Free(*t*_*i*_) is the deuterium uptake for free AIMP3 after the same exchange period. Time-course deuterium incorporation levels were generated by an MEM fitting method [52].

A deuterium uptake “heat map” is a visual representation of the localized deuteration for a given protein. It can confirm and complement structural information discovered by other methods such as X-ray crystallography. In this experiment, “heat maps” are drawn by summarizing deuterium uptake information for all peptides from each protein. Briefly, the deuterium uptake of each residue is calculated by averaging the deuteration levels of that residue from each overlapping peptide containing it, and the deuteration level of each residue is calculated by dividing the observed deuterium uptake by the maximum possible deuterium uptake for each peptide. Although deuterium uptake for each residue could vary across the peptide, so that this calculation does not represent the accurate deuteration for each residue, this approach incorporates all available information from all overlapping peptides without introducing bias by manually selecting which peptide to display in the “heat map”.

## Results and discussion

### Constructs for analyzing the AIMP3-LmnA interaction

**Fig 1:**
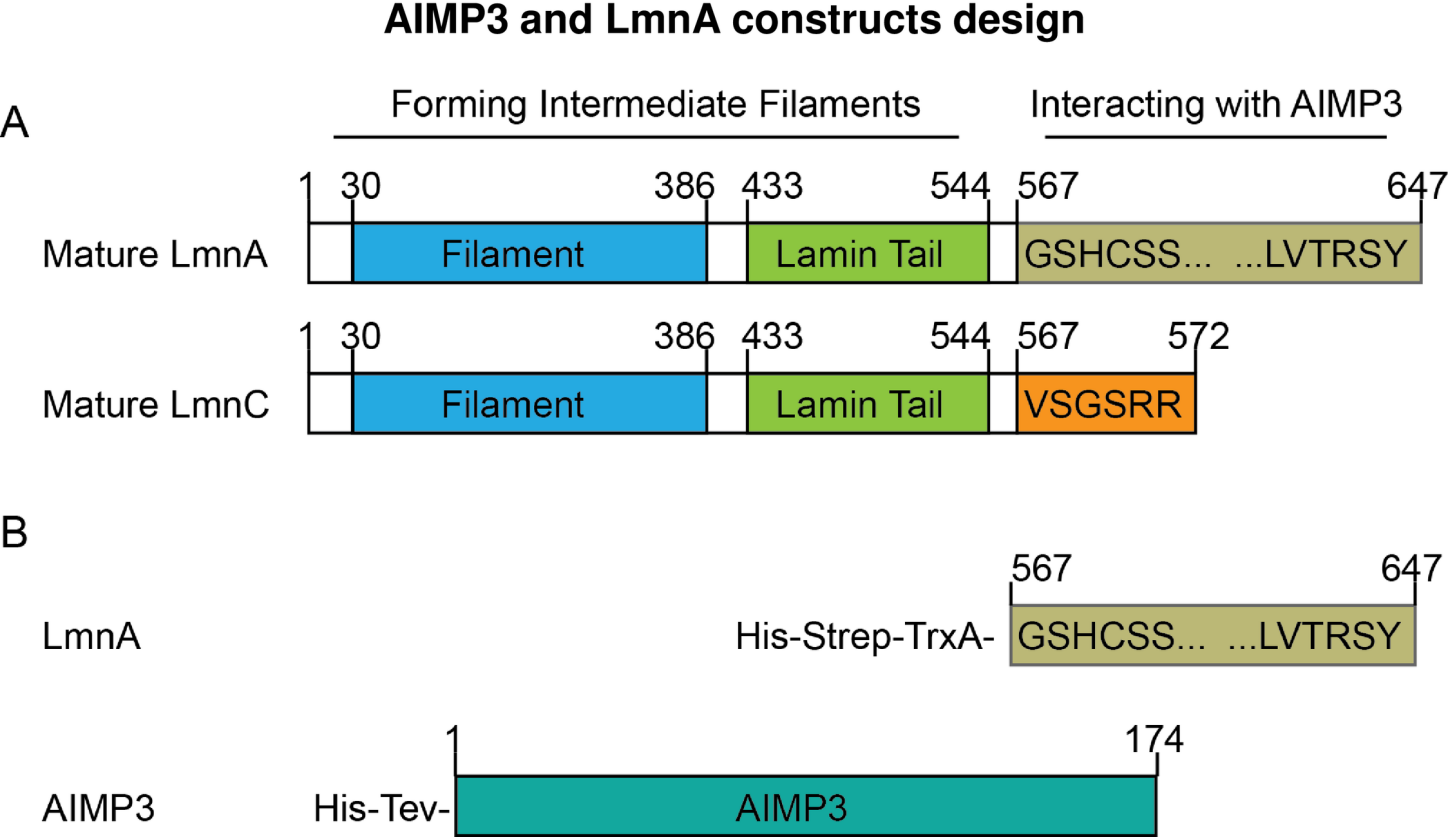
Constructs for analyzing the AIMP3-LmnA interaction. A. Primary structure difference between mature LmnA and LmnC. B. Recombinant protein constructs for interaction analysis.

AIMP3 specifically interacts with LmnA but not its isoform LmnC [7]. LmnA shares an identical N-terminal sequence (aa1-566) with LmnC. They differ only at the C-terminal region (aa567-647, Fig 1A). Together, these findings pinpoint the mature C-terminal region, aa567-647 as the site of binding to AIMP3. Therefore, we cloned LmnA (aa567-647) with an N-terminal monomeric thioredoxin (TrxA) tag to avoid aggregation of full-length LmnA (Fig 1B). The His-Tev-AIMP3 construct is ~22.4 kDa with a 6-his tag and a Tobacco Etch Virus protease cutting site at the N-terminus (aa1-22). His-Strep-TrxA-LmnA is composed of 80aa from the binding site of LmnA (aa567-647) linked to a streptavidin (Strep) tag and a thioredoxin (TrxA) tag.

### Sequence coverage for free AIMP3 and AIMP3-LmnA complex

**Fig 2:**
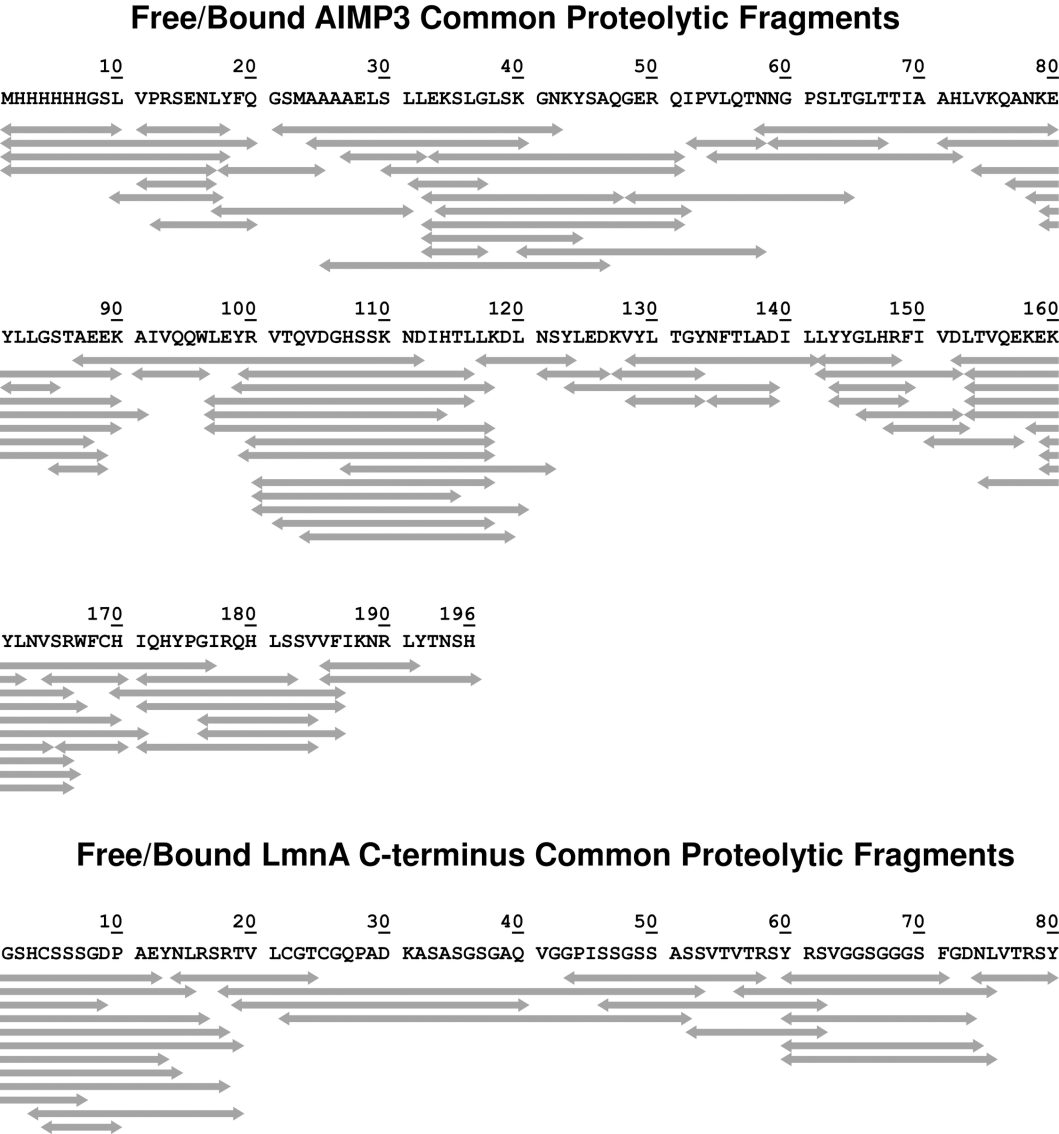
Sequence coverage for proteolytic peptides (5–30 aa in length) common to free His-Tev-AIMP3 and His-Tev-AIMP3 in complex (Top); and free His-Strep-TrxA-LmnA and His-Strep-TrxA-LmnA in complex (bottom). Peptides containing less than 5 or more than 30 amino acids are not considered, due to increased ambiguity and poor sequence localization. The displayed segments cover 100% of the sequences based on the common segments.

The first step in HDX analysis is to determine the sequence coverage for proteolytic fragments common to free and bound AIMP3. 147 peptides were identified for free His-Tev-AIMP3, and 122 for His-Tev-AIMP3 in the AIMP3-LmnA complex, with 73 common peptides. For His-Strep-TrxA-LmnA, 200 peptides in the free protein and 164 peptides in the AIMP3-LmnA complex were identified, with 83 common peptides. Among them, 23 peptides have coverage for the LmnA C-terminal 80 amino acids. Based on common peptides between the free and bound forms of both proteins, sequence coverage was 100% (Fig 2). Only those proteolytic peptides common to both free and bound are compared for HDX analysis. We generally disregard ambiguous isobaric peptides. For example, the following peptides have isobaric sequences in the protein:

140_ILLYYGLHRF_149 = 141_LLYYGLHRFI_150
142_LLYYGLHRFIVD_152 = 143_LYYGLHRFIVDL_153
171_IQHYPGIRQHLSSVVF_186 = 172_QHYPGIRQHLSSVVFI_187

We therefore replaced the binding site 142_LLYYGLHRFIVD_152 peptide by 143_LYYGLHR_149, for which there is no ambiguity. We retained the other two pairs of isobaric peptides, because they span very similar sequences.

### Determination of deuterium incorporation

LC runs from each of 12 HDX incubation periods in triplicate were collected for each HDX experiment. Peptides were identified by the custom software package, based on time domain transients acquired from ~150 LC fractions collected between ~1.3 min and ~3.5 min (S1 Fig). Proteolytic peptides whose masses matched within 2 ppm mass error tolerance for free and complexed His-Tev-AIMP3 were identified from the blanks (with no D_2_O exposure). For data collected after HDX incubation, the 1000 highest-magnitude peaks were picked from each acquisition (with a peak threshold magnitude of at least 6σ of baseline noise). A mass error tolerance of 1 ppm was used for picking deuterated ions.

### Deuterium uptake (“Heat”) map for free AIMP3 and LmnA

**Fig 3:**
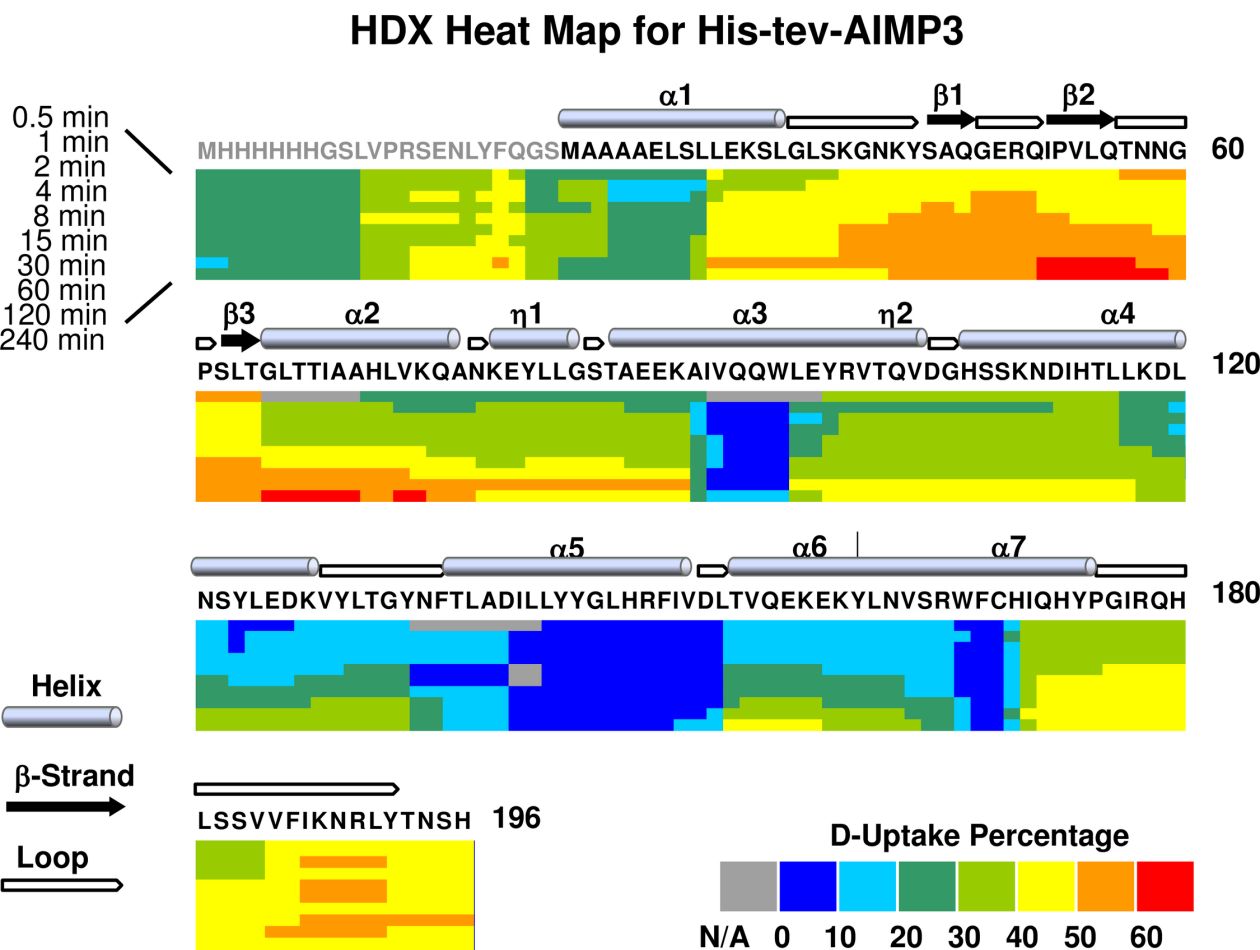
HDX heat map for deuterium uptake by free His-Tev-AIMP3. The His-Tev tag sequence is in grey. The deuteration level percentage is calculated by dividing the observed deuterium uptake by the total number of amide hydrogens (not counting proline) in that segment. For each peptide, the calculated deuteration level for each HDX incubation period (top left, proceeding from top to bottom: incubation periods of 0.5, 1, 2, 4, 8, 15, 30, 60, 120, and 240 min) is mapped onto the sequence. Secondary structure is noted on top of the sequence (PDB 2UZ8) [53]. Alpha helices and beta strands are numbered in order from N to C terminus.

For free AIMP3, D-uptake for the His-Tev-AIMP3 construct was measured following 0, 0.5, 1, 2, 4, 8, 15, 30, 60, 120, and 240 min H/D exchange periods. For each proteolytic peptide, the percentage of D-uptake (i.e., number of deuteriums divided by the number of amide hydrogens (not counting proline(s)) after each incubation period was compiled into a heat map. Examination of the free AIMP3 data reveals a significant correlation of solvent exposure with a previously developed model of the AIMP3 crystal structure (PDBID: 2UZ8) (Fig 3) [53]. AIMP3 contains seven α-helices and three β-strands, which are less solvent accessible than loops. The segment 37–64 presents the highest deuteration level in the N-terminal domain (α1, β1-β3, and α2), more than 40%, consistent with the crystal structure finding that this region consists of only β strands and loops. In fact, this region contains three anti-parallel β strands, which form a β sheet on the exterior surface away from the helices. The C-terminal domain consists of five helices (α3 to α7) and a coiled region at the extreme C-terminus. Among them, α3 and α5 form a bundle-like structure with α1 and α2 of the N-terminal domain, which stabilizes the structure through hydrophobic and ion interactions [53]. Overall, D-uptake profiles for peptides agree with the crystal structure very well.

**Fig 4:**
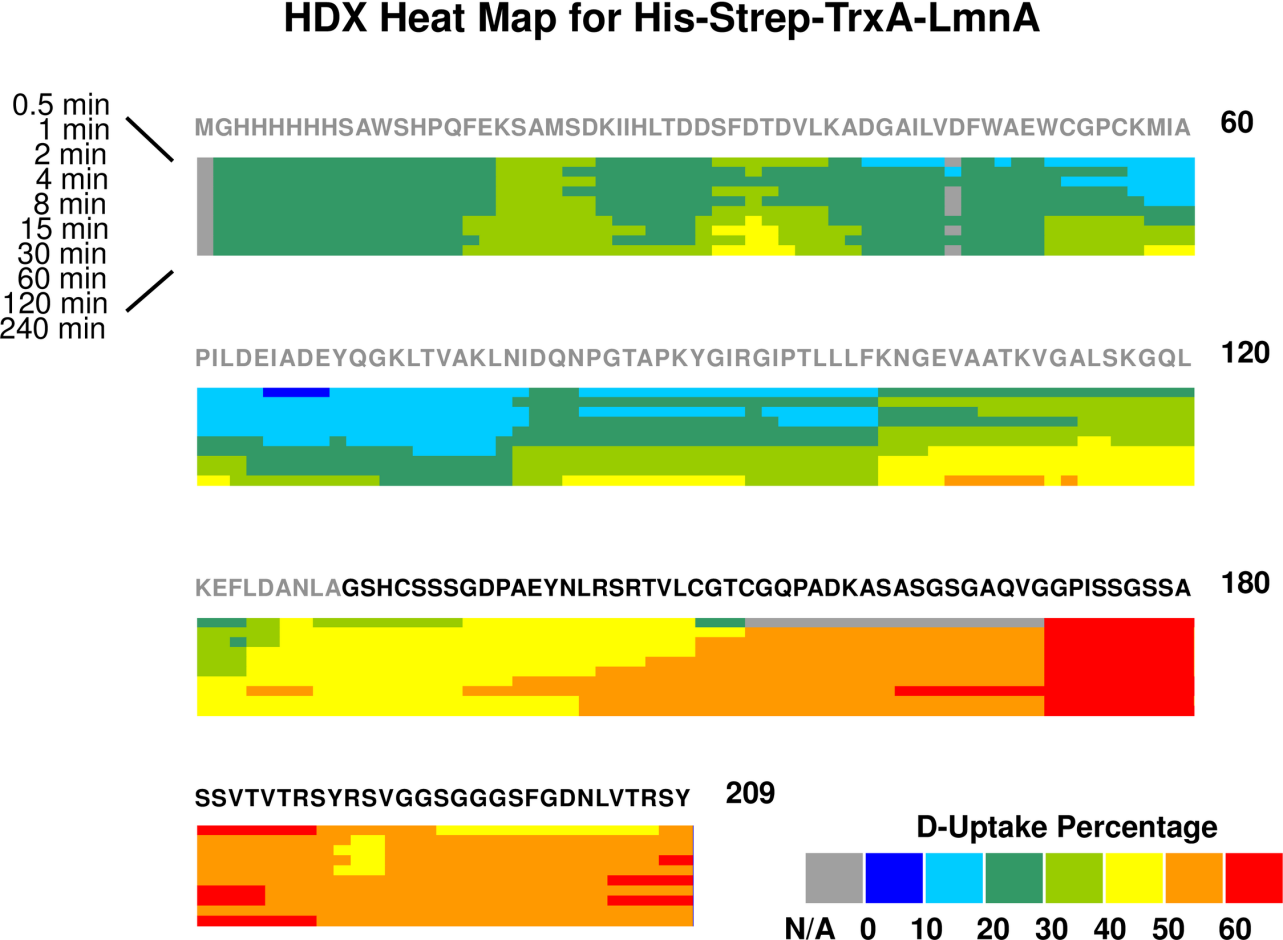
HDX heat map for deuterium uptake by free His-Strep-TrxA-LmnA. The His-Strep-TrxA tag sequence is in gray. The deuteration level percentage is calculated as for AIMP3.

Deuteration level for LmnA is calculated in the same way as for AIMP3. Compared to the His-Strep-TrxA tag, LmnA 80 amino acids have higher deuteration level, indicating that the C-terminus of LmnA is very likely un-structured and flexible. Specifically, regions aa170-188 and 203–209 (regions 608–626 and 641–647 in the full-length LmnA) have the highest D-uptake, >60%. (Fig 4) Because no structural information is available for that region, presumably due to the flexible nature of the C-terminus, preventing it to crystallize, our data is the first to reveal solvent exposure of the LmnA C-terminus.

### AIMP3-LmnA binding sites identified by HDX-MS

**Fig 5:**
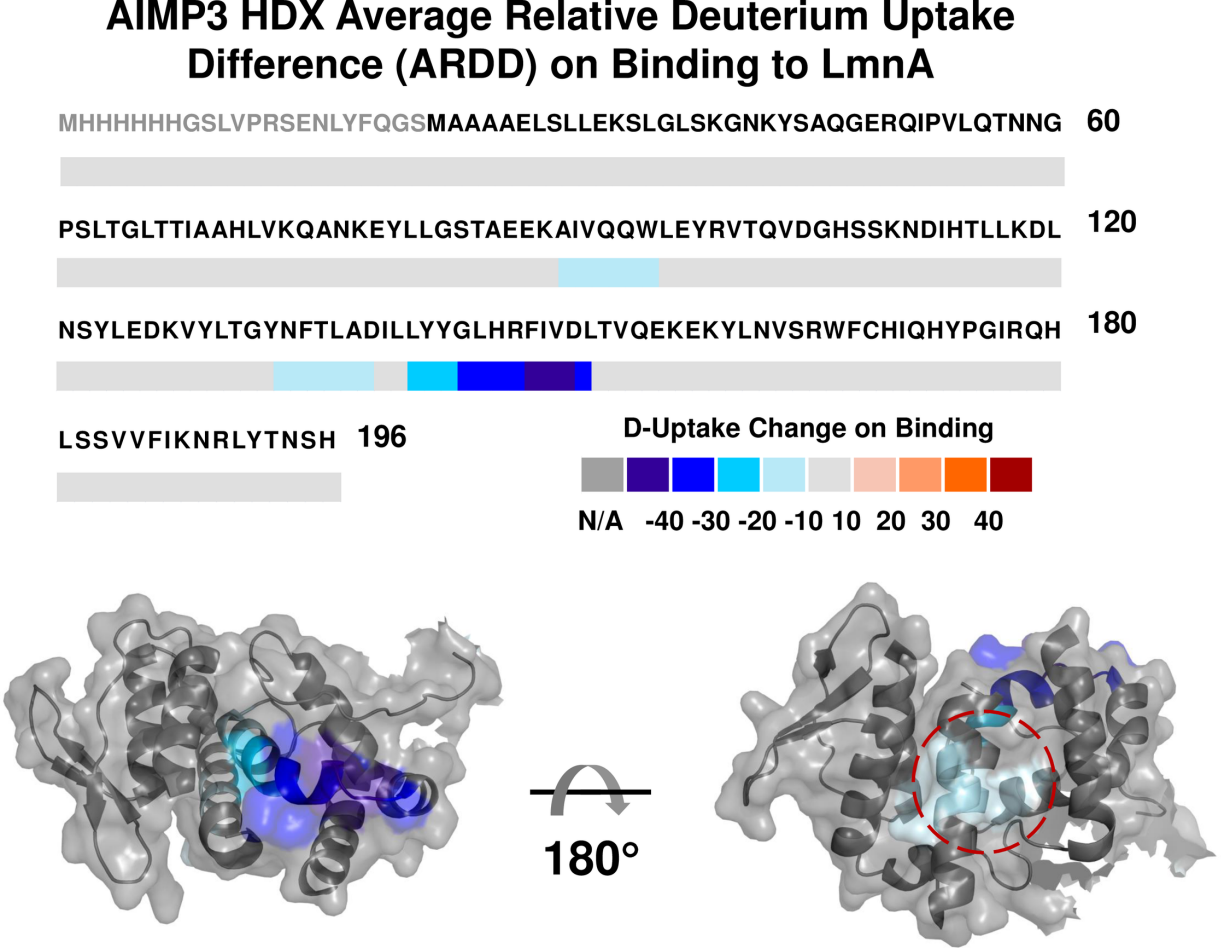
H/D exchange results for free and complexed AIMP3 with LmnA. For each of the proteolytic peptides common to free and bound AIMP3, the relative D-uptake change for AIMP3 on binding to LmnA (ARDD) is calculated as described by Eq 1. Peptide regions with significant deuterium uptake differences are mapped onto the crystal structure. Top: AIMP3 shows decreases in D-uptake for segments 91–96 and 134–152 upon binding to LmnA, consistent with the binding interface between AIMP3 and LmnA. Bottom: ARDD mapped onto the crystal structure (PDB 2UZ8). Note that segments 91–96 and 134–139 (red circle) are spatially close to each other.

**Fig 6:**
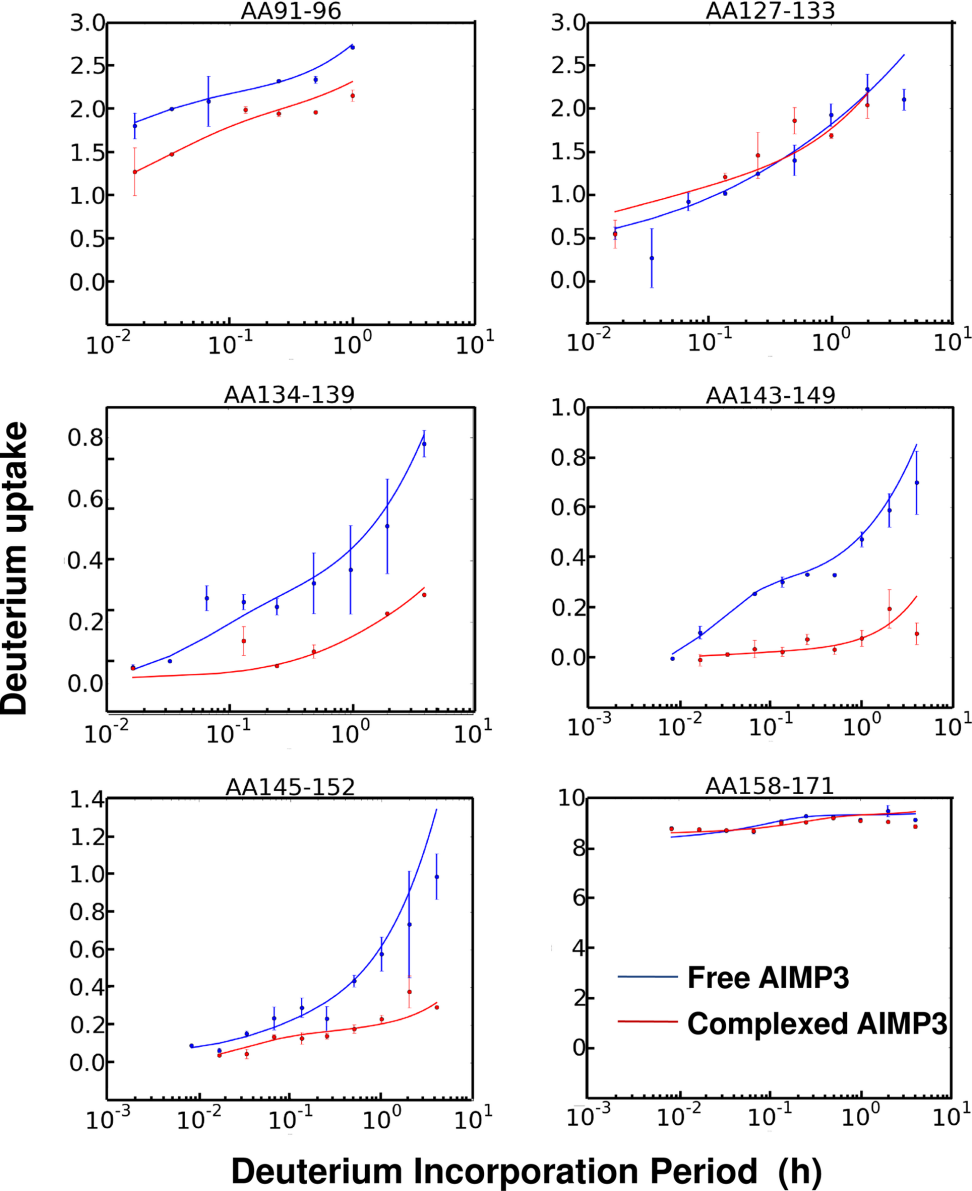
Deuterium uptake profiles (data points) and maximum-entropy fits (smooth curves [54]) vs. H/D exchange period (log_10_ scale) for selected segments of free and complexed AIMP3. Segment 91–96 of putative binding Interface I exhibits a significant decrease in D-uptake upon forming the complex. Segments 127–133 and 158–171 constitute putative Interface II, but show no change in D-uptake. Significant decreases are also observed for segments 134–139 and 143–152, thereby defining the AIMP3 binding surface to LmnA.

HDX experiments were performed for both AIMP3 alone and with LmnA. D-uptake of each peptide was calculated and the common proteolytic peptides 5–30 aa in length are compared. Because HDX rate is highly sensitive to the structure of the protein, binding to ligands affects the amide exchange at protein surfaces; therefore HDX can be used to identify interfaces, and localization of the exchange differences potentially maps out the interacting surface. To identify the AIMP3 segments involved in binding to LmnA, we assess the induced conformational changes by calculating ARDD of each common peptide (Fig 5). The only significant decreases in D-uptake are observed from the ARDD map for Interface I at segments A_91_IVQQW_96_, N_134_FTLAD_139_ and L_143_YYGLHR_149_ (Fig 6). The rest of the protein remains unaffected. A previous structure model also shows that binding to MRS or EPRS imposes little change in the global conformation of AIMP3 [18]. Thus, AIMP3 does not appear to substantially change its global conformation to facilitate binding. Rather, AIMP3 binds through its solvent-accessible regions without causing much change in the rest of the protein conformation. Although the issue of whether both segments (N_134_FTLAD_139_ and L_143_YYGLHR_149_) participate in binding, or one segment binds and the other changes its conformation due to the binding is still open to discussion, our result narrows down the binding surface of AIMP3 to a specific area in the protein.

**Fig 7:**
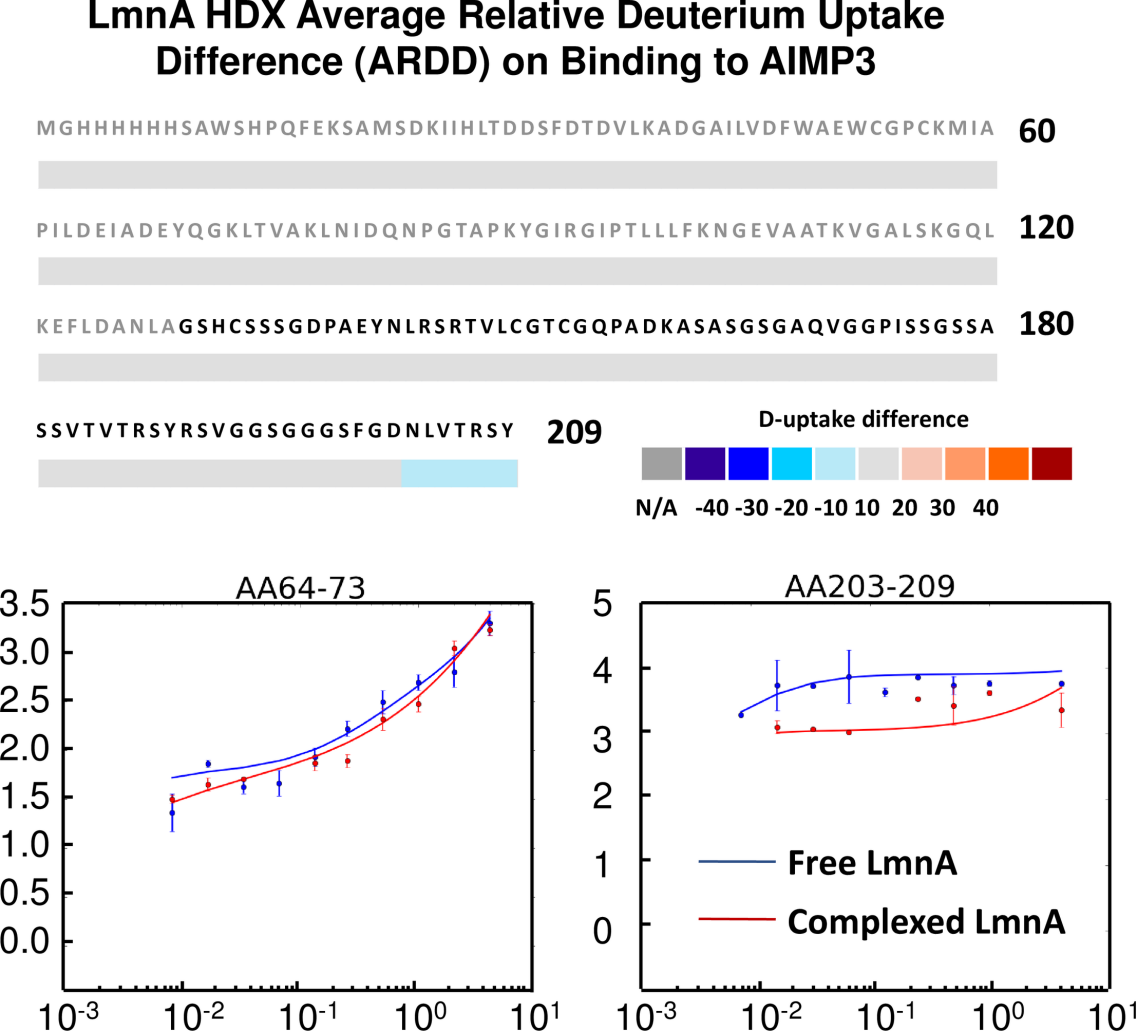
H/D exchange results for free and complexed LmnA with AIMP3. ARDD for LmnA is calculated as for AIMP3. Top: LmnA shows decreased D-uptake for segment 203–209 (641–647) upon binding AIMP3. Bottom: Deuterium uptake profiles (data points) and maximum-entropy fits (smooth curves [54]) vs. H/D exchange period (log_10_ scale) for selected segments of free and complexed LmnA. Left: Selected peptide 64–73, representing peptides in the His-Strep-TrxA tag, shows unaltered deuteration level. Right: Peptide 203–209 (641–647) shows significantly decreased D-uptake upon binding AIMP3.

Next, we examined the D-uptake difference for LmnA and LmnA complexed with AIMP3, and identified regions with altered deuteration level. The LmnA C-terminus exhibits unperturbed deuteration, except for peptide N_203_LVTRSY_209_ (641–647 for full-length LmnA), indicating that the extreme C-terminus is responsible for binding AIMP3 (Fig 7). The deuteration heat map reveals that the C-terminus of LmnA is unstructured with relatively higher deuteration level than the His-Strep-TrxA tag, suggesting that its flexibility is important for the binding process, because it allows the C-terminal tail to be in close proximity with Interface I and segment 134–152 region of AIMP3.

### Implications for AIMP3-LmnA interactions in the MSC complex

Accumulating evidence suggests that structurally similar glutathione transferase (GST) domains shared among AIMP3, MRS, and EPRS are responsible for binding [18]. The GST domain of AIMP3 preferentially forms a hetero-dimer with the GST domain of MRS or EPRS. Crystallographic results for AIMP3 also show that the interaction between two AIMP3 molecules with crystallographic 2-fold symmetry is similar to the monomer:monomer interaction in the GST dimer. Comparison of bio-similar proteins with GST-like domains also reveals two putative binding sites for AIMP3 [53]. The binding sites were further validated in our crystal structure of the heterotetrameric GST domains of AIMP3, MRS, and EPRS, thereby elucidating the interaction between them [18]. In the yeast system, the AIMP homolog, Arc1p, binds to ERS (glutamate-tRNA synthetase) and MRS to form a ternary MRS:Arc1p:ERS complex [55]. The binding site of Arc1p in the MRS:Arc1p:ERS complex is similar to that of human MRS:AIMP3:EPRS at interface I and interface II. Interface I of AIMP3 participates in binding the GST domain of human MRS, whereas Interface II is involved in complex formation with the GST domain of EPRS. Mutagenesis for Gln^95^ validates its role in the interacting surface of AIMP3:MRS, and Arg166 participation in the binding between AIMP3 and EPRS.

**Fig 8:**
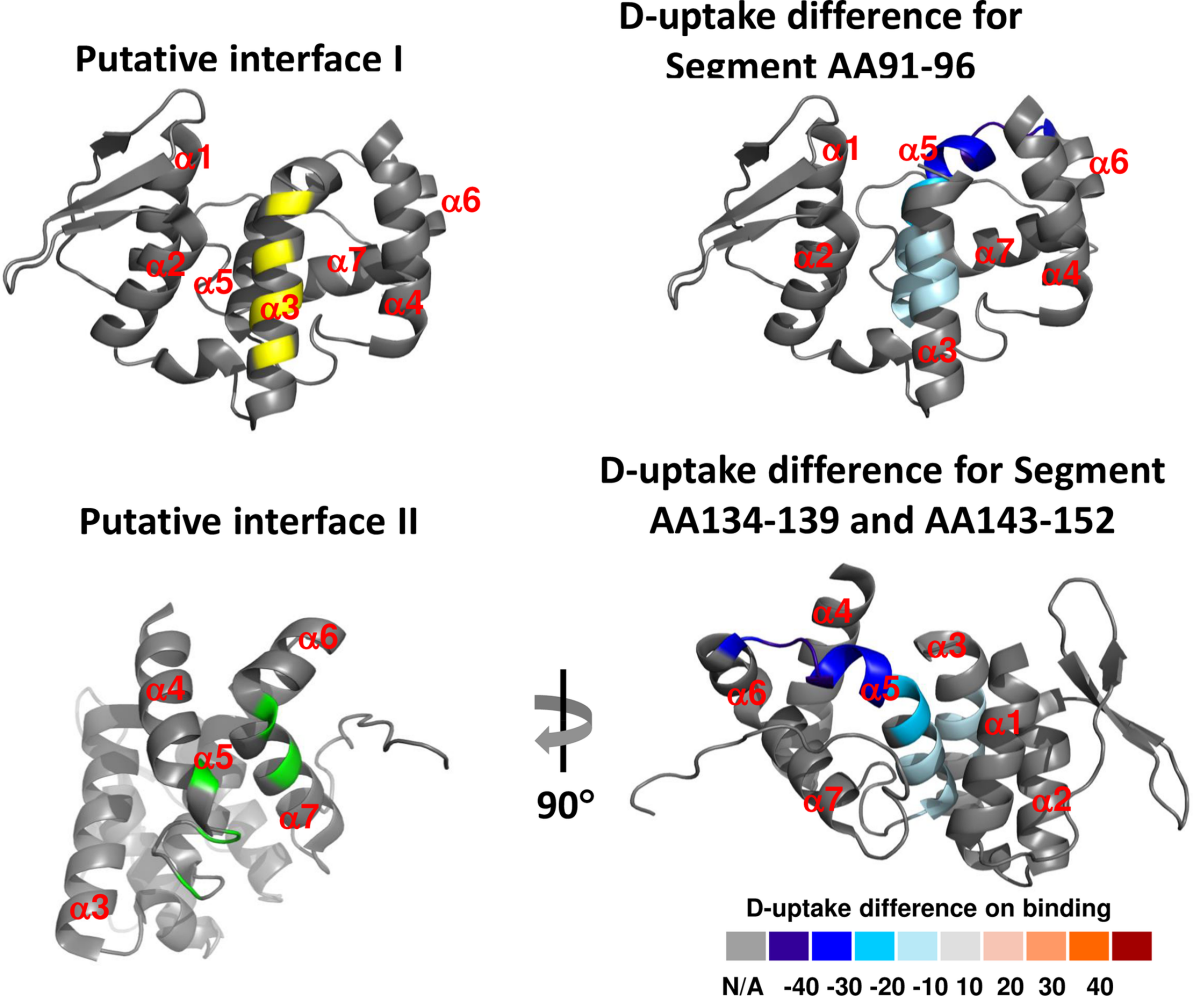
Potential binding regions mapped onto the crystal structure of AIMP3. Upper left: Interface I in yellow, including residues Arg^50^, Thr^68^, Lys^75^, Ala^91^, Gln^94^, Gln^95^, Glu^98^, and Asp^119^. Upper right: Segment 91–96 with significant decrease in D-uptake, overlapping with putative binding interface I. Lower left: Putative binding Interface II in green, including residues Glu^125^, Val^128^, Tyr^129^, Tyr^133^, Leu^162^, Arg^166^, and Phe^186^, does not exhibit any D-uptake change. Lower right: Novel binding site including segments N_134_FTLAD_139_, and L_143_YYGLHRFIV_152_. Color codes for the residues represent Average Relative D-uptake difference (see Eq 1).

Among the AIMP3 segments showing decrease in D-uptake upon binding LmnA, segment A_91_IVQQW_96_ corresponds to the previously identified binding interface I, (known to bind MRS), which includes residues Ala^91^, Gln^94^, Gln^95^, Glu^98^, and Thr^102^, and is responsible for binding to the GST domain of MRS. N_134_FTLAD_139_ and L_143_YYGLHRFIV_152,_ on the other hand, do not overlap with putative binding interface II, but are on a different side of the protein surface compared to E_125_DKVY_129_ and N_163_VSRWFCH_170_ (Fig 8). To rationalize the HDX differences observed for both putative and non-putative binding sites upon binding LmnA, we mapped the changes onto the crystal structure of AIMP3. Although A_91_IVQQW_96_ and N_134_FTLADILLYYGLHRFIV_152_ appear to be on opposite sides of the protein surface, they are in fact in close spatial proximity. The crystal structure of AIMP3 suggests that helices α3 and α5 form a bundle-like structure with α1 and α2 of the N-terminal domain, which stabilizes the structure through hydrophobic and ion interactions [53]. Helix α5 is bent in the middle, with the N-terminal end parallel to helix α3, and the C-terminal end stretches out on the protein surface. Together with our HDX results, it is likely that LmnA makes initial contact with AIMP3 through the C-terminal end of helix α5 and the surrounding loop region, and subsequently binds fully into the hydrophobic cavity between the α3 and α5. Moreover, given the modest change in D-uptake at the extreme C-terminus of LmnA upon binding AIMP3, another possible interpretation of this result is LmnA binds to Interface I of AIMP3 and causes conformational changes on the other side of the protein, manifested by a tightening of the hydrogen bonding network in Helix α5. This conformational change is detected by HDX but not by X-ray crystallography because the former simultaneously measures both tertiary structure solvent accessibility and secondary structure hydrogen bonding, and the latter detects the static state of the protein. Either way, our HDX results show that the contact surface of AIMP3 to LmnA includes Interface I and segment 143–152, with Interface I directly participating in binding.

The MRS-AIMP3-ERS complex shows that the two interfaces of AIMP3 are on two opposite sides of the molecule. Interface I, corresponding to MRS interaction, consists of α2, α3, and α4, whereas interface II, corresponding to ERS interaction, is formed by α7 and the loop between α4 and α5. Therefore, the LmnA interface mapped by our HDX analysis covers the middle region of interface I (α3) and possibly a novel interface (C-terminal half of α5), leaving the interface II unaffected, and thus potentially available for molecular interaction with another protein.

### Implications of AIMP3 binding specifically to mature LmnA

Previous biochemical experiments demonstrated specific binding between AIMP3 and the C-terminal tail of LmnA. Proteasome-dependent degradation induced by AIMP3 is specific to mature LmnA, but not to LmnC, prelamin A, or progerin. Co-immunoprecipitation and pull-down assays confirm the specific binding between AIMP3 and mature LmnA, but not other isoforms [7]. Examining the differences between mature LmnA and the isoforms is helpful in narrowing down the possible binding site for this interaction. LmnA and LmnC are encoded by the same gene; alternative splicing [10,11] results in a unique C-terminal region for LmnA. The fact that AIMP3 does not bind to LmnC suggests that the unique C-terminus must contain the binding site. Progerin is also incapable of binding AIMP3, and lacks 50 residues in the C-terminal region compared to LmnA, further localizing the binding site to the C-terminal 50 residues. Previously, it was difficult to explain why AIMP3 fails to bind prelamin A, because prelamin A has essentially the same sequence as mature LmnA, and the only difference is that prelamin A contains an additional 17 residues at the C-terminus, which are removed during LmnA maturation. Because our HDX data suggest that the extreme C-terminus is responsible for binding AIMP3, it is highly likely that the exposed C-terminus is critical for the binding to occur. Prelamin A loses the ability to bind AIMP3 because the capped binding site cannot access the AIMP3 binding surface.

In summary, we performed HDX experiments to elucidate the binding between AIMP3 and LmnA. Our results suggest that the extreme C-terminus (residues 640–646) of LmnA binds to the α3-α5 side (interface I and possibly a novel interface) of AIMP3. Both interfaces of AIMP3 overlap partially with the MRS-AIMP3-EPRS interface in the MSC, thus forming an on/off switch for the non-translational function of AIMP3, which is further coordinated with its nuclear translocation. Together, our results provide structural insights for understanding the function of AIMP3 in LmnA degradation.

## Supporting information

S1 Fig Optimized LC gradient. Top: Total ion chromatogram. The first 0.75 min is the desalting step, in which an isocratic flow of solvent A is connected to the MS. Bottom: Solvent composition and number of new peptides identified over the gradient. Most of the peptides elute from 3–3.5 min, followed by the undigested protein and the protease. Although a short gradient is necessary to minimize back-exchange, excellent separation is achieved and is essential for detecting low-abundance peptides at good signal-to-noise ratio.
(TIF)

S2 Fig SDS PAGE gels demonstrating the purity of AIMP3 and LmnA. (TIF)

S1 File Comparison figures of D-uptake for proteolytic peptides (5–30 aa in length) common to free His-Tev-AIMP3 and His-Tev-AIMP3 in complex. (ZIP)

S2 File Comparison figures of D-uptake for proteolytic peptides (5–30 aa in length) common to free His-Strep-TrxA-LmnA and His-Strep-TrxA-LmnA in complex. (ZIP)
