# Supplementary figures and images for: Mapping the contact surfaces in the Lamin A:AIMP3 complex by hydrogen/deuterium exchange FT-ICR mass spectrometry (part 1 of 3)
Source: PLoS One. 2017 Aug 10;12(8):e0181869. doi: 10.1371/journal.pone.0181869 (PMC5552228; doi:10.1371/journal.pone.0181869)

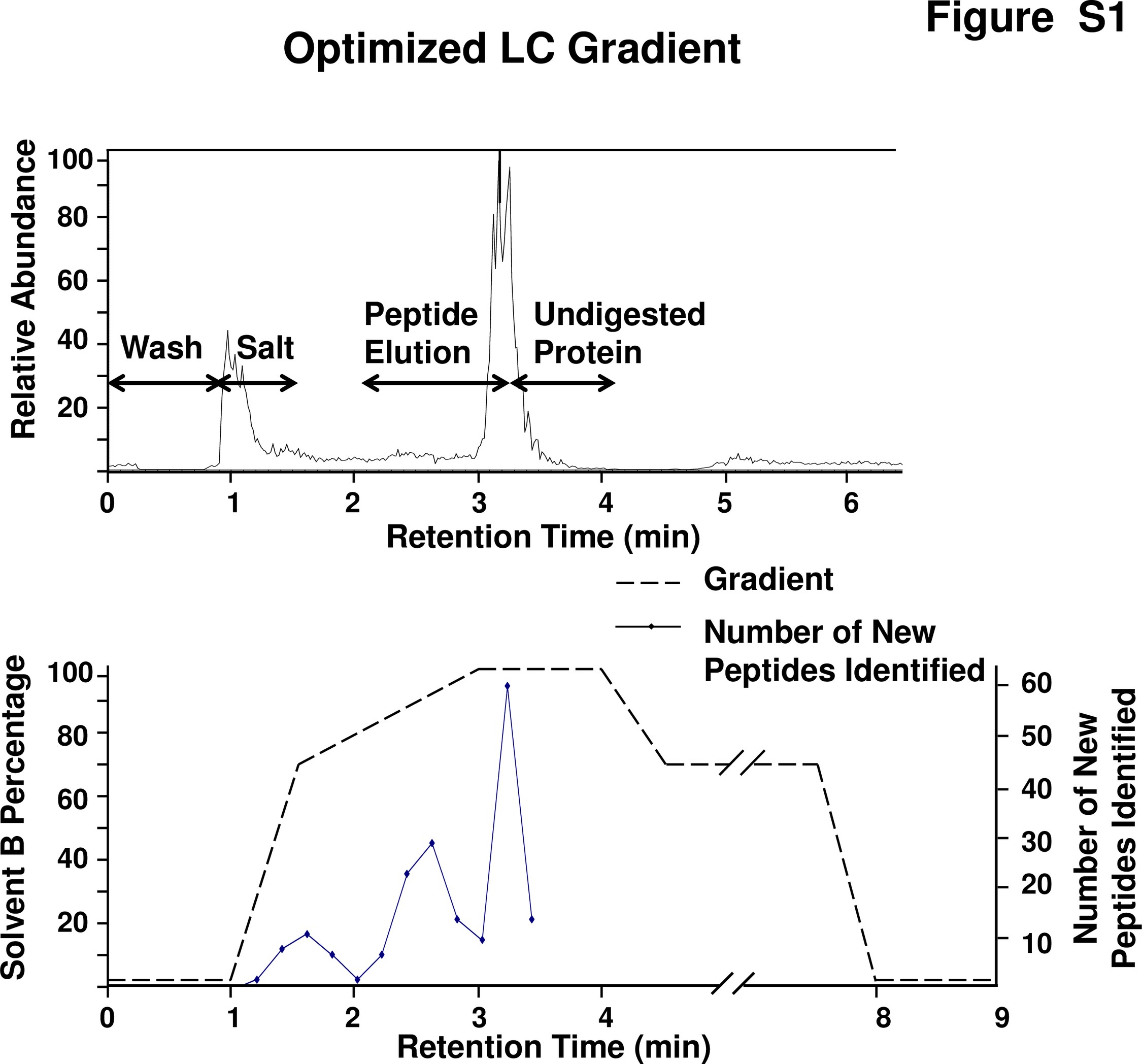

Supplement: S1 Fig — Top: Total ion chromatogram. The first 0.75 min is the desalting step, in which an isocratic flow of solvent A is connected to the MS. Bottom: Solvent composition and number of new peptides identified over the gradient. Most of the peptides elute from 3–3.5 min, followed by the undigested protein and the protease. Although a short gradient is necessary to minimize back-exchange, excellent separation is achieved and is essential for detecting low-abundance peptides at good signal-to-noise ratio. (TIF) [file pone.0181869.s001.tif]

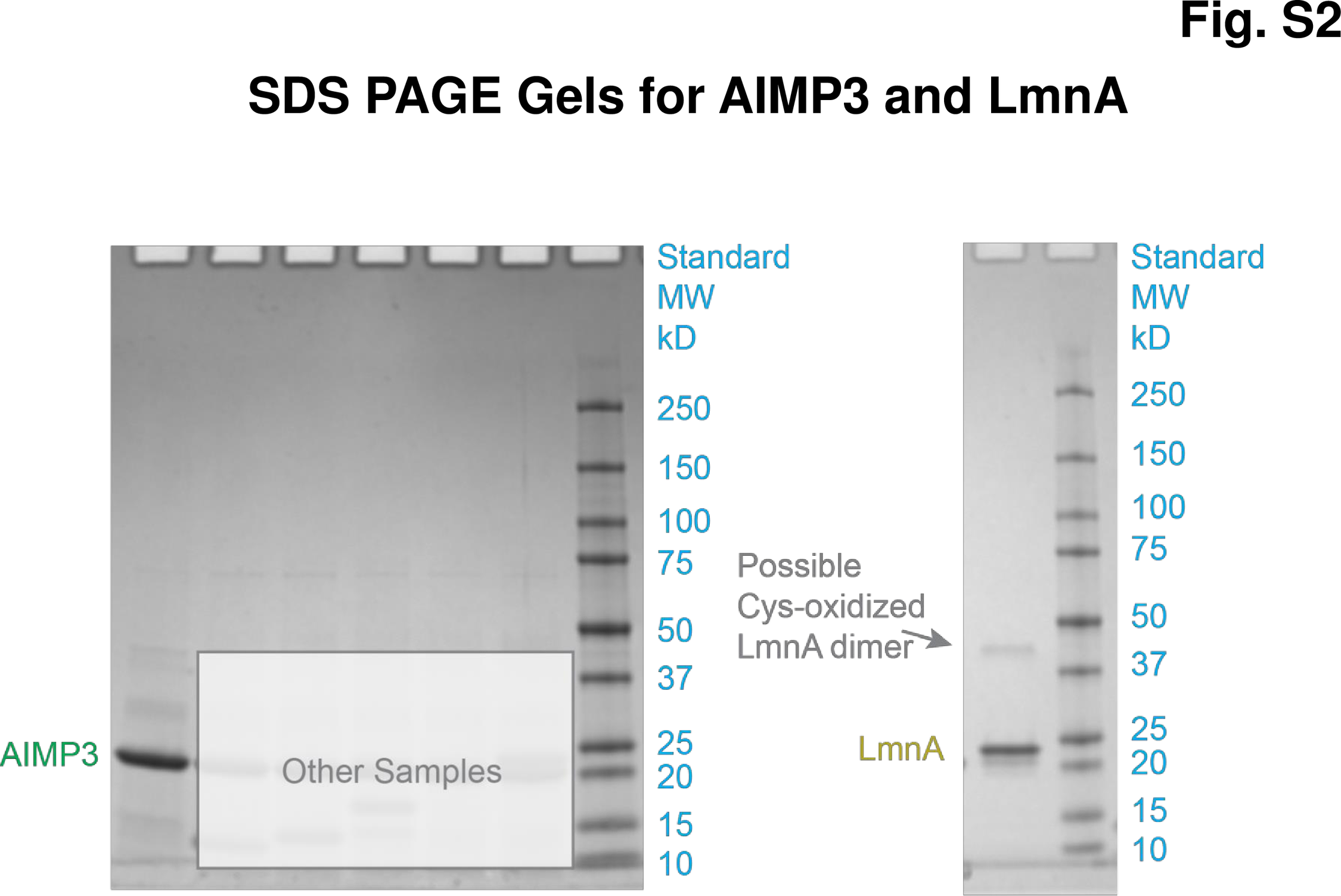

Supplement: S2 Fig — (TIF) [file pone.0181869.s002.tif]

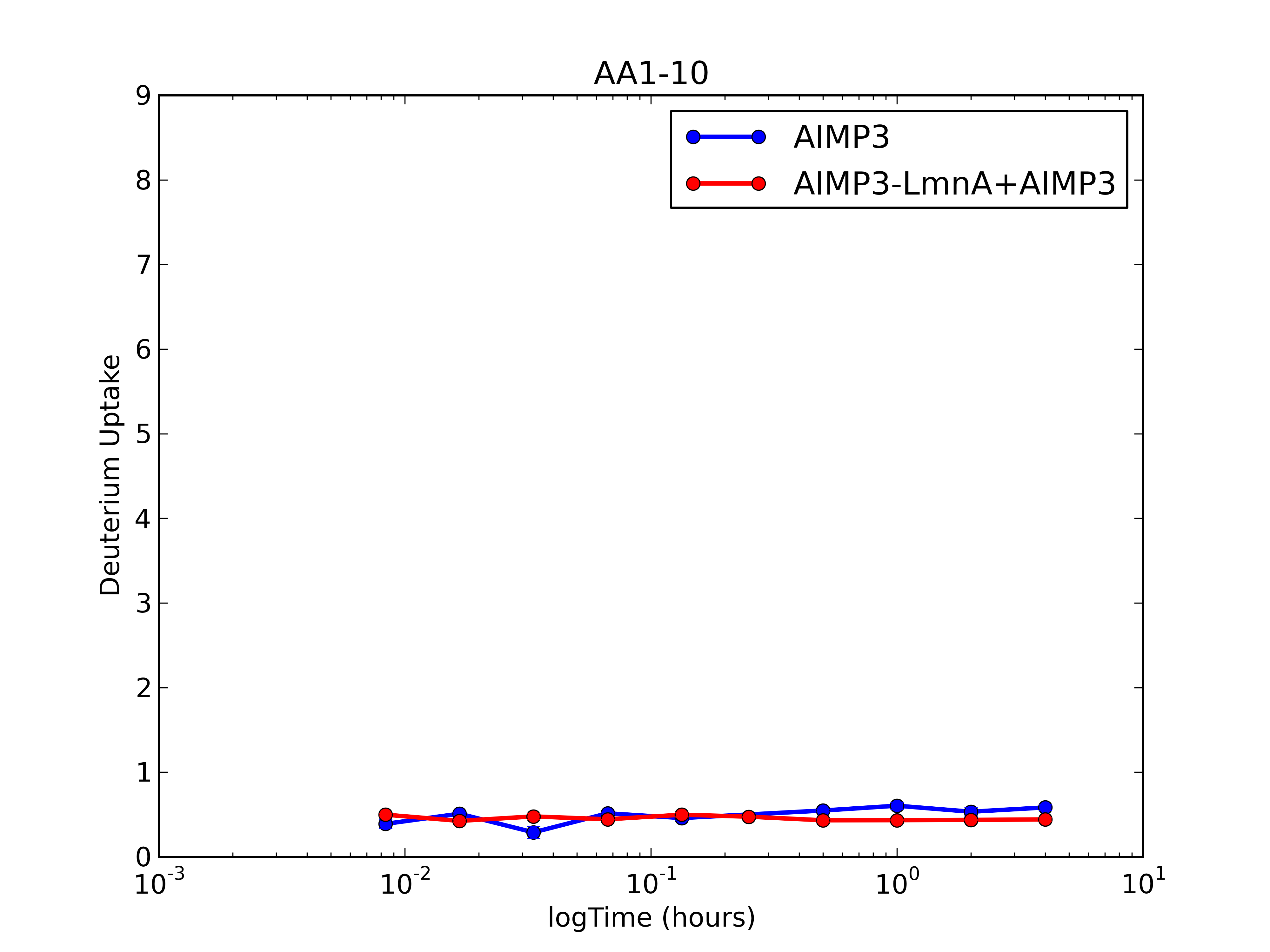

Supplement: S1 File — (ZIP) [file pone.0181869.s003.zip › logfigure-AIMP3-scale/AA1-10_charge_3_mz410.5.csv.csv.png]

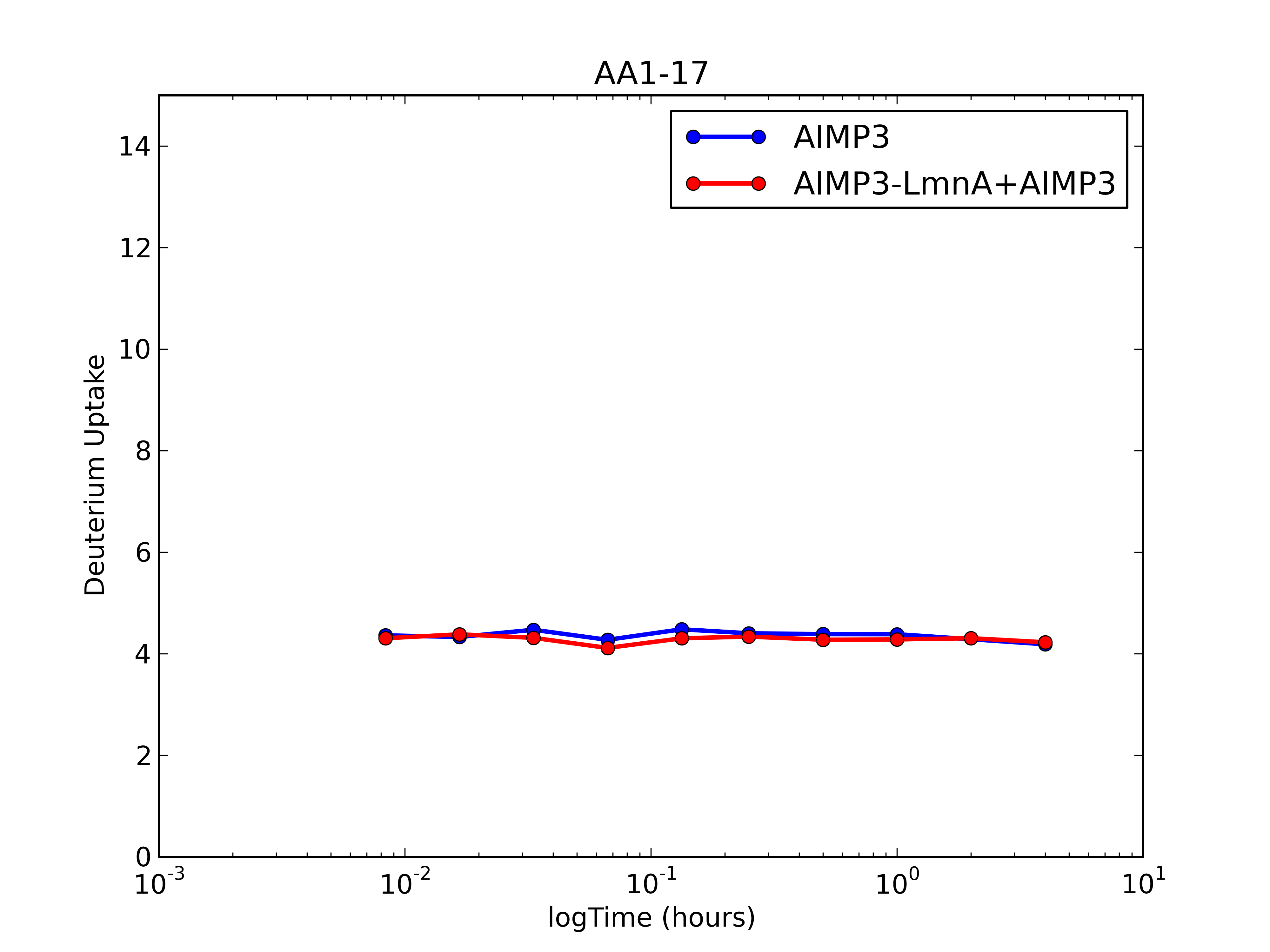

Supplement: S1 File — (ZIP) [file pone.0181869.s003.zip › logfigure-AIMP3-scale/AA1-17_charge_4_mz507.0.csv.csv.png]

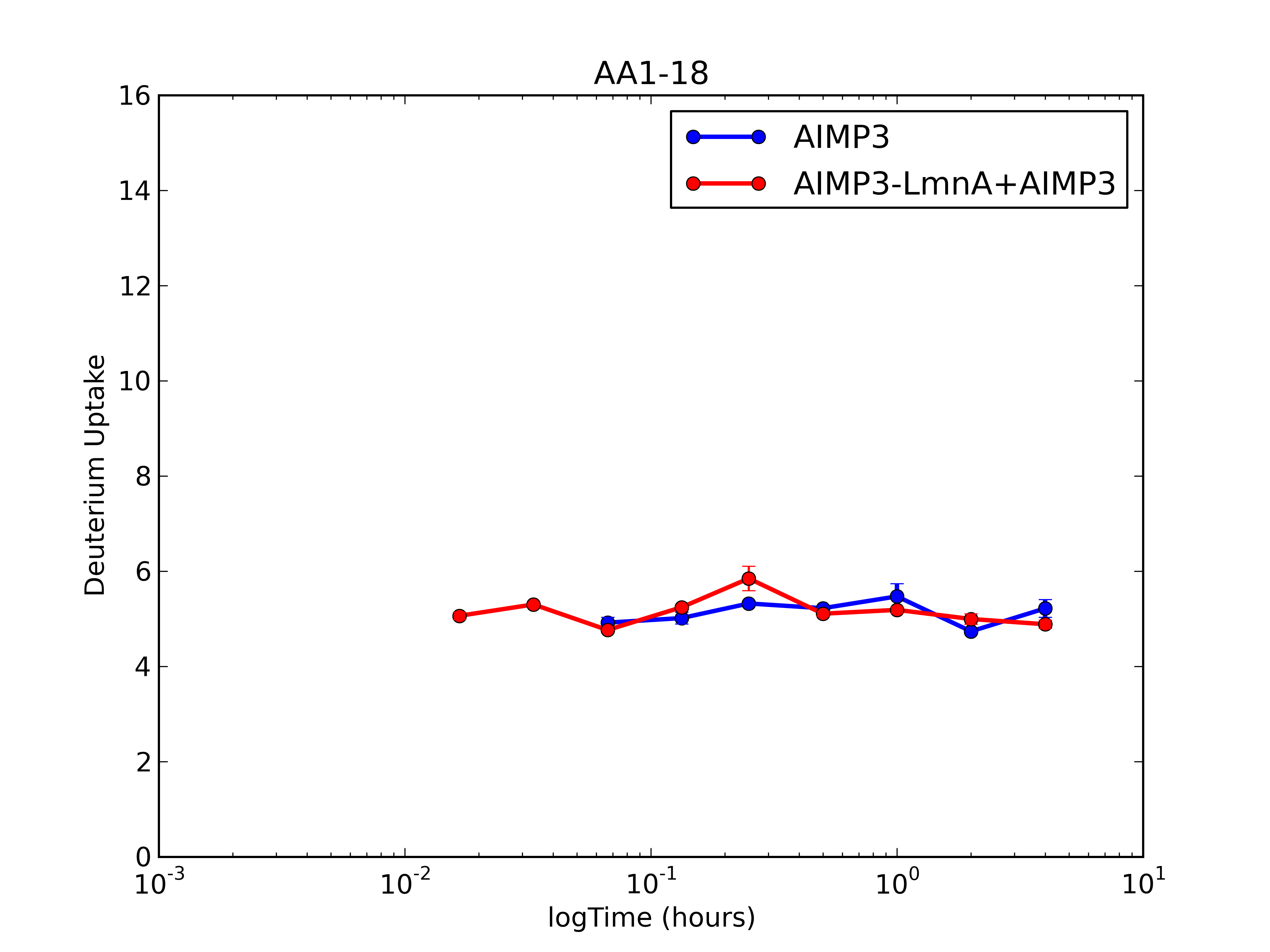

Supplement: S1 File — (ZIP) [file pone.0181869.s003.zip › logfigure-AIMP3-scale/AA1-18_charge_3_mz730.0.csv.csv.png]

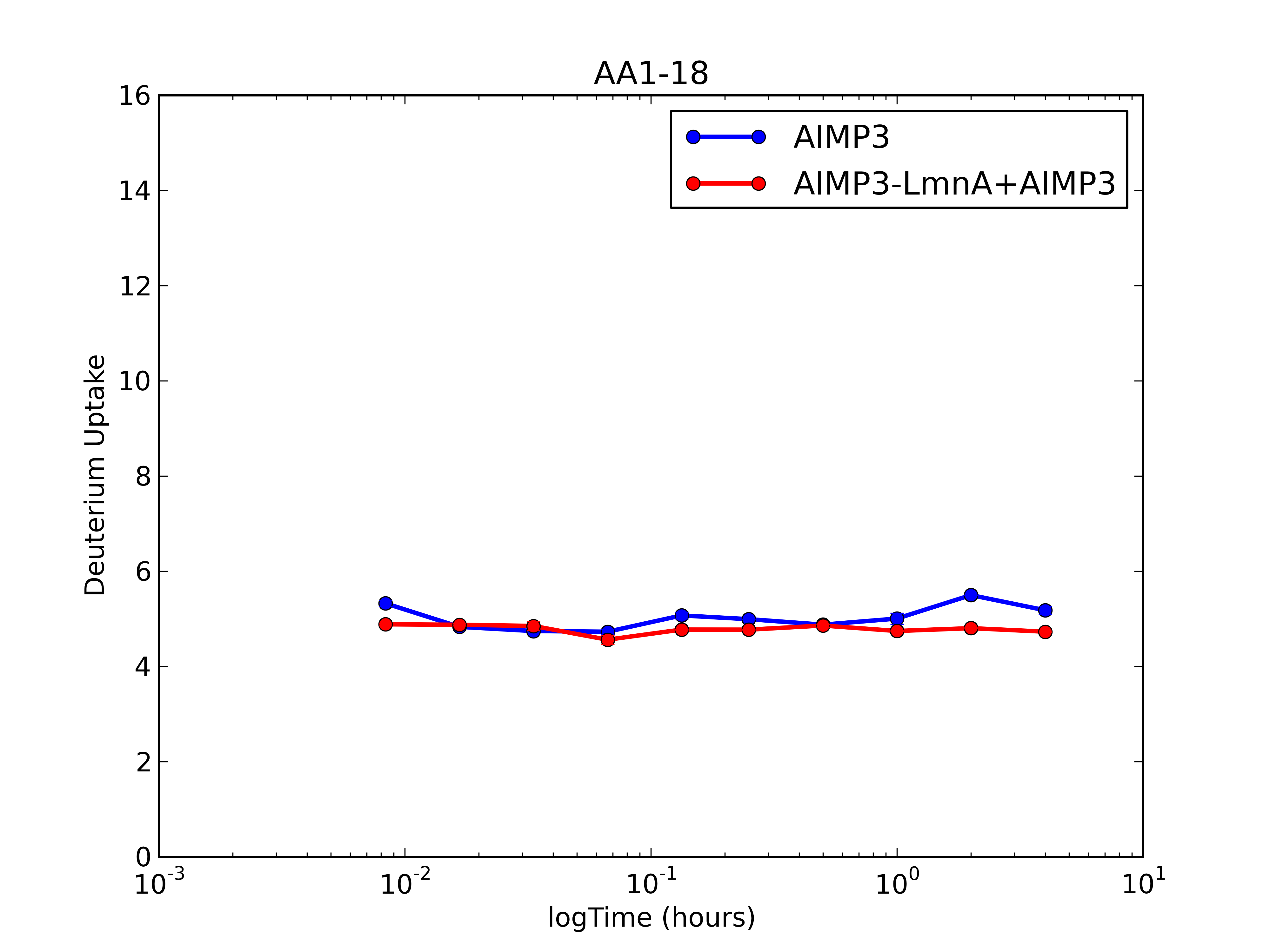

Supplement: S1 File — (ZIP) [file pone.0181869.s003.zip › logfigure-AIMP3-scale/AA1-18_charge_4_mz547.7.csv.csv.png]

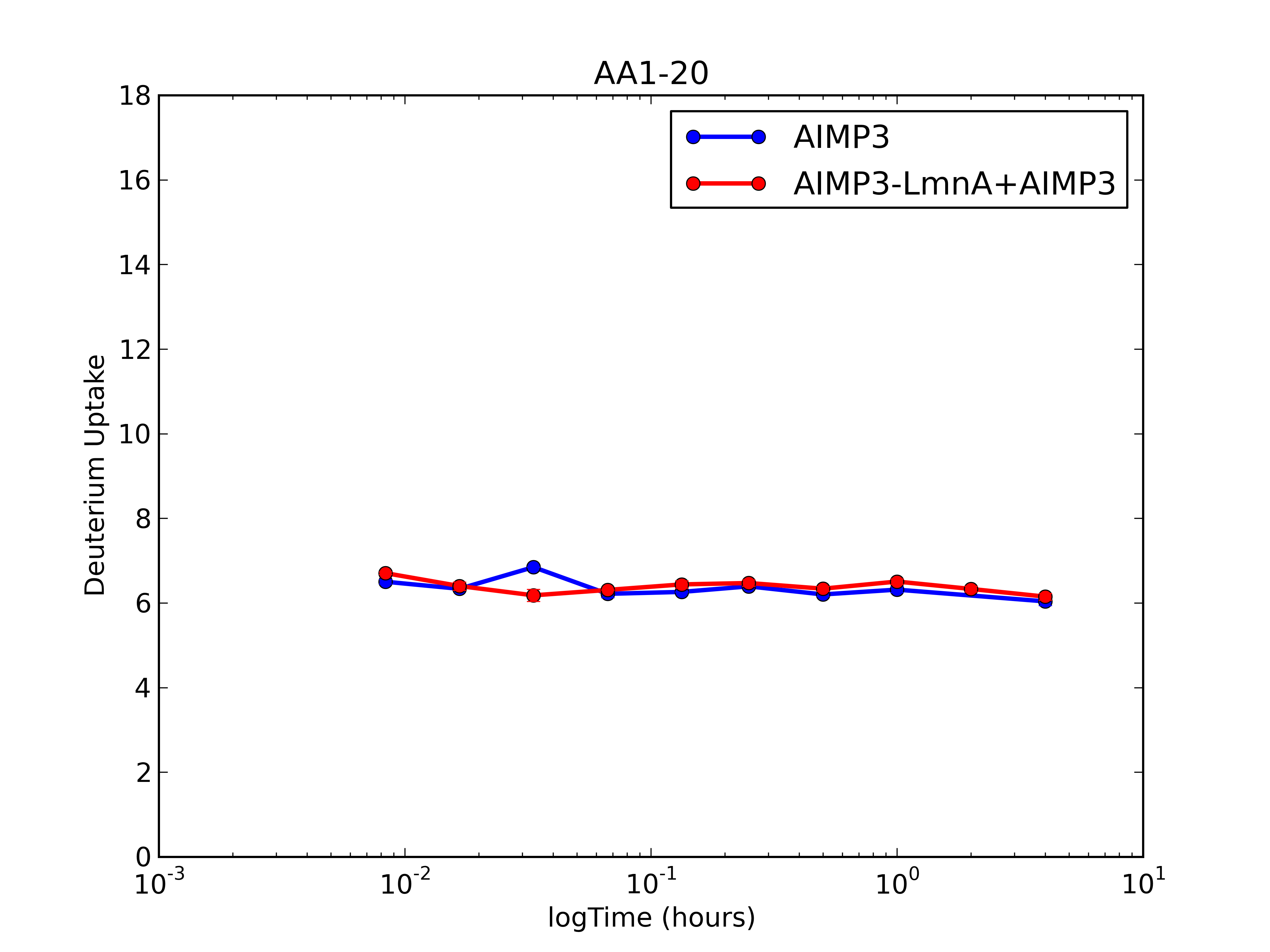

Supplement: S1 File — (ZIP) [file pone.0181869.s003.zip › logfigure-AIMP3-scale/AA1-20_charge_4_mz616.5.csv.csv.png]

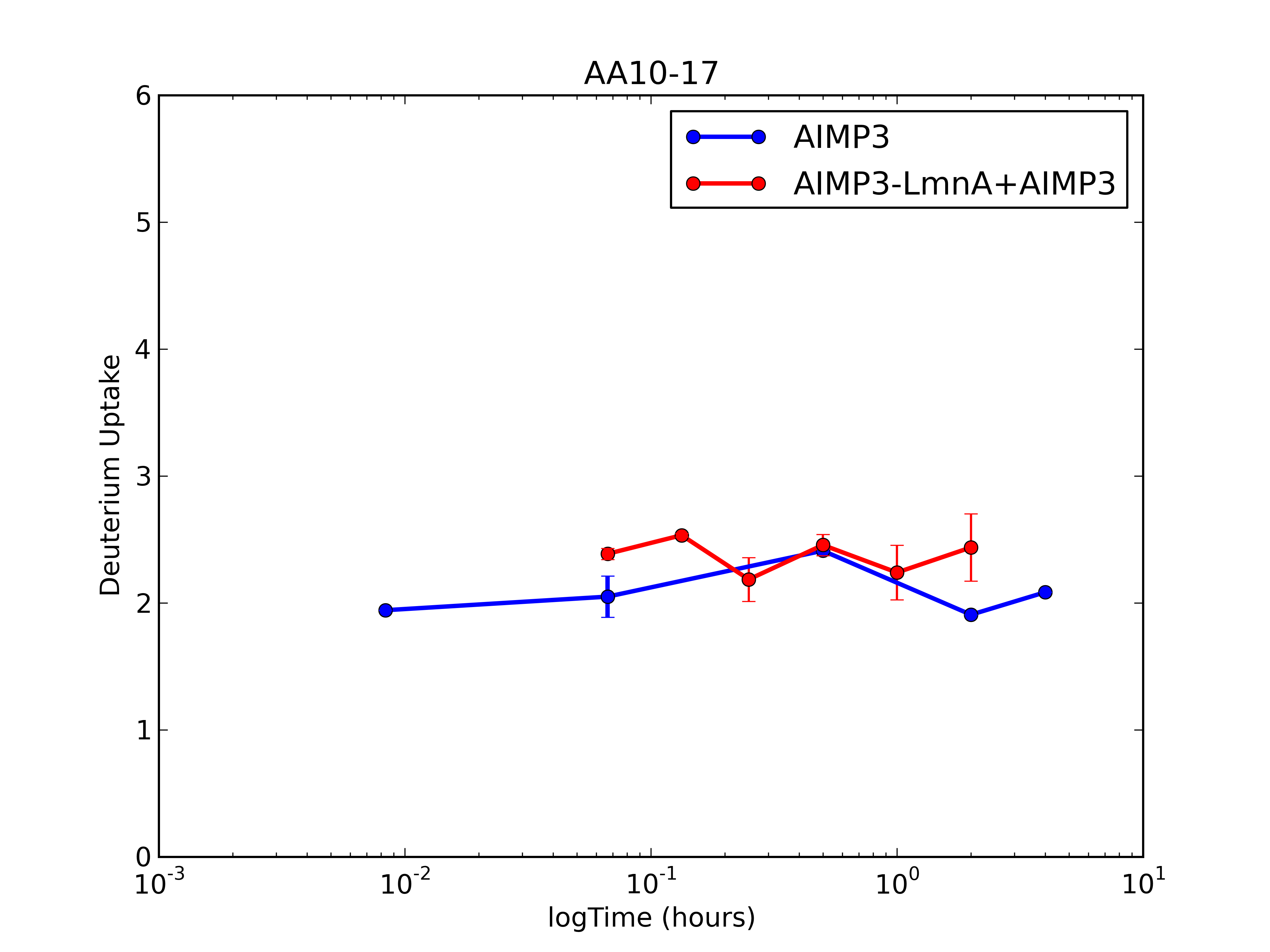

Supplement: S1 File — (ZIP) [file pone.0181869.s003.zip › logfigure-AIMP3-scale/AA10-17_charge_2_mz464.2.csv.csv.png]

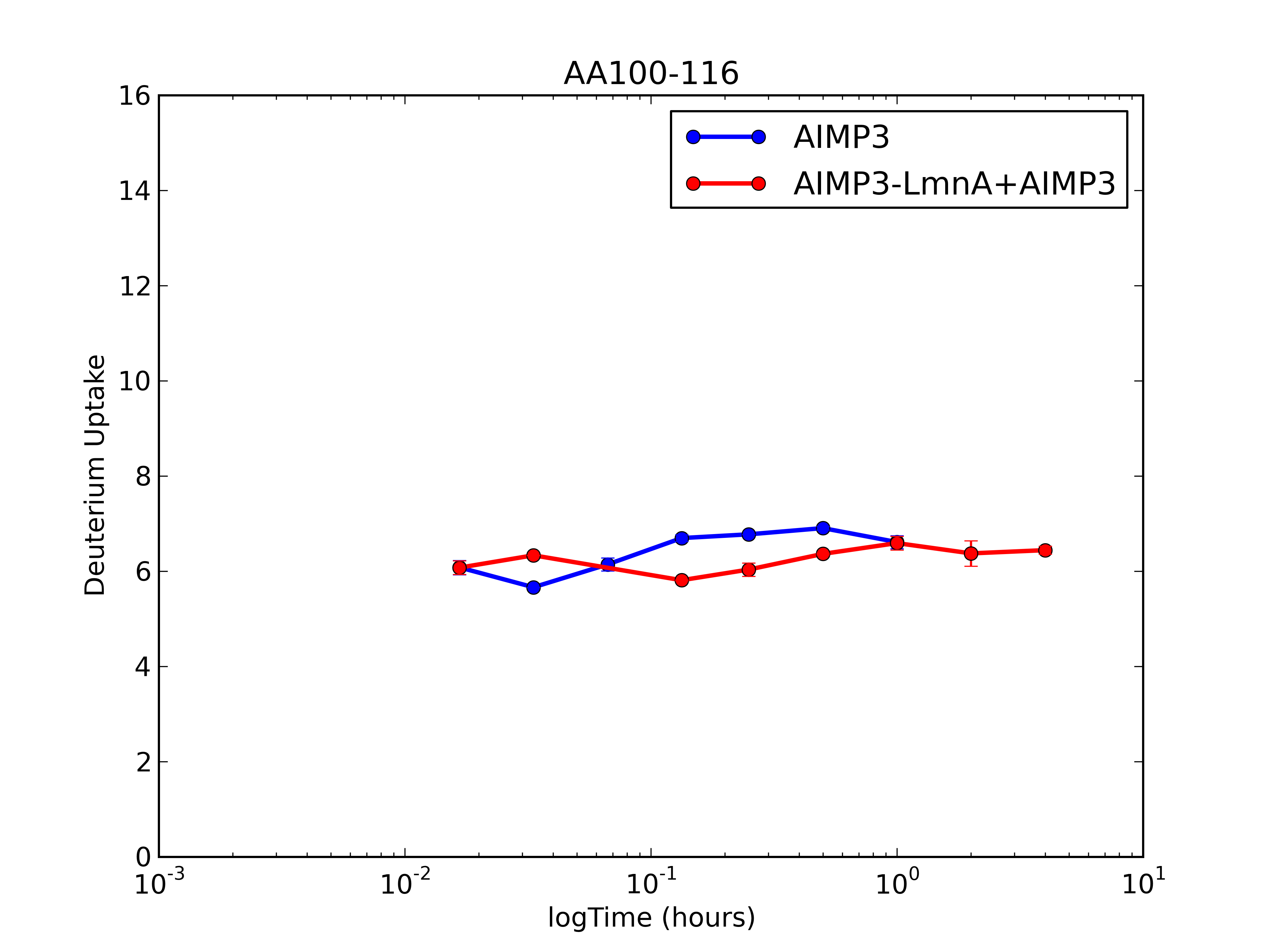

Supplement: S1 File — (ZIP) [file pone.0181869.s003.zip › logfigure-AIMP3-scale/AA100-116_charge_2_mz953.9.csv.csv.png]

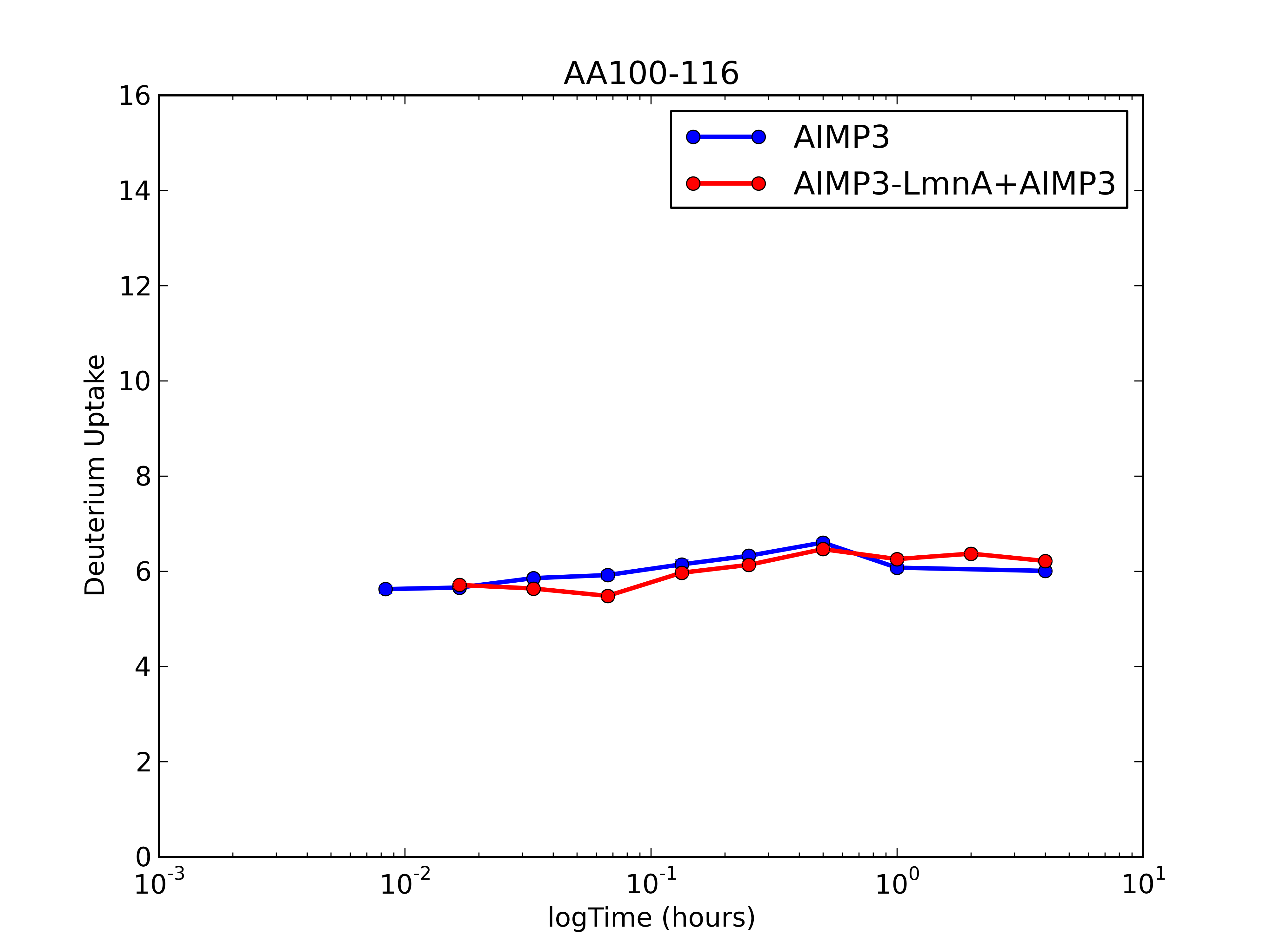

Supplement: S1 File — (ZIP) [file pone.0181869.s003.zip › logfigure-AIMP3-scale/AA100-116_charge_3_mz636.3.csv.csv.png]

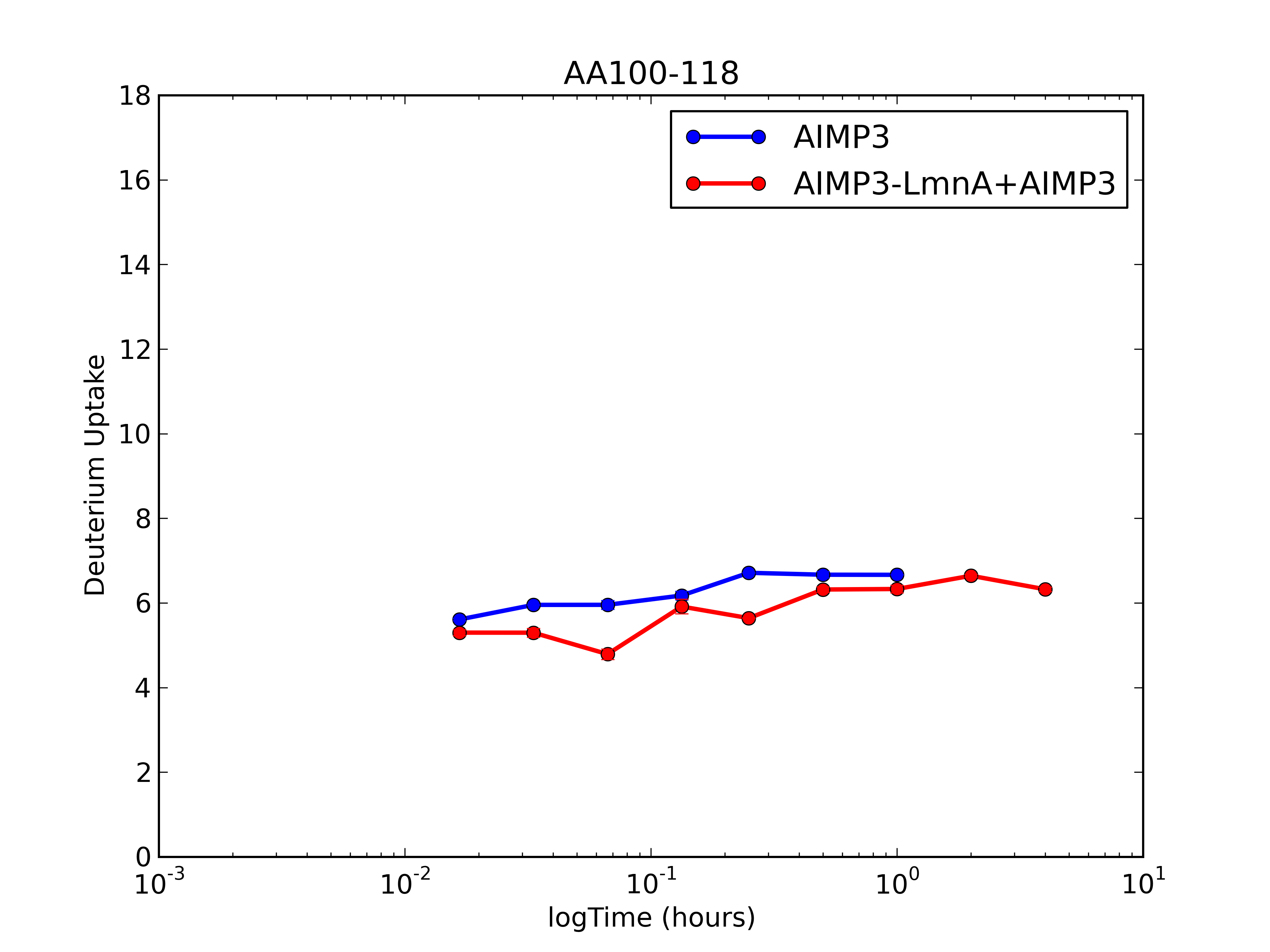

Supplement: S1 File — (ZIP) [file pone.0181869.s003.zip › logfigure-AIMP3-scale/AA100-118_charge_3_mz716.7.csv.csv.png]

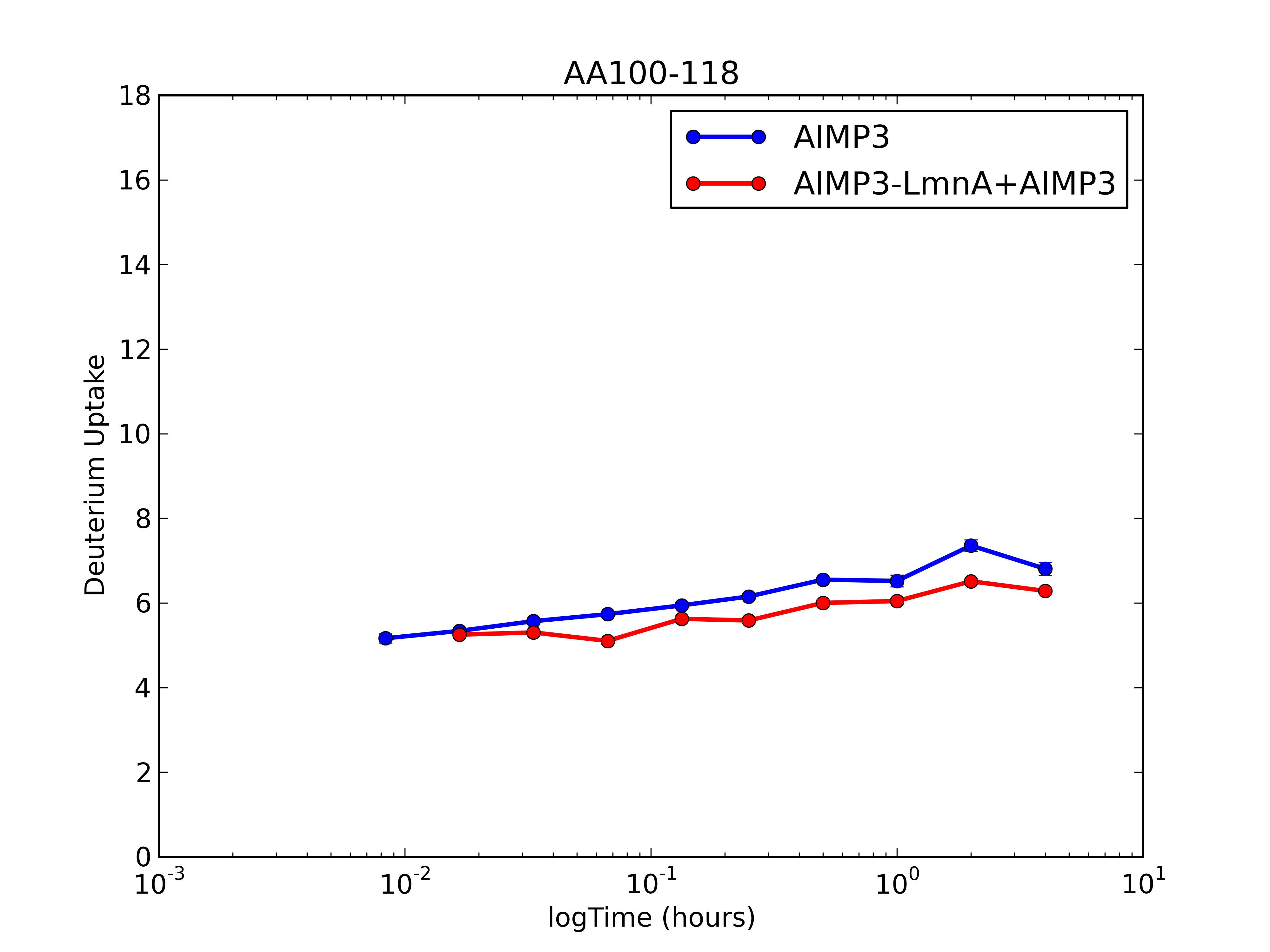

Supplement: S1 File — (ZIP) [file pone.0181869.s003.zip › logfigure-AIMP3-scale/AA100-118_charge_4_mz537.7.csv.csv.png]

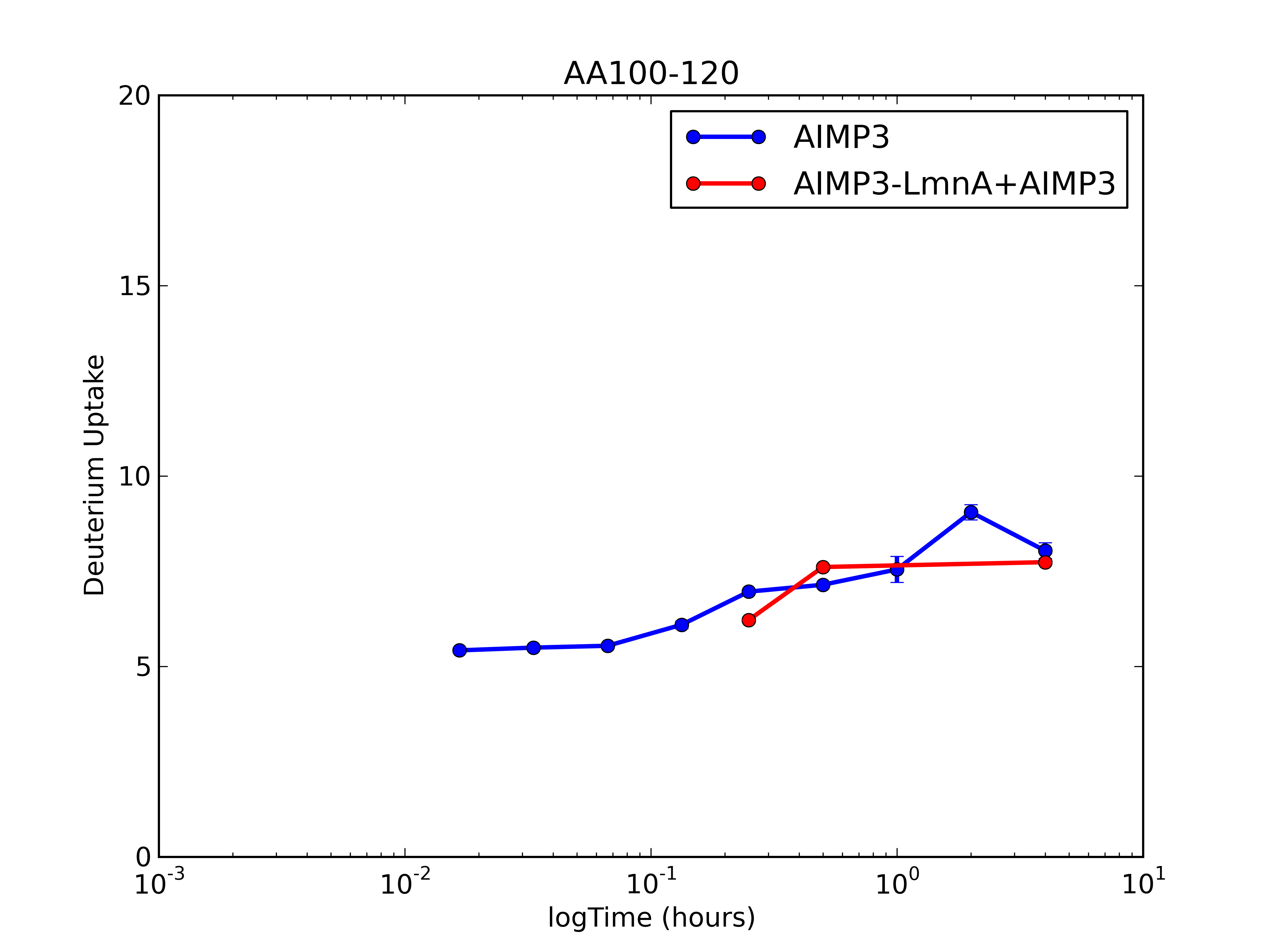

Supplement: S1 File — (ZIP) [file pone.0181869.s003.zip › logfigure-AIMP3-scale/AA100-120_charge_4_mz594.8.csv.csv.png]

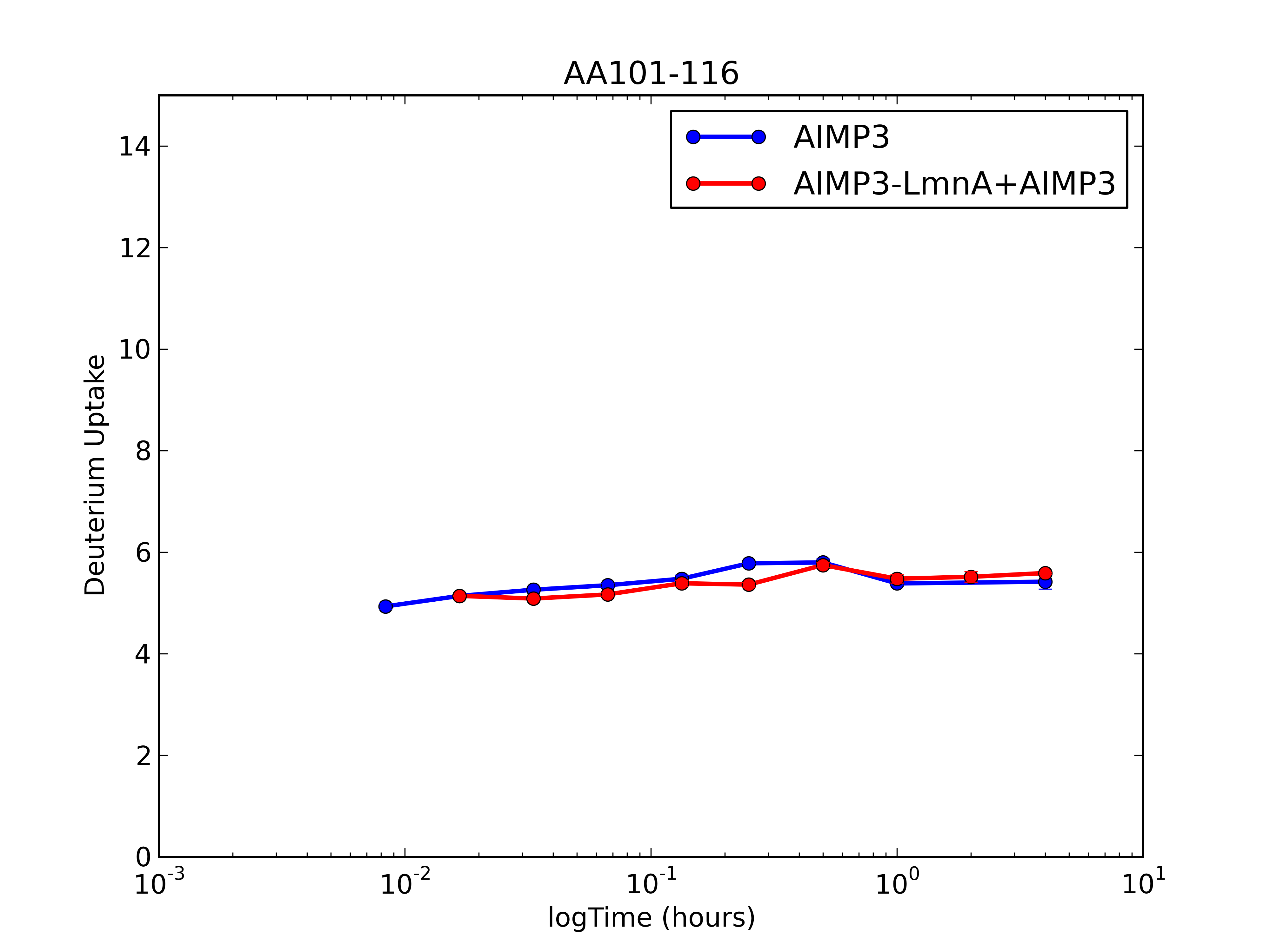

Supplement: S1 File — (ZIP) [file pone.0181869.s003.zip › logfigure-AIMP3-scale/AA101-116_charge_3_mz584.2.csv.csv.png]

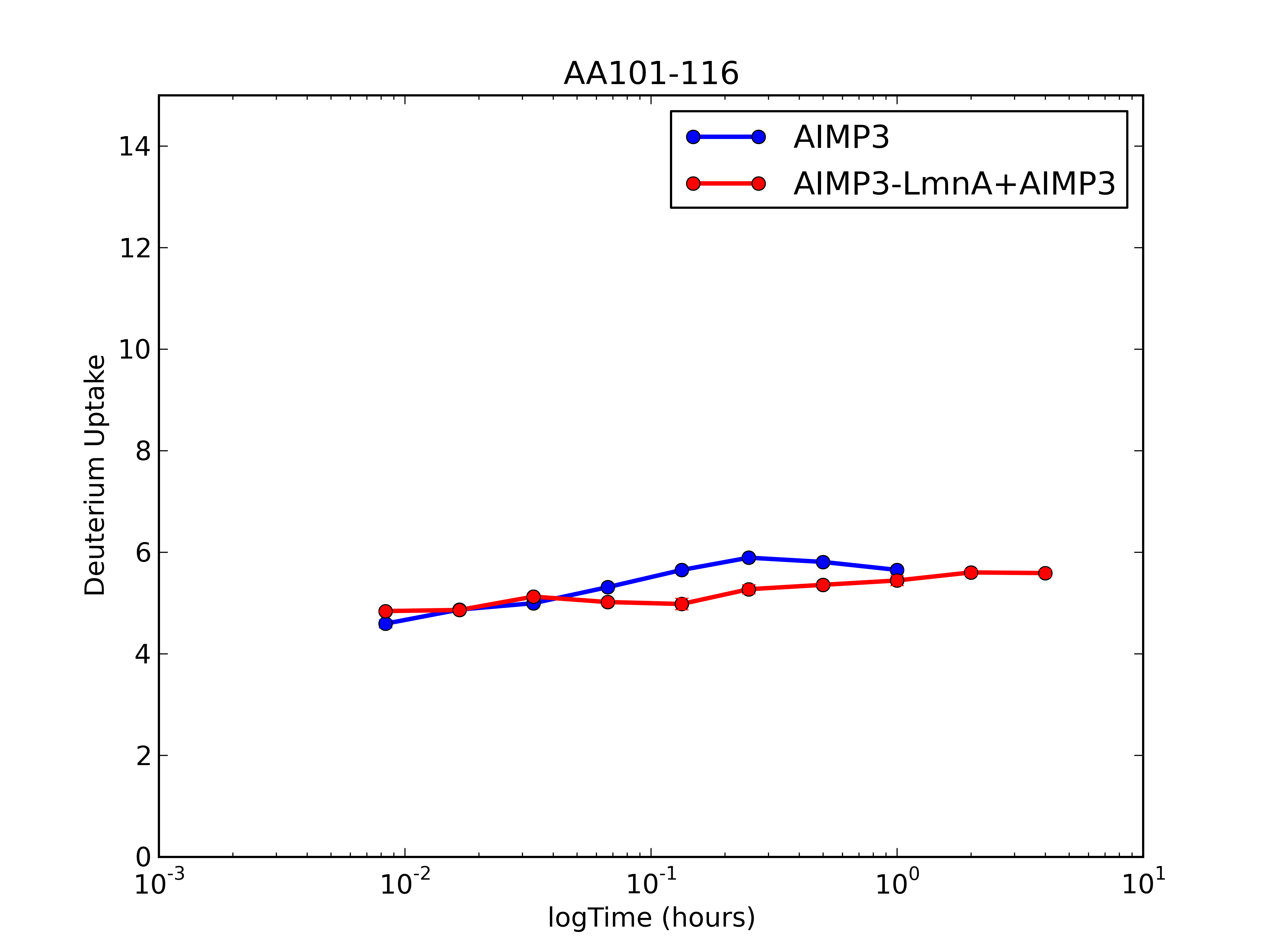

Supplement: S1 File — (ZIP) [file pone.0181869.s003.zip › logfigure-AIMP3-scale/AA101-116_charge_4_mz438.4.csv.csv.png]

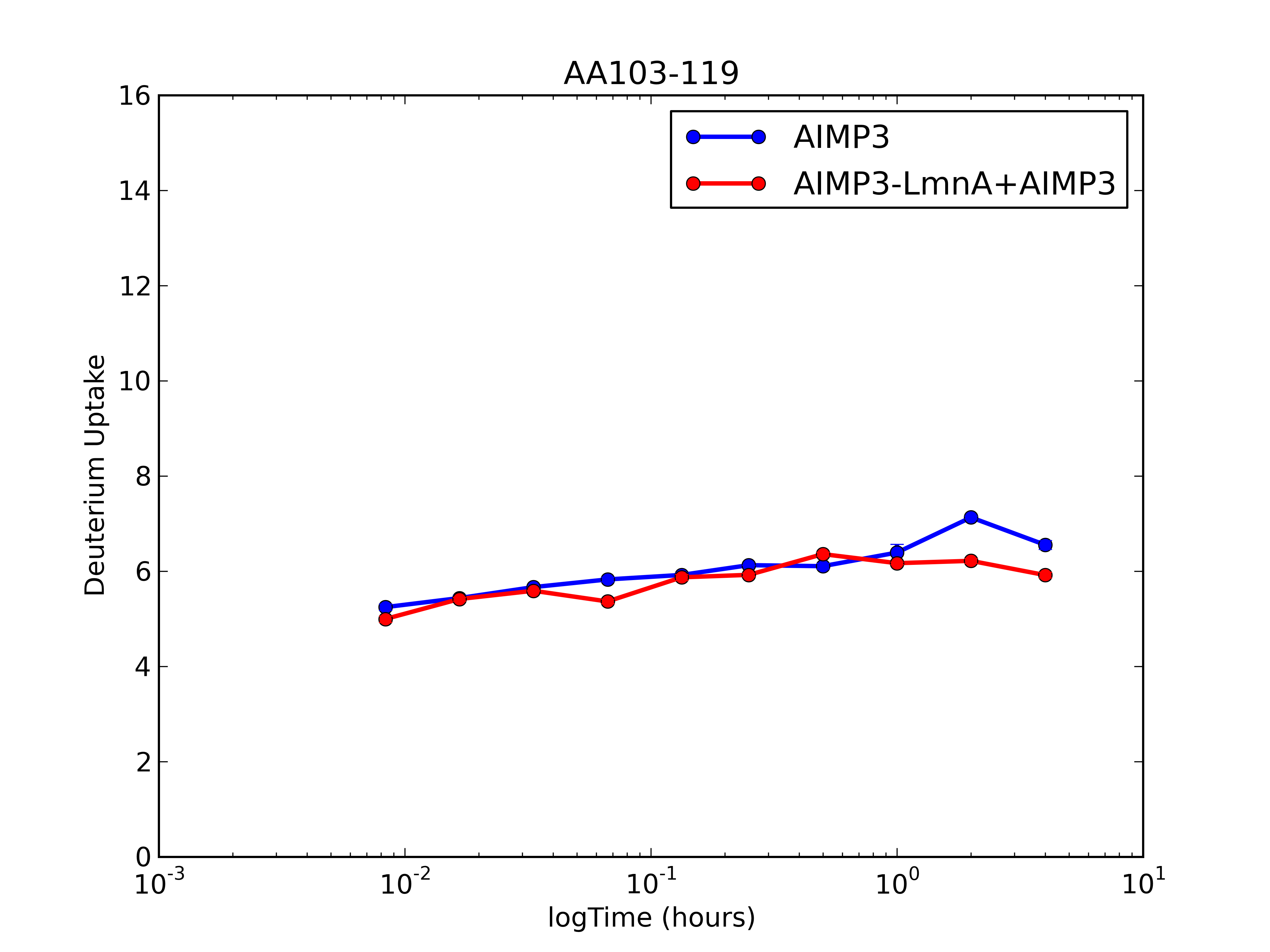

Supplement: S1 File — (ZIP) [file pone.0181869.s003.zip › logfigure-AIMP3-scale/AA103-119_charge_4_mz477.4.csv.csv.png]

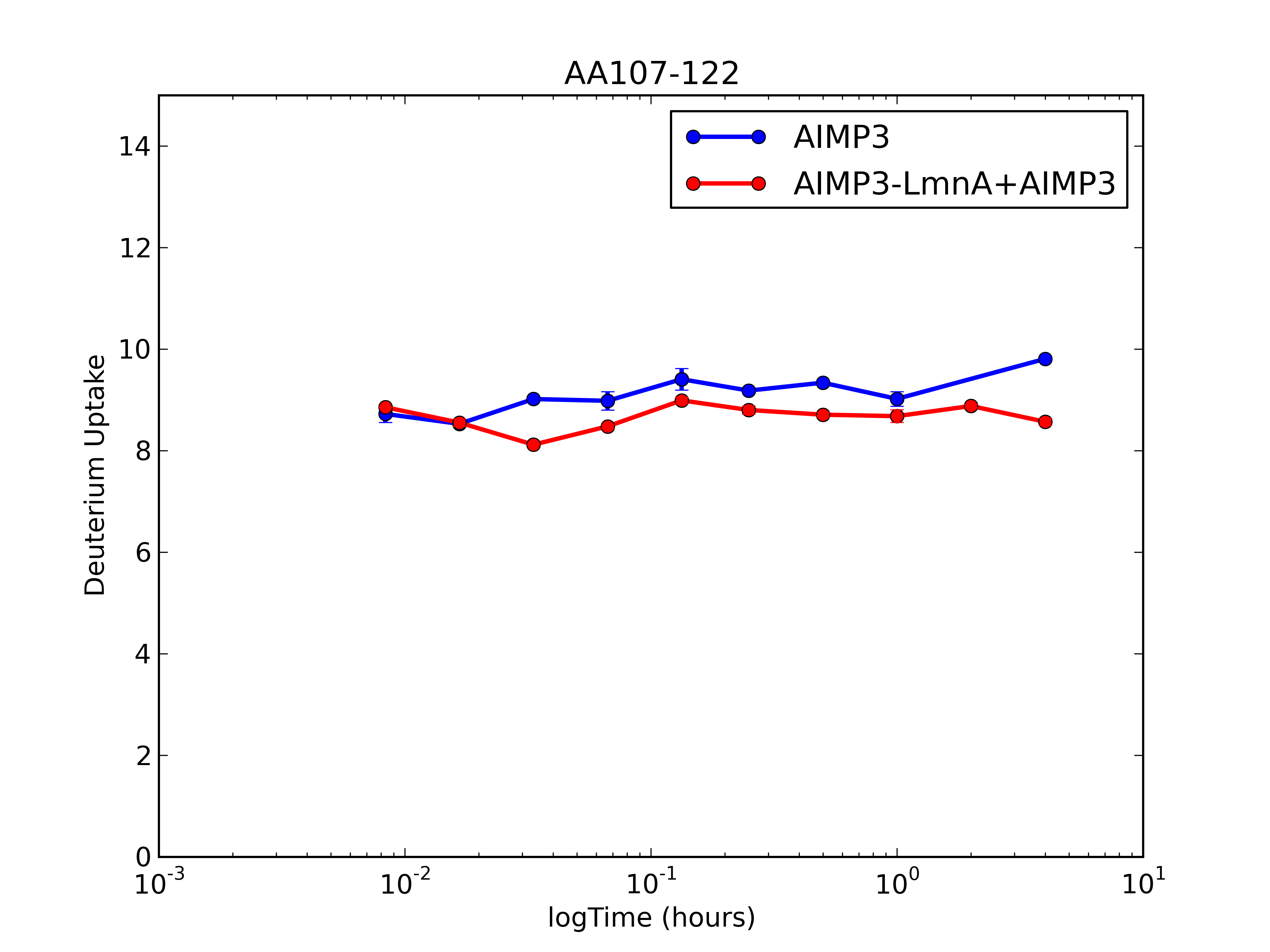

Supplement: S1 File — (ZIP) [file pone.0181869.s003.zip › logfigure-AIMP3-scale/AA107-122_charge_4_mz456.2.csv.csv.png]

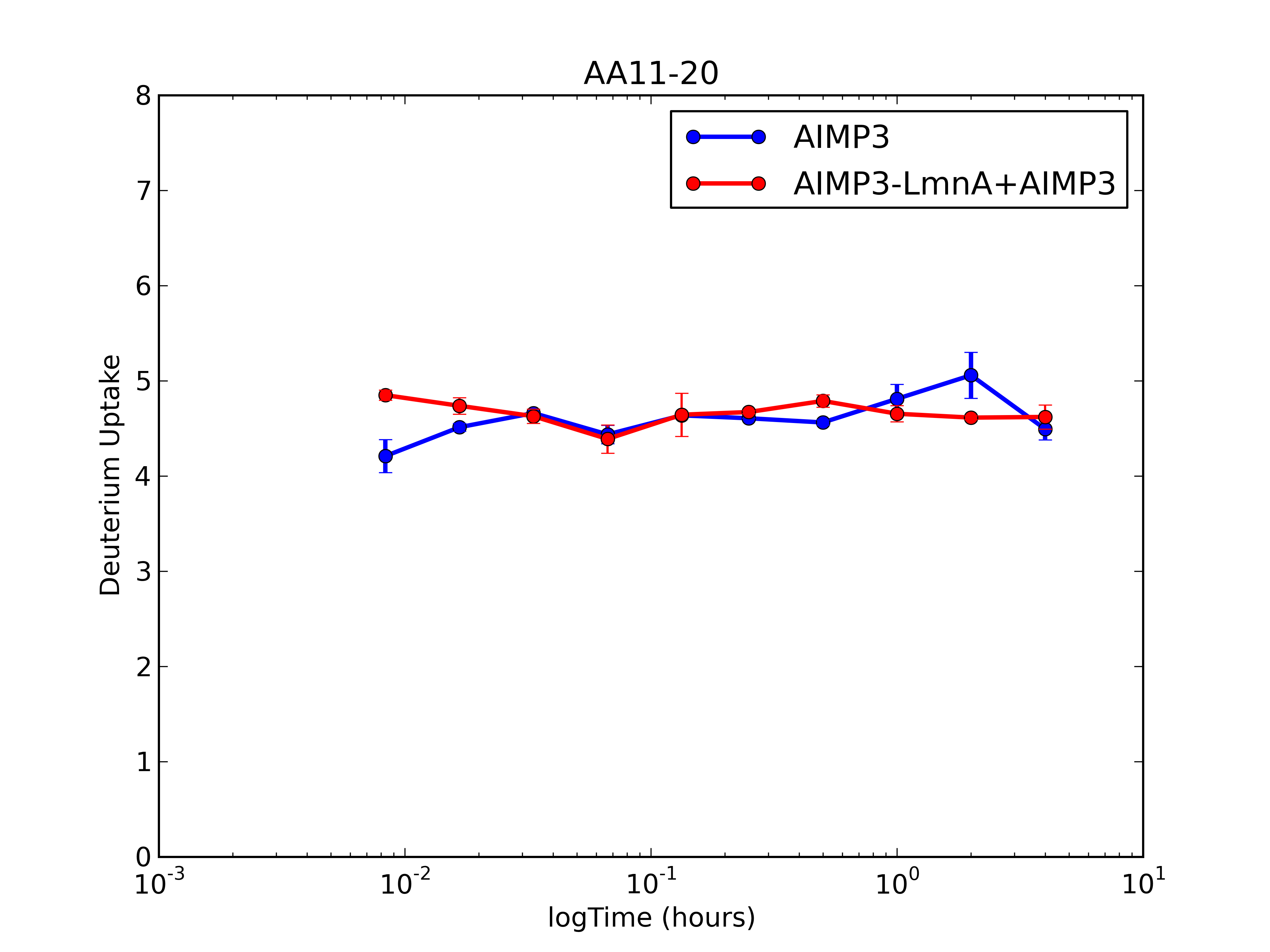

Supplement: S1 File — (ZIP) [file pone.0181869.s003.zip › logfigure-AIMP3-scale/AA11-20_charge_2_mz626.8.csv.csv.png]

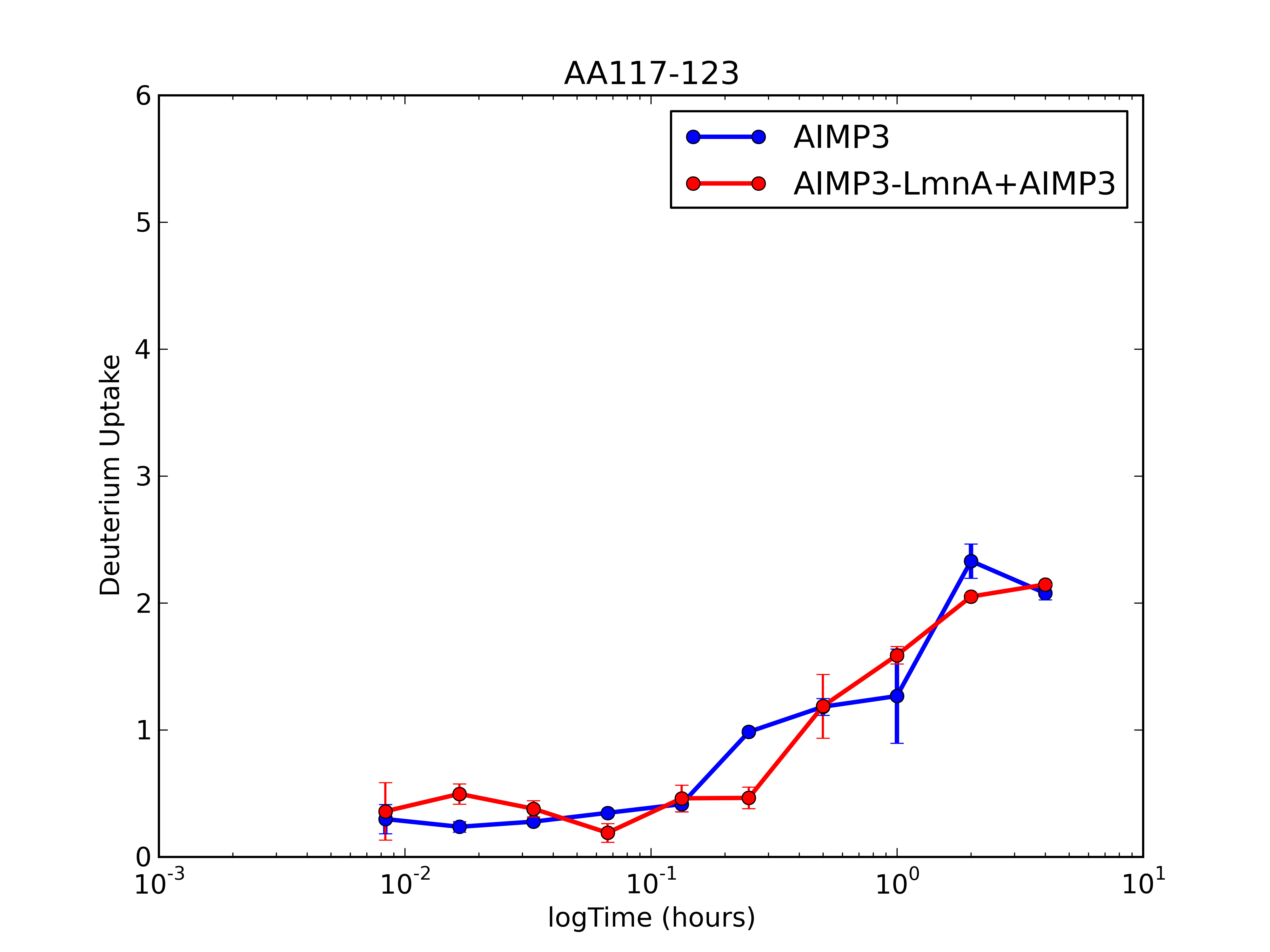

Supplement: S1 File — (ZIP) [file pone.0181869.s003.zip › logfigure-AIMP3-scale/AA117-123_charge_2_mz426.7.csv.csv.png]

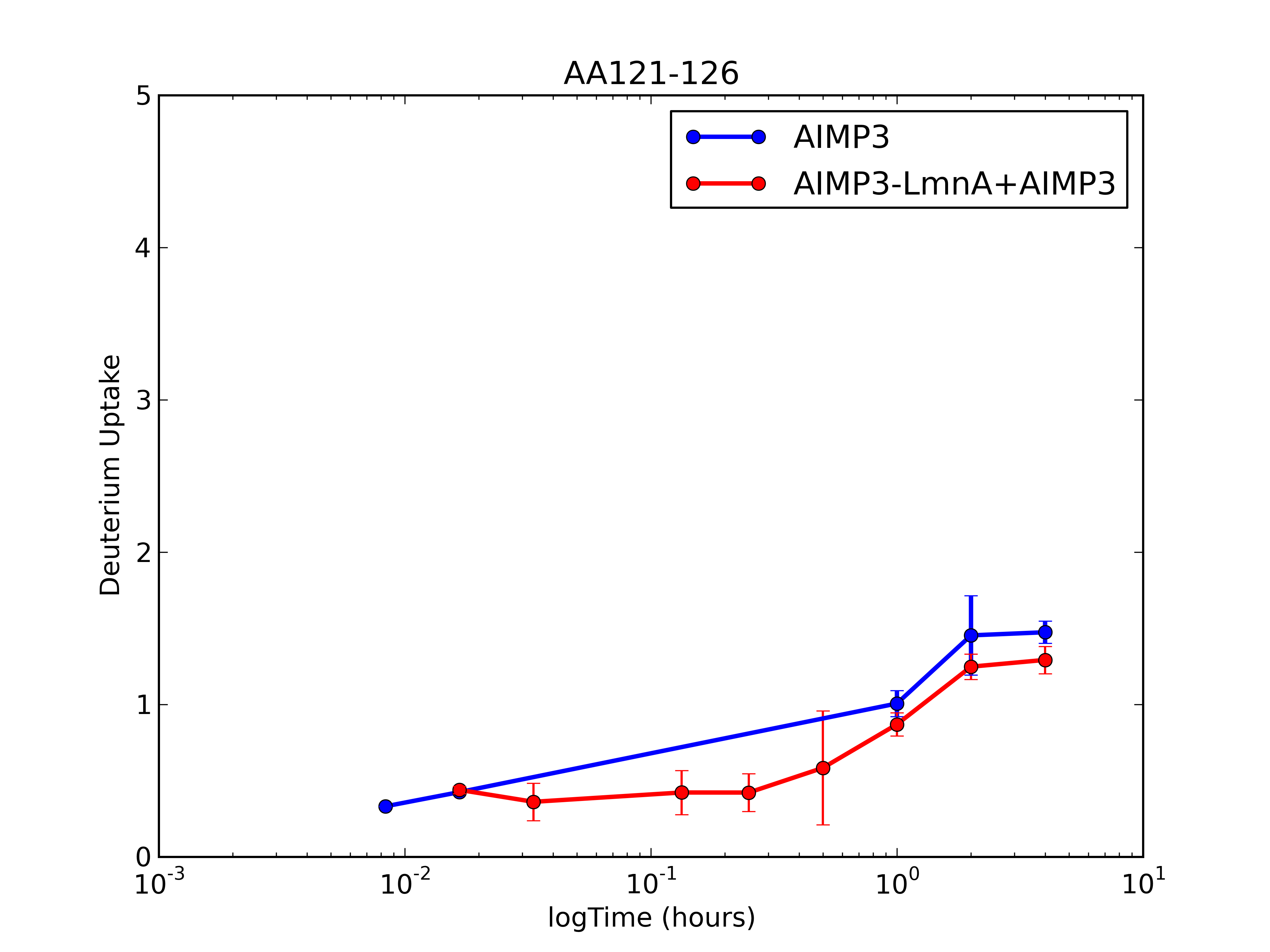

Supplement: S1 File — (ZIP) [file pone.0181869.s003.zip › logfigure-AIMP3-scale/AA121-126_charge_1_mz740.3.csv.csv.png]

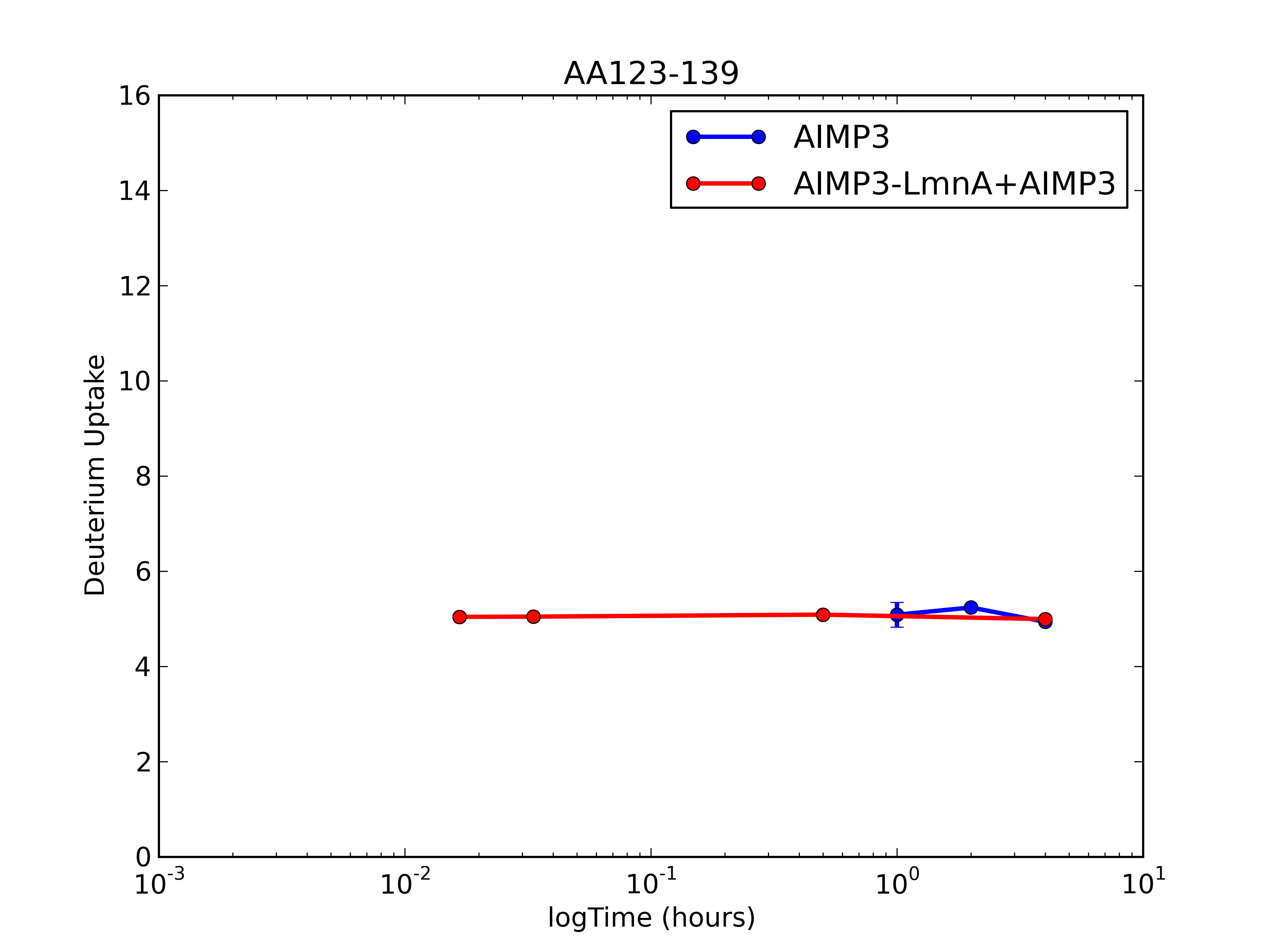

Supplement: S1 File — (ZIP) [file pone.0181869.s003.zip › logfigure-AIMP3-scale/AA123-139_charge_2_mz1012.9.csv.csv.png]

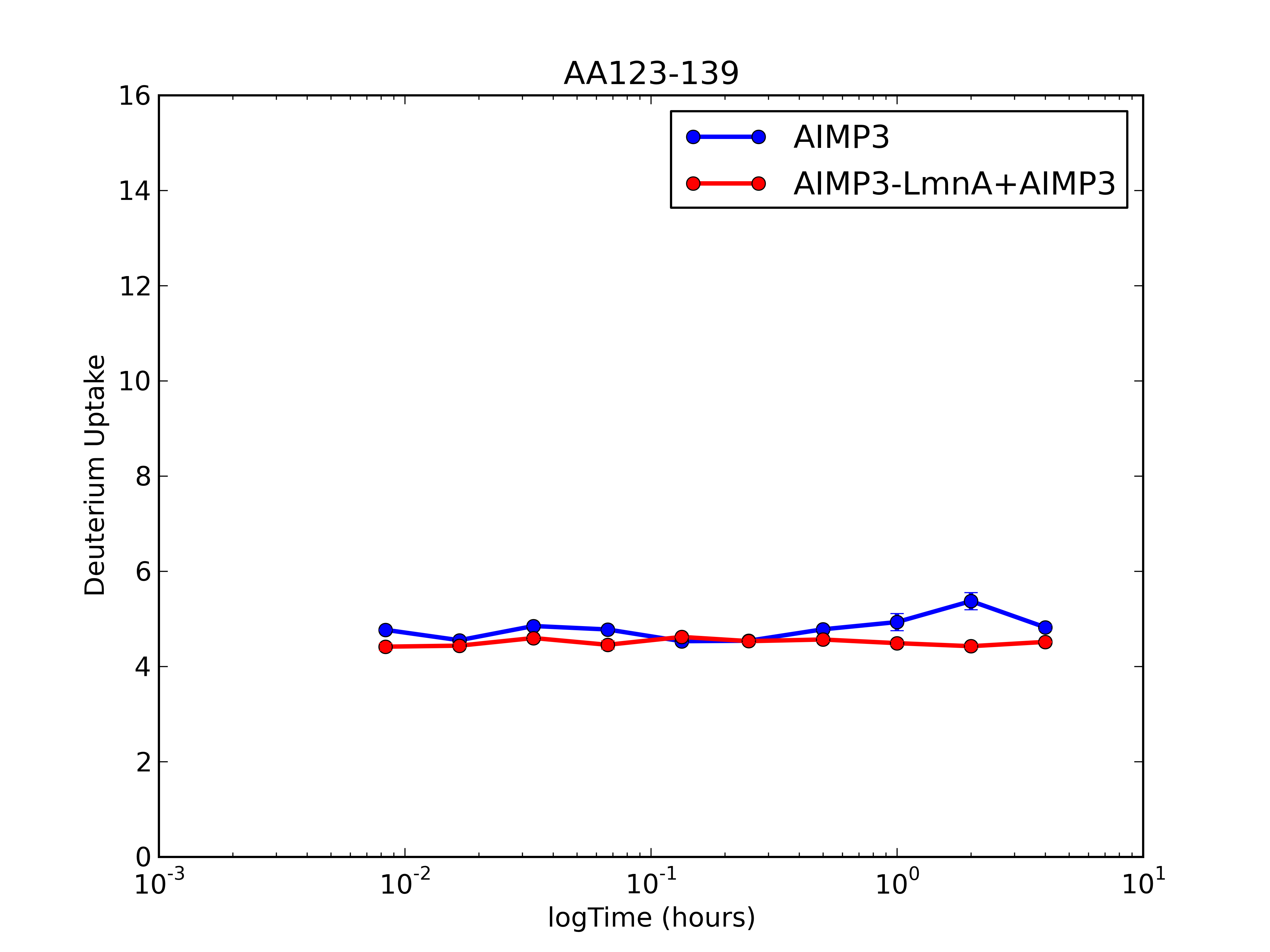

Supplement: S1 File — (ZIP) [file pone.0181869.s003.zip › logfigure-AIMP3-scale/AA123-139_charge_3_mz675.6.csv.csv.png]

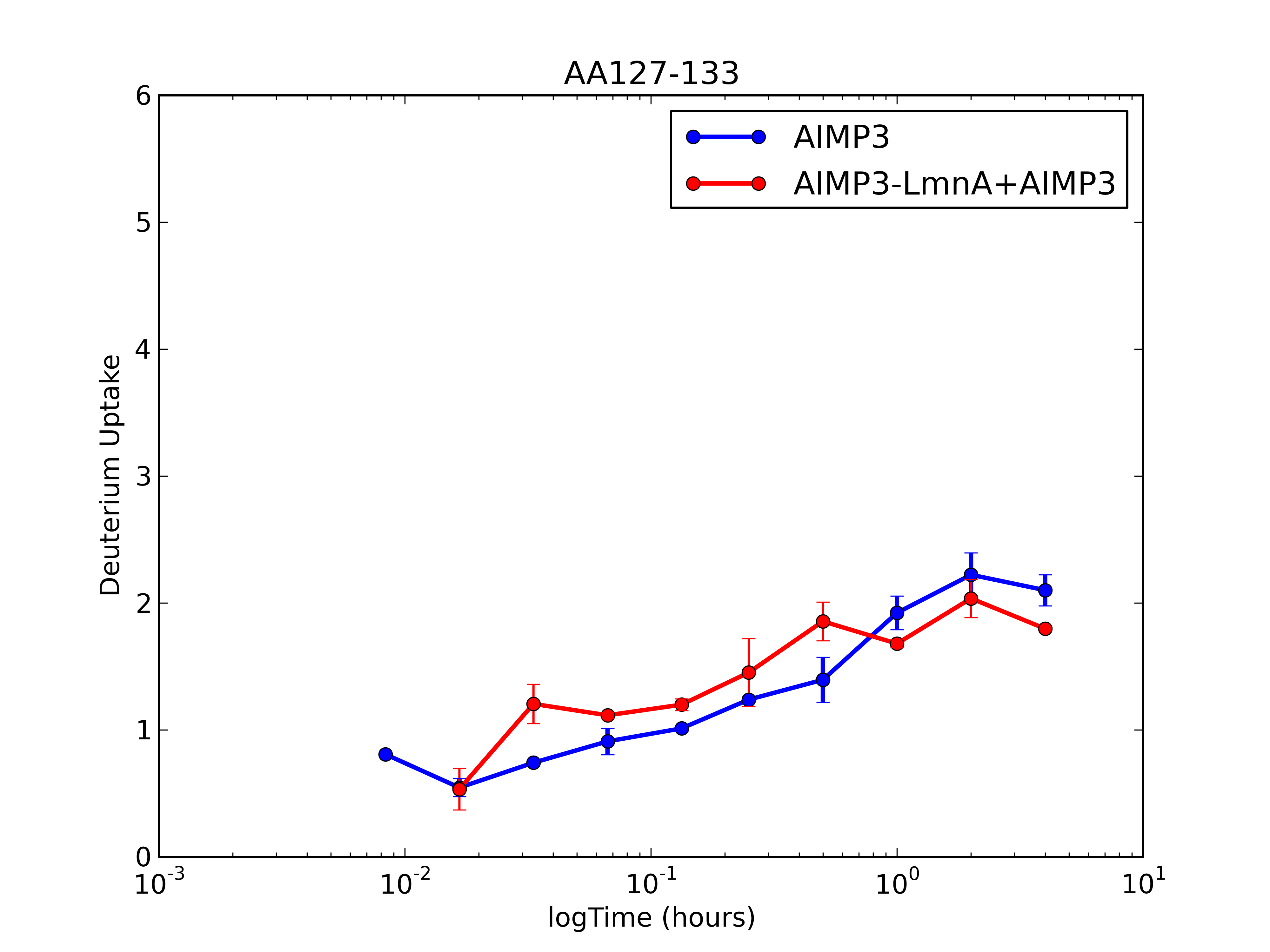

Supplement: S1 File — (ZIP) [file pone.0181869.s003.zip › logfigure-AIMP3-scale/AA127-133_charge_2_mz422.2.csv.csv.png]

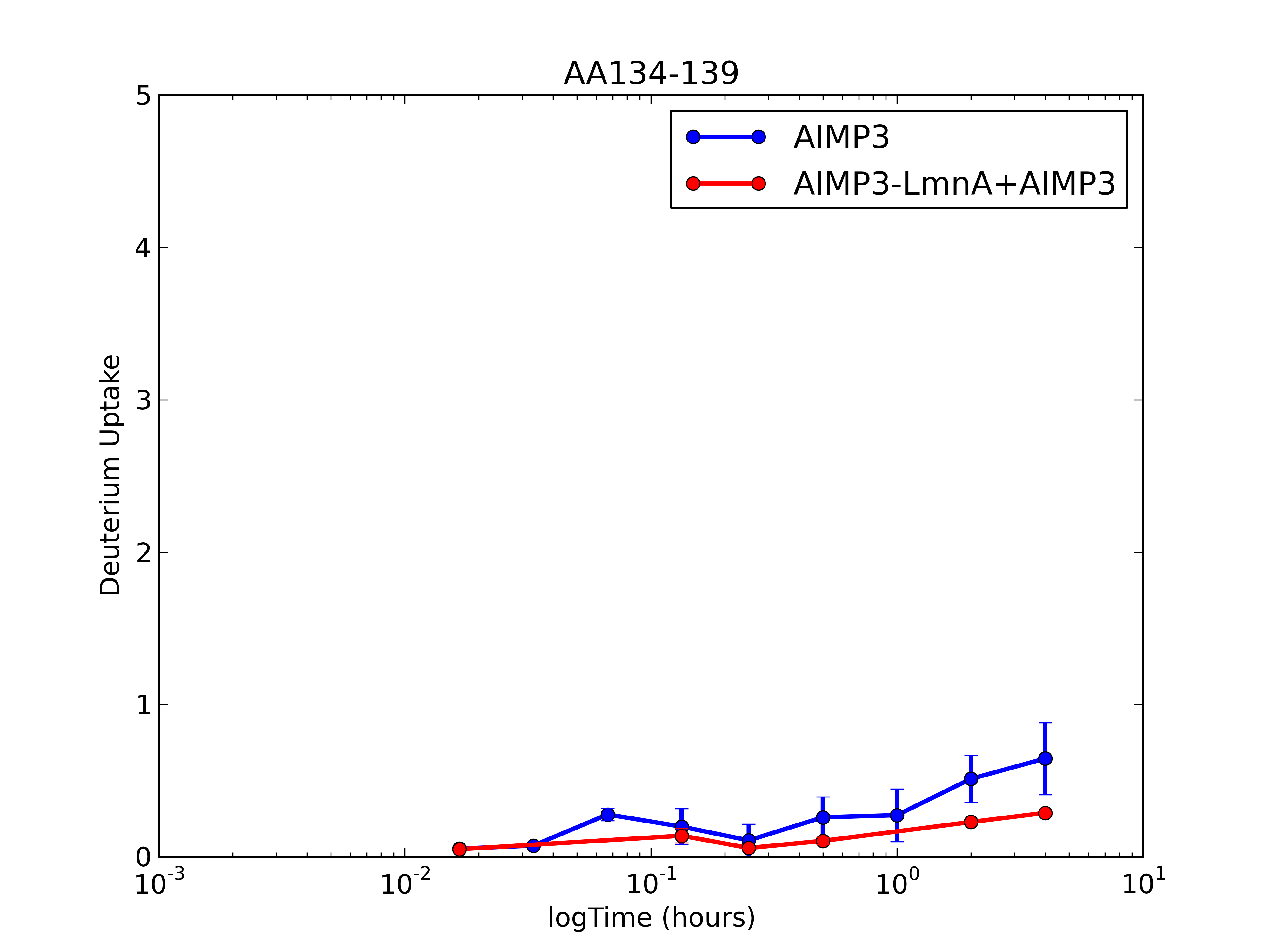

Supplement: S1 File — (ZIP) [file pone.0181869.s003.zip › logfigure-AIMP3-scale/AA134-139_charge_1_mz680.3.csv.csv.png]

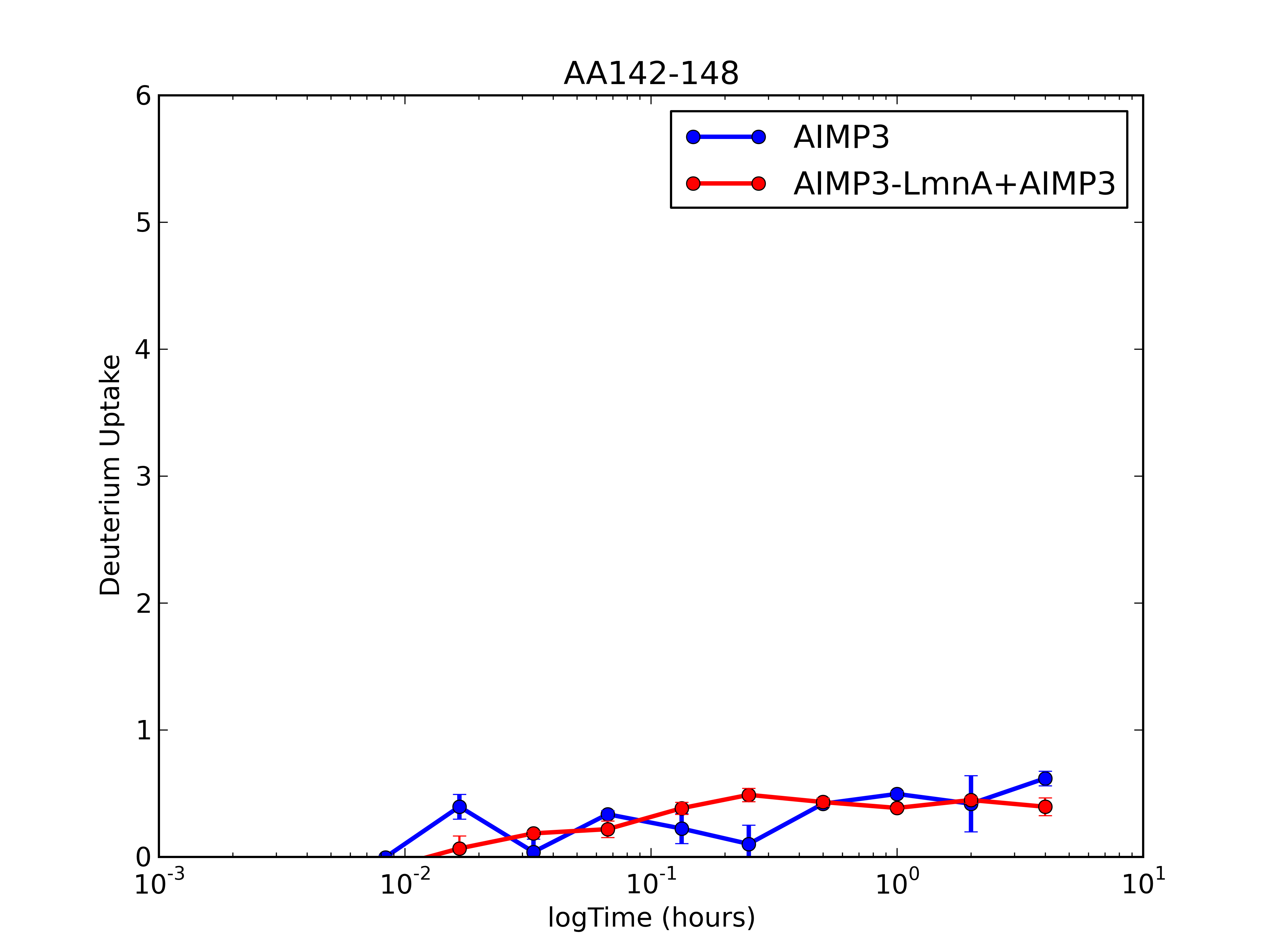

Supplement: S1 File — (ZIP) [file pone.0181869.s003.zip › logfigure-AIMP3-scale/AA142-148_charge_2_mz461.2.csv.csv.png]

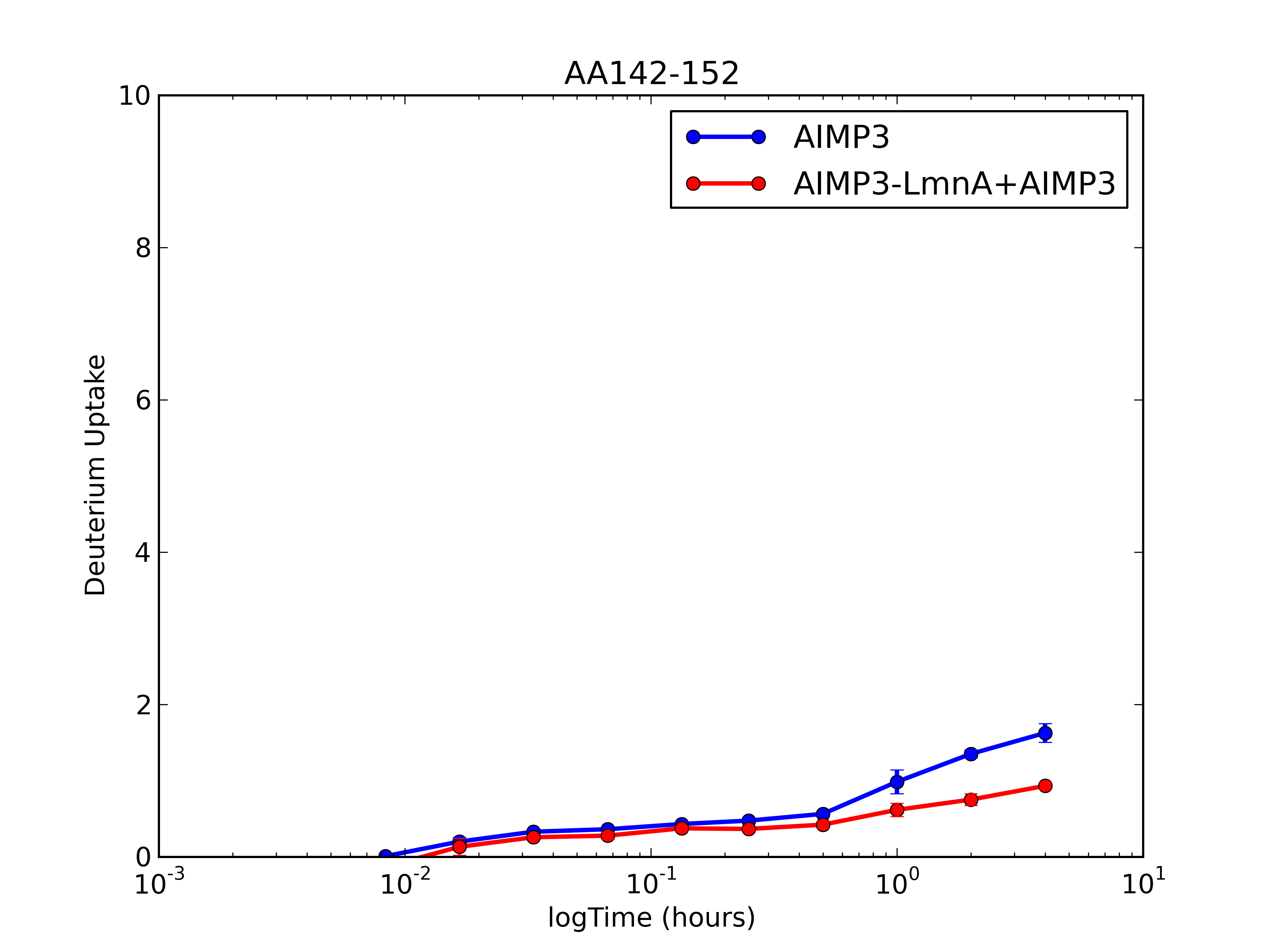

Supplement: S1 File — (ZIP) [file pone.0181869.s003.zip › logfigure-AIMP3-scale/AA142-152_charge_2_mz698.3.csv.csv.png]

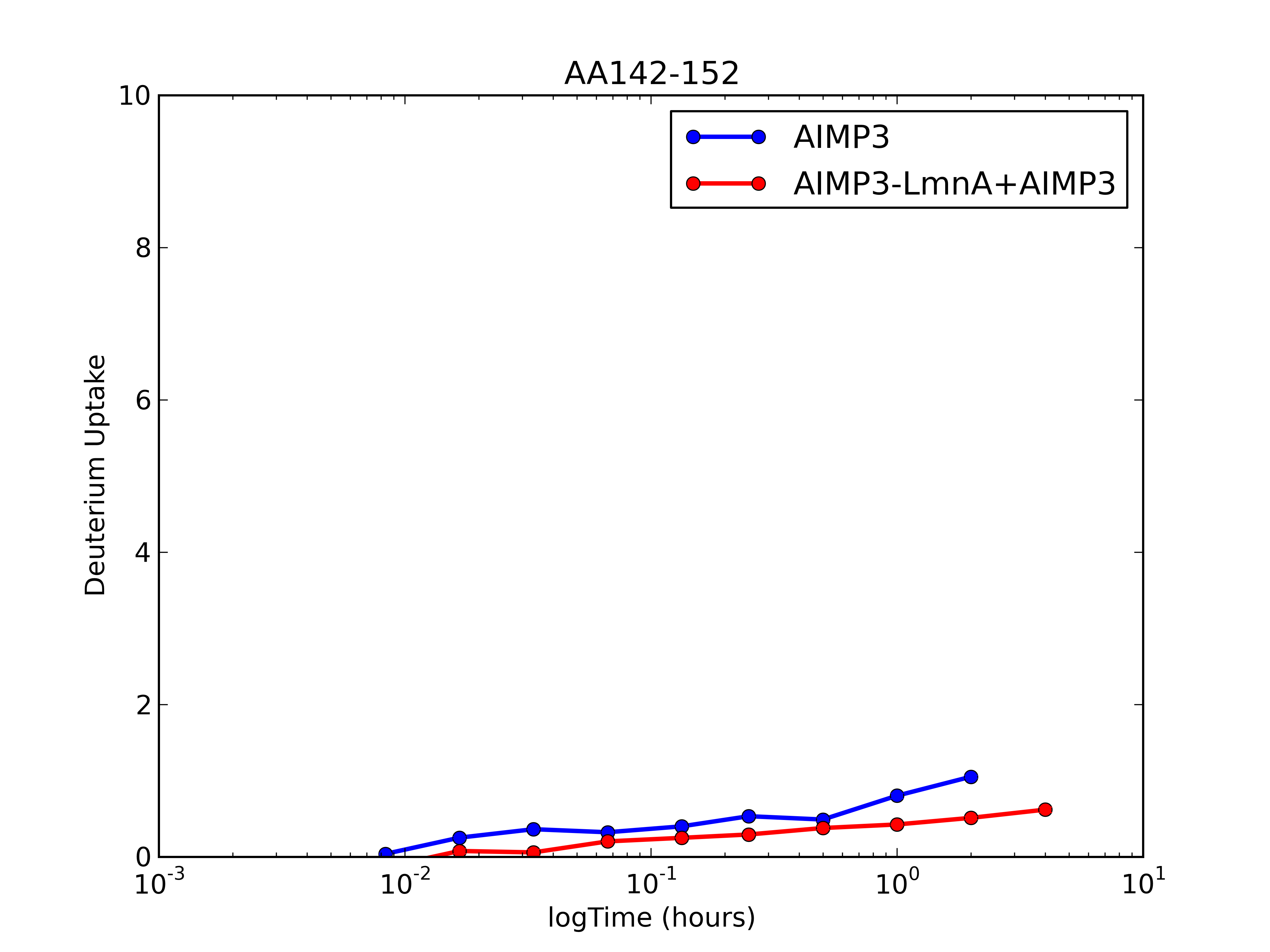

Supplement: S1 File — (ZIP) [file pone.0181869.s003.zip › logfigure-AIMP3-scale/AA142-152_charge_3_mz465.9.csv.csv.png]

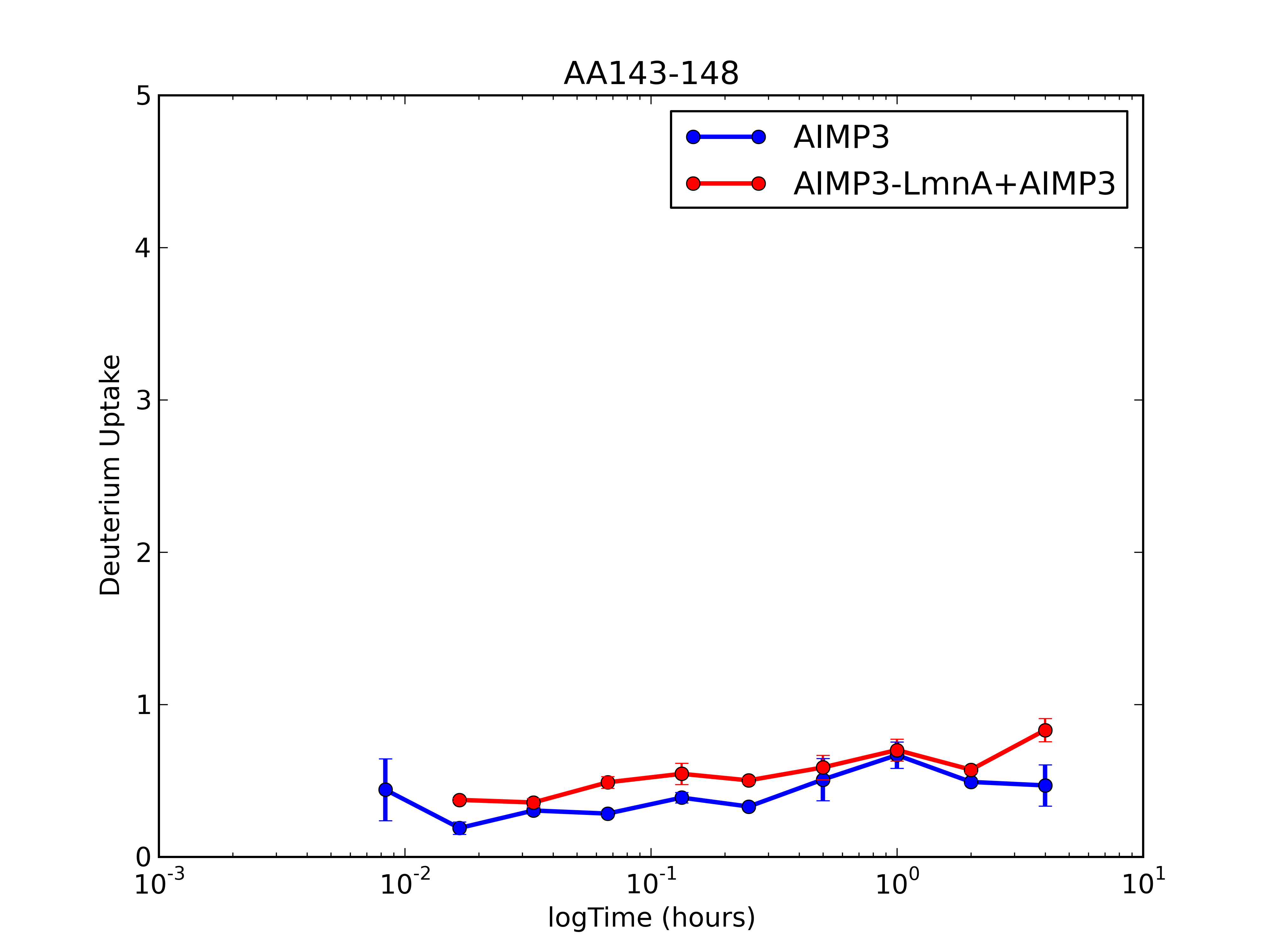

Supplement: S1 File — (ZIP) [file pone.0181869.s003.zip › logfigure-AIMP3-scale/AA143-148_charge_2_mz404.7.csv.csv.png]

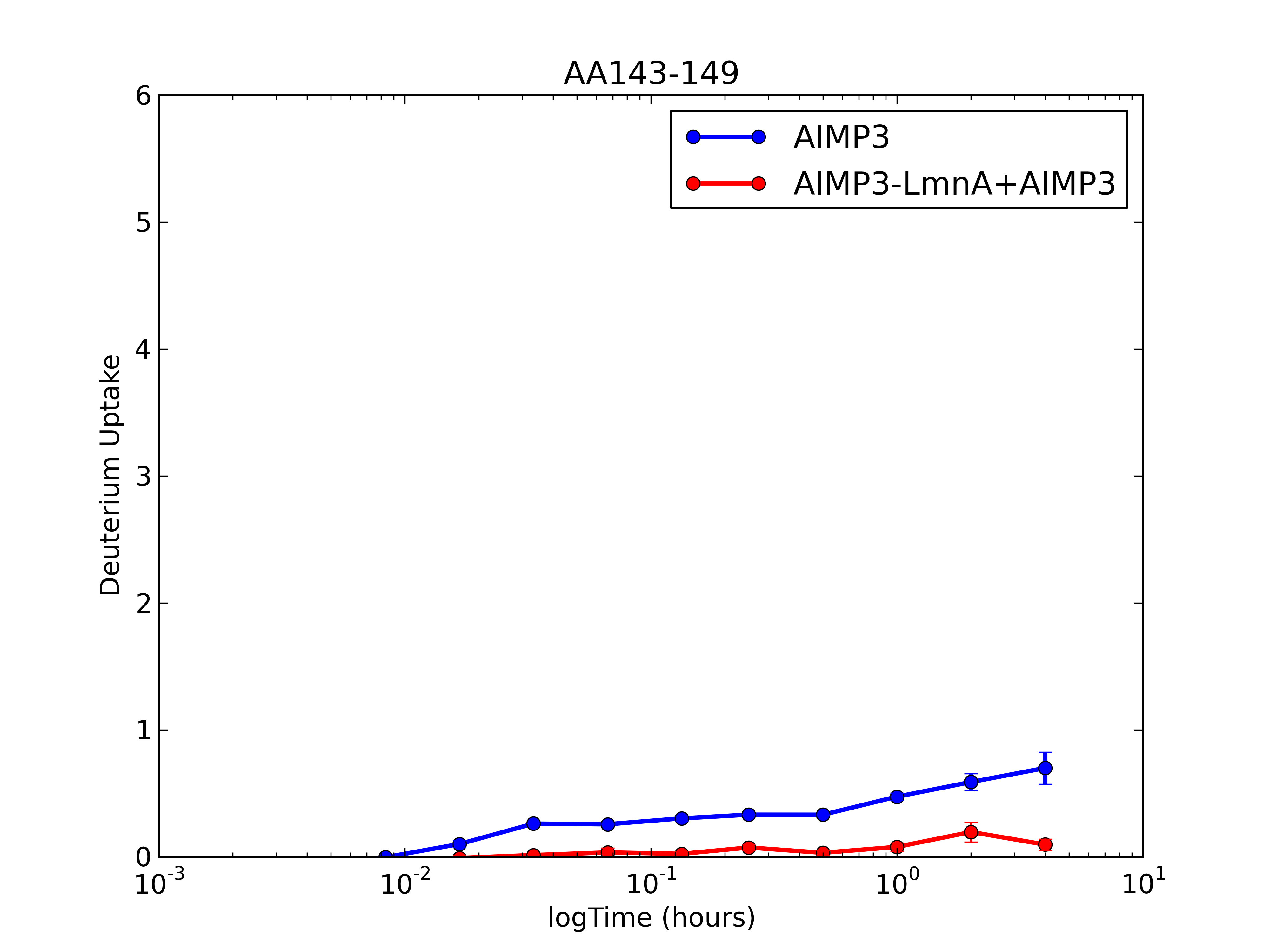

Supplement: S1 File — (ZIP) [file pone.0181869.s003.zip › logfigure-AIMP3-scale/AA143-149_charge_2_mz478.2.csv.csv.png]

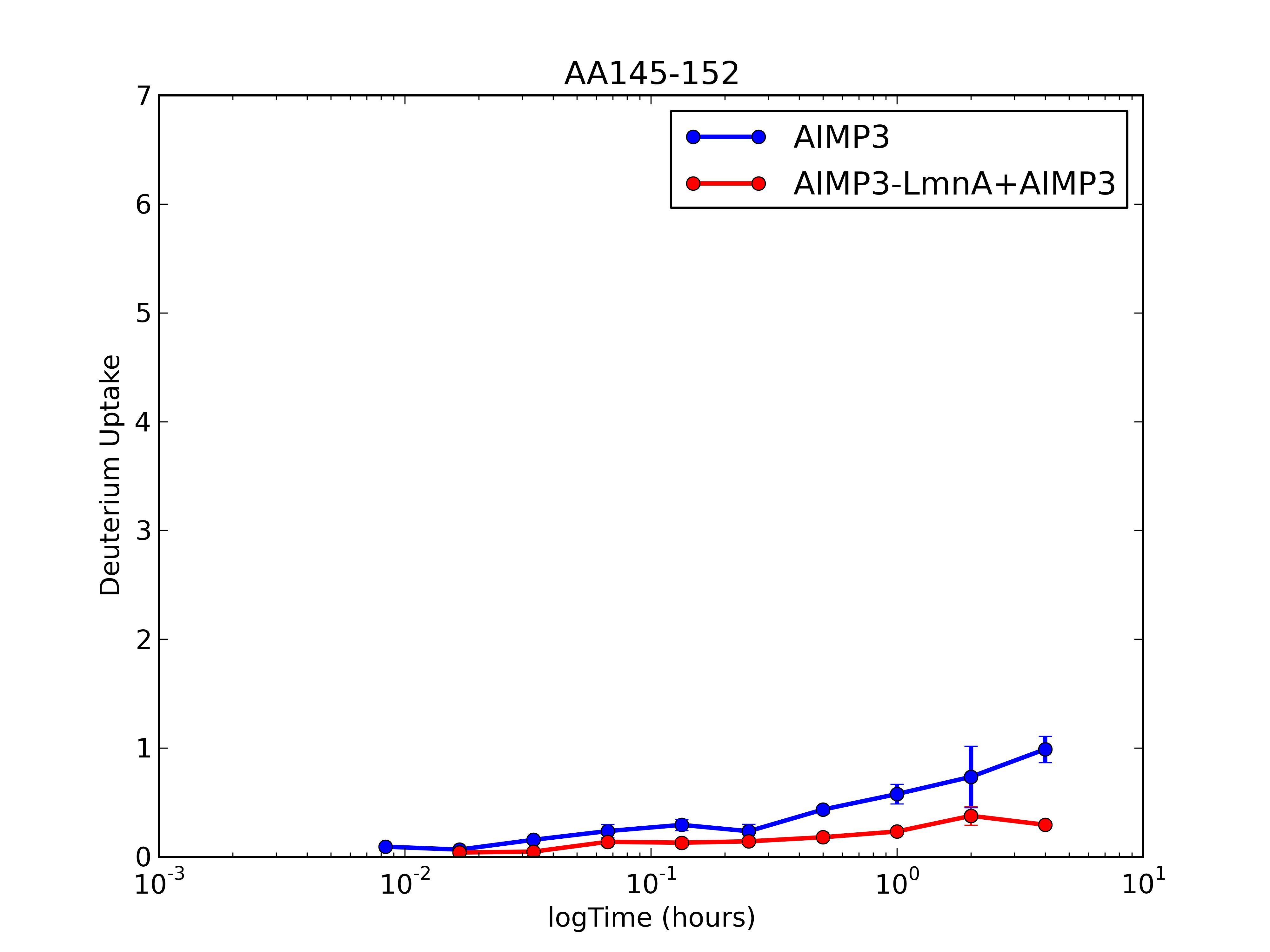

Supplement: S1 File — (ZIP) [file pone.0181869.s003.zip › logfigure-AIMP3-scale/AA145-152_charge_2_mz478.7.csv.csv.png]

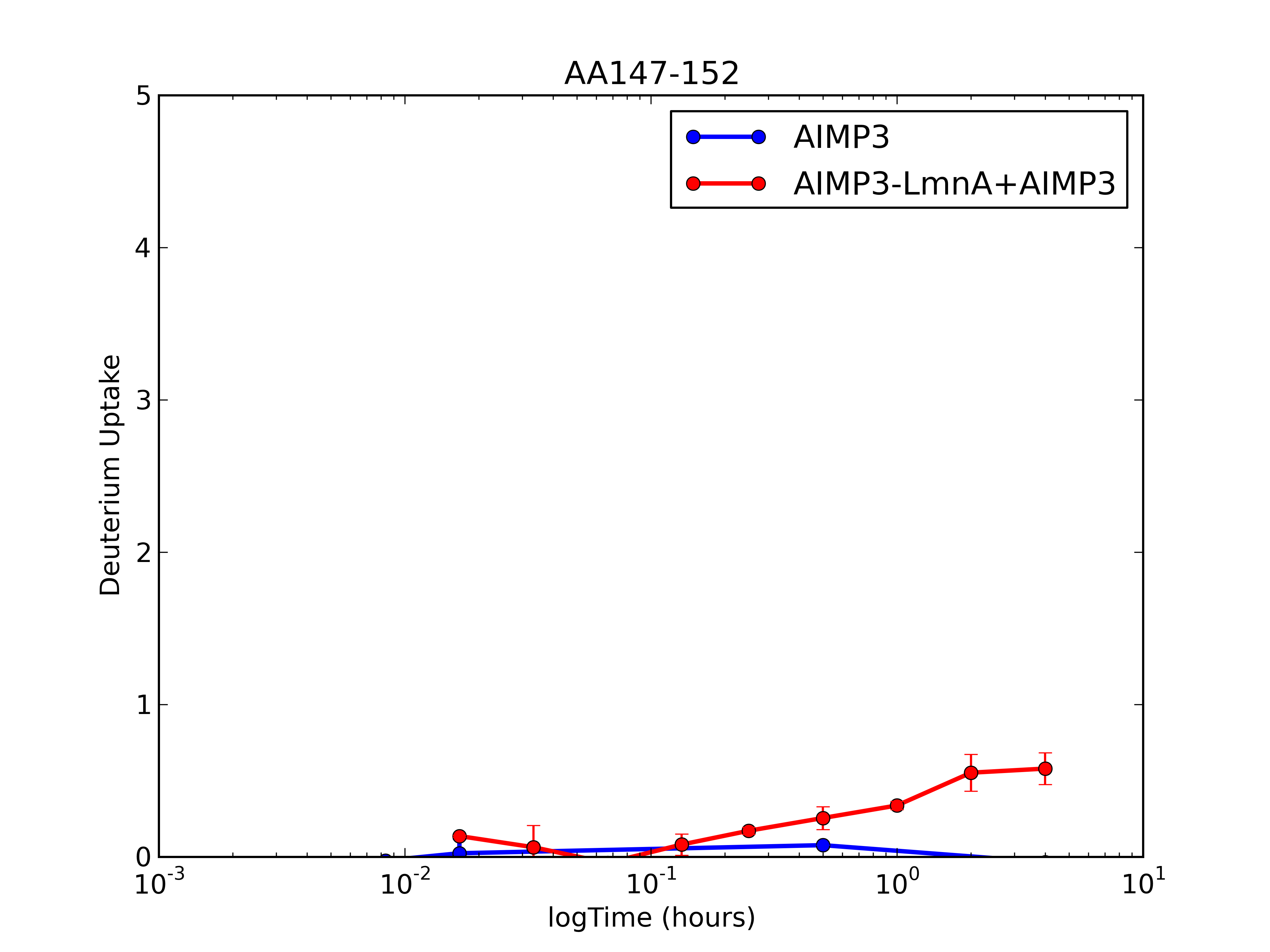

Supplement: S1 File — (ZIP) [file pone.0181869.s003.zip › logfigure-AIMP3-scale/AA147-152_charge_1_mz786.4.csv.csv.png]

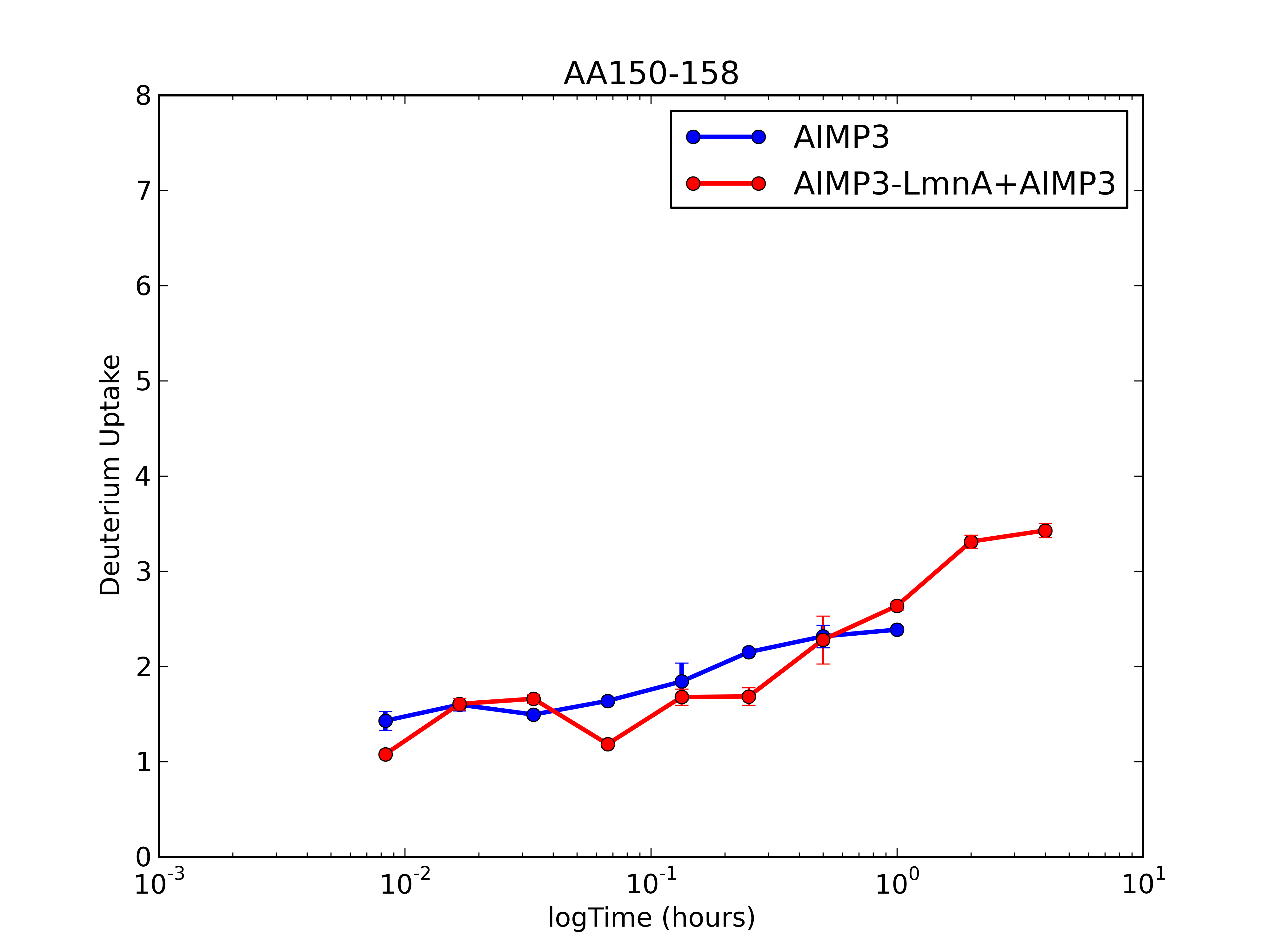

Supplement: S1 File — (ZIP) [file pone.0181869.s003.zip › logfigure-AIMP3-scale/AA150-158_charge_2_mz522.8.csv.csv.png]

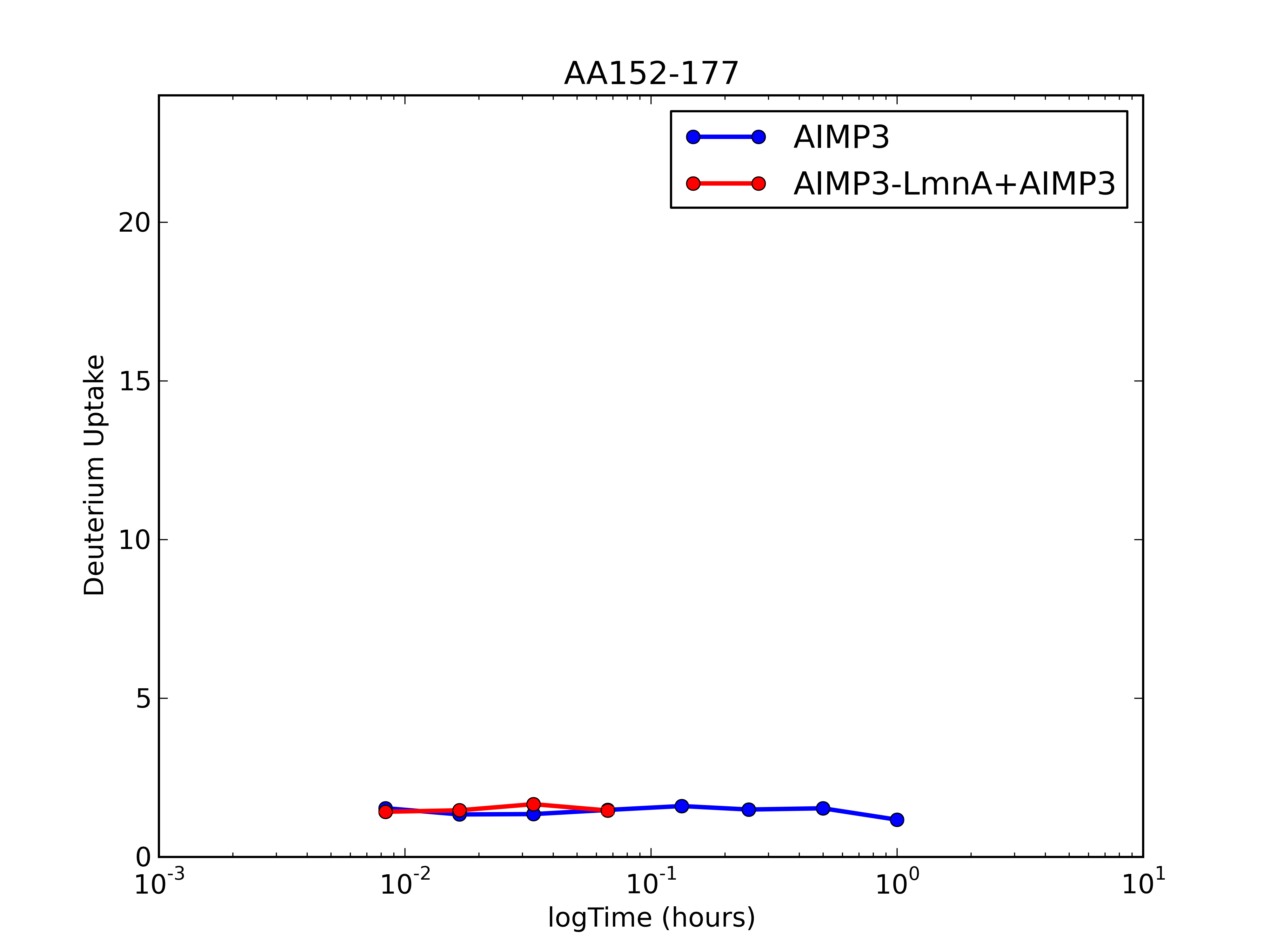

Supplement: S1 File — (ZIP) [file pone.0181869.s003.zip › logfigure-AIMP3-scale/AA152-177_charge_4_mz801.6.csv.csv.png]

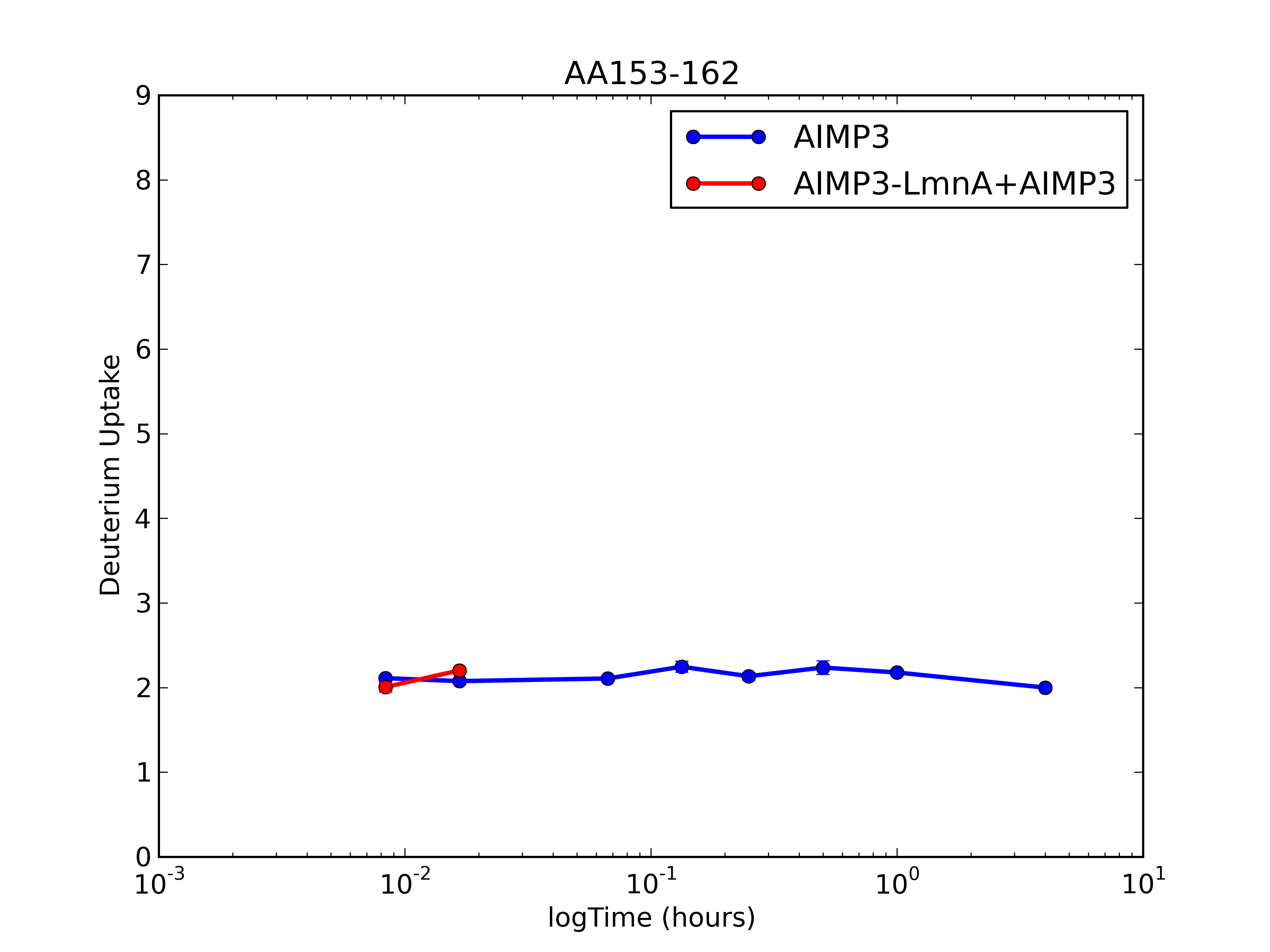

Supplement: S1 File — (ZIP) [file pone.0181869.s003.zip › logfigure-AIMP3-scale/AA153-162_charge_3_mz417.5.csv.csv.png]

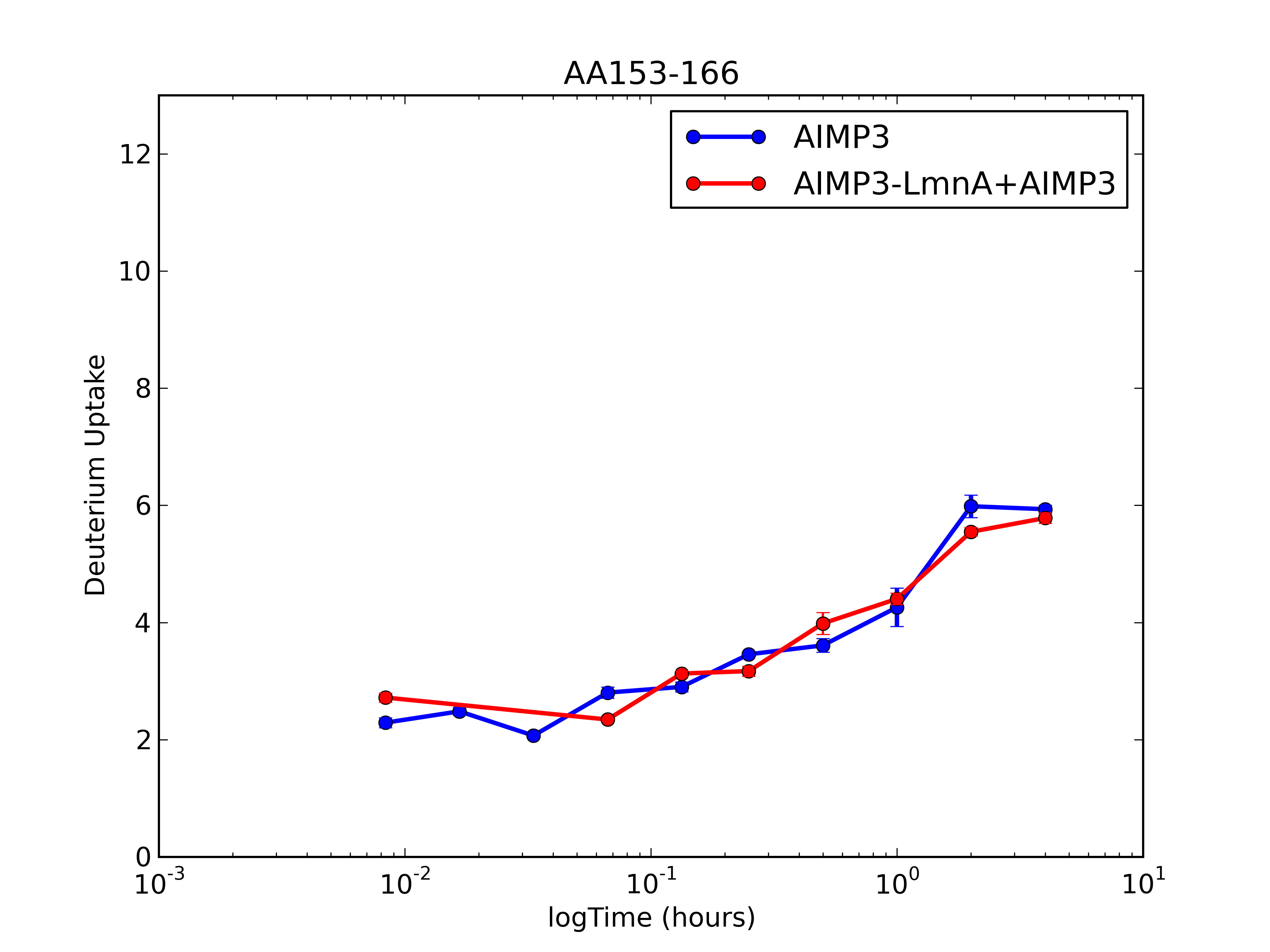

Supplement: S1 File — (ZIP) [file pone.0181869.s003.zip › logfigure-AIMP3-scale/AA153-166_charge_3_mz569.6.csv.csv.png]

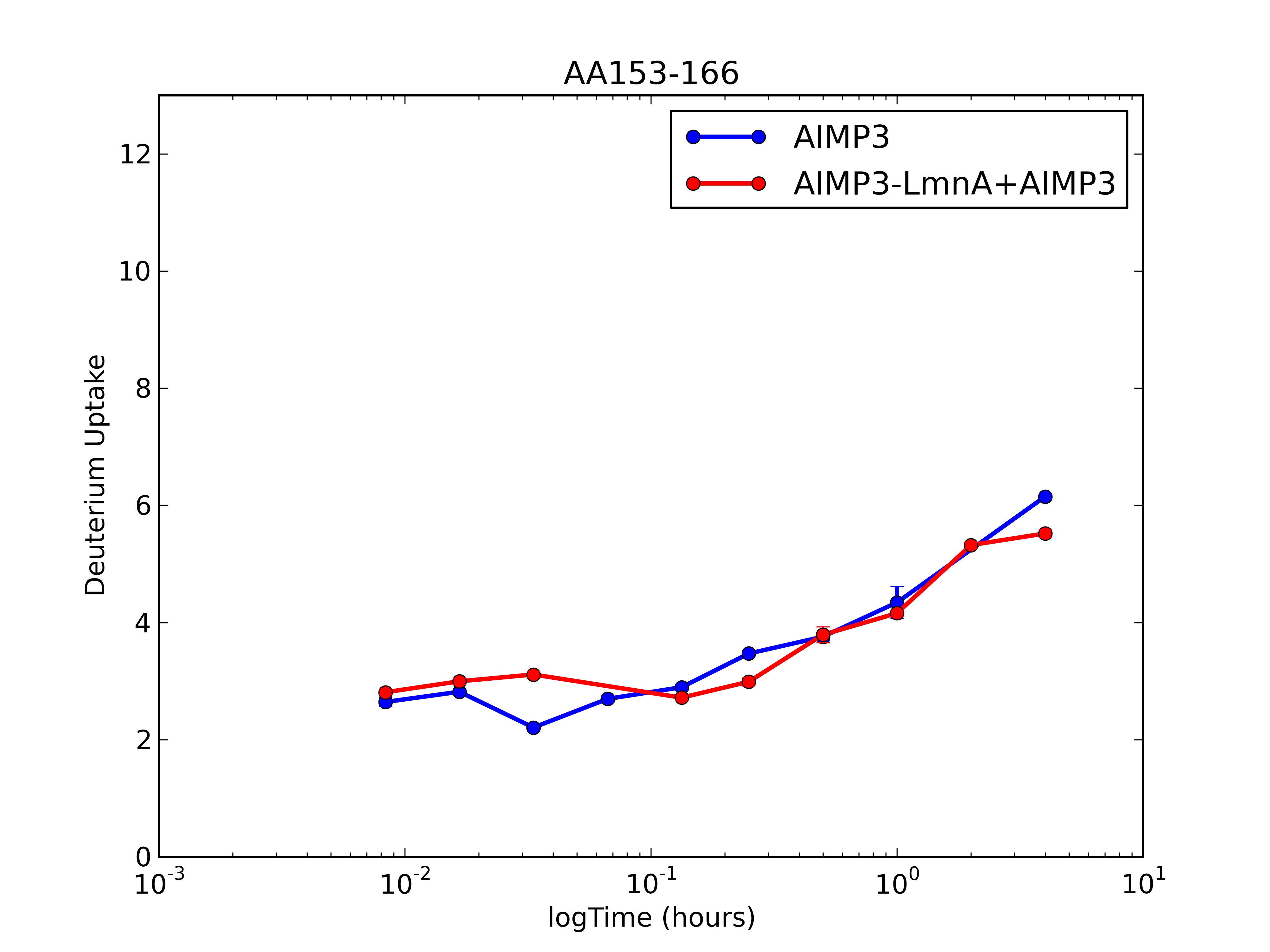

Supplement: S1 File — (ZIP) [file pone.0181869.s003.zip › logfigure-AIMP3-scale/AA153-166_charge_4_mz427.4.csv.csv.png]

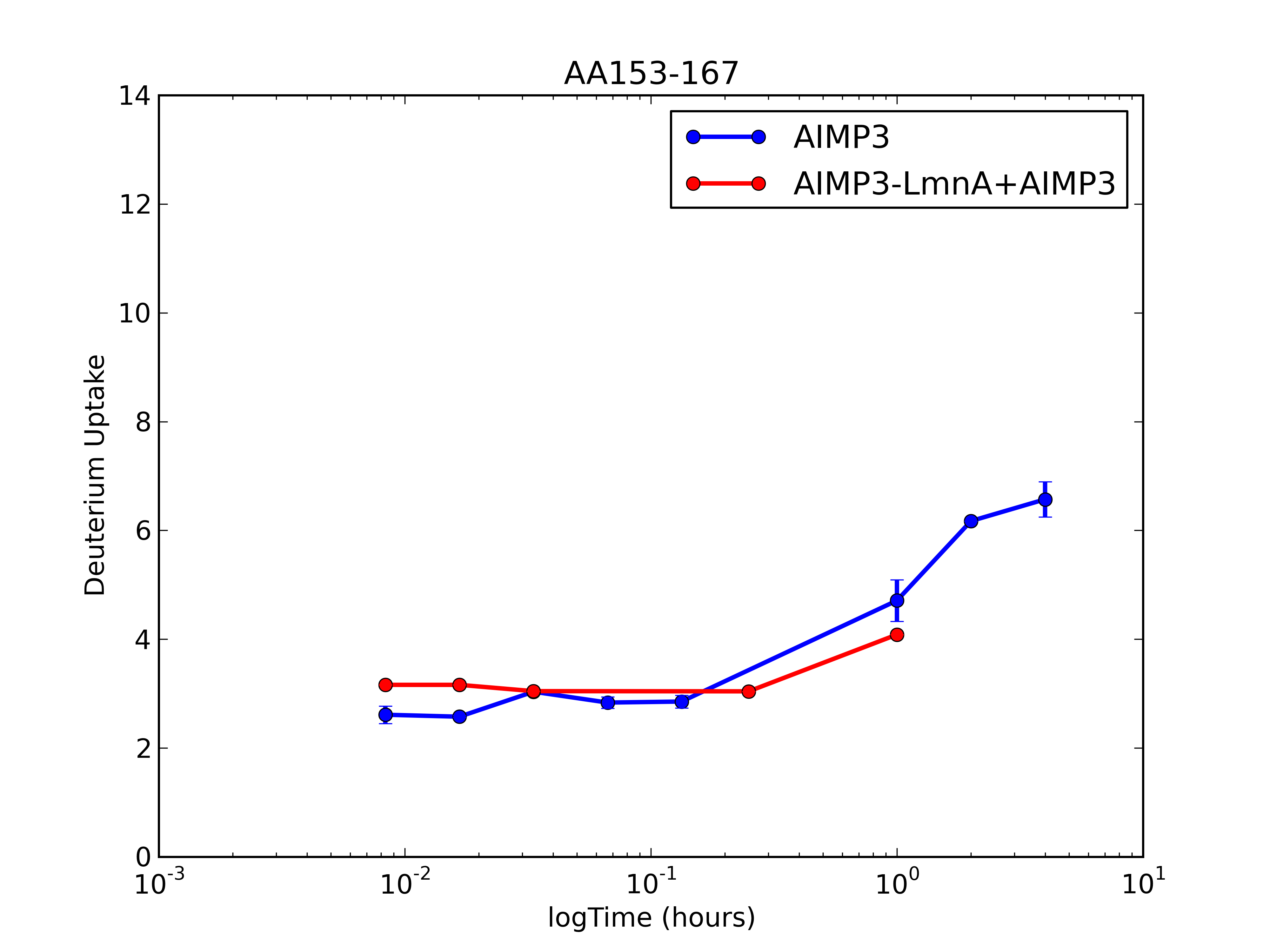

Supplement: S1 File — (ZIP) [file pone.0181869.s003.zip › logfigure-AIMP3-scale/AA153-167_charge_3_mz631.6.csv.csv.png]

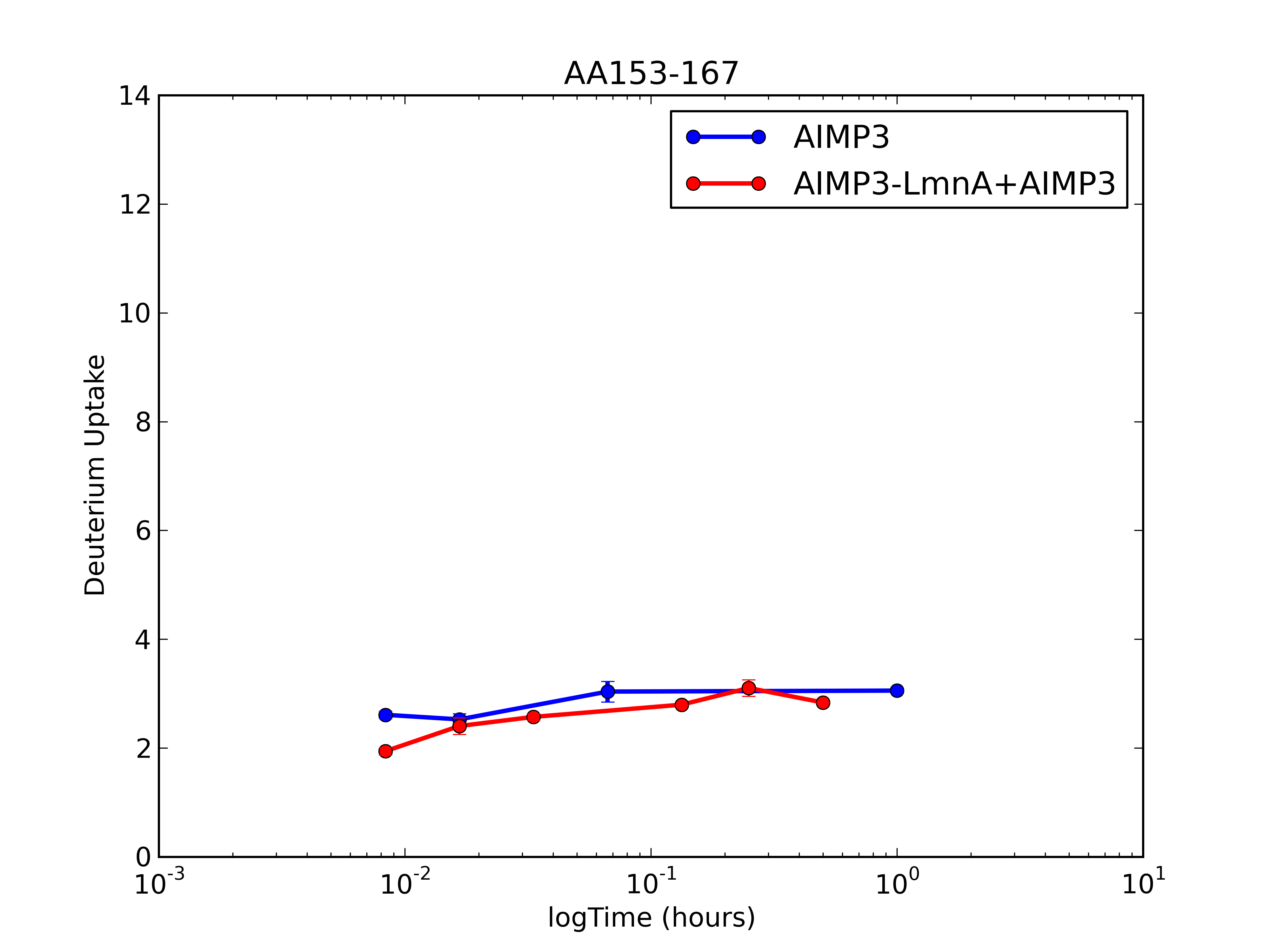

Supplement: S1 File — (ZIP) [file pone.0181869.s003.zip › logfigure-AIMP3-scale/AA153-167_charge_4_mz474.0.csv.csv.png]

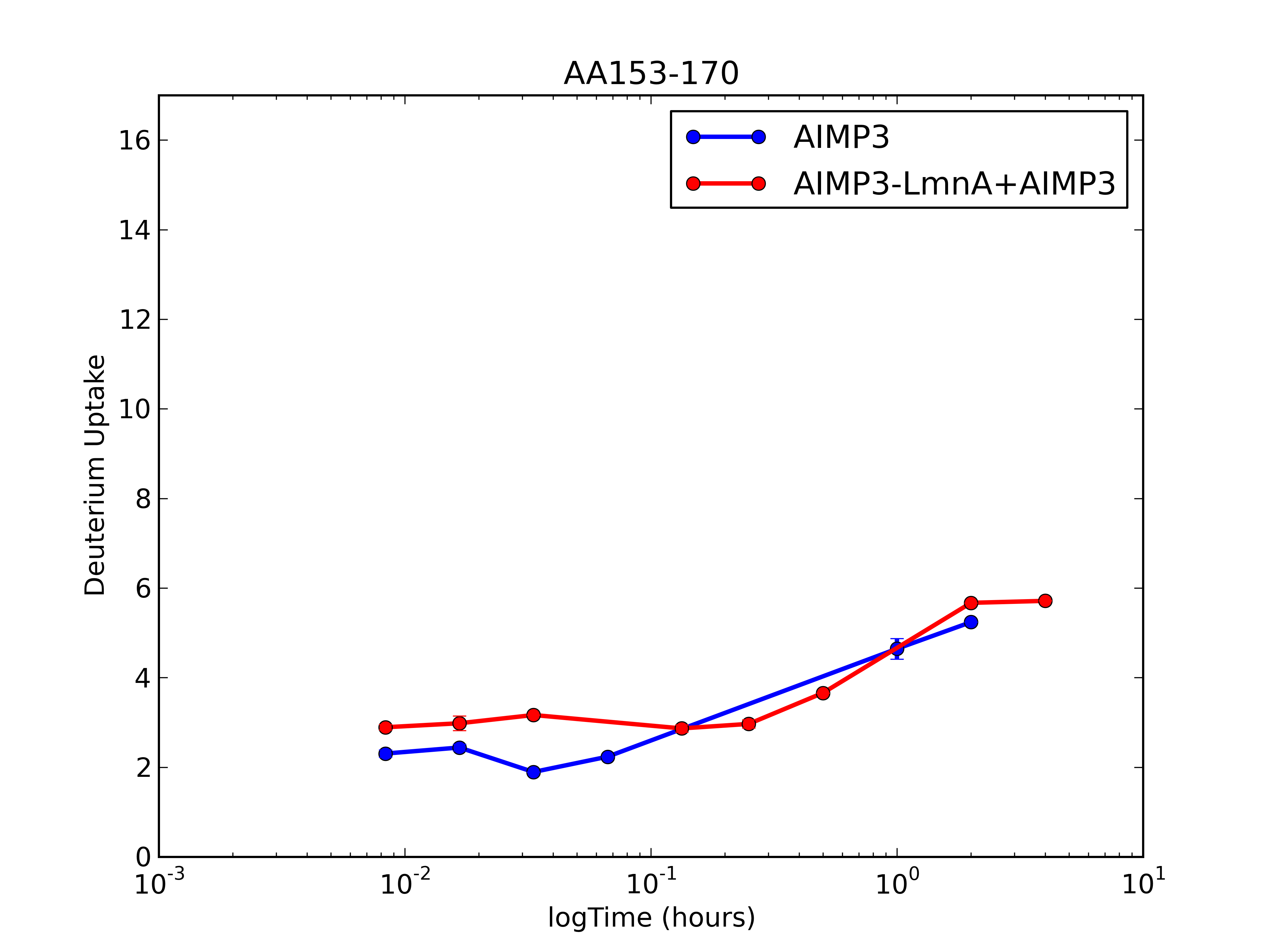

Supplement: S1 File — (ZIP) [file pone.0181869.s003.zip › logfigure-AIMP3-scale/AA153-170_charge_4_mz570.7.csv.csv.png]

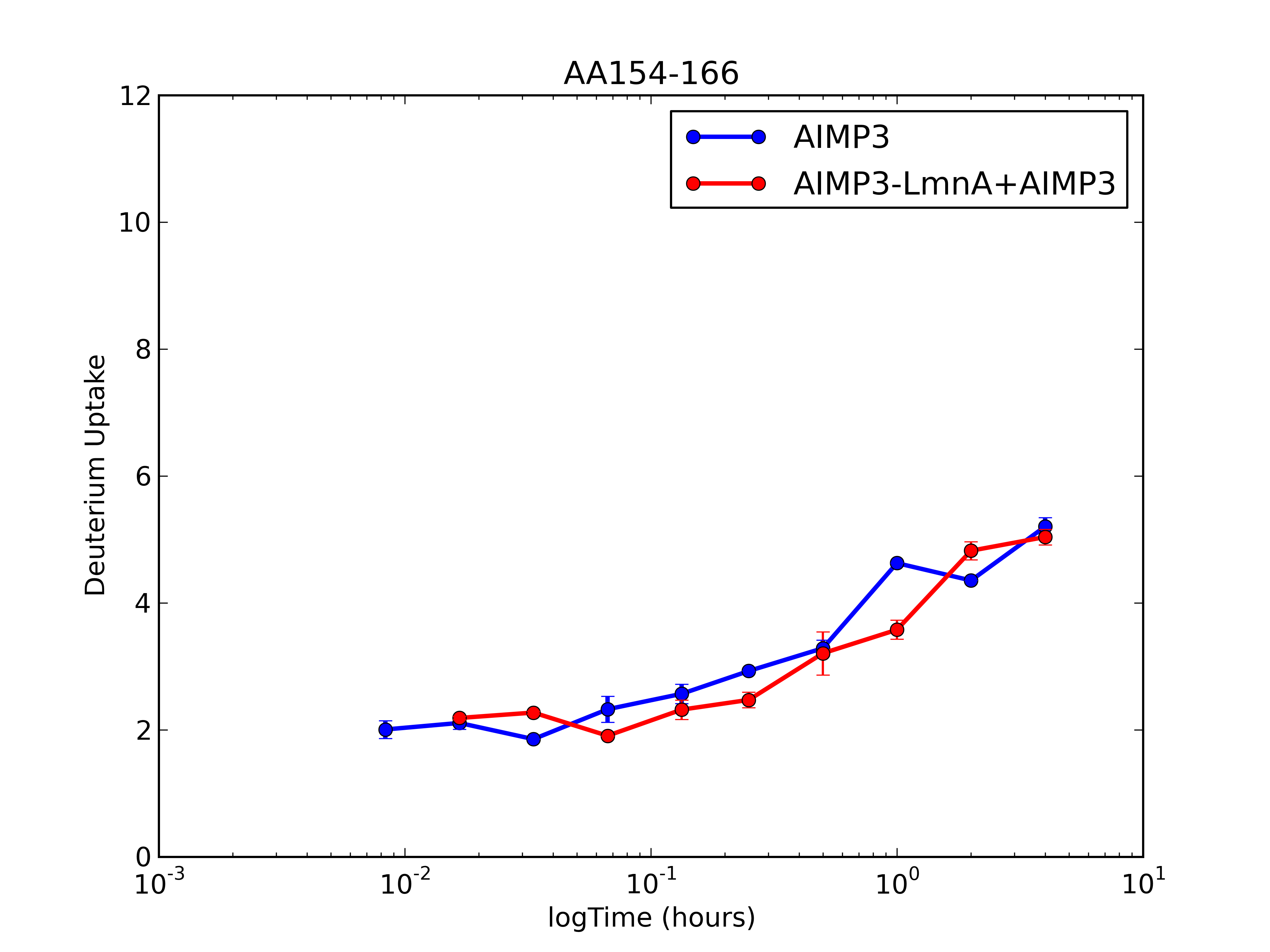

Supplement: S1 File — (ZIP) [file pone.0181869.s003.zip › logfigure-AIMP3-scale/AA154-166_charge_2_mz797.4.csv.csv.png]

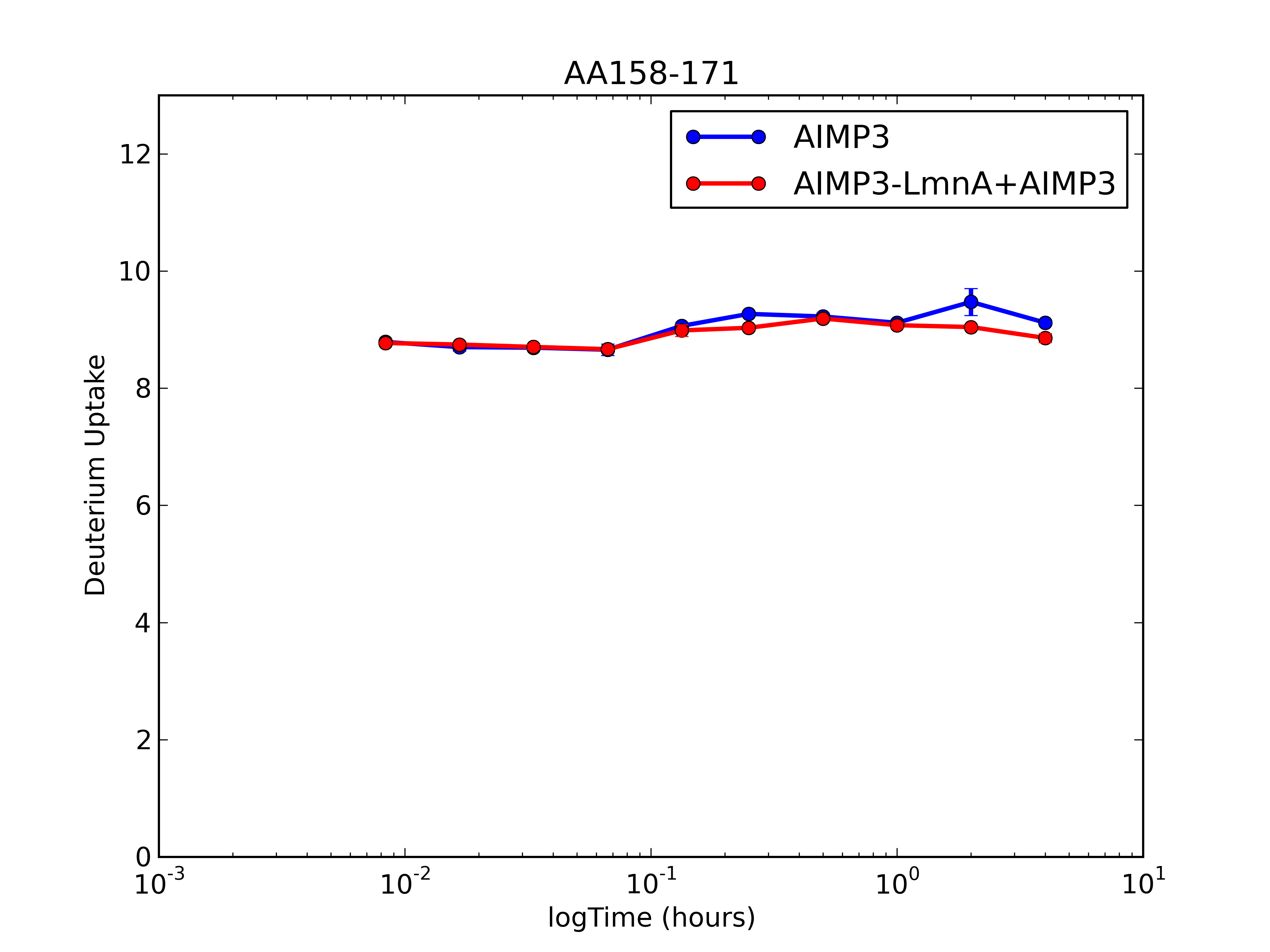

Supplement: S1 File — (ZIP) [file pone.0181869.s003.zip › logfigure-AIMP3-scale/AA158-171_charge_3_mz608.3.csv.csv.png]

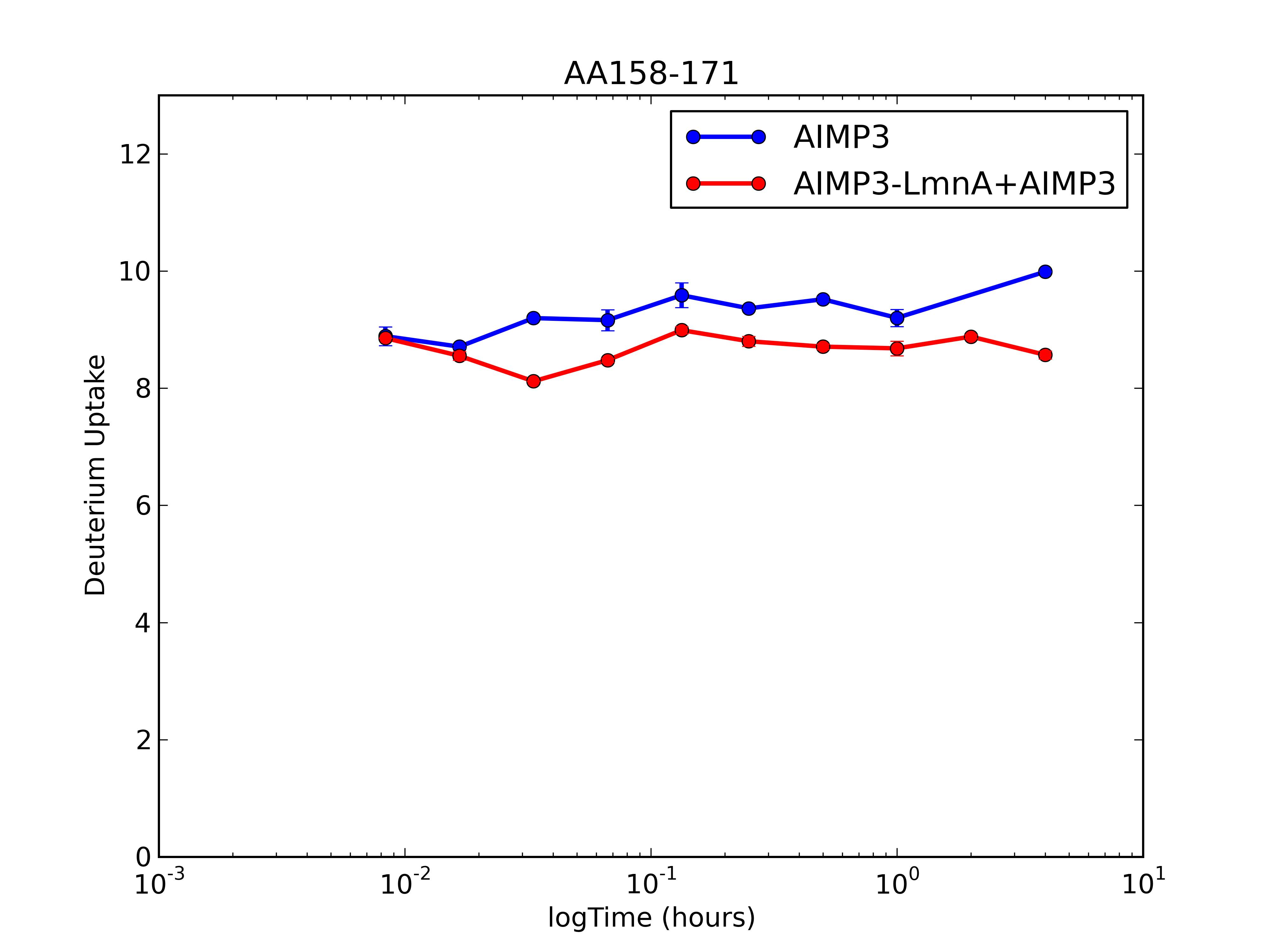

Supplement: S1 File — (ZIP) [file pone.0181869.s003.zip › logfigure-AIMP3-scale/AA158-171_charge_4_mz456.4.csv.csv.png]

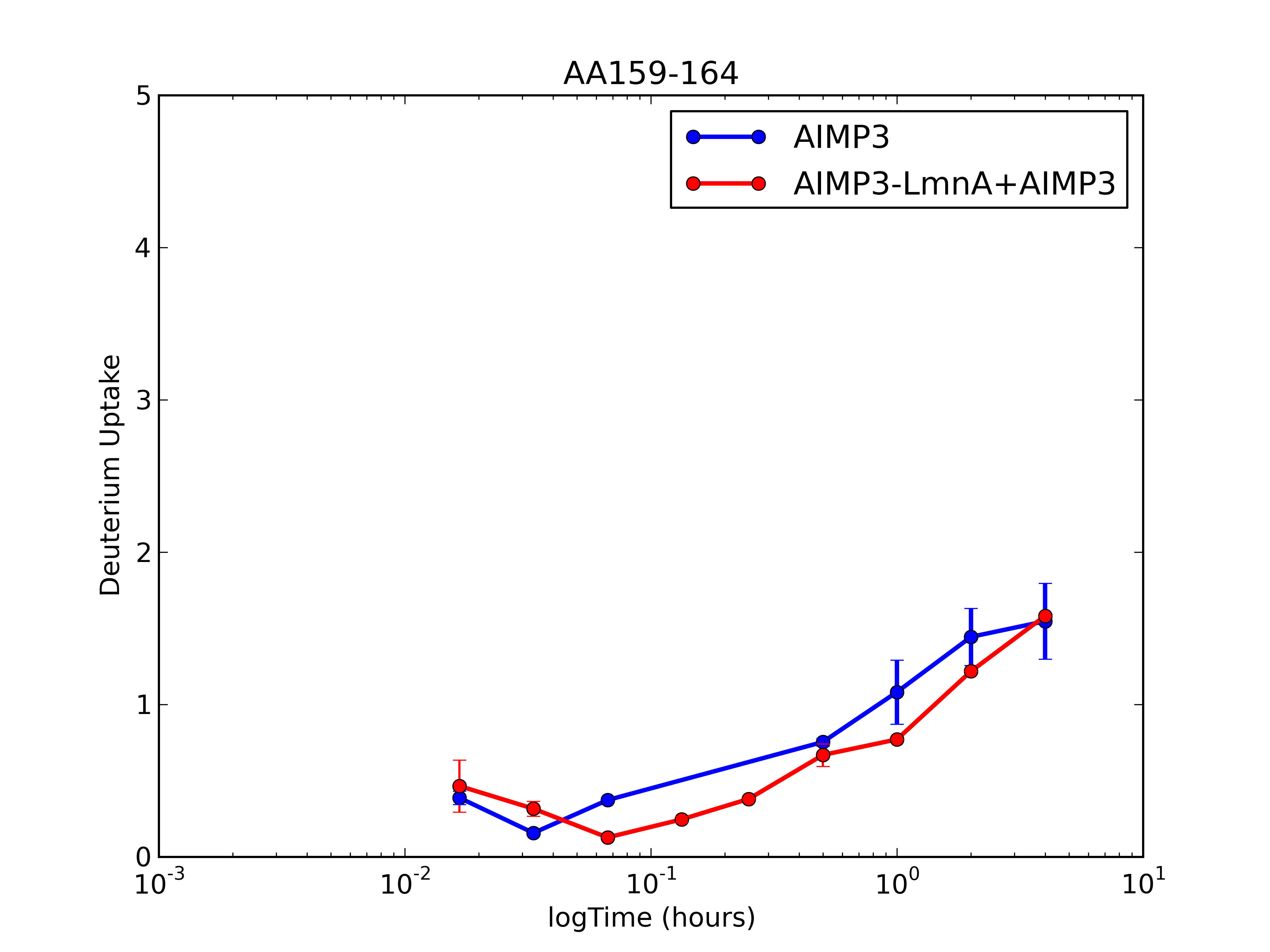

Supplement: S1 File — (ZIP) [file pone.0181869.s003.zip › logfigure-AIMP3-scale/AA159-164_charge_1_mz765.4.csv.csv.png]

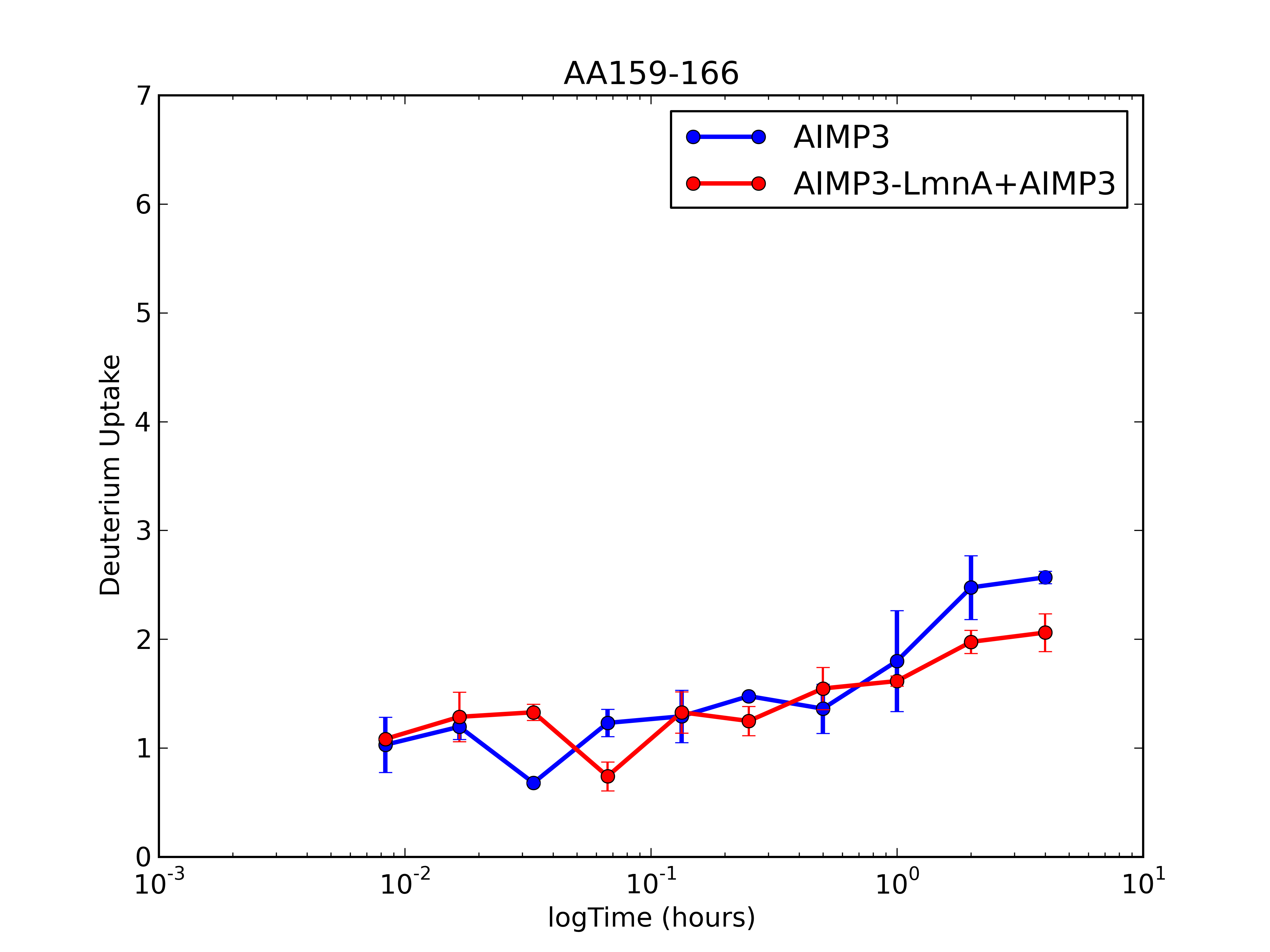

Supplement: S1 File — (ZIP) [file pone.0181869.s003.zip › logfigure-AIMP3-scale/AA159-166_charge_1_mz1008.5.csv.csv.png]

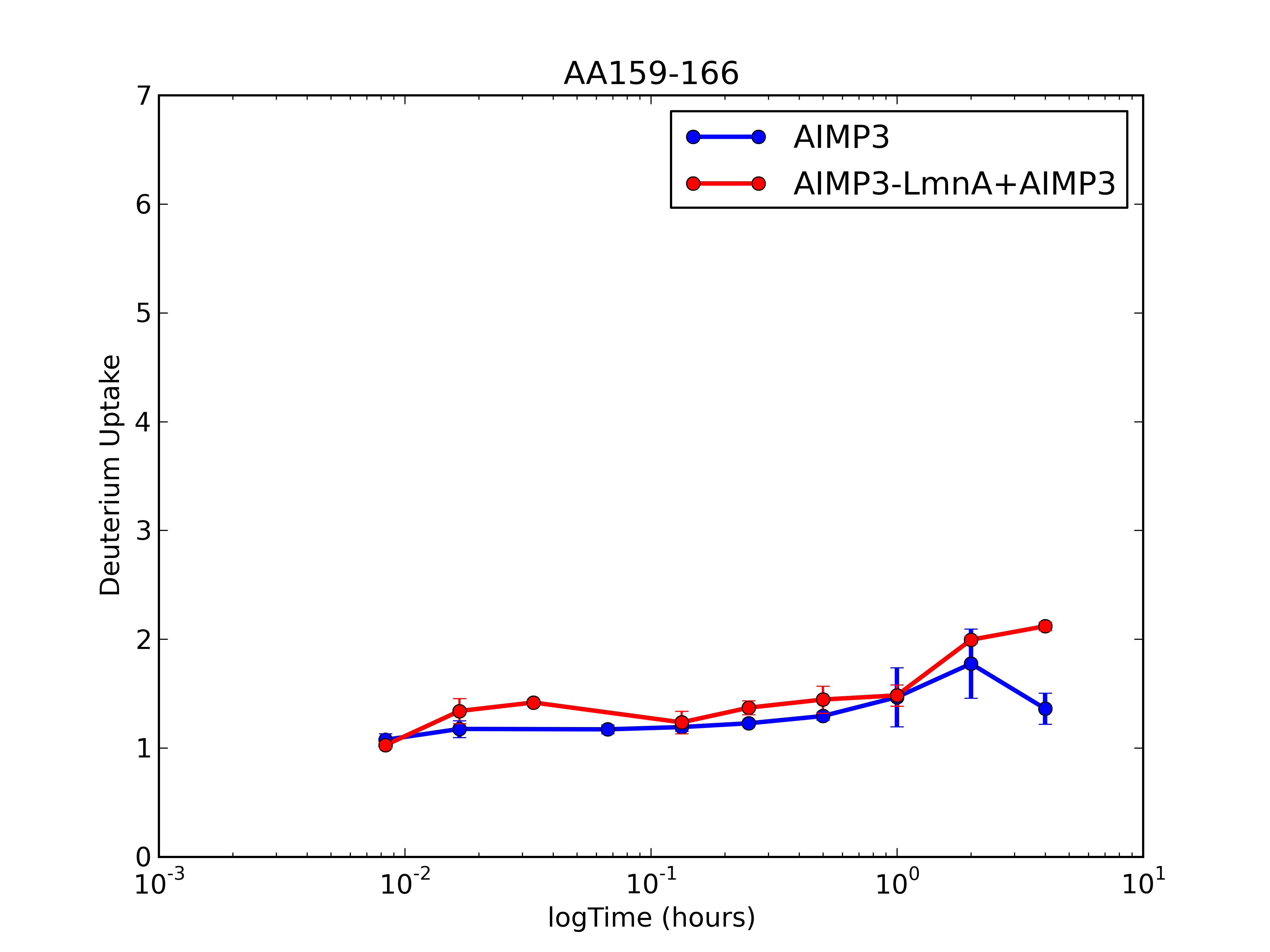

Supplement: S1 File — (ZIP) [file pone.0181869.s003.zip › logfigure-AIMP3-scale/AA159-166_charge_2_mz504.7.csv.csv.png]

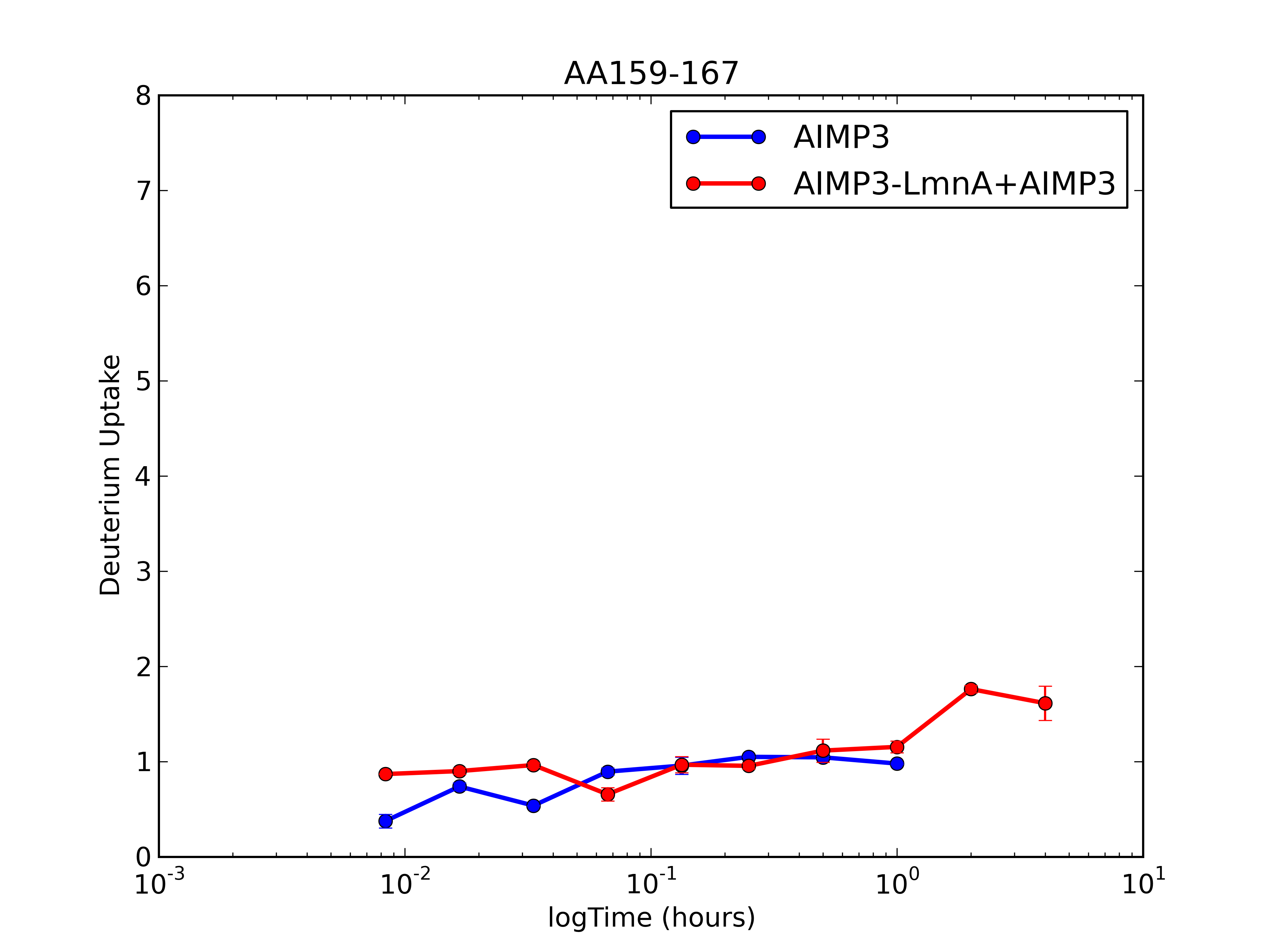

Supplement: S1 File — (ZIP) [file pone.0181869.s003.zip › logfigure-AIMP3-scale/AA159-167_charge_2_mz597.8.csv.csv.png]

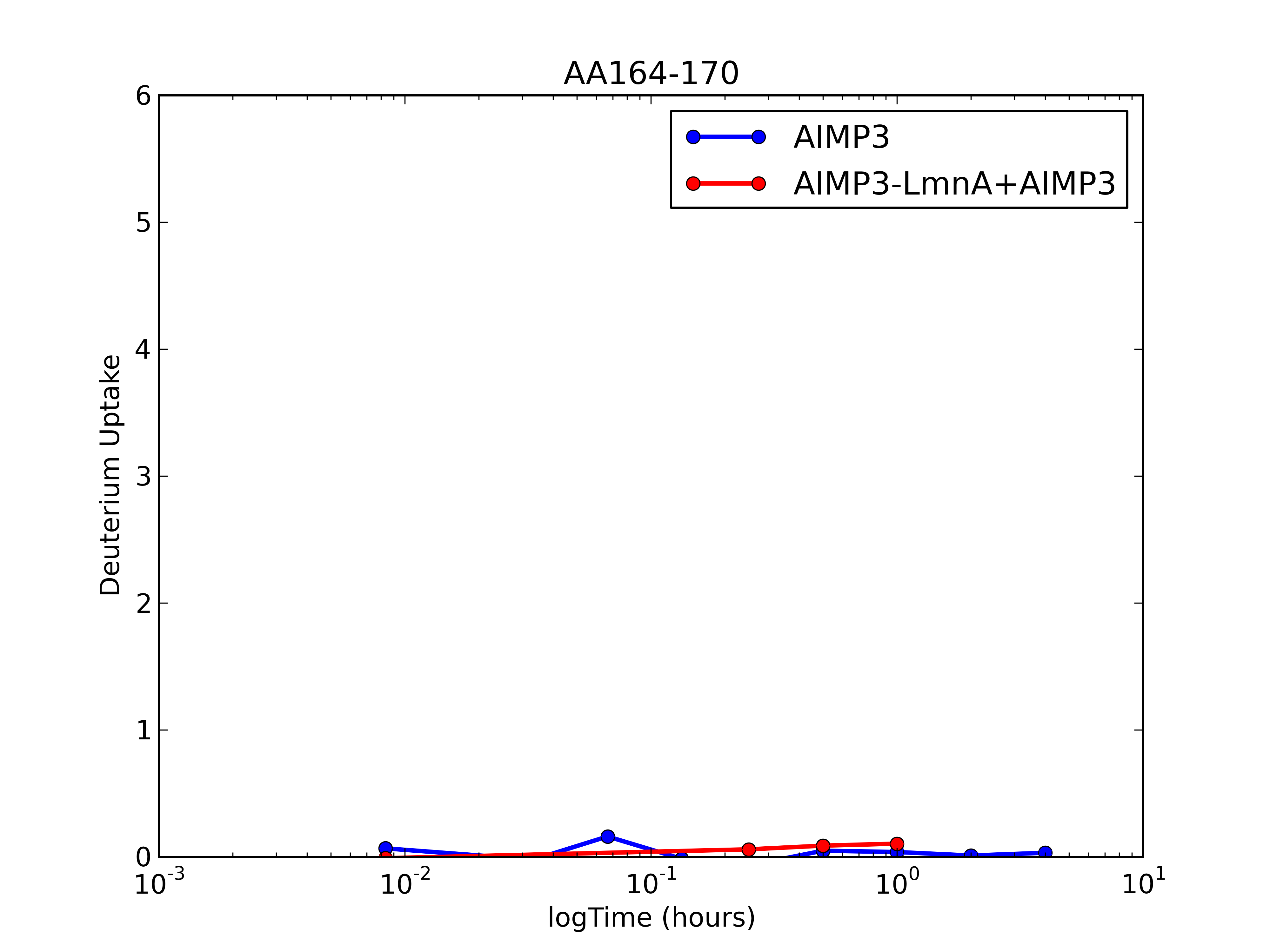

Supplement: S1 File — (ZIP) [file pone.0181869.s003.zip › logfigure-AIMP3-scale/AA164-170_charge_2_mz467.7.csv.csv.png]

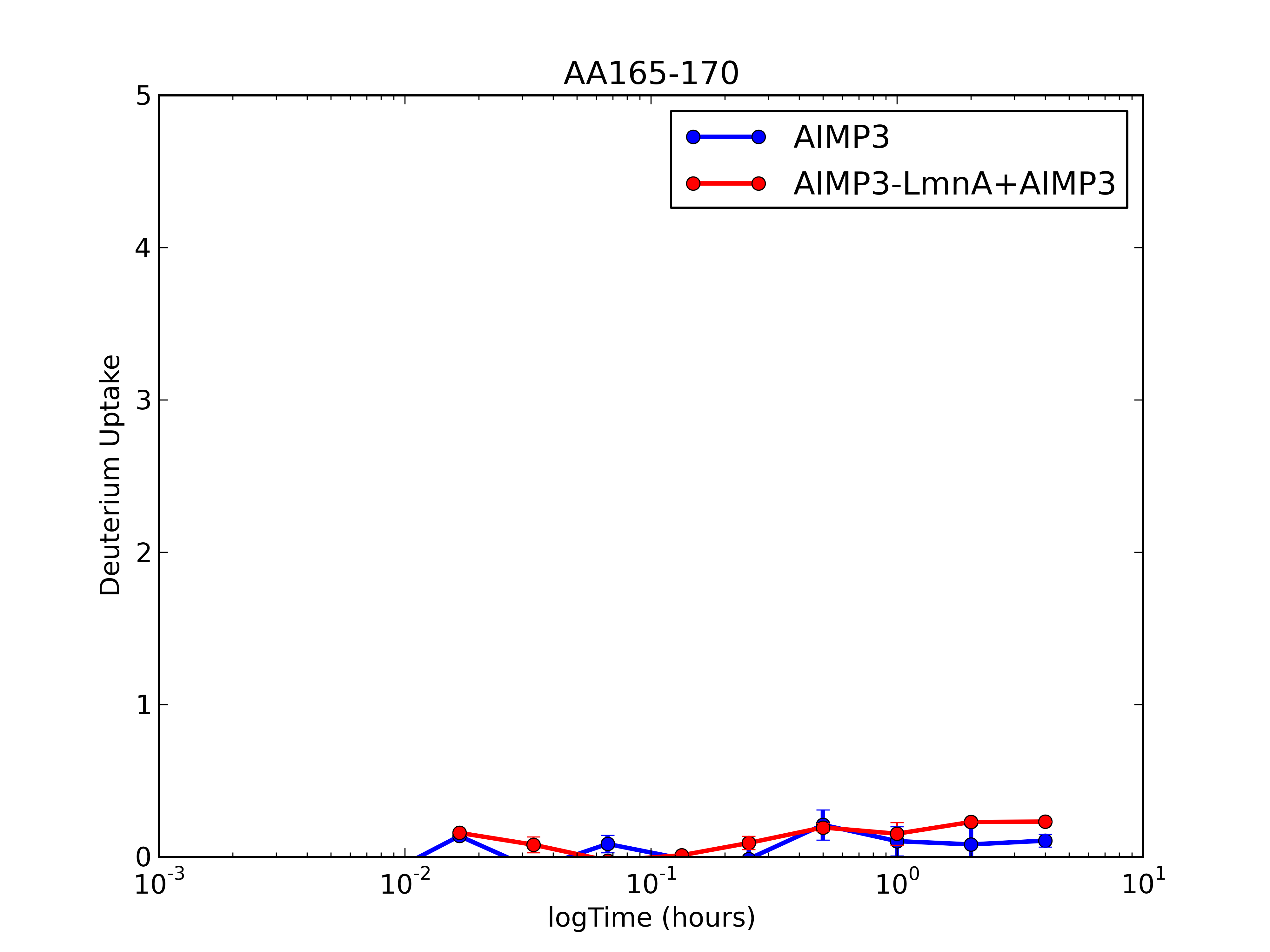

Supplement: S1 File — (ZIP) [file pone.0181869.s003.zip › logfigure-AIMP3-scale/AA165-170_charge_2_mz418.1.csv.csv.png]

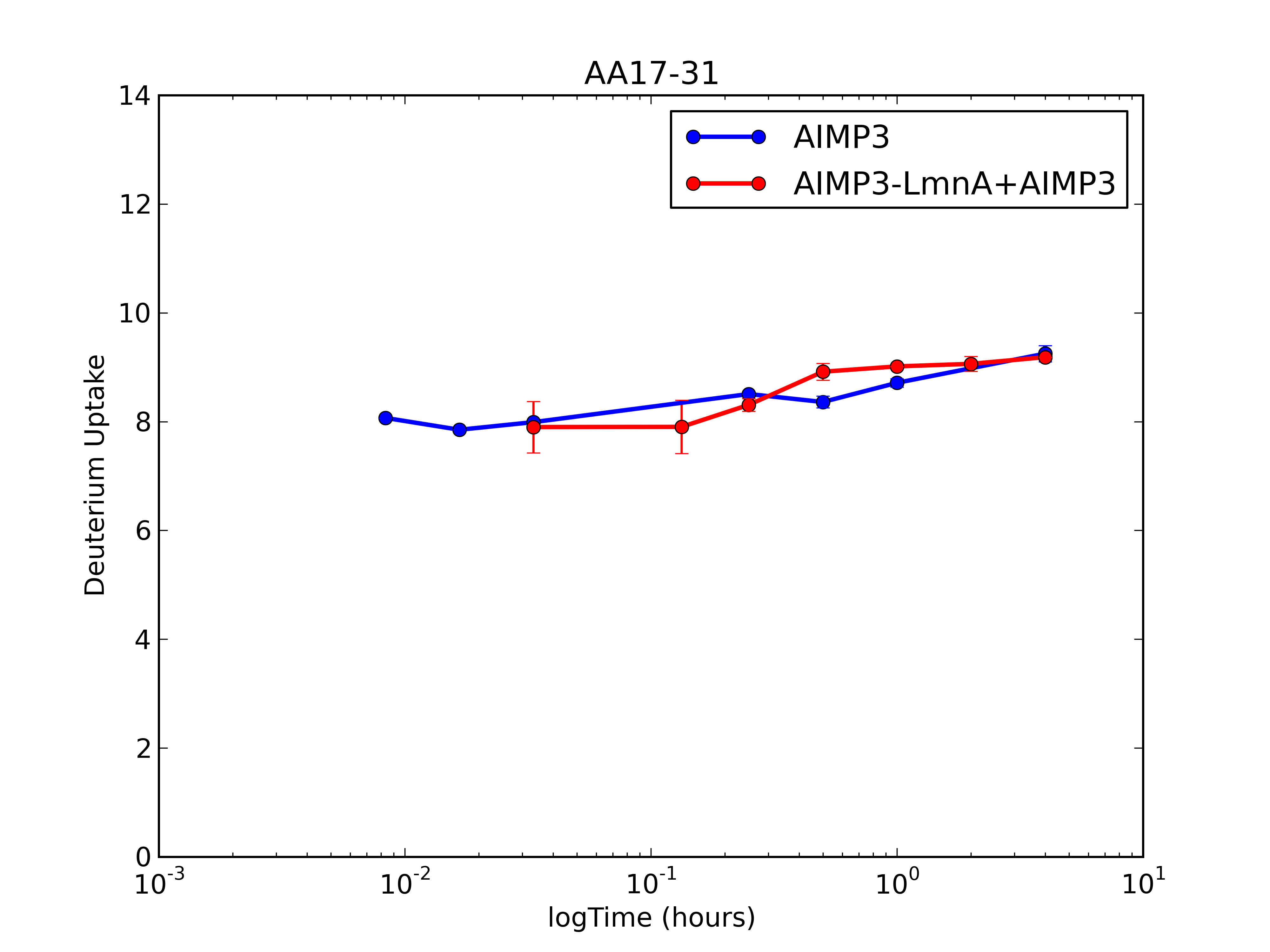

Supplement: S1 File — (ZIP) [file pone.0181869.s003.zip › logfigure-AIMP3-scale/AA17-31_charge_2_mz786.3.csv.csv.png]

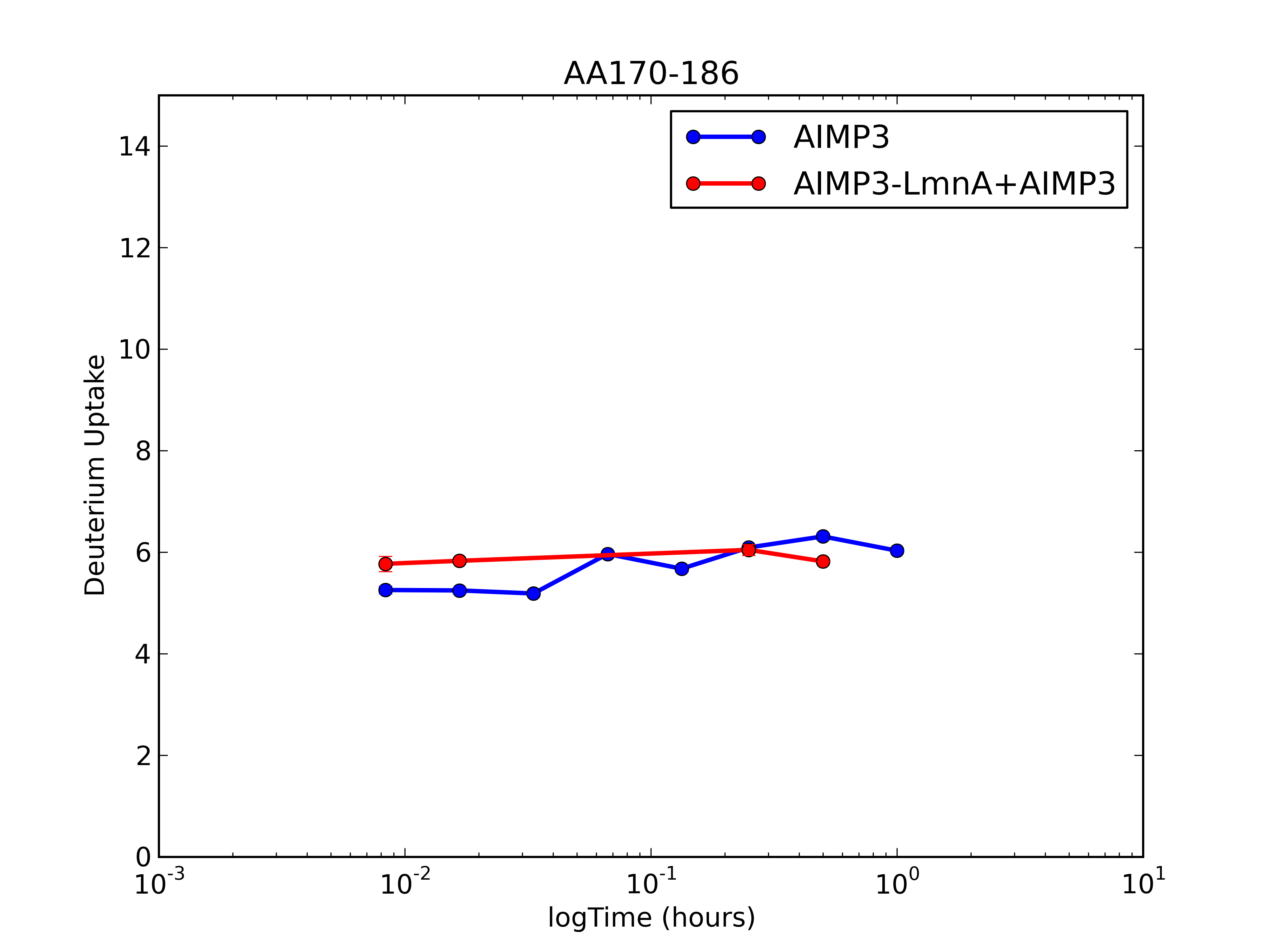

Supplement: S1 File — (ZIP) [file pone.0181869.s003.zip › logfigure-AIMP3-scale/AA170-186_charge_3_mz673.3.csv.csv.png]

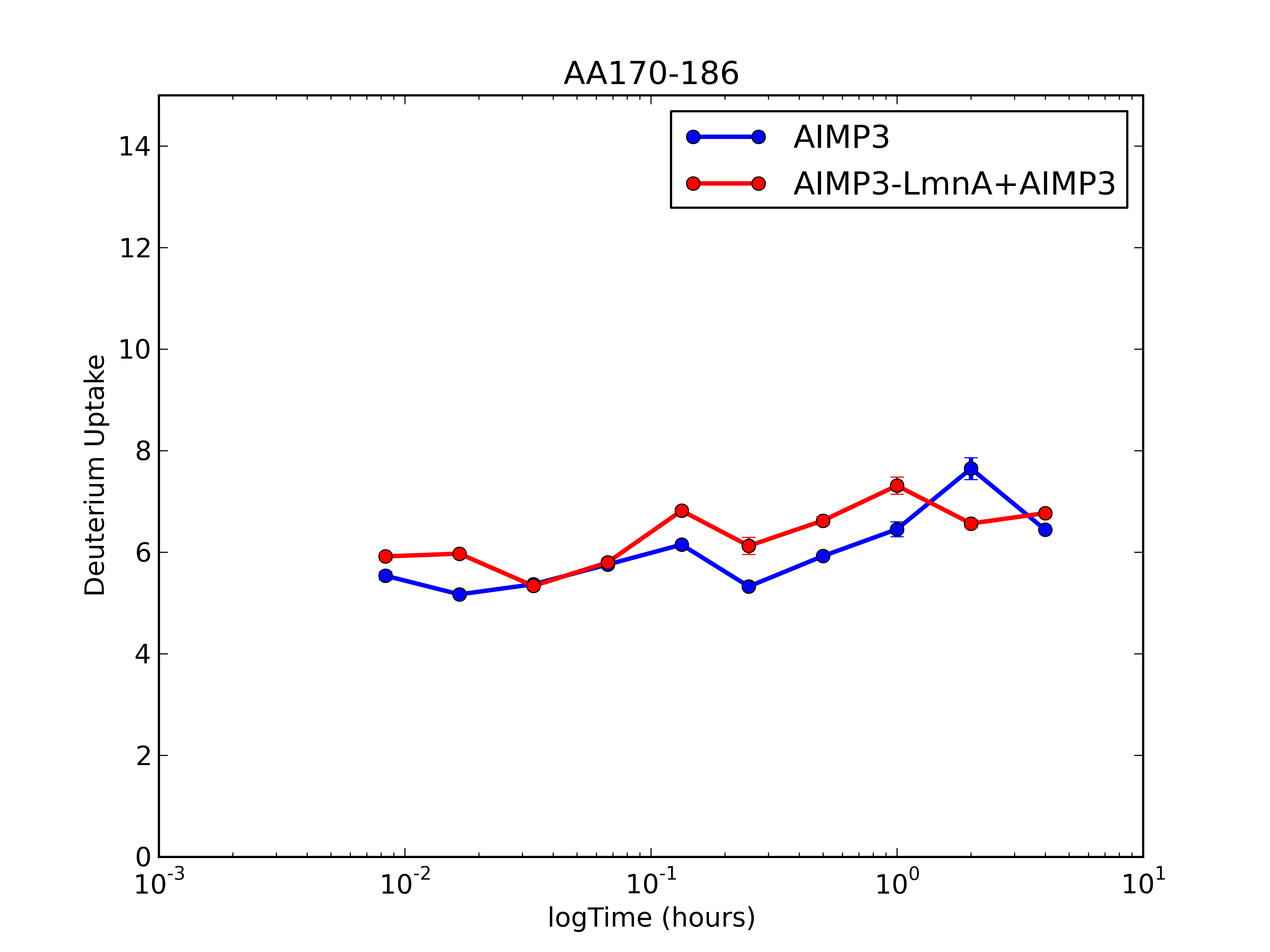

Supplement: S1 File — (ZIP) [file pone.0181869.s003.zip › logfigure-AIMP3-scale/AA170-186_charge_4_mz505.2.csv.csv.png]

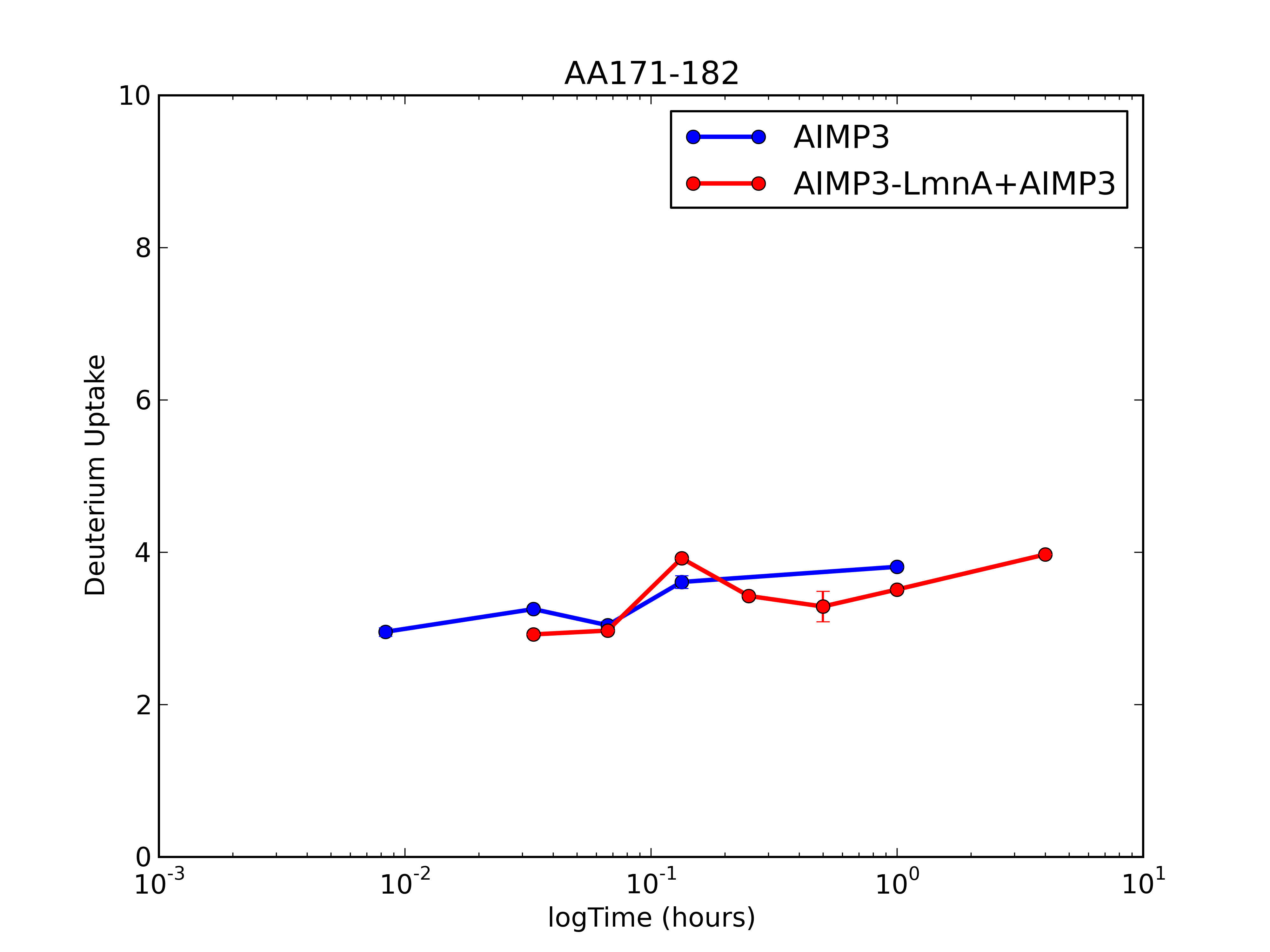

Supplement: S1 File — (ZIP) [file pone.0181869.s003.zip › logfigure-AIMP3-scale/AA171-182_charge_3_mz483.5.csv.csv.png]

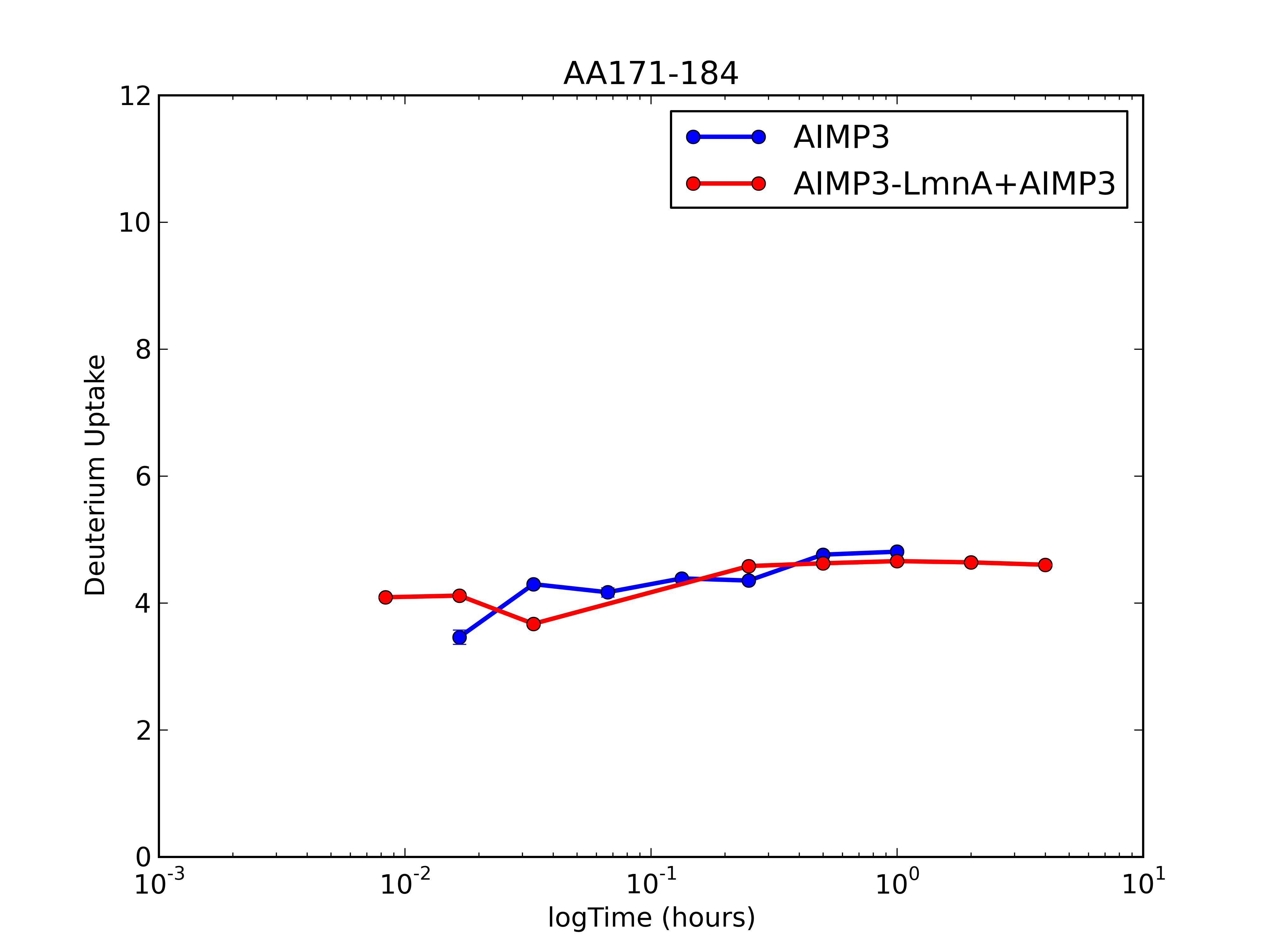

Supplement: S1 File — (ZIP) [file pone.0181869.s003.zip › logfigure-AIMP3-scale/AA171-184_charge_2_mz817.9.csv.csv.png]

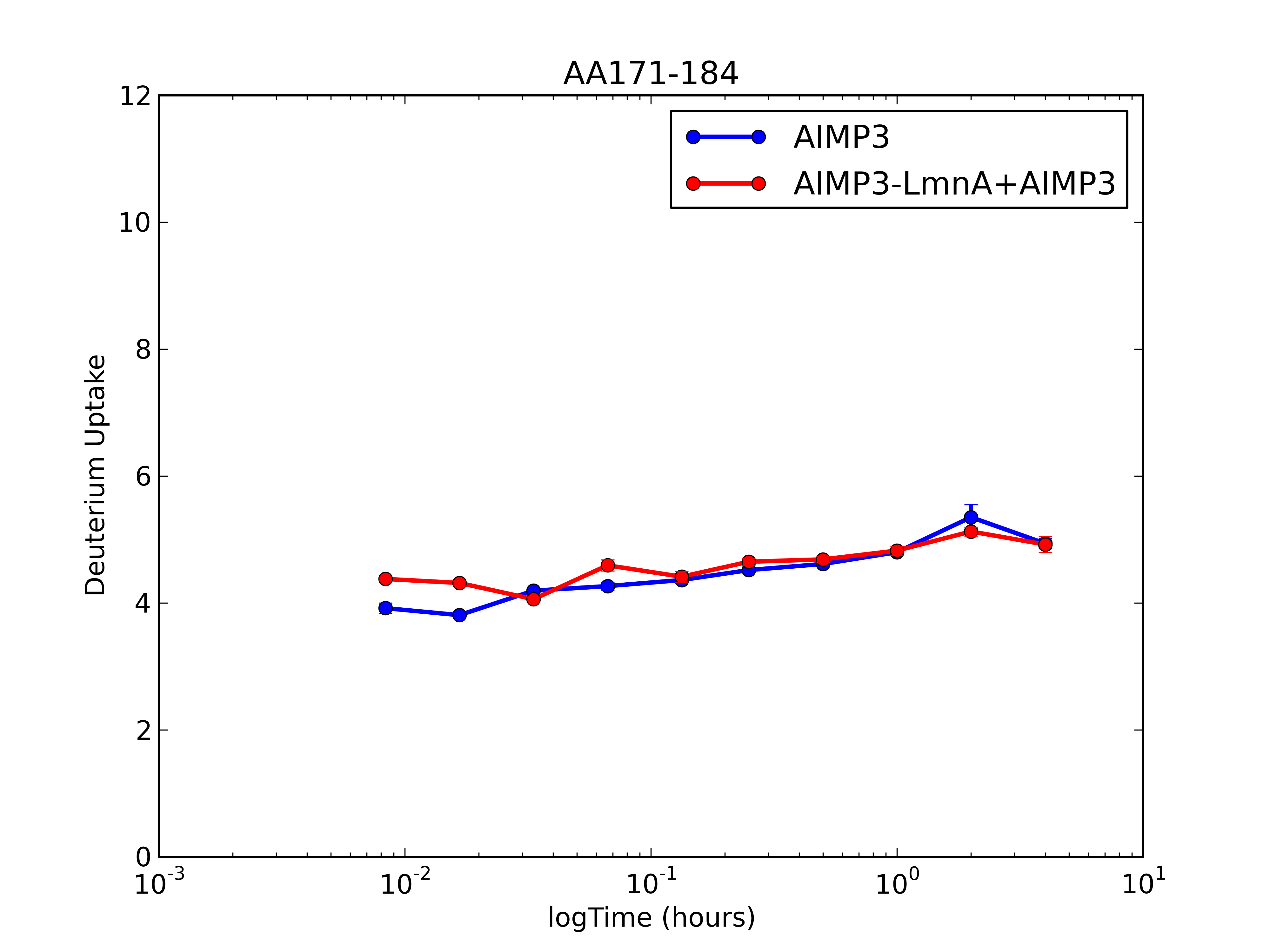

Supplement: S1 File — (ZIP) [file pone.0181869.s003.zip › logfigure-AIMP3-scale/AA171-184_charge_3_mz545.6.csv.csv.png]

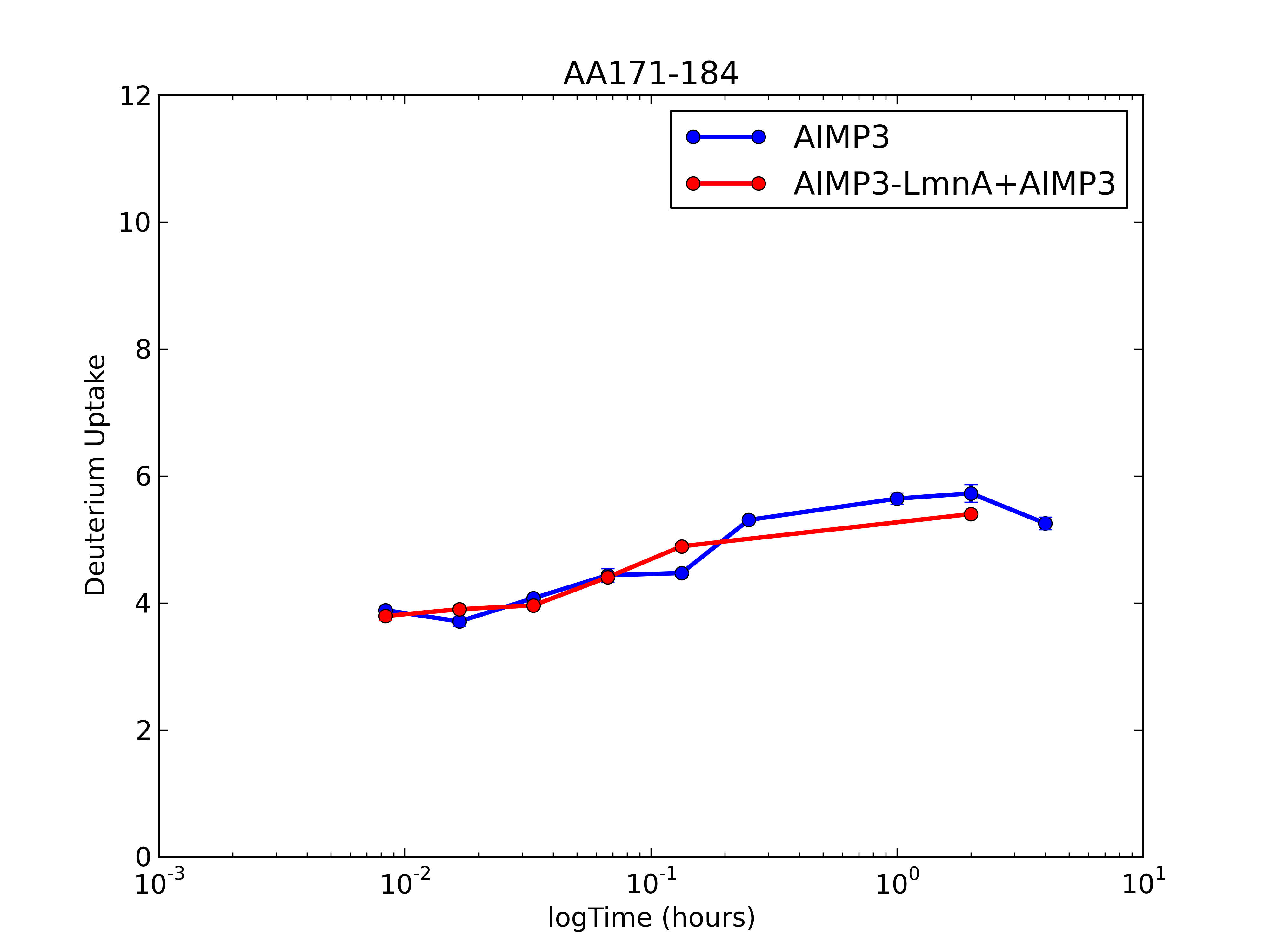

Supplement: S1 File — (ZIP) [file pone.0181869.s003.zip › logfigure-AIMP3-scale/AA171-184_charge_4_mz409.4.csv.csv.png]

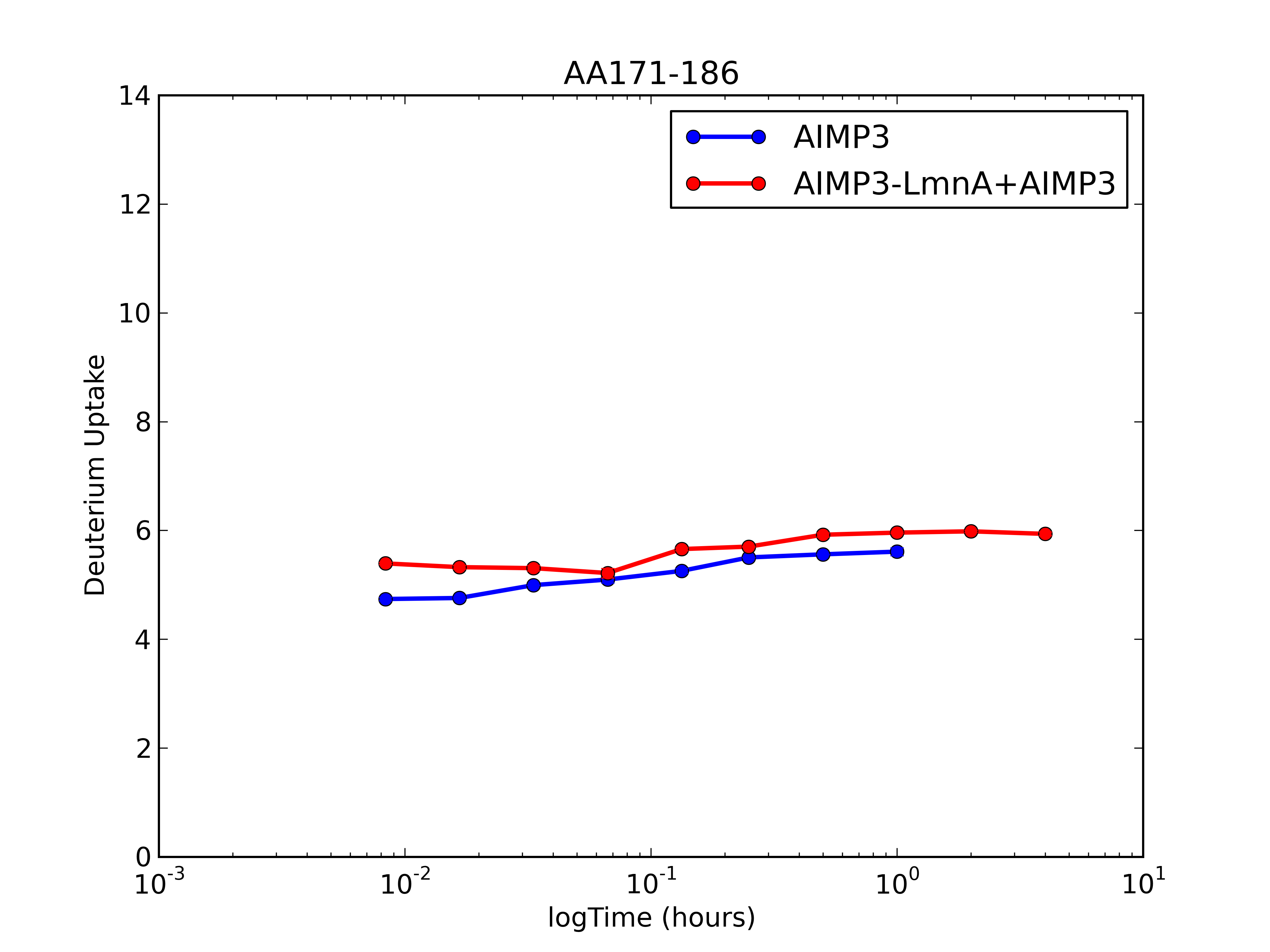

Supplement: S1 File — (ZIP) [file pone.0181869.s003.zip › logfigure-AIMP3-scale/AA171-186_charge_3_mz627.6.csv.csv.png]

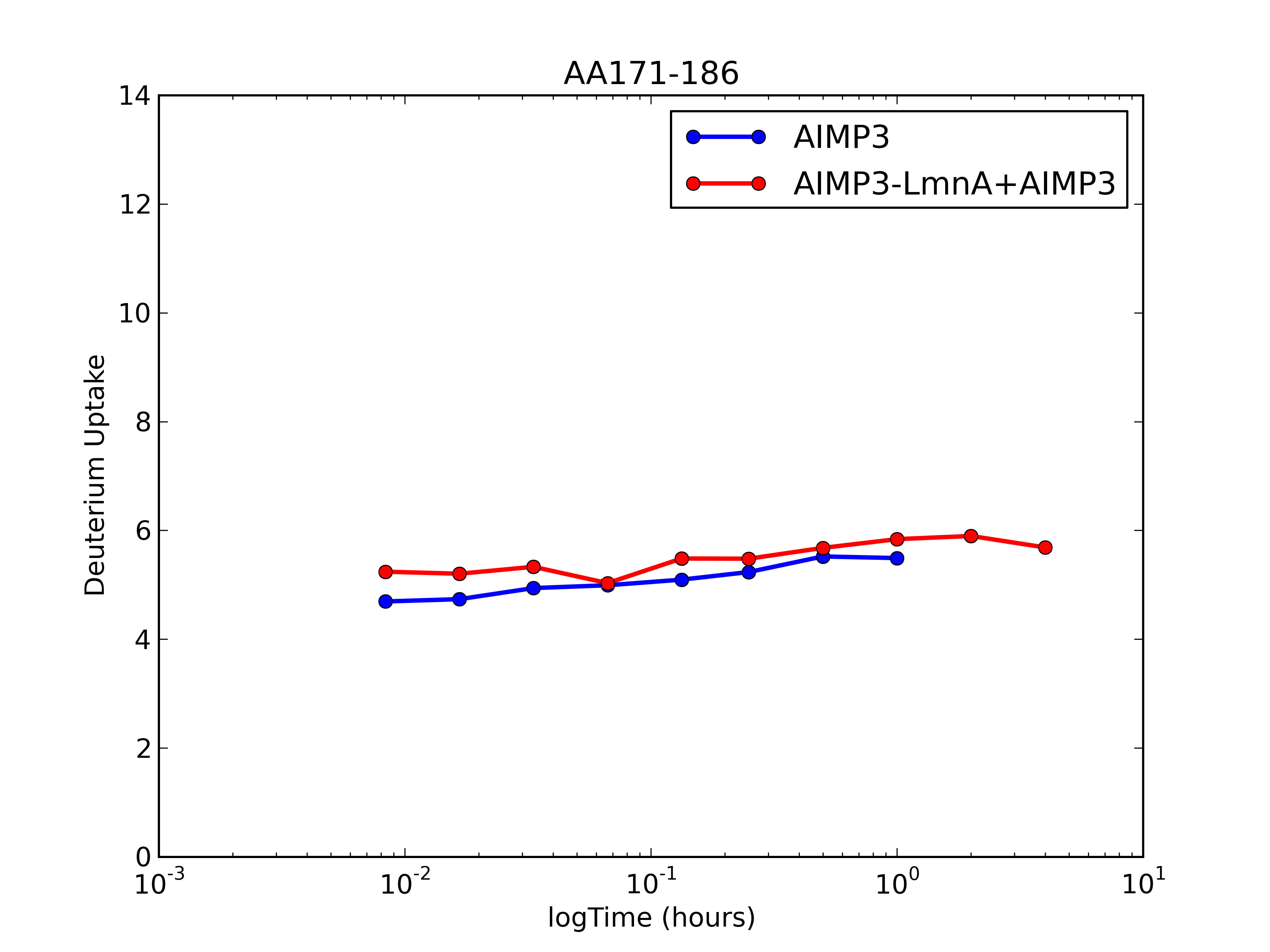

Supplement: S1 File — (ZIP) [file pone.0181869.s003.zip › logfigure-AIMP3-scale/AA171-186_charge_4_mz471.0.csv.csv.png]

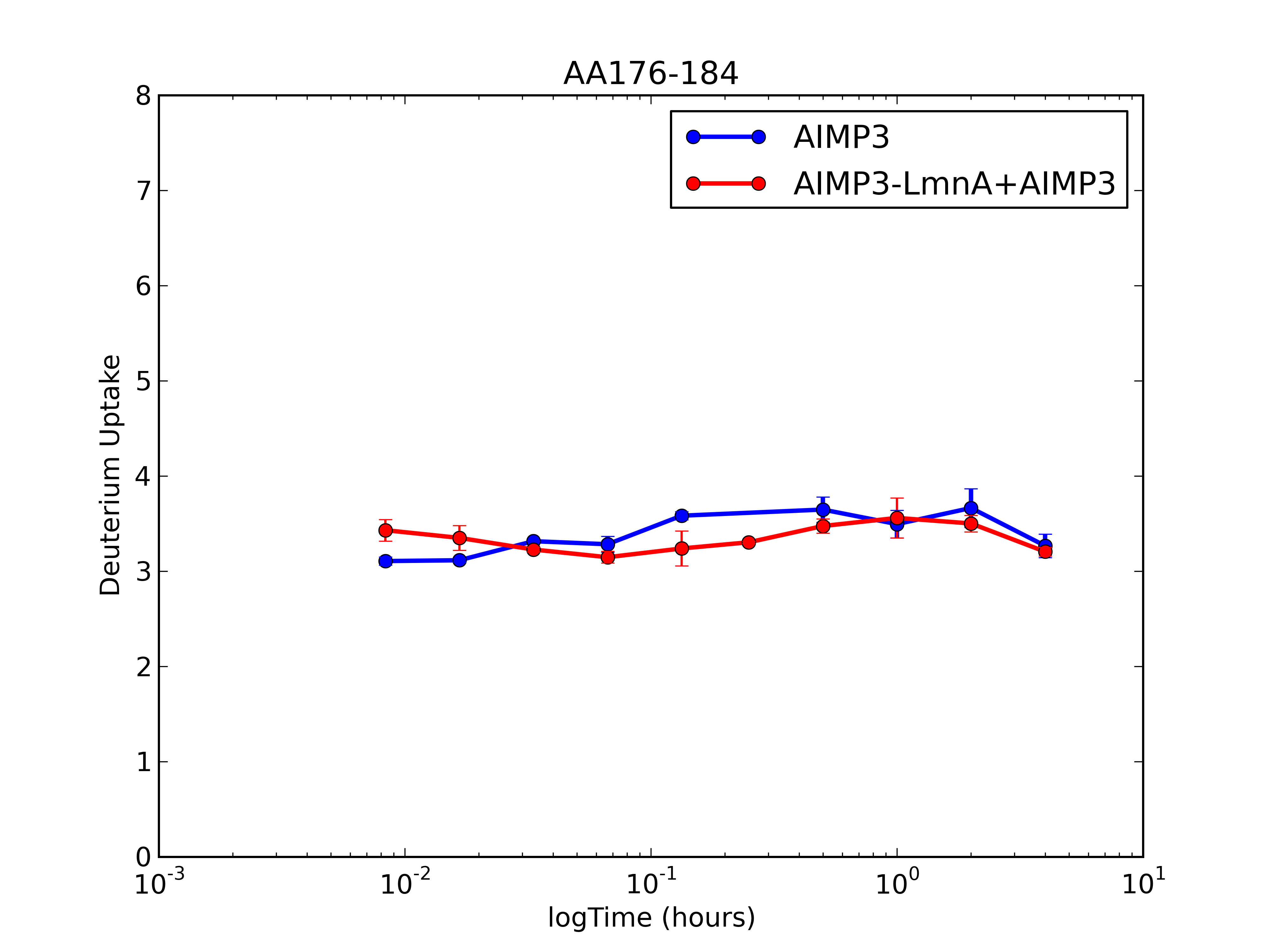

Supplement: S1 File — (ZIP) [file pone.0181869.s003.zip › logfigure-AIMP3-scale/AA176-184_charge_2_mz498.7.csv.csv.png]

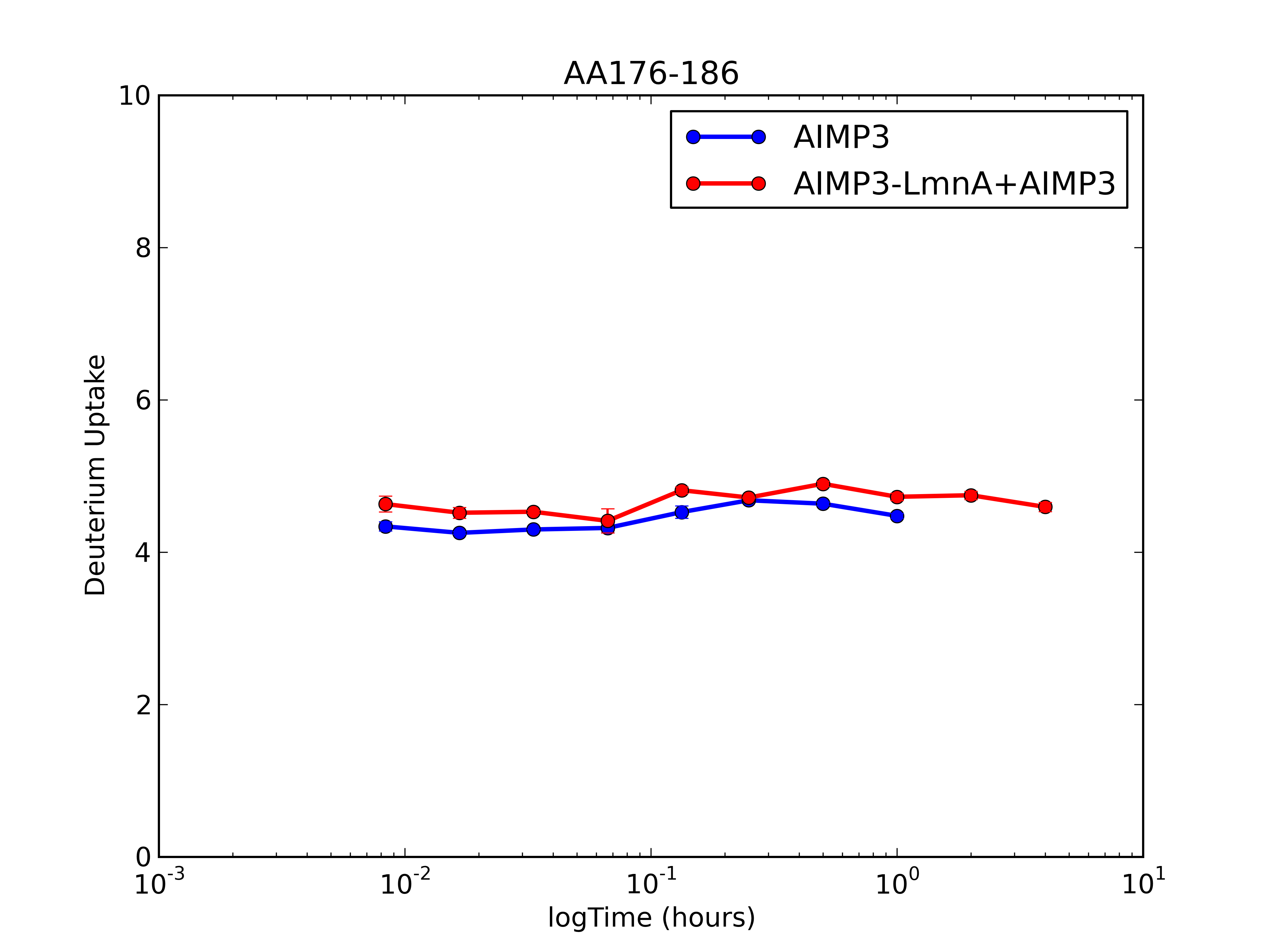

Supplement: S1 File — (ZIP) [file pone.0181869.s003.zip › logfigure-AIMP3-scale/AA176-186_charge_2_mz621.8.csv.csv.png]

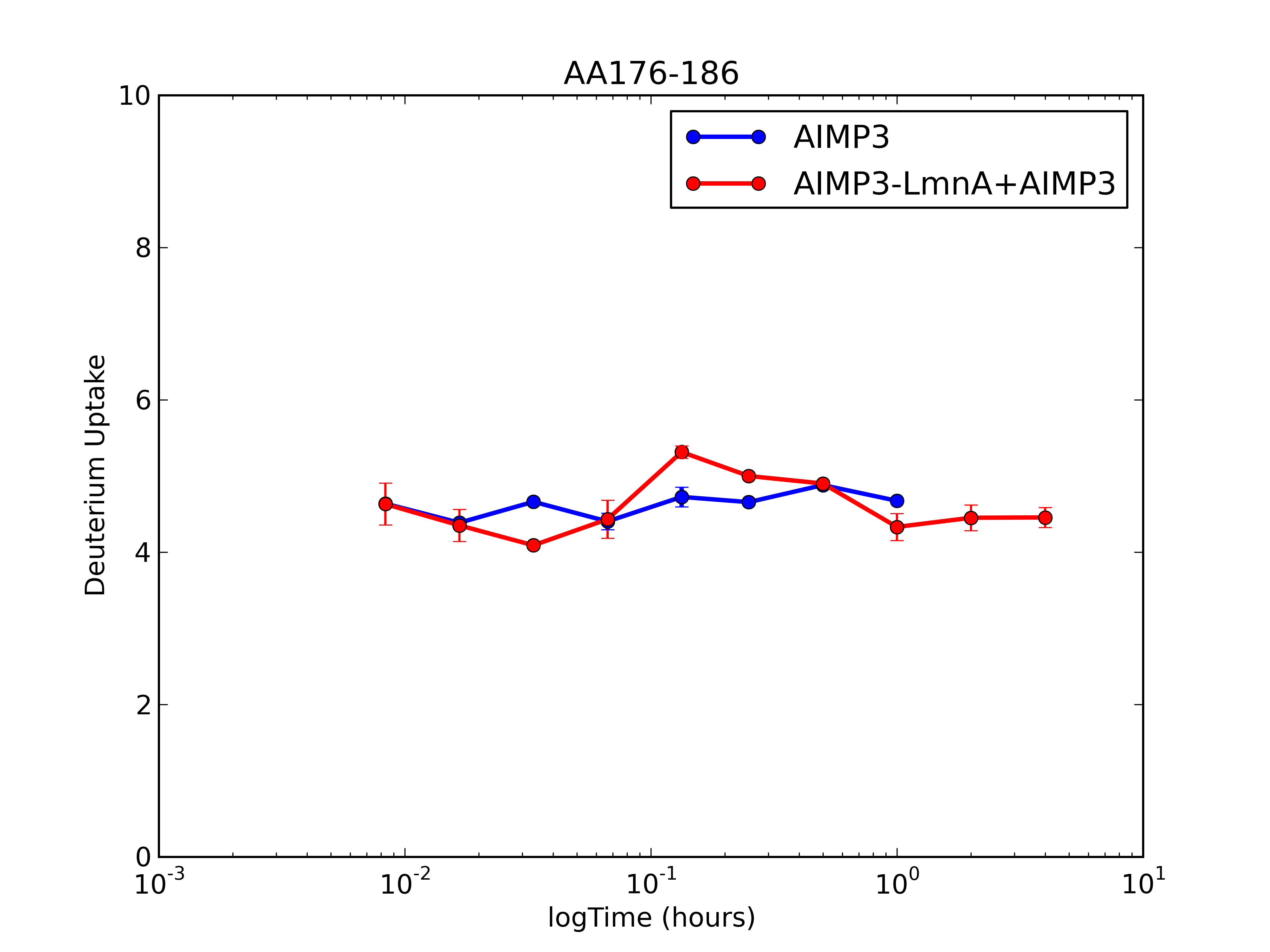

Supplement: S1 File — (ZIP) [file pone.0181869.s003.zip › logfigure-AIMP3-scale/AA176-186_charge_3_mz414.9.csv.csv.png]

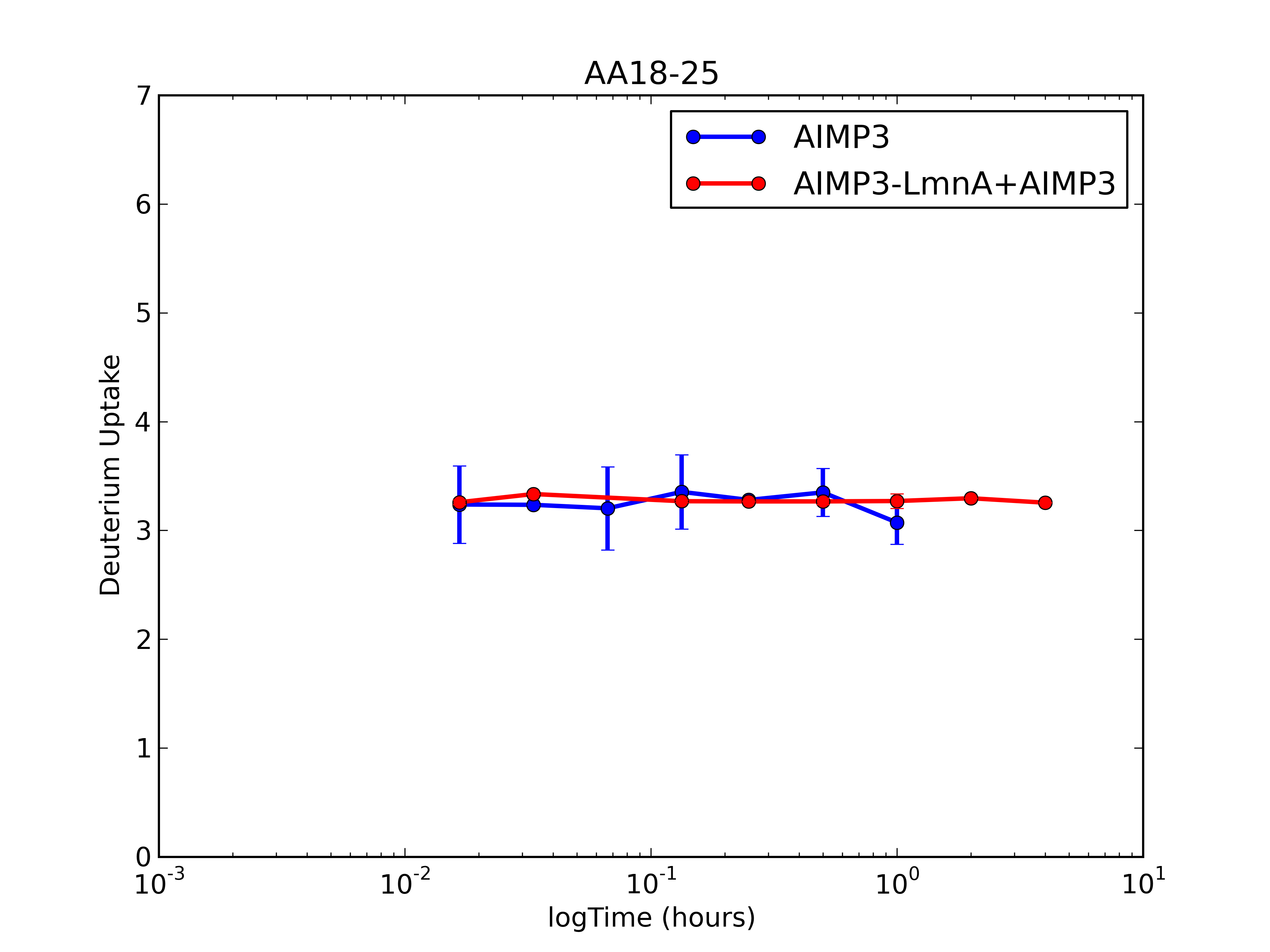

Supplement: S1 File — (ZIP) [file pone.0181869.s003.zip › logfigure-AIMP3-scale/AA18-25_charge_1_mz874.3.csv.csv.png]

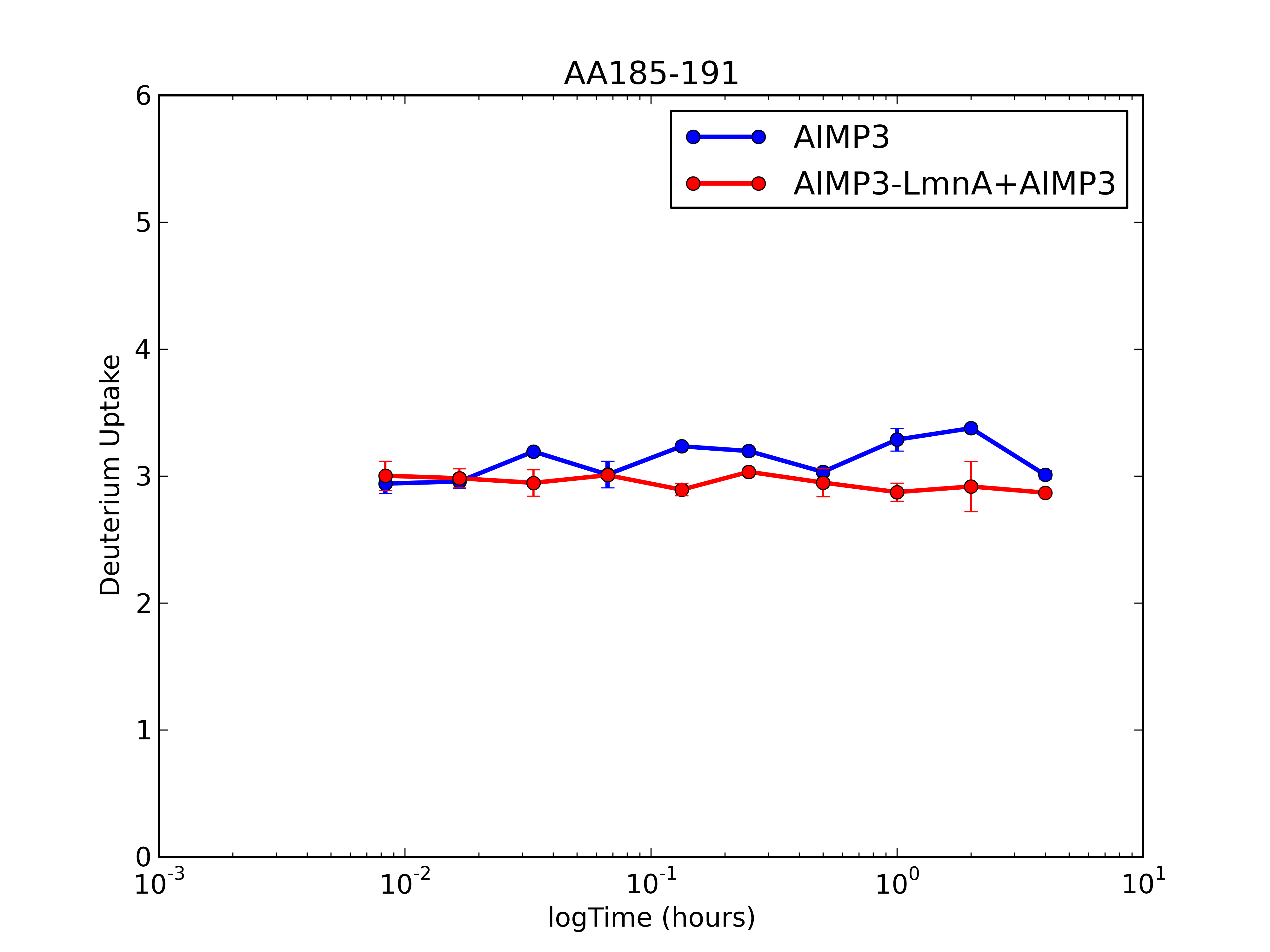

Supplement: S1 File — (ZIP) [file pone.0181869.s003.zip › logfigure-AIMP3-scale/AA185-191_charge_2_mz445.2.csv.csv.png]

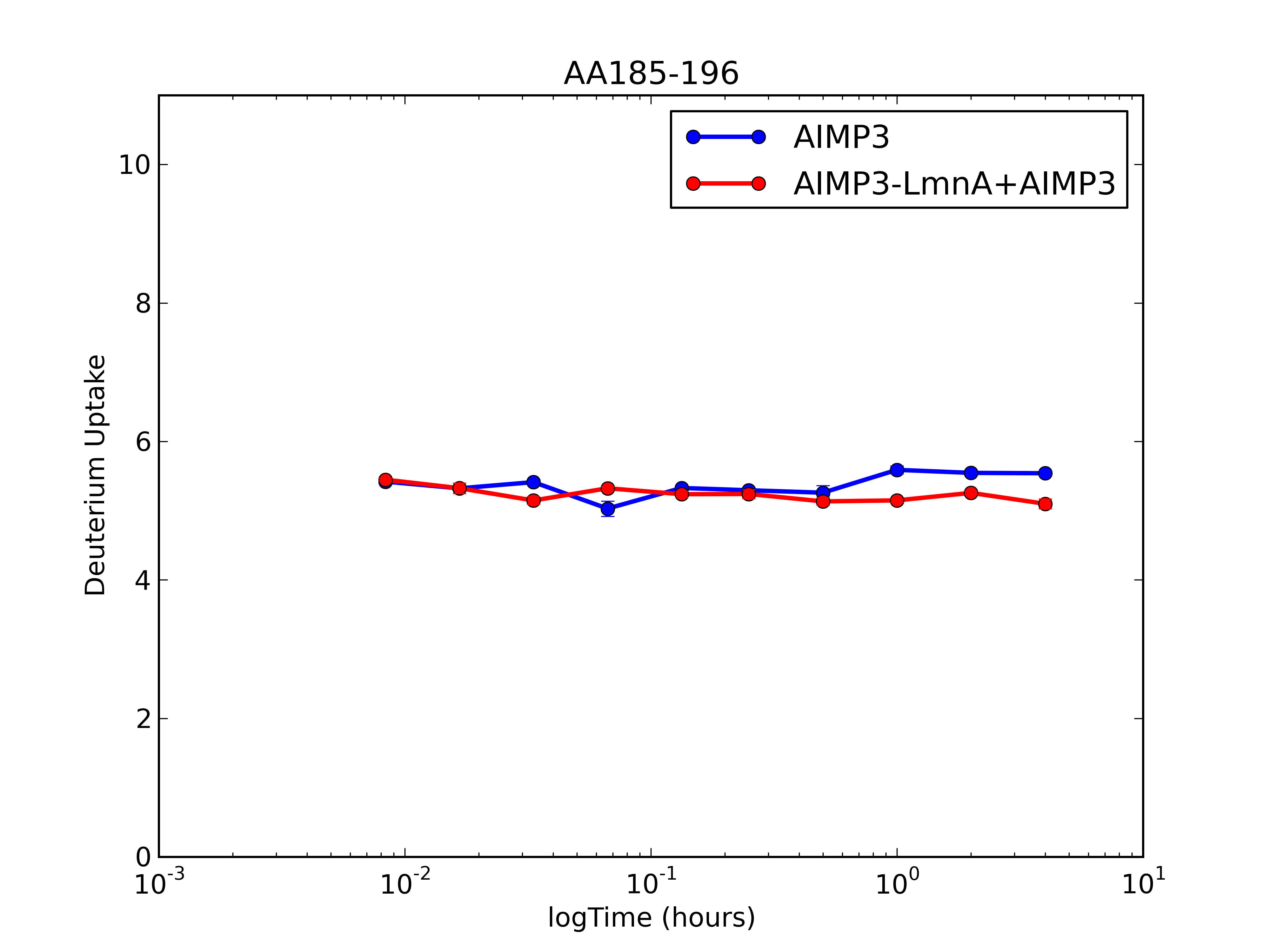

Supplement: S1 File — (ZIP) [file pone.0181869.s003.zip › logfigure-AIMP3-scale/AA185-196_charge_3_mz497.9.csv.csv.png]

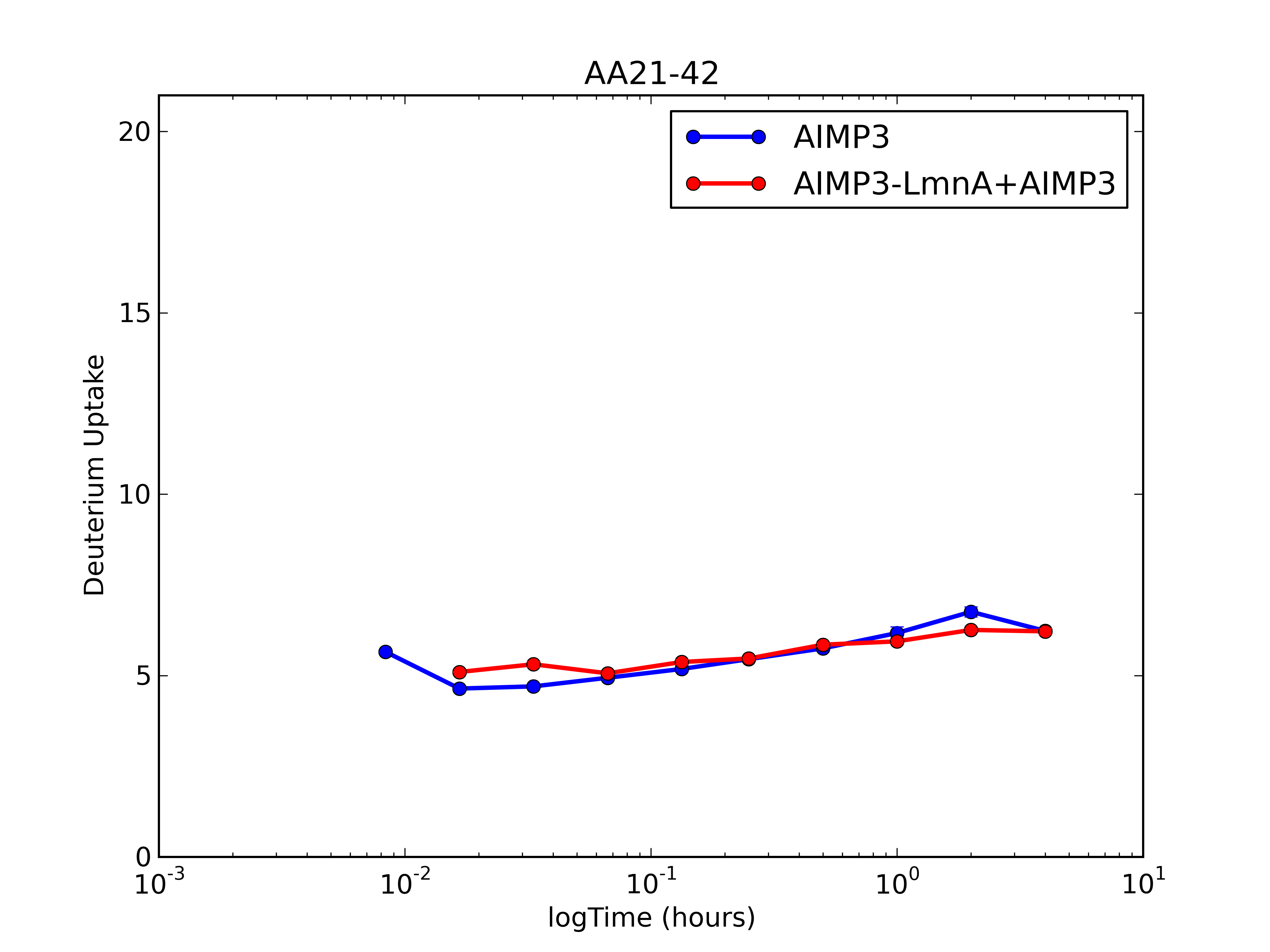

Supplement: S1 File — (ZIP) [file pone.0181869.s003.zip › logfigure-AIMP3-scale/AA21-42_charge_5_mz430.2.csv.csv.png]

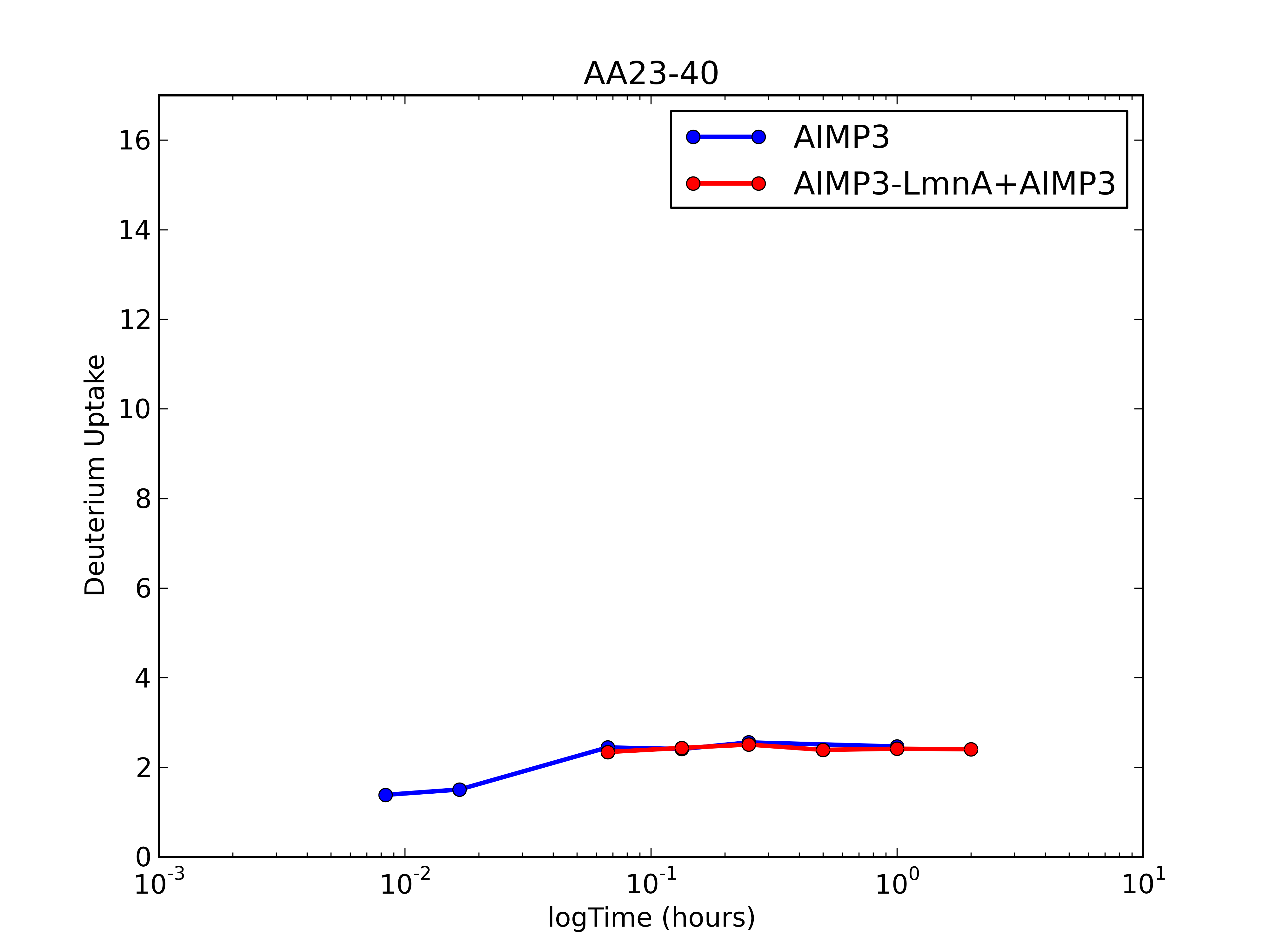

Supplement: S1 File — (ZIP) [file pone.0181869.s003.zip › logfigure-AIMP3-scale/AA23-40_charge_3_mz611.3.csv.csv.png]

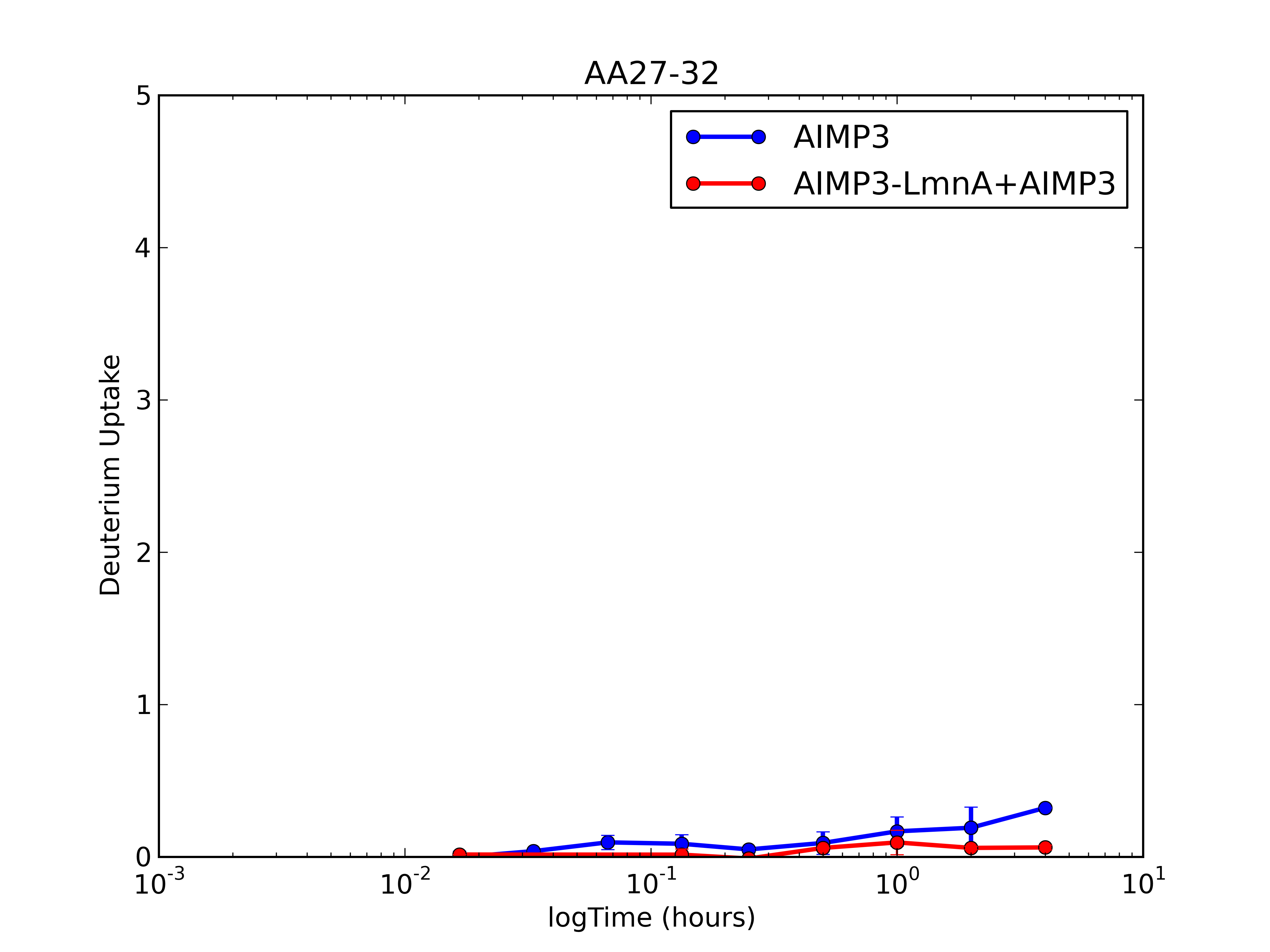

Supplement: S1 File — (ZIP) [file pone.0181869.s003.zip › logfigure-AIMP3-scale/AA27-32_charge_1_mz645.3.csv.csv.png]

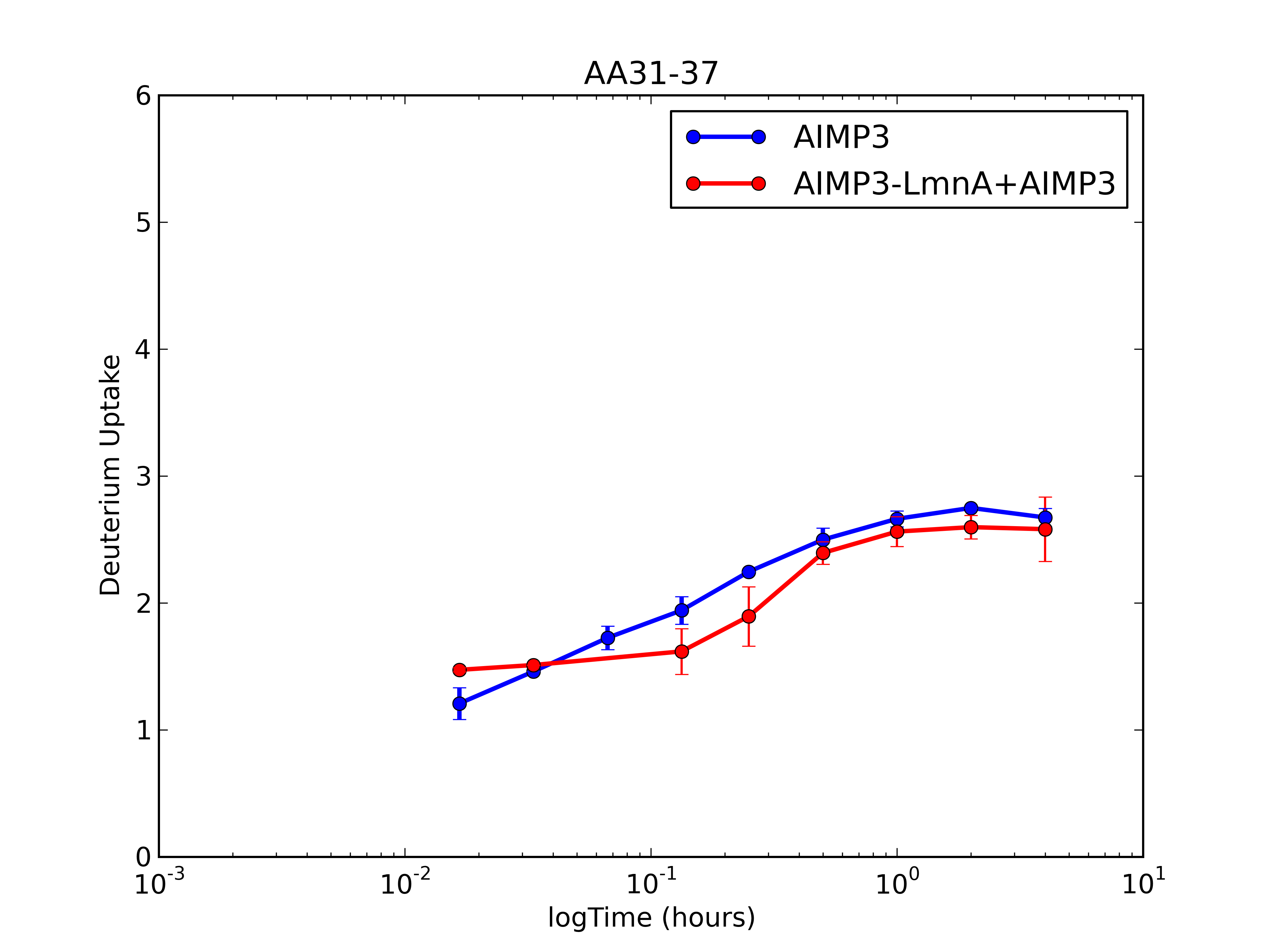

Supplement: S1 File — (ZIP) [file pone.0181869.s003.zip › logfigure-AIMP3-scale/AA31-37_charge_1_mz759.4.csv.csv.png]

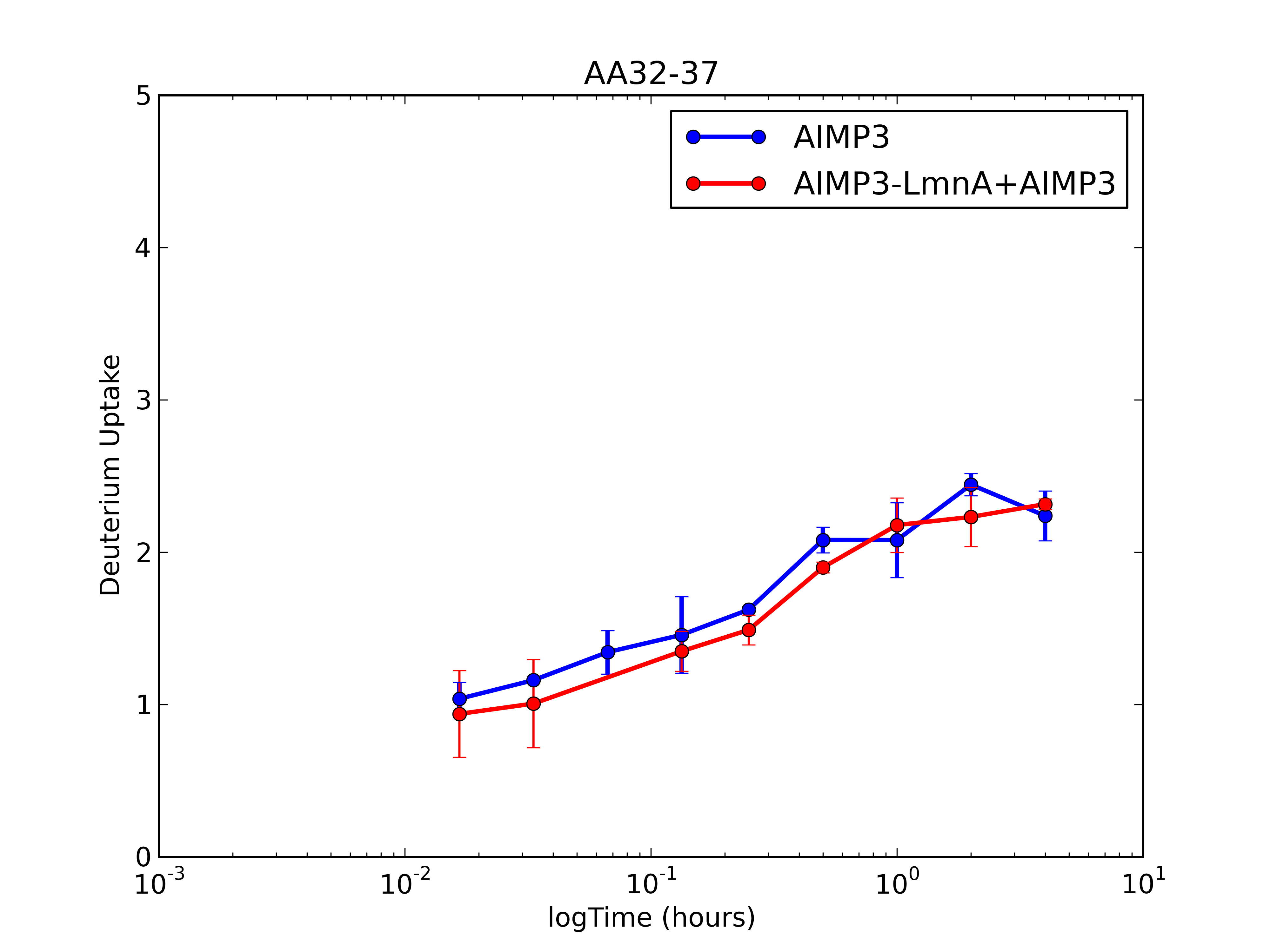

Supplement: S1 File — (ZIP) [file pone.0181869.s003.zip › logfigure-AIMP3-scale/AA32-37_charge_1_mz646.3.csv.csv.png]

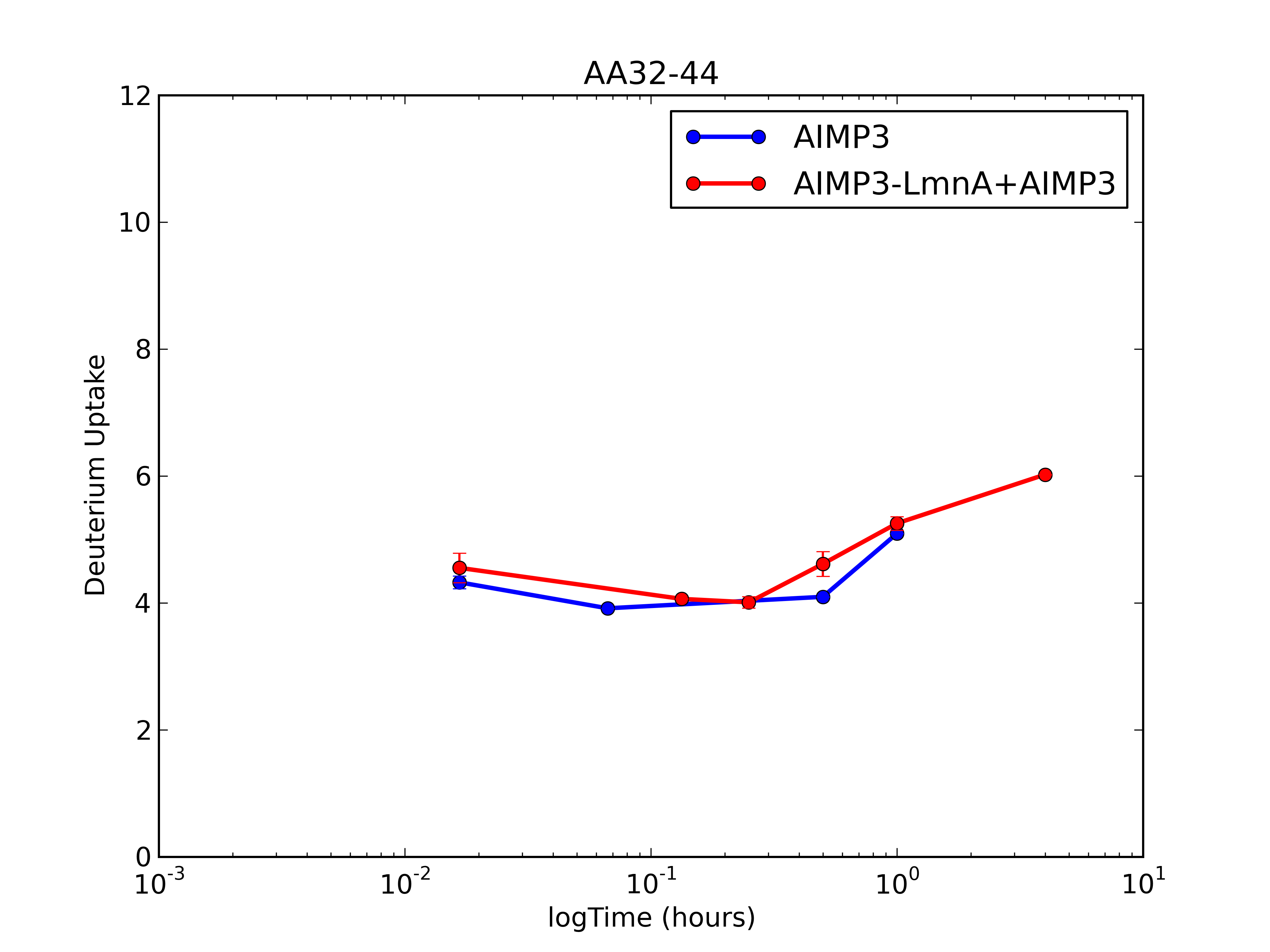

Supplement: S1 File — (ZIP) [file pone.0181869.s003.zip › logfigure-AIMP3-scale/AA32-44_charge_3_mz479.6.csv.csv.png]

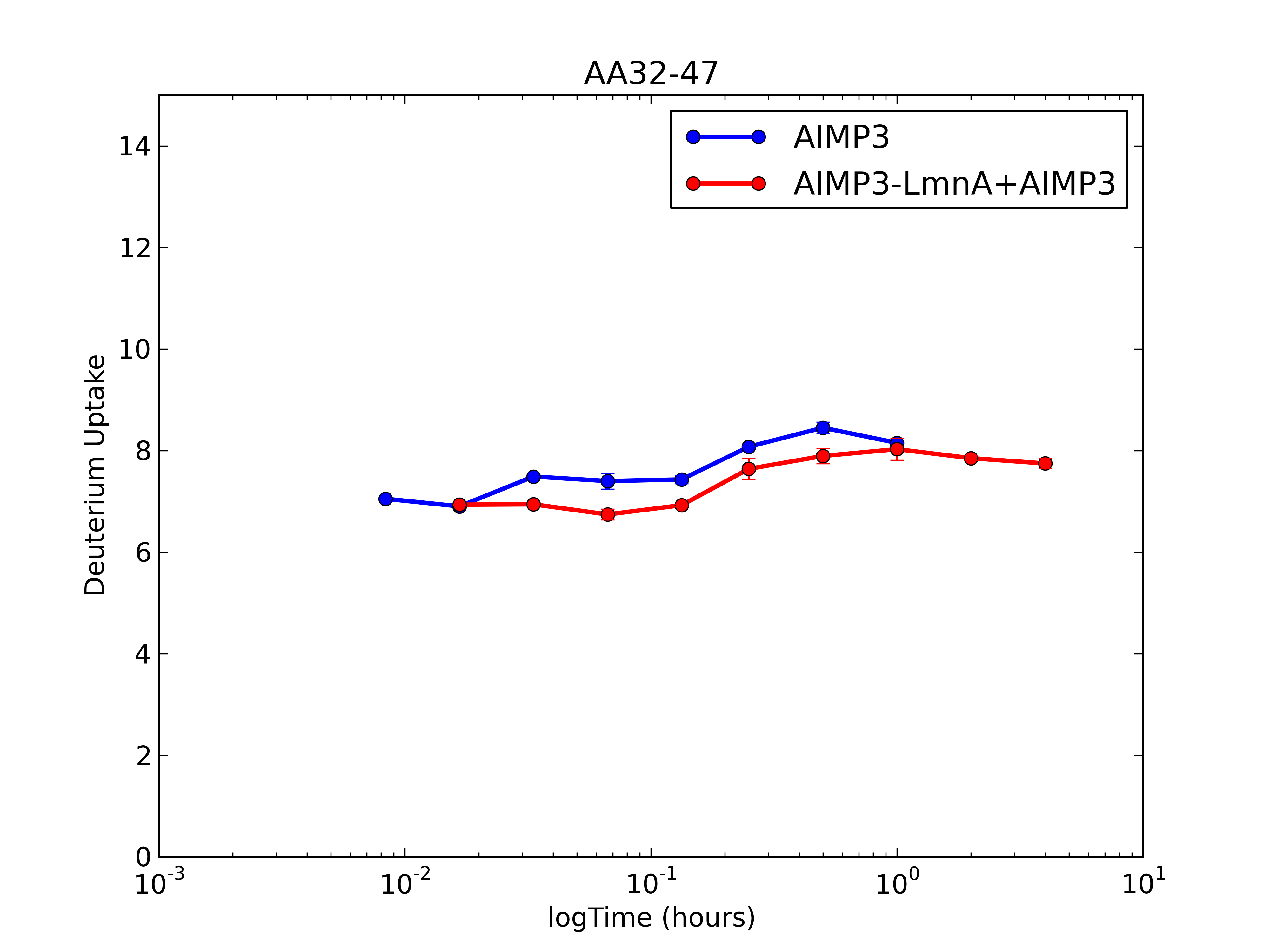

Supplement: S1 File — (ZIP) [file pone.0181869.s003.zip › logfigure-AIMP3-scale/AA32-47_charge_3_mz574.9.csv.csv.png]

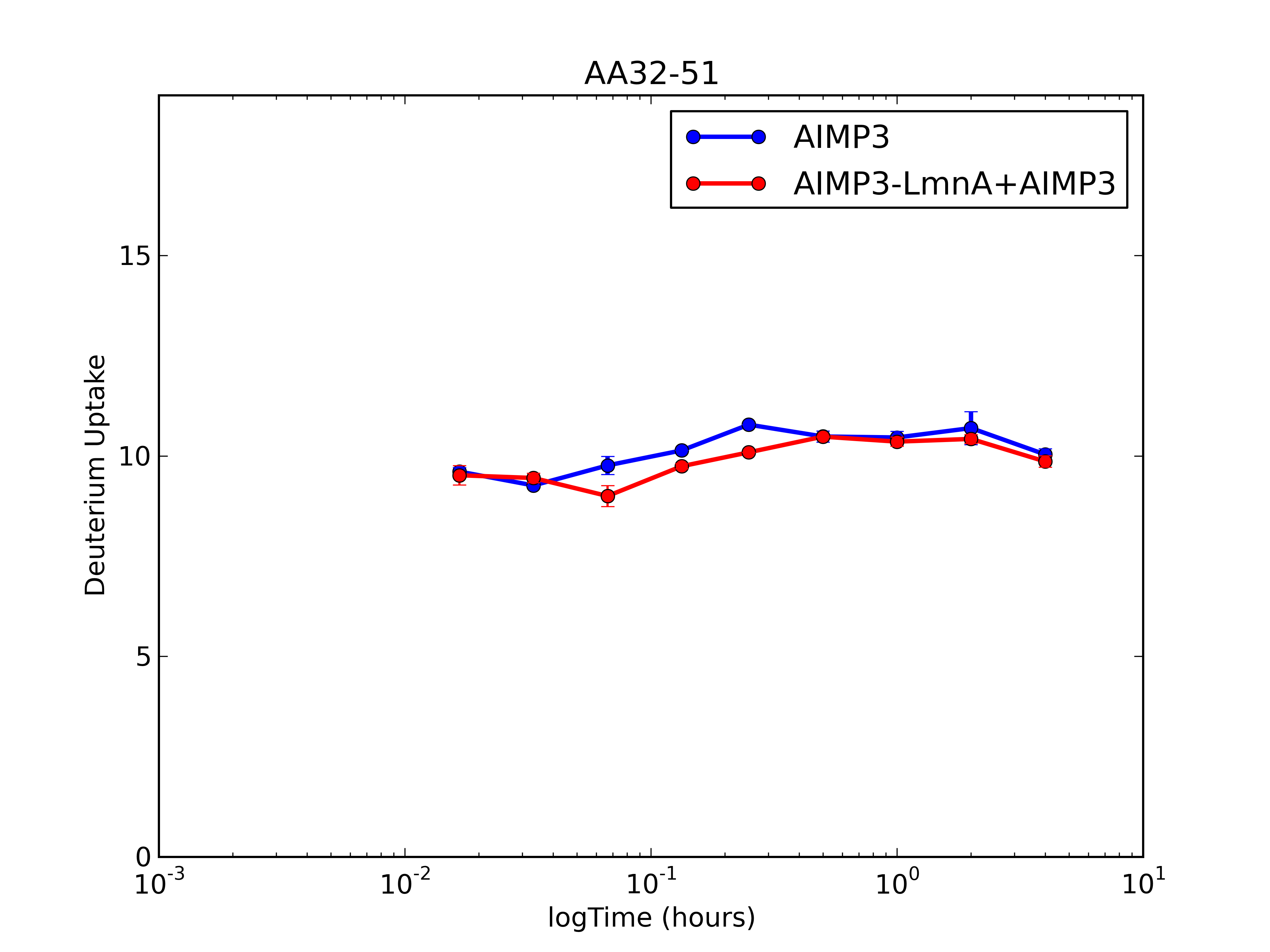

Supplement: S1 File — (ZIP) [file pone.0181869.s003.zip › logfigure-AIMP3-scale/AA32-51_charge_2_mz1097.0.csv.csv.png]

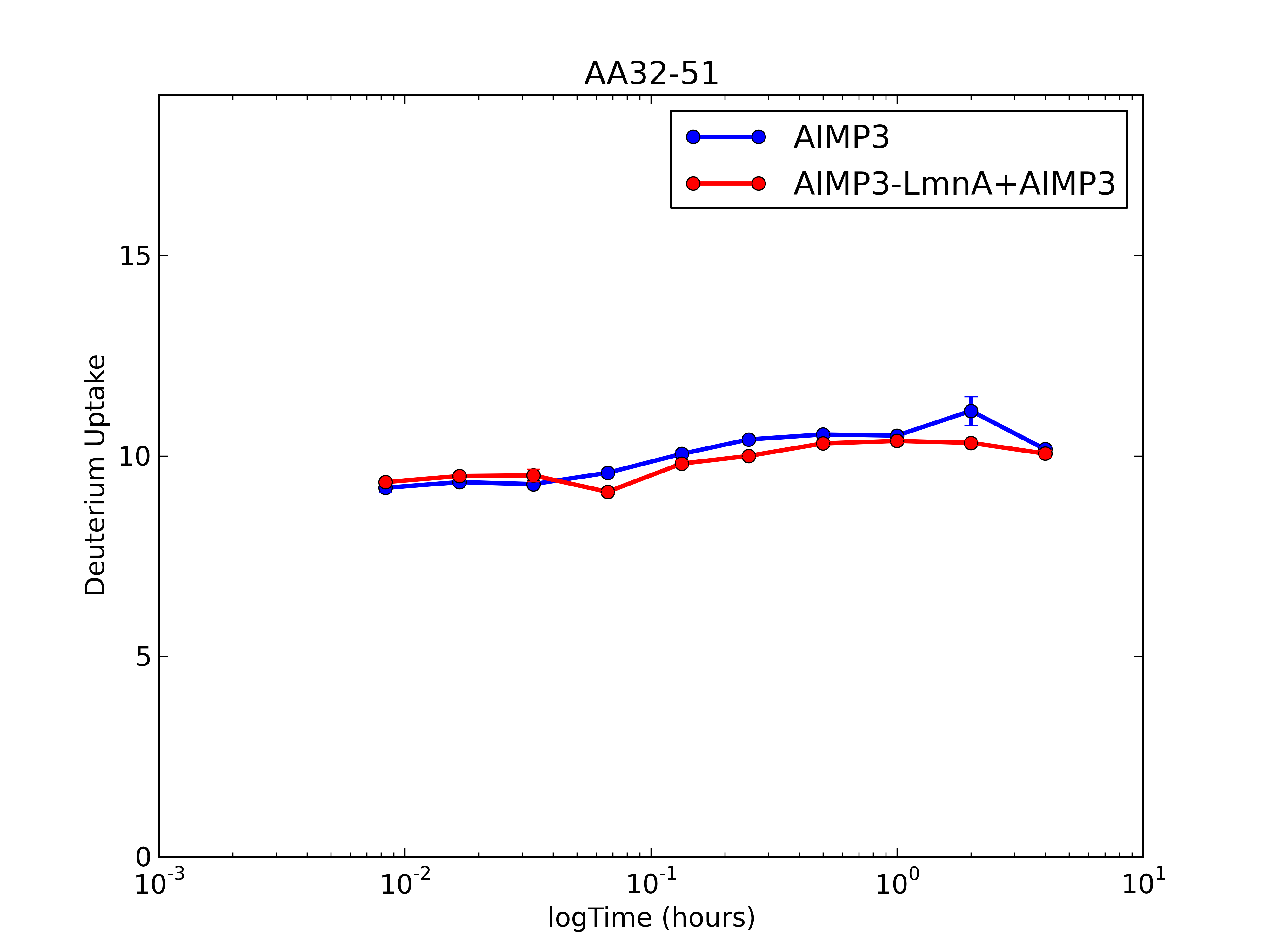

Supplement: S1 File — (ZIP) [file pone.0181869.s003.zip › logfigure-AIMP3-scale/AA32-51_charge_3_mz731.7.csv.csv.png]

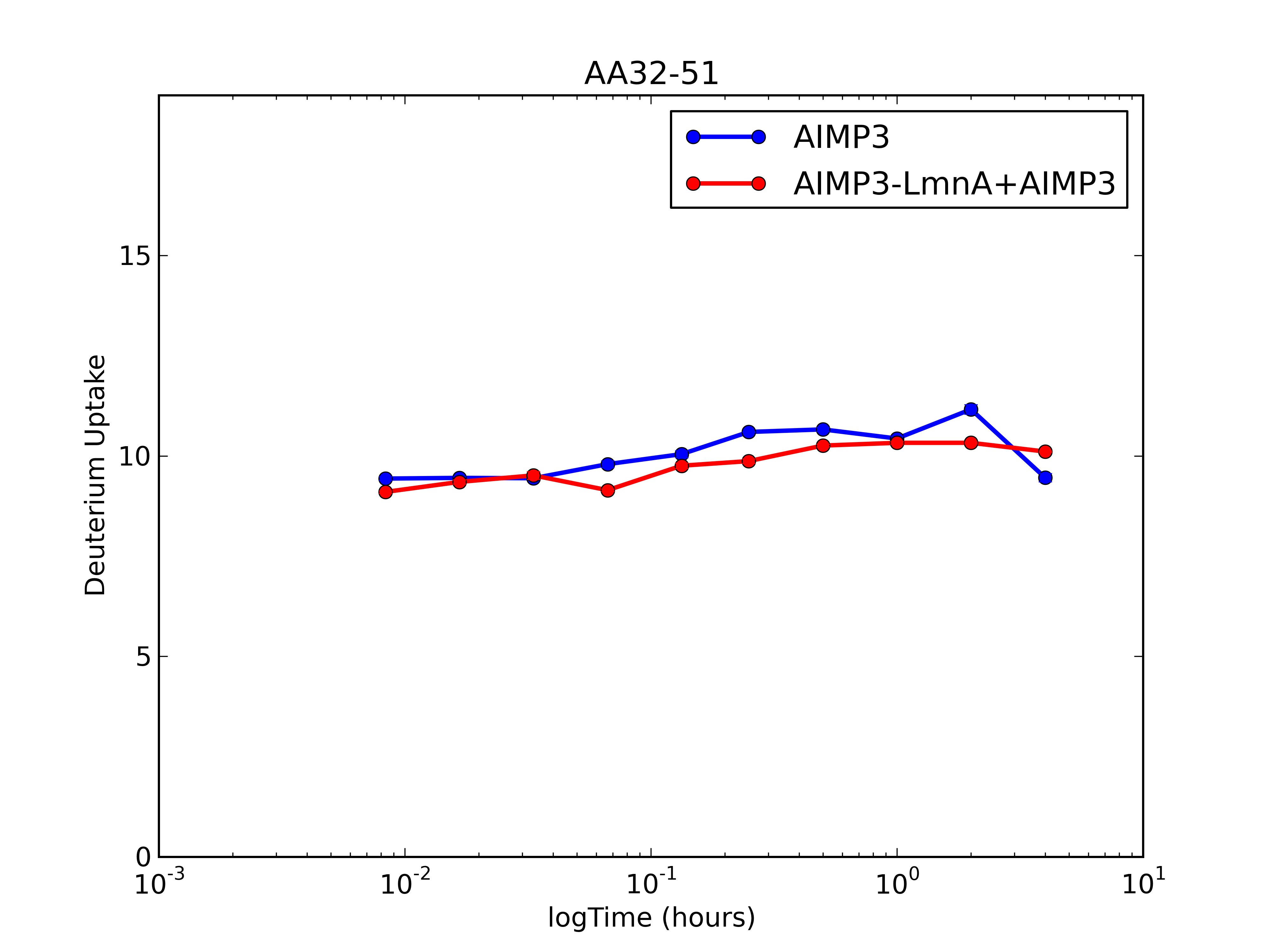

Supplement: S1 File — (ZIP) [file pone.0181869.s003.zip › logfigure-AIMP3-scale/AA32-51_charge_4_mz549.0.csv.csv.png]

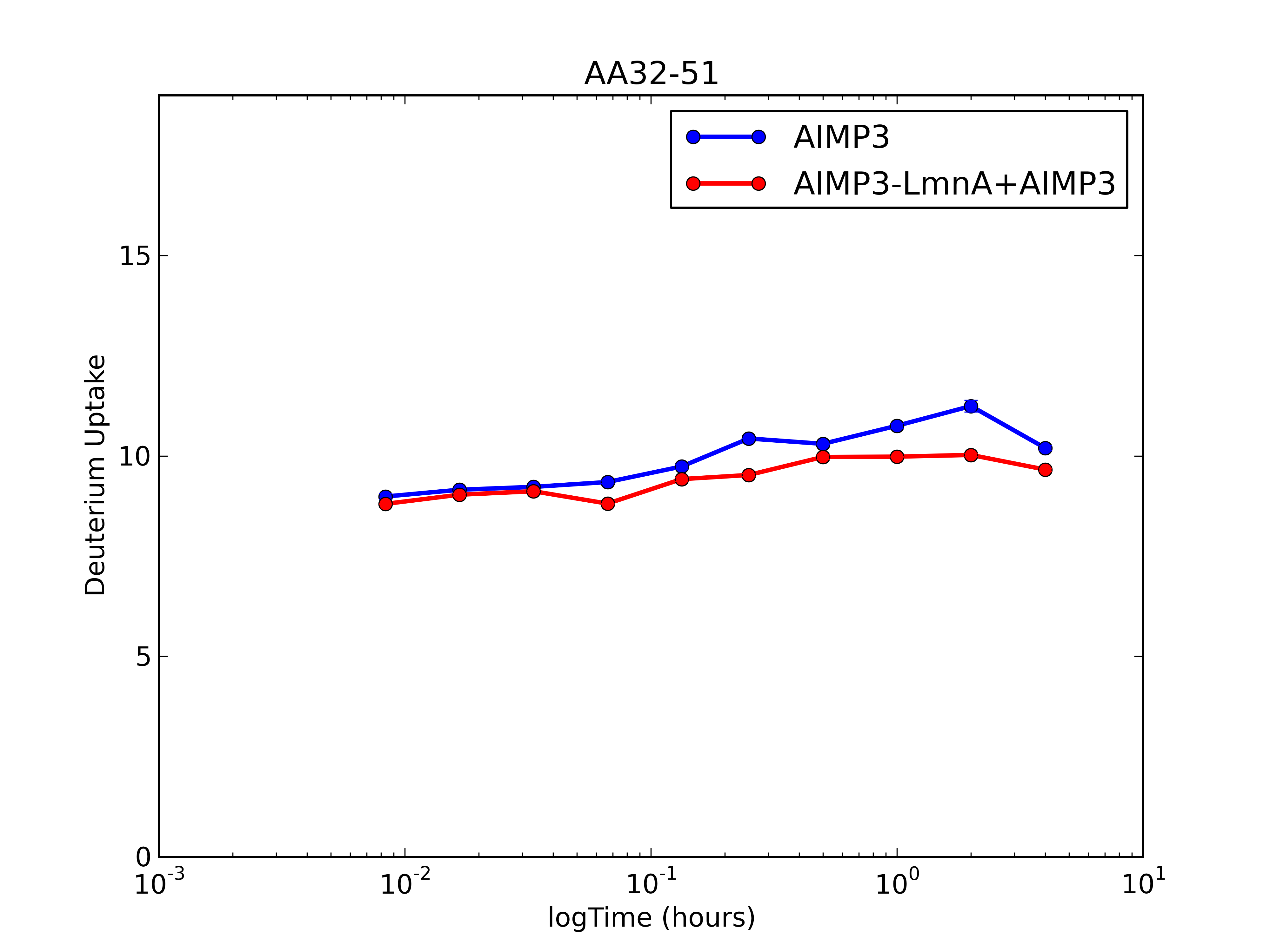

Supplement: S1 File — (ZIP) [file pone.0181869.s003.zip › logfigure-AIMP3-scale/AA32-51_charge_5_mz439.4.csv.csv.png]

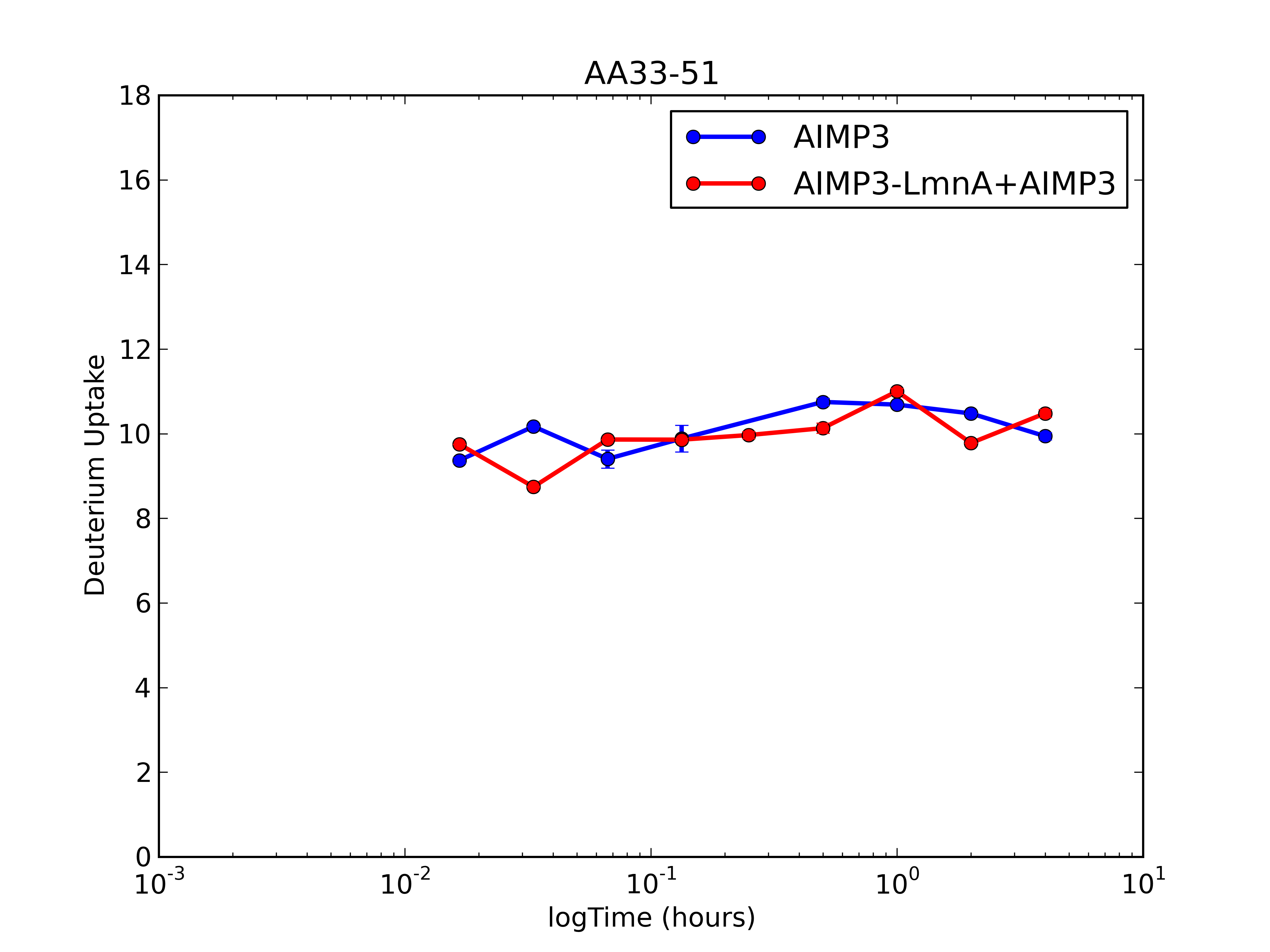

Supplement: S1 File — (ZIP) [file pone.0181869.s003.zip › logfigure-AIMP3-scale/AA33-51_charge_3_mz694.0.csv.csv.png]

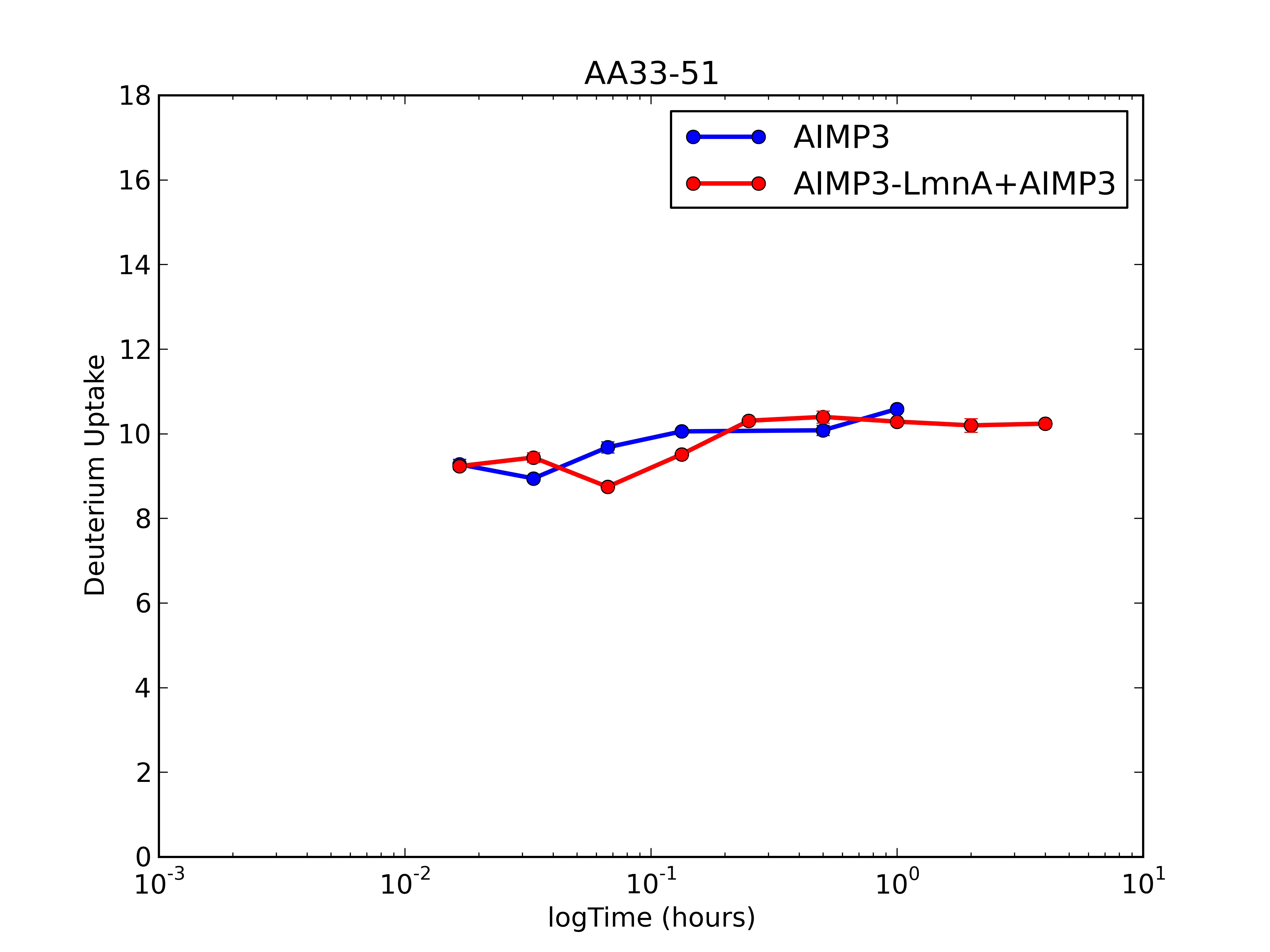

Supplement: S1 File — (ZIP) [file pone.0181869.s003.zip › logfigure-AIMP3-scale/AA33-51_charge_4_mz520.7.csv.csv.png]

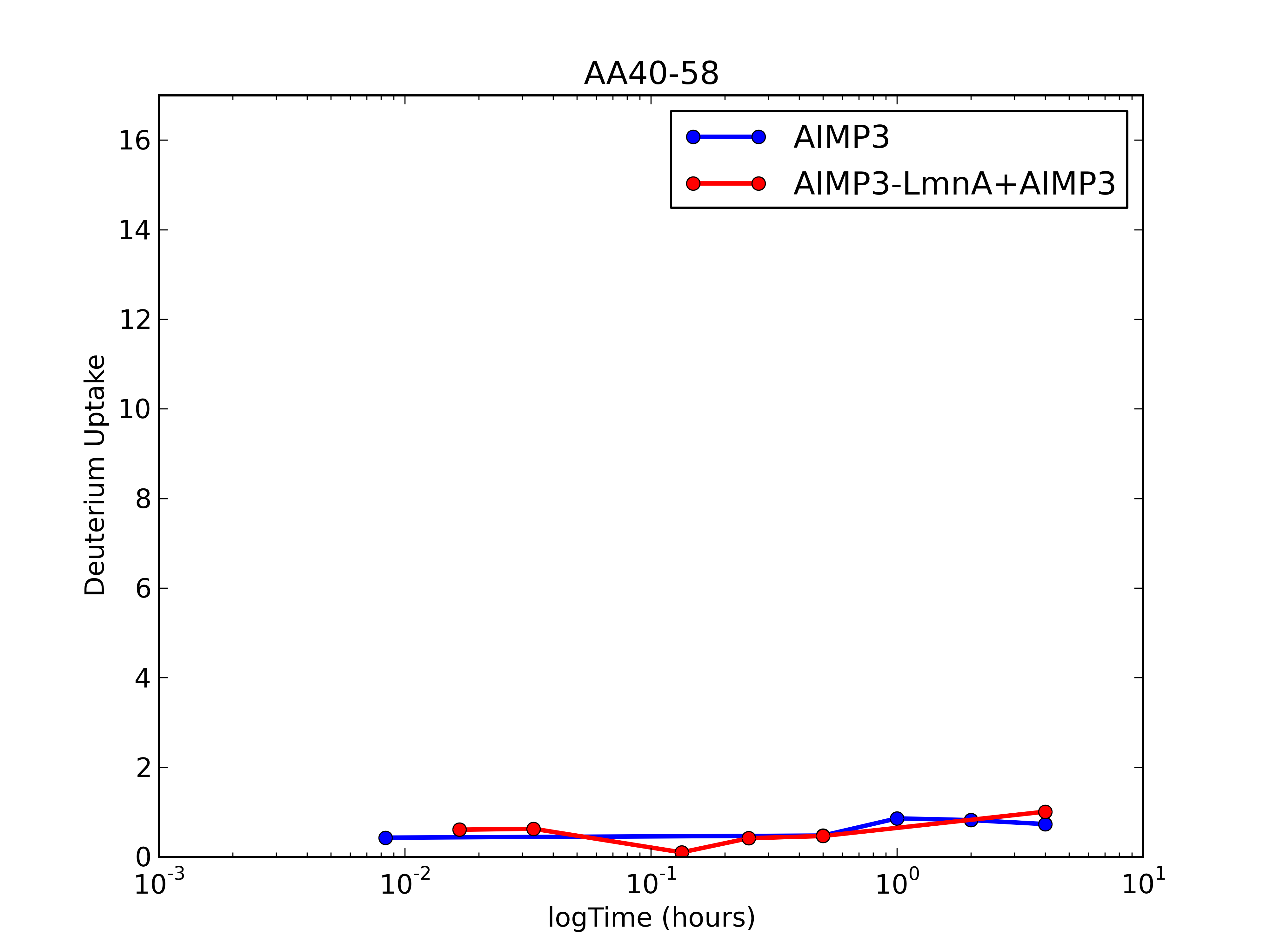

Supplement: S1 File — (ZIP) [file pone.0181869.s003.zip › logfigure-AIMP3-scale/AA40-58_charge_5_mz427.0.csv.csv.png]

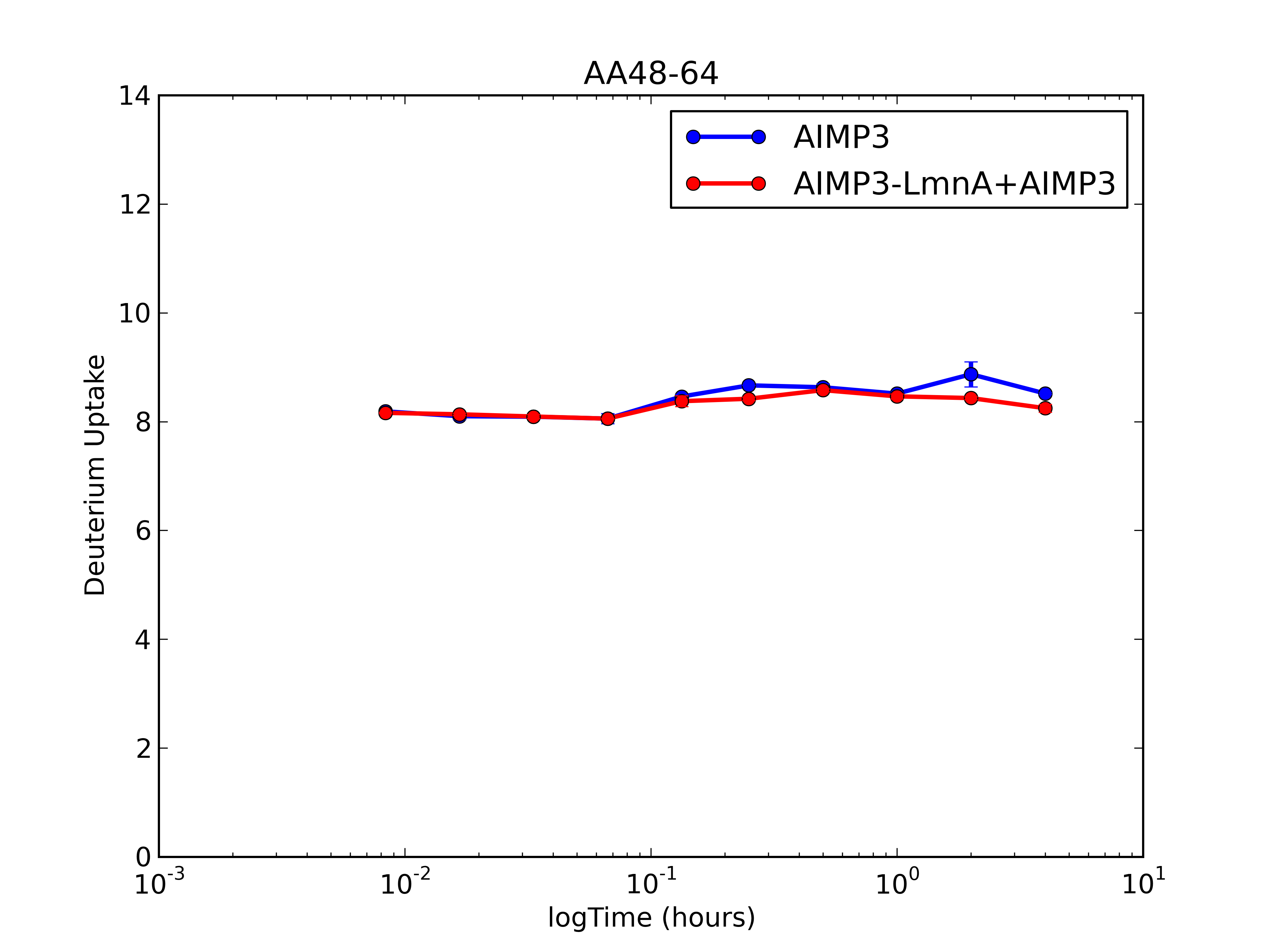

Supplement: S1 File — (ZIP) [file pone.0181869.s003.zip › logfigure-AIMP3-scale/AA48-64_charge_3_mz608.6.csv.csv.png]

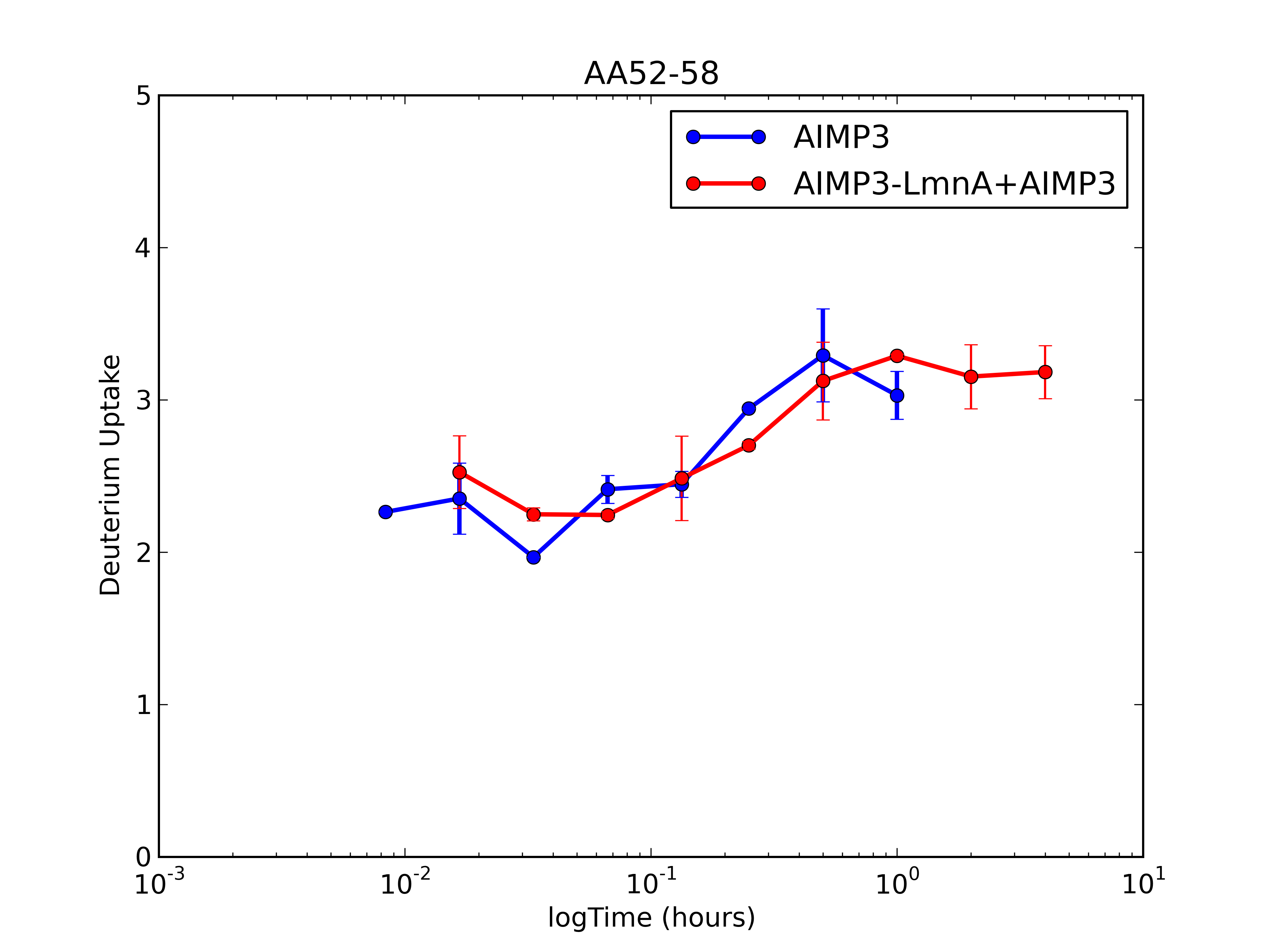

Supplement: S1 File — (ZIP) [file pone.0181869.s003.zip › logfigure-AIMP3-scale/AA52-58_charge_1_mz784.4.csv.csv.png]

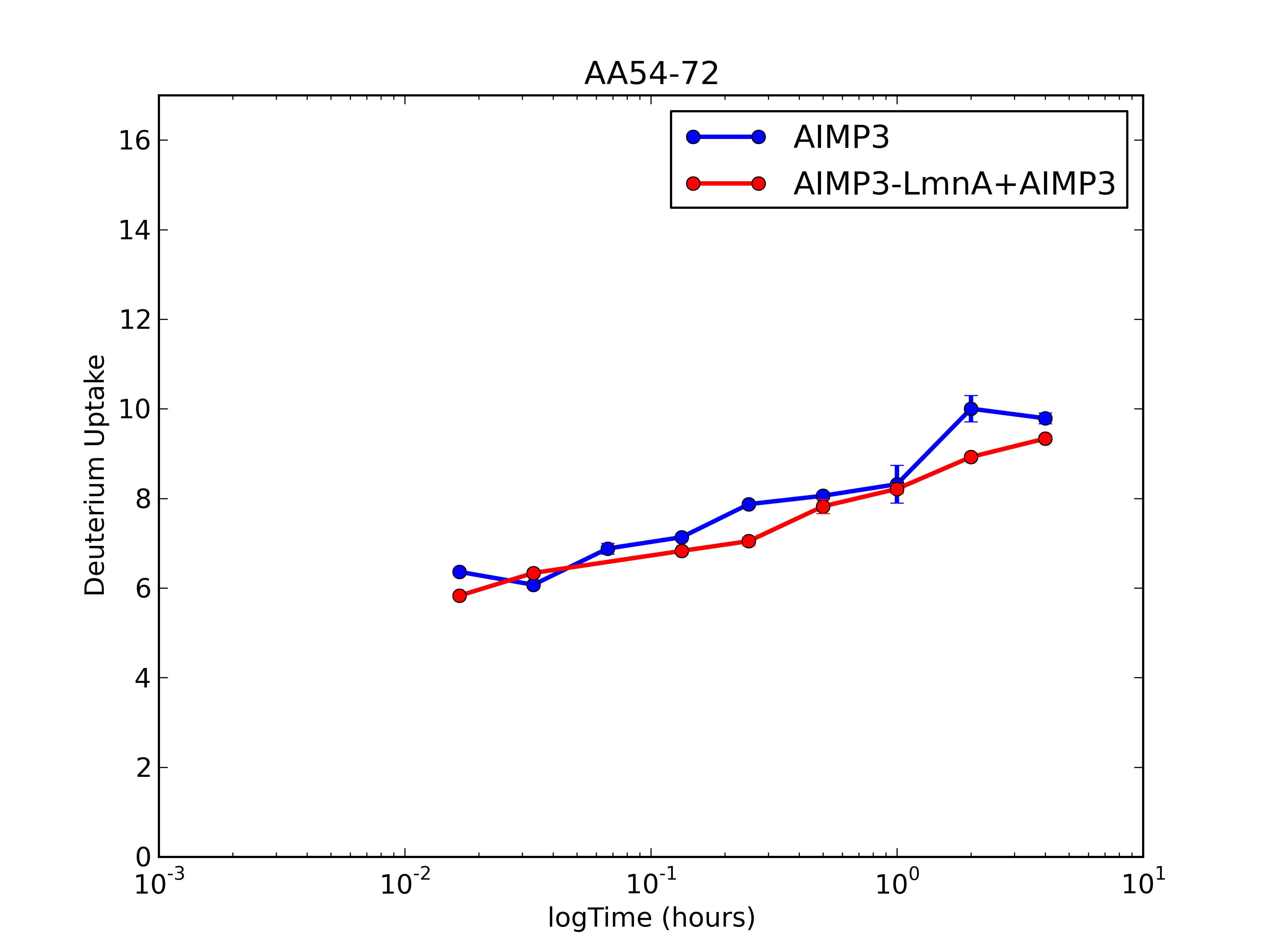

Supplement: S1 File — (ZIP) [file pone.0181869.s003.zip › logfigure-AIMP3-scale/AA54-72_charge_3_mz636.6.csv.csv.png]

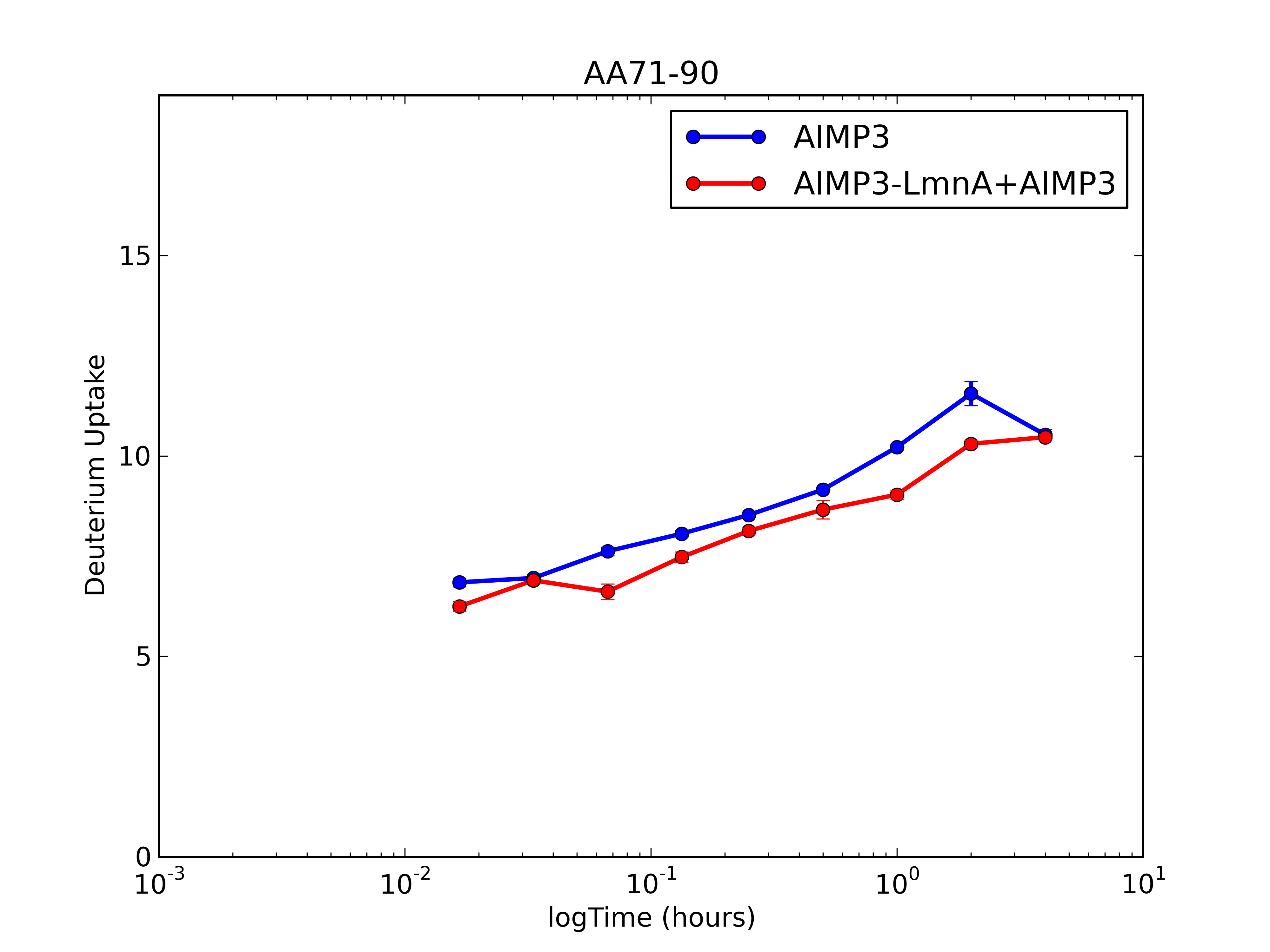

Supplement: S1 File — (ZIP) [file pone.0181869.s003.zip › logfigure-AIMP3-scale/AA71-90_charge_3_mz743.7.csv.csv.png]

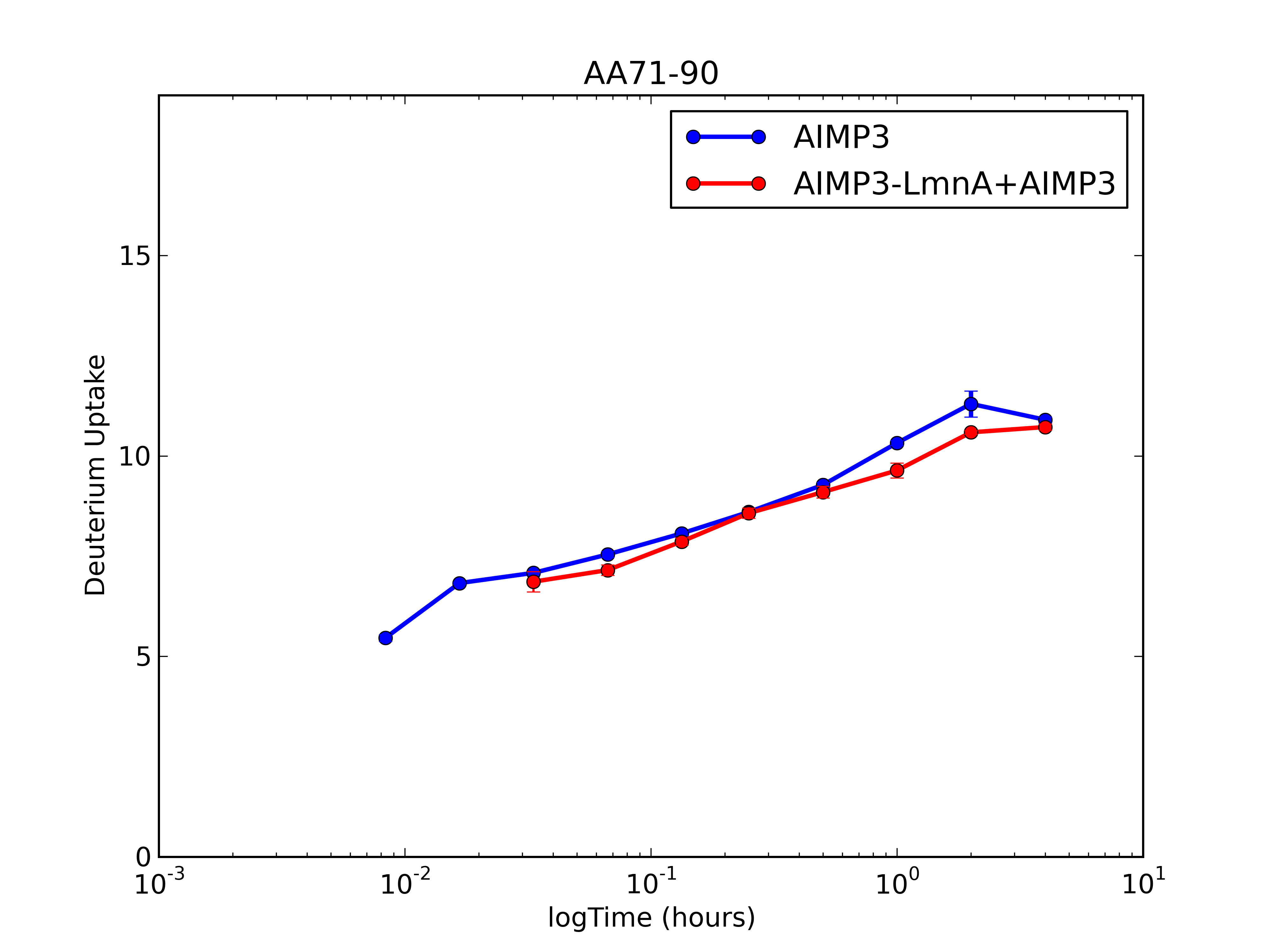

Supplement: S1 File — (ZIP) [file pone.0181869.s003.zip › logfigure-AIMP3-scale/AA71-90_charge_4_mz558.0.csv.csv.png]

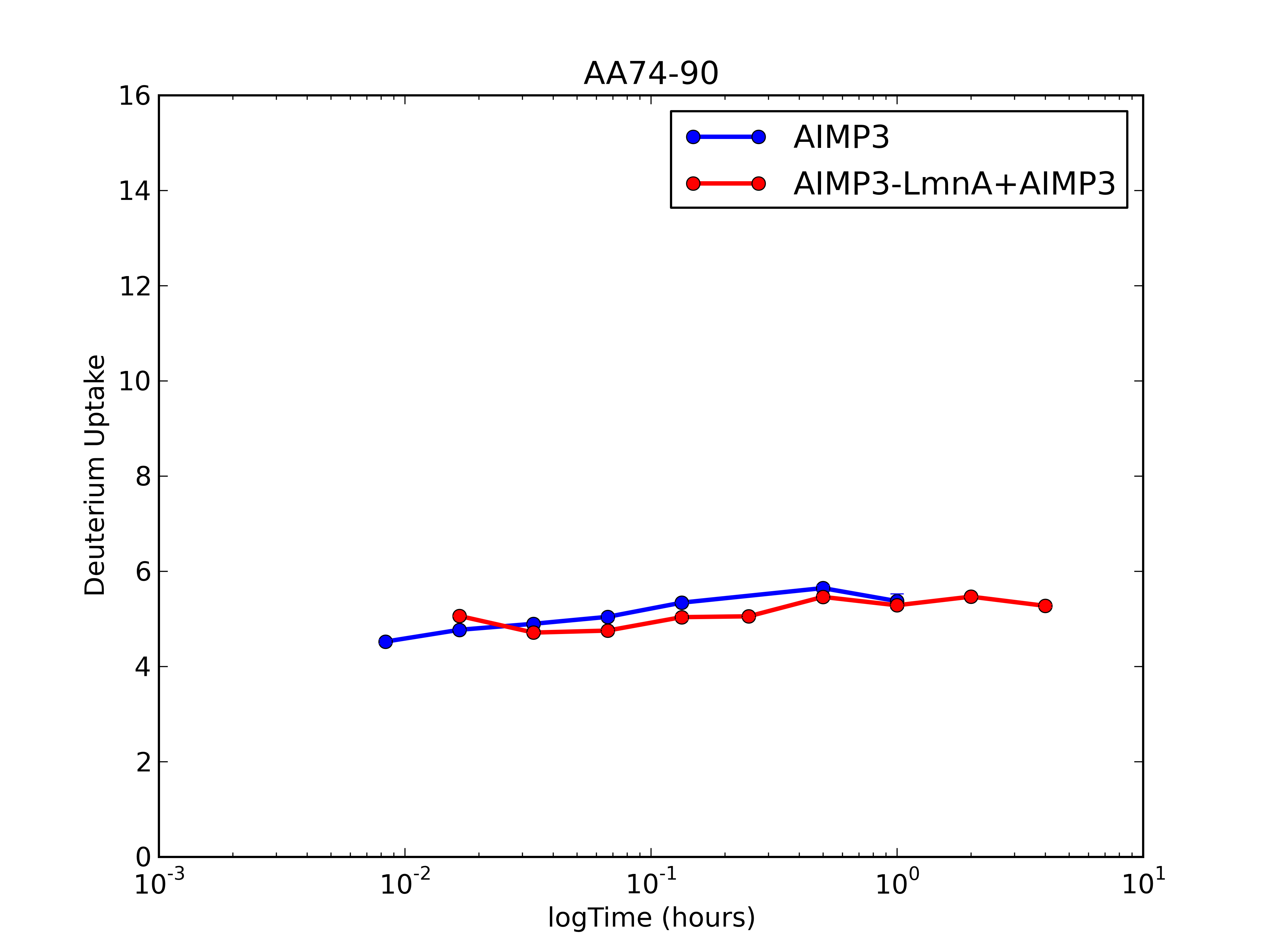

Supplement: S1 File — (ZIP) [file pone.0181869.s003.zip › logfigure-AIMP3-scale/AA74-90_charge_4_mz477.7.csv.csv.png]

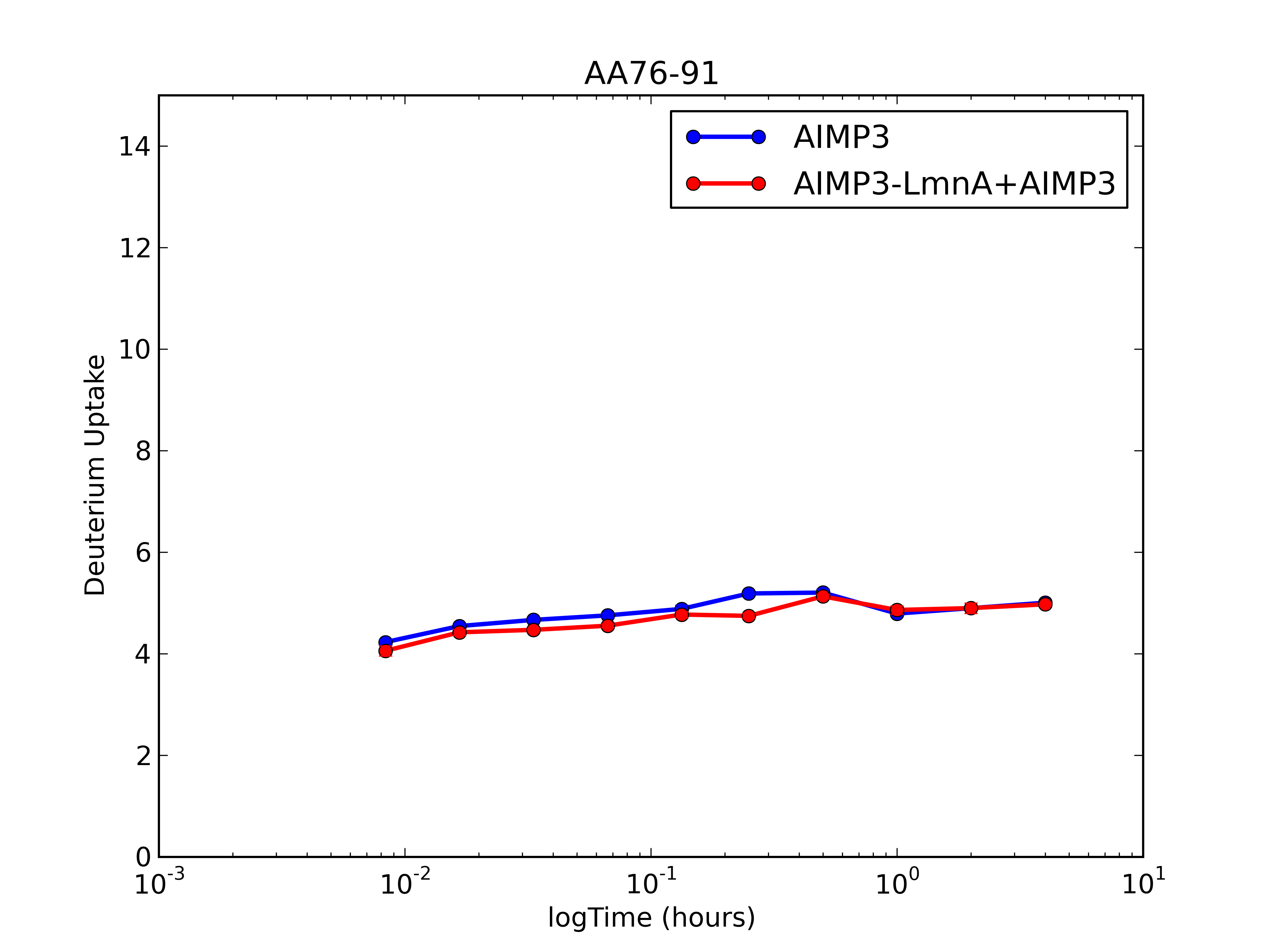

Supplement: S1 File — (ZIP) [file pone.0181869.s003.zip › logfigure-AIMP3-scale/AA76-91_charge_3_mz584.6.csv.csv.png]

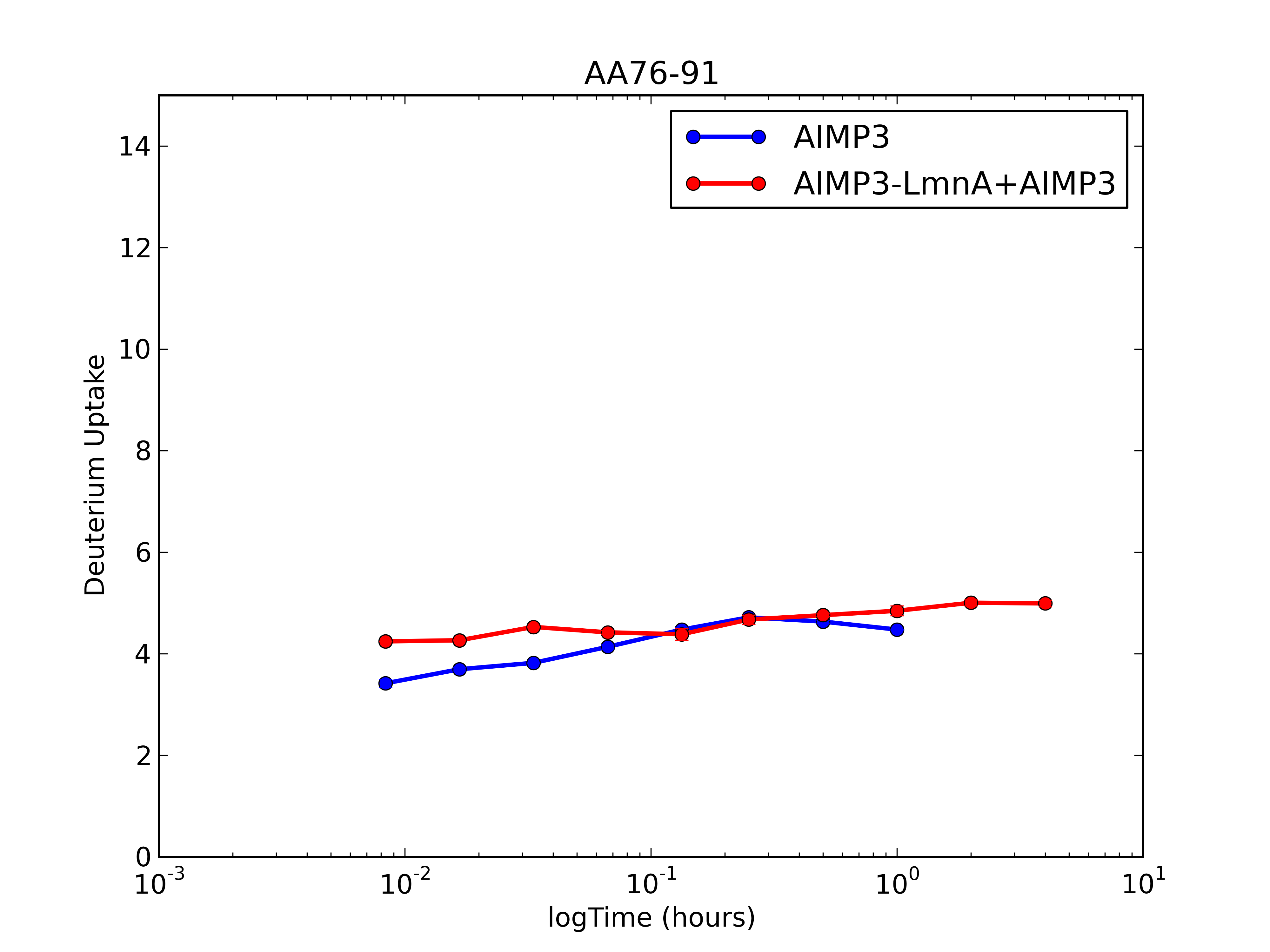

Supplement: S1 File — (ZIP) [file pone.0181869.s003.zip › logfigure-AIMP3-scale/AA76-91_charge_4_mz438.7.csv.csv.png]

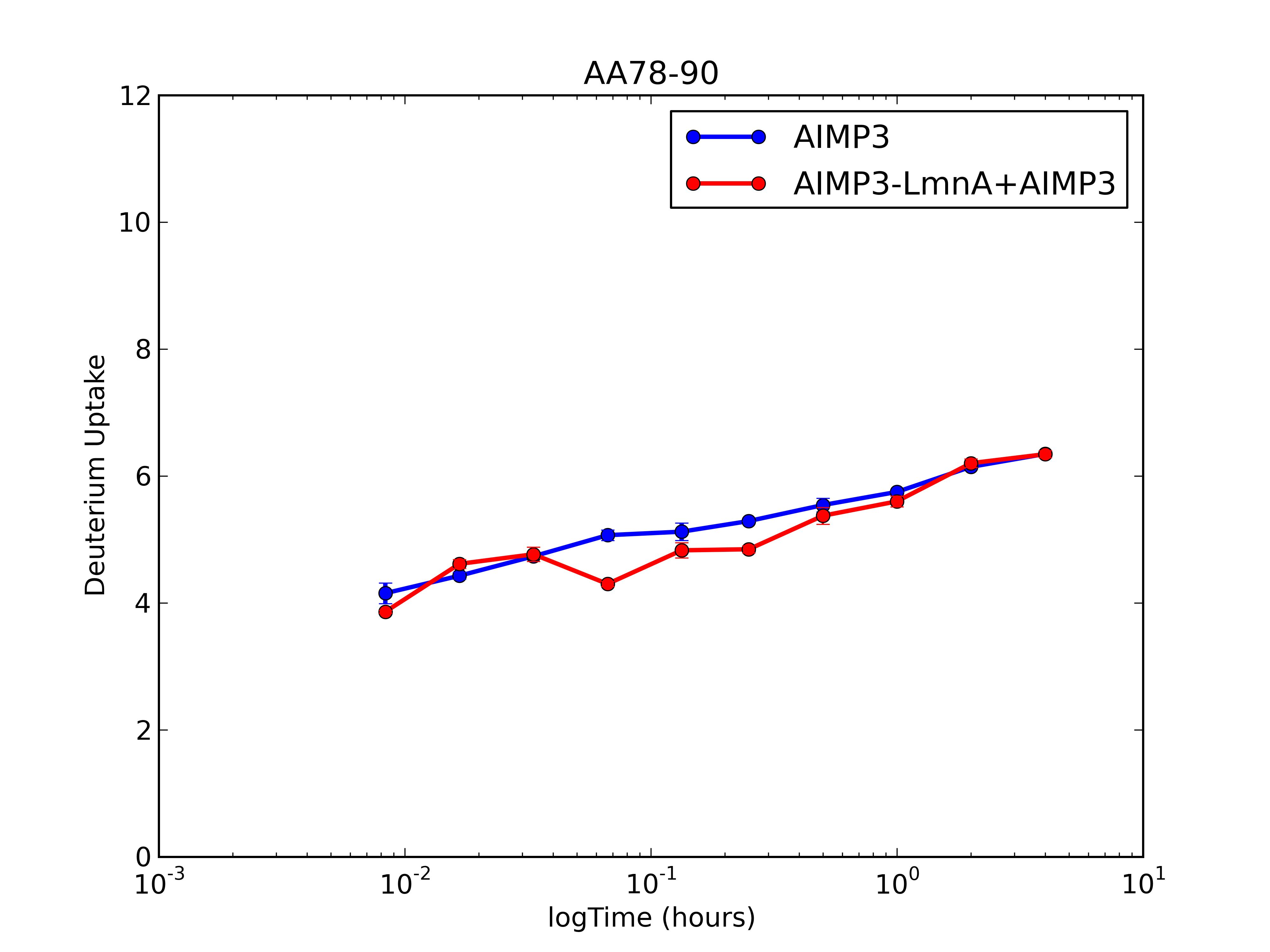

Supplement: S1 File — (ZIP) [file pone.0181869.s003.zip › logfigure-AIMP3-scale/AA78-90_charge_2_mz741.3.csv.csv.png]

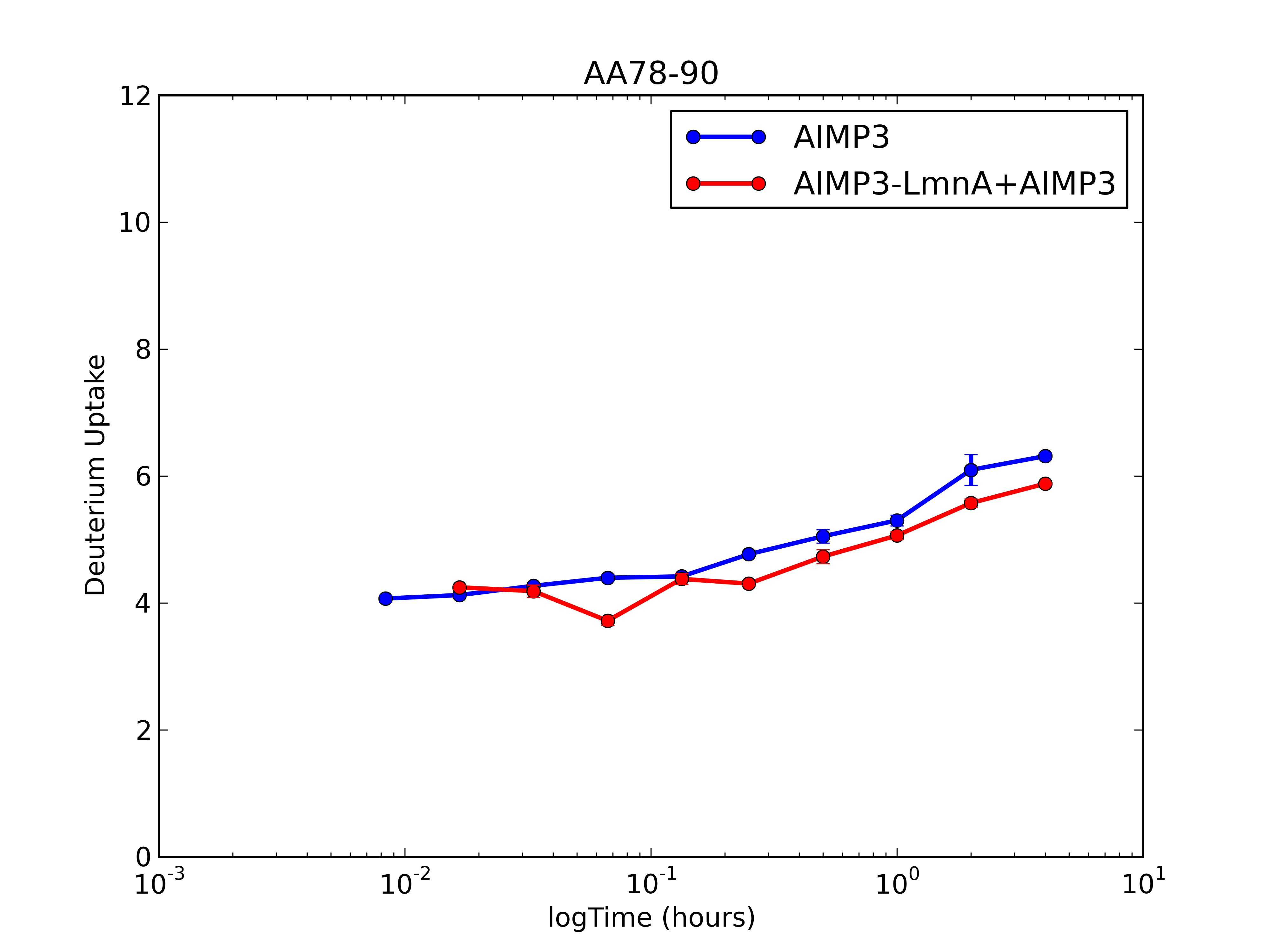

Supplement: S1 File — (ZIP) [file pone.0181869.s003.zip › logfigure-AIMP3-scale/AA78-90_charge_3_mz494.5.csv.csv.png]

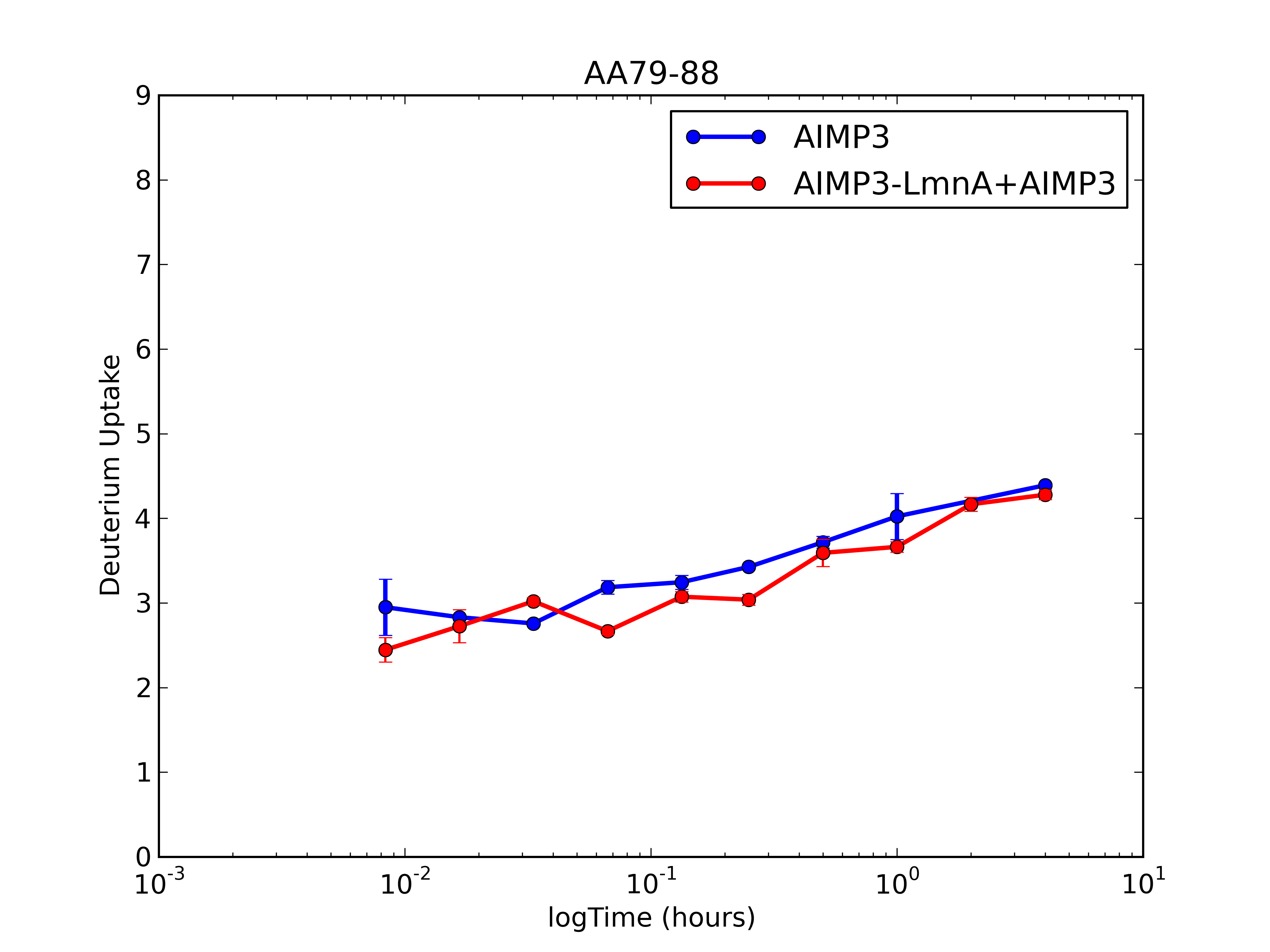

Supplement: S1 File — (ZIP) [file pone.0181869.s003.zip › logfigure-AIMP3-scale/AA79-88_charge_2_mz555.7.csv.csv.png]

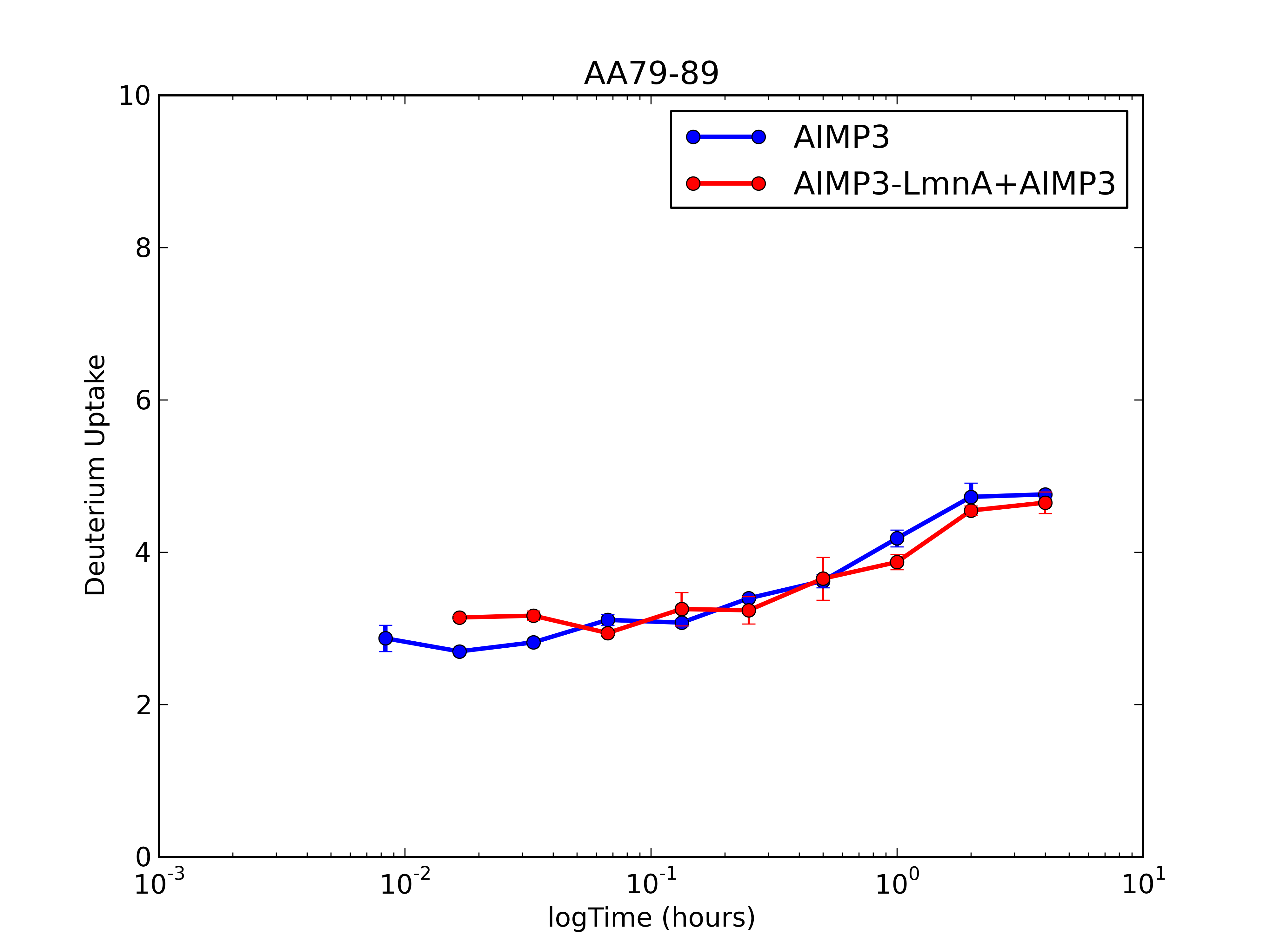

Supplement: S1 File — (ZIP) [file pone.0181869.s003.zip › logfigure-AIMP3-scale/AA79-89_charge_2_mz620.3.csv.csv.png]

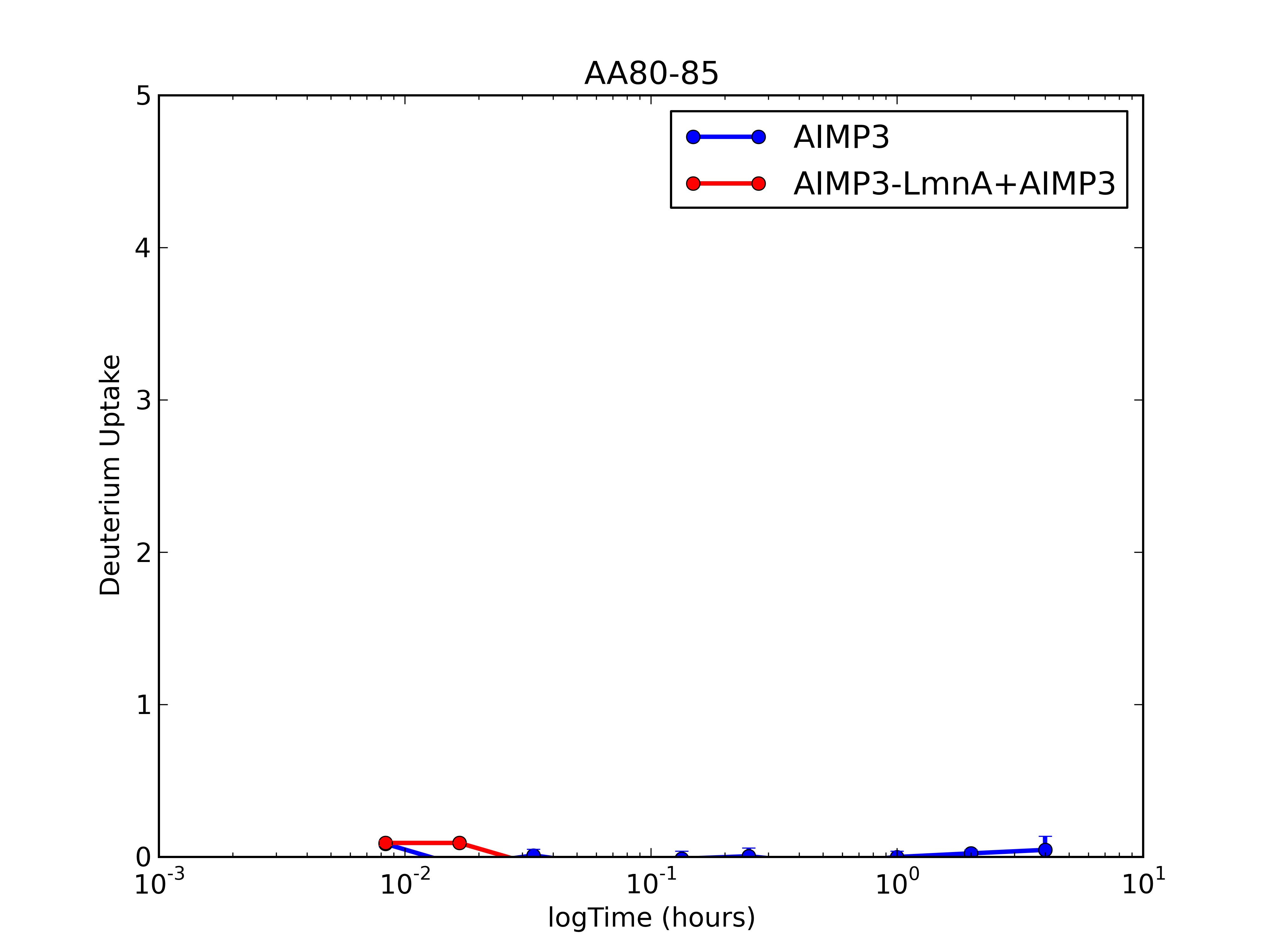

Supplement: S1 File — (ZIP) [file pone.0181869.s003.zip › logfigure-AIMP3-scale/AA80-85_charge_1_mz681.3.csv.csv.png]

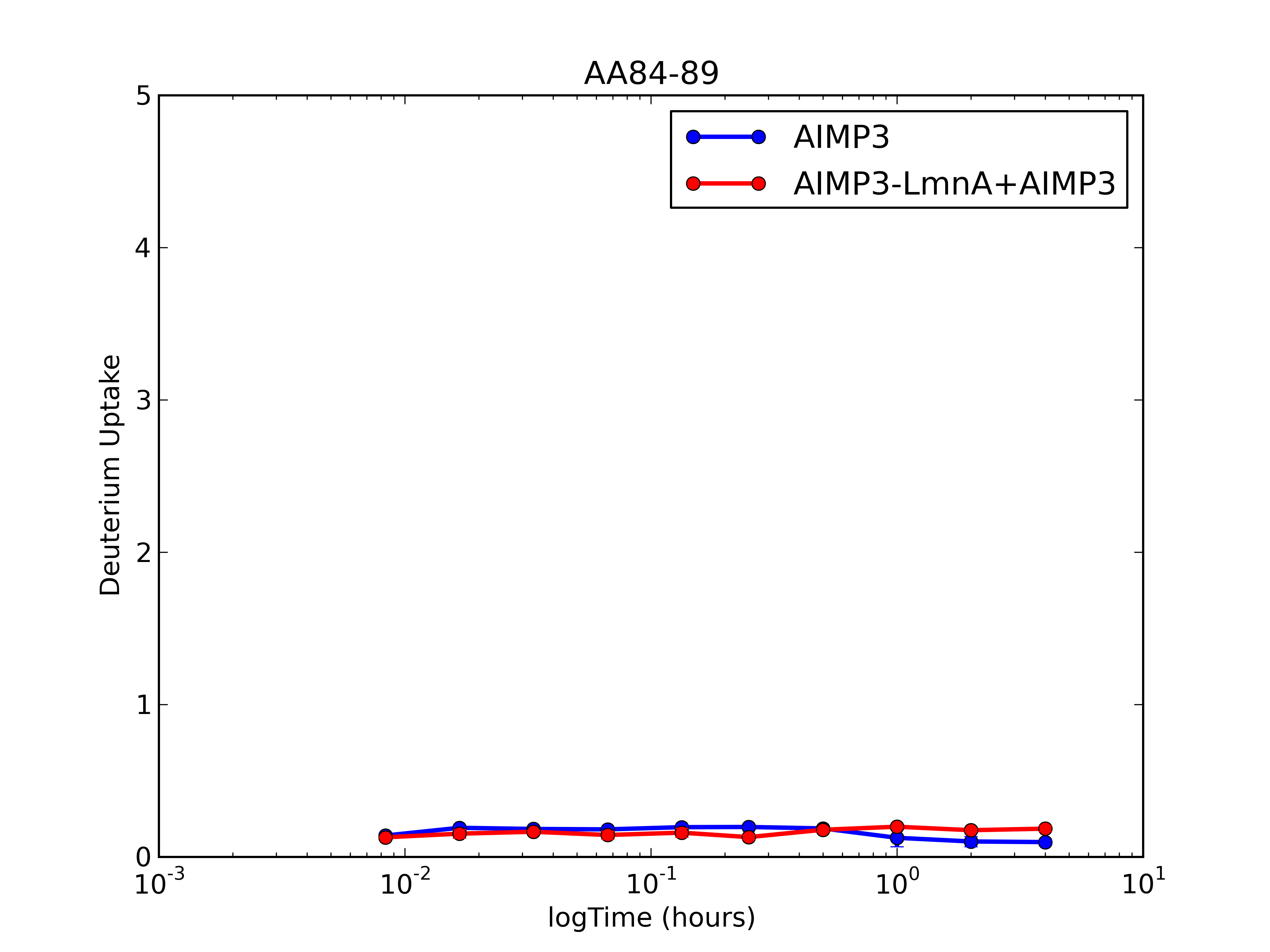

Supplement: S1 File — (ZIP) [file pone.0181869.s003.zip › logfigure-AIMP3-scale/AA84-89_charge_1_mz593.2.csv.csv.png]

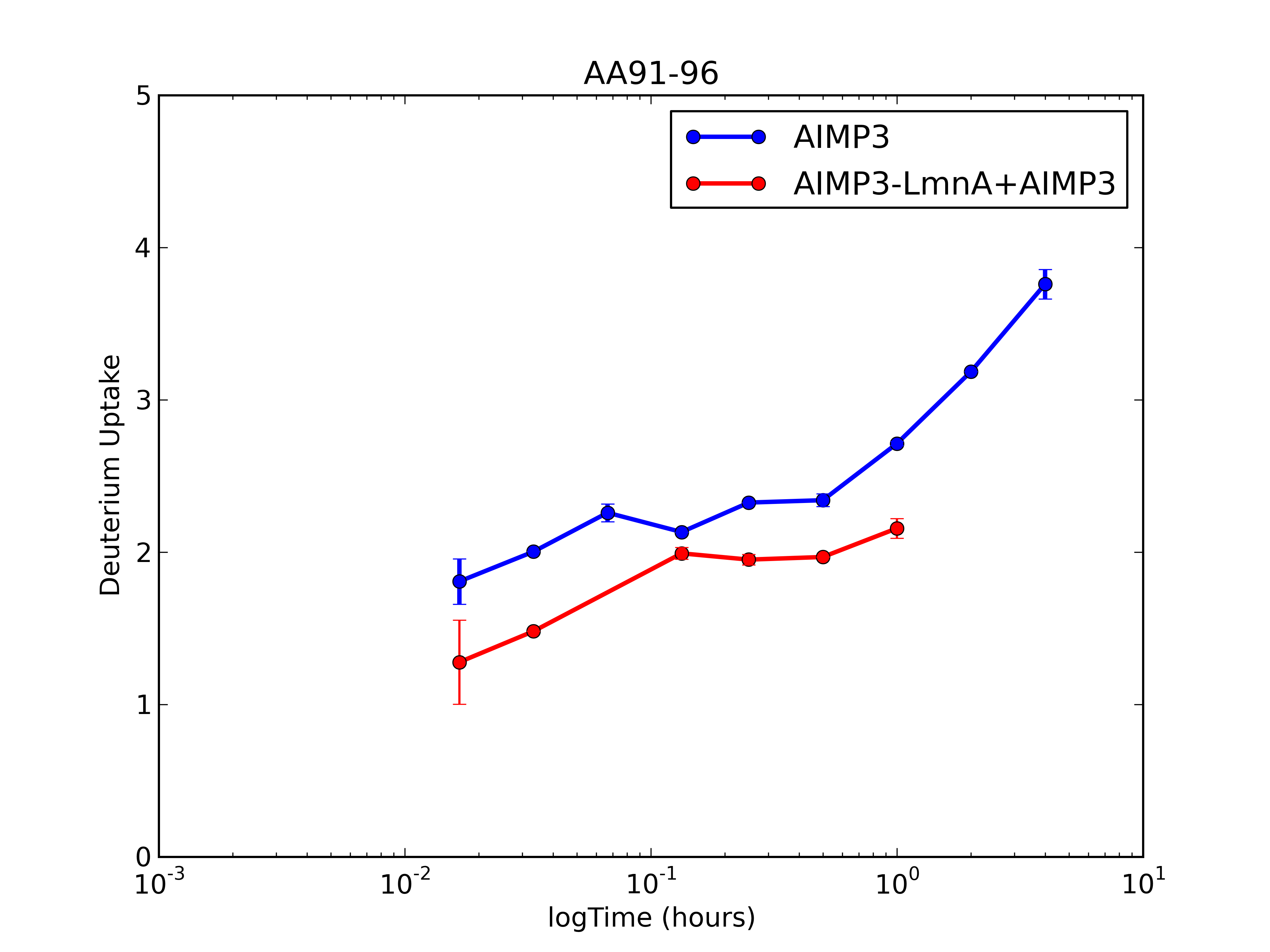

Supplement: S1 File — (ZIP) [file pone.0181869.s003.zip › logfigure-AIMP3-scale/AA91-96_charge_1_mz744.4.csv.csv.png]

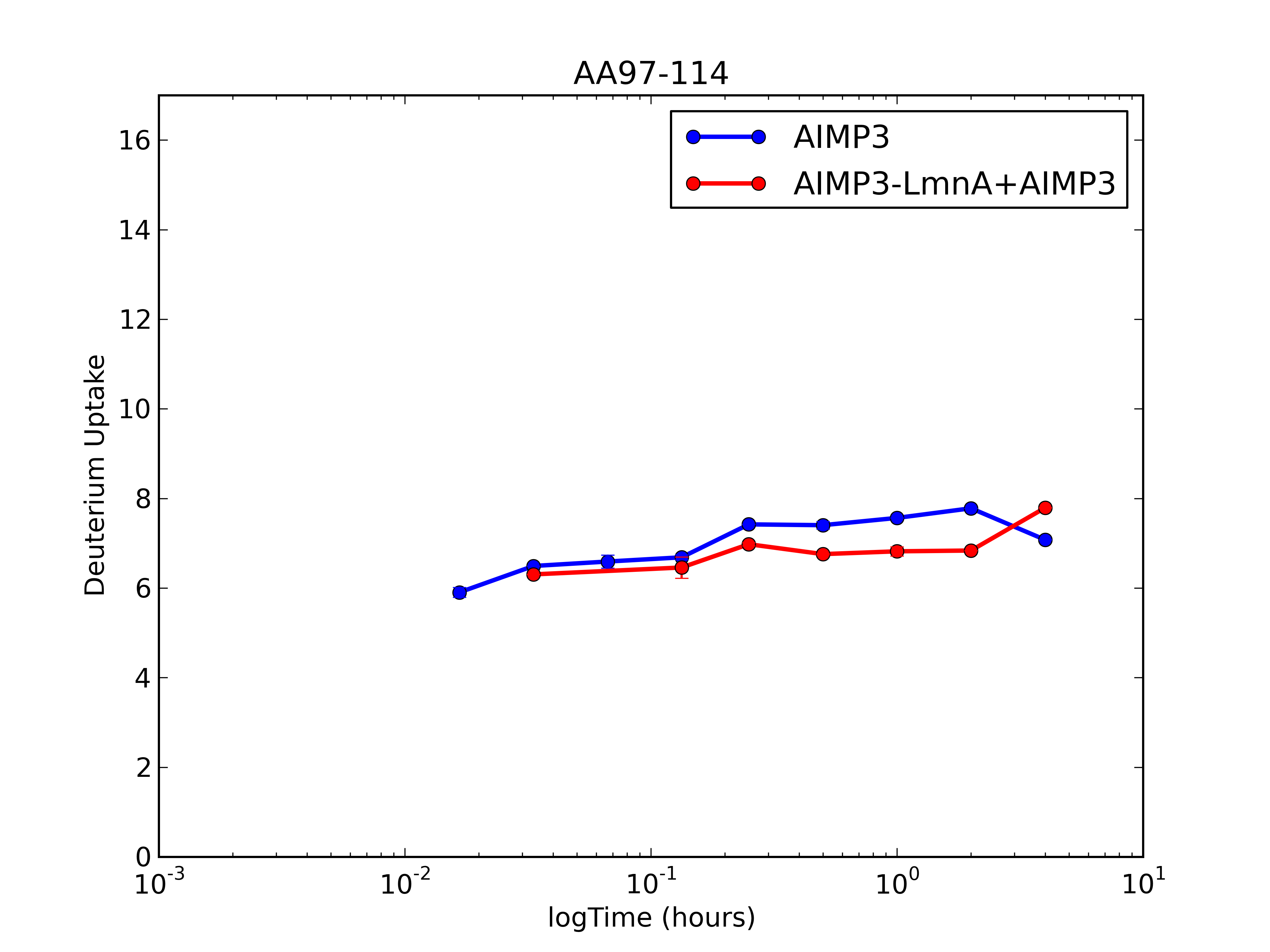

Supplement: S1 File — (ZIP) [file pone.0181869.s003.zip › logfigure-AIMP3-scale/AA97-114_charge_4_mz525.2.csv.csv.png]

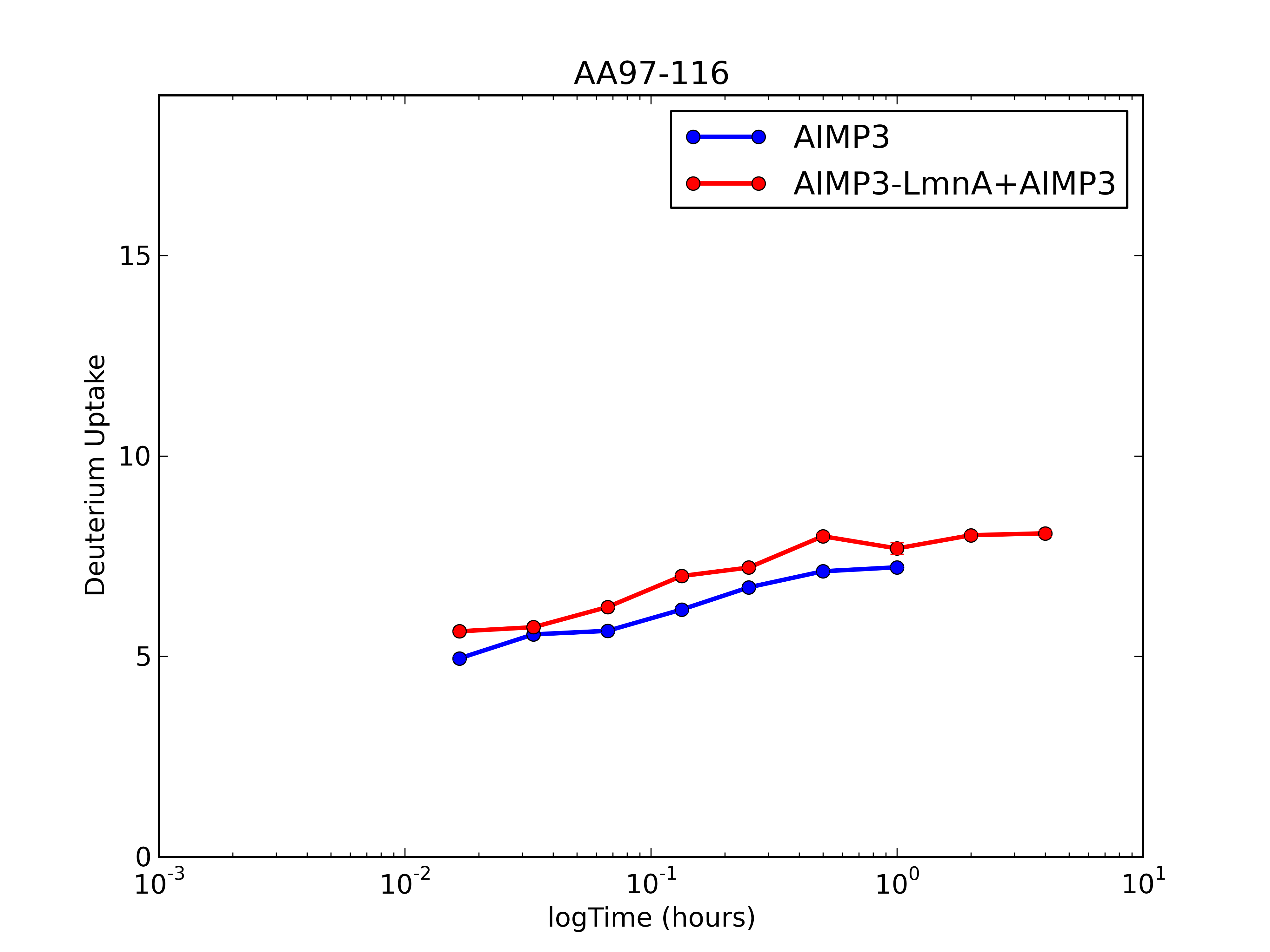

Supplement: S1 File — (ZIP) [file pone.0181869.s003.zip › logfigure-AIMP3-scale/AA97-116_charge_4_mz578.7.csv.csv.png]

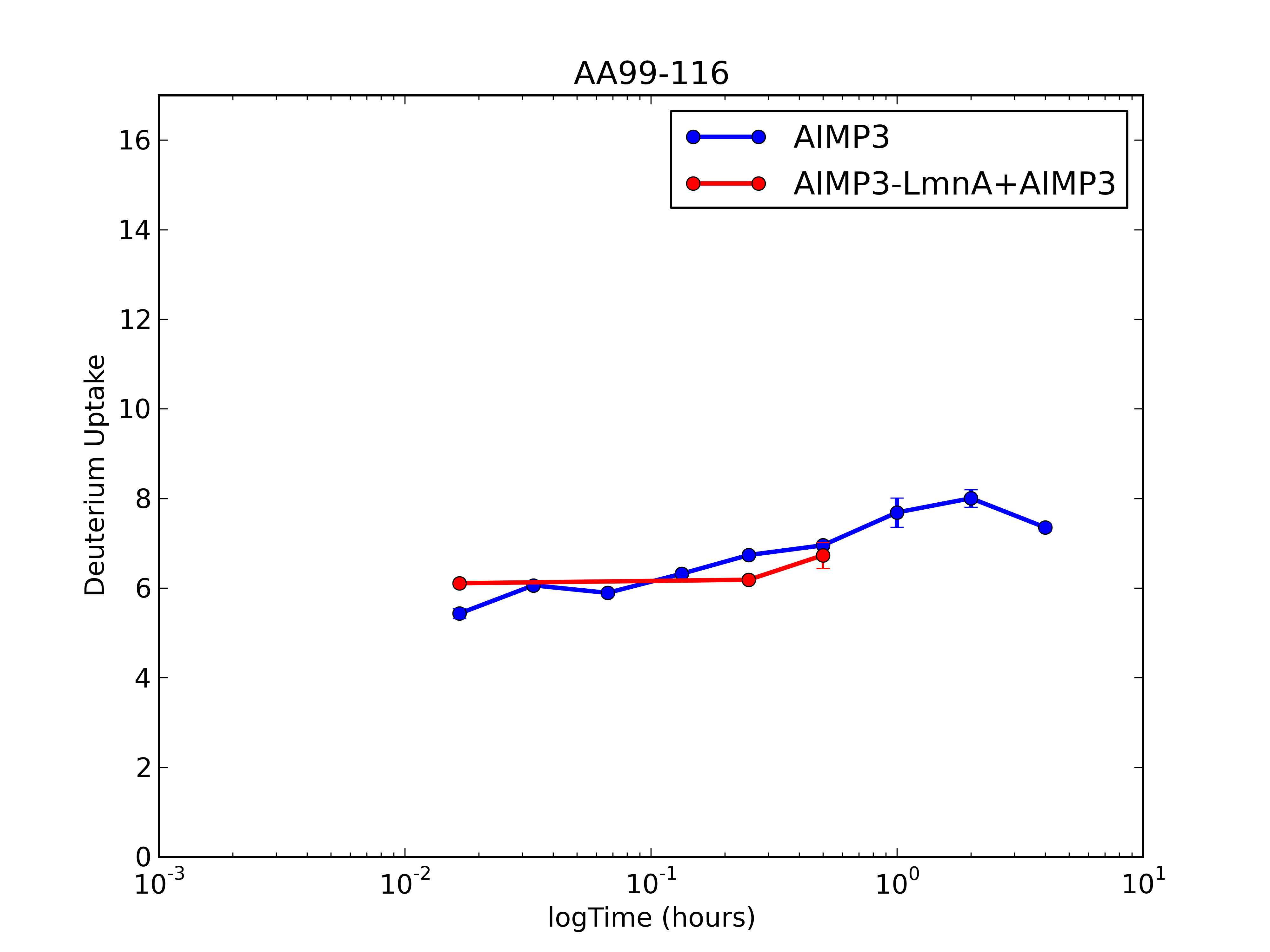

Supplement: S1 File — (ZIP) [file pone.0181869.s003.zip › logfigure-AIMP3-scale/AA99-116_charge_3_mz690.6.csv.csv.png]

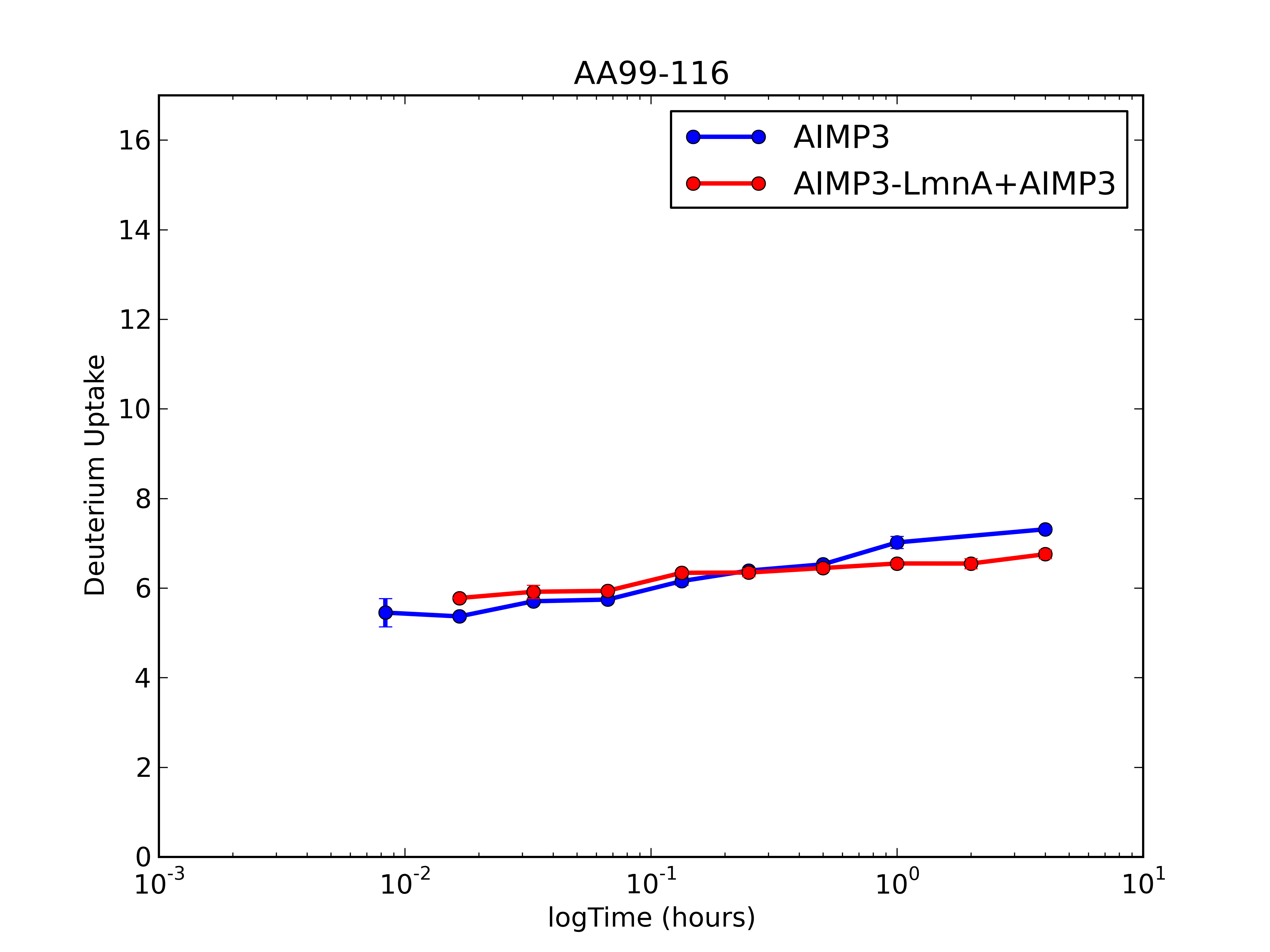

Supplement: S1 File — (ZIP) [file pone.0181869.s003.zip › logfigure-AIMP3-scale/AA99-116_charge_4_mz518.2.csv.csv.png]

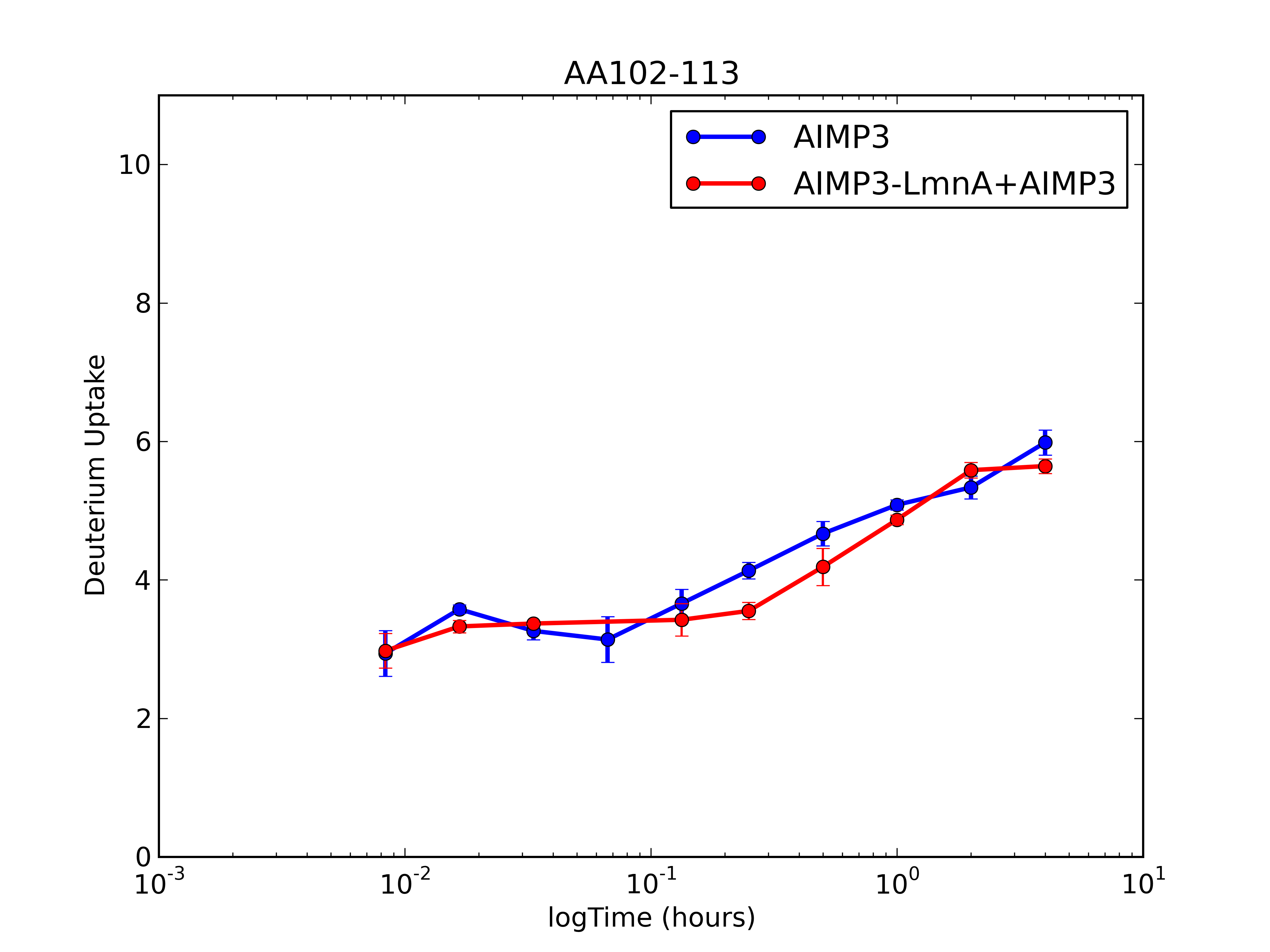

Supplement: S2 File — (ZIP) [file pone.0181869.s004.zip › logfigure-LmnA-scale/AA102-113_charge_2_mz610.8.csv.csv.png]

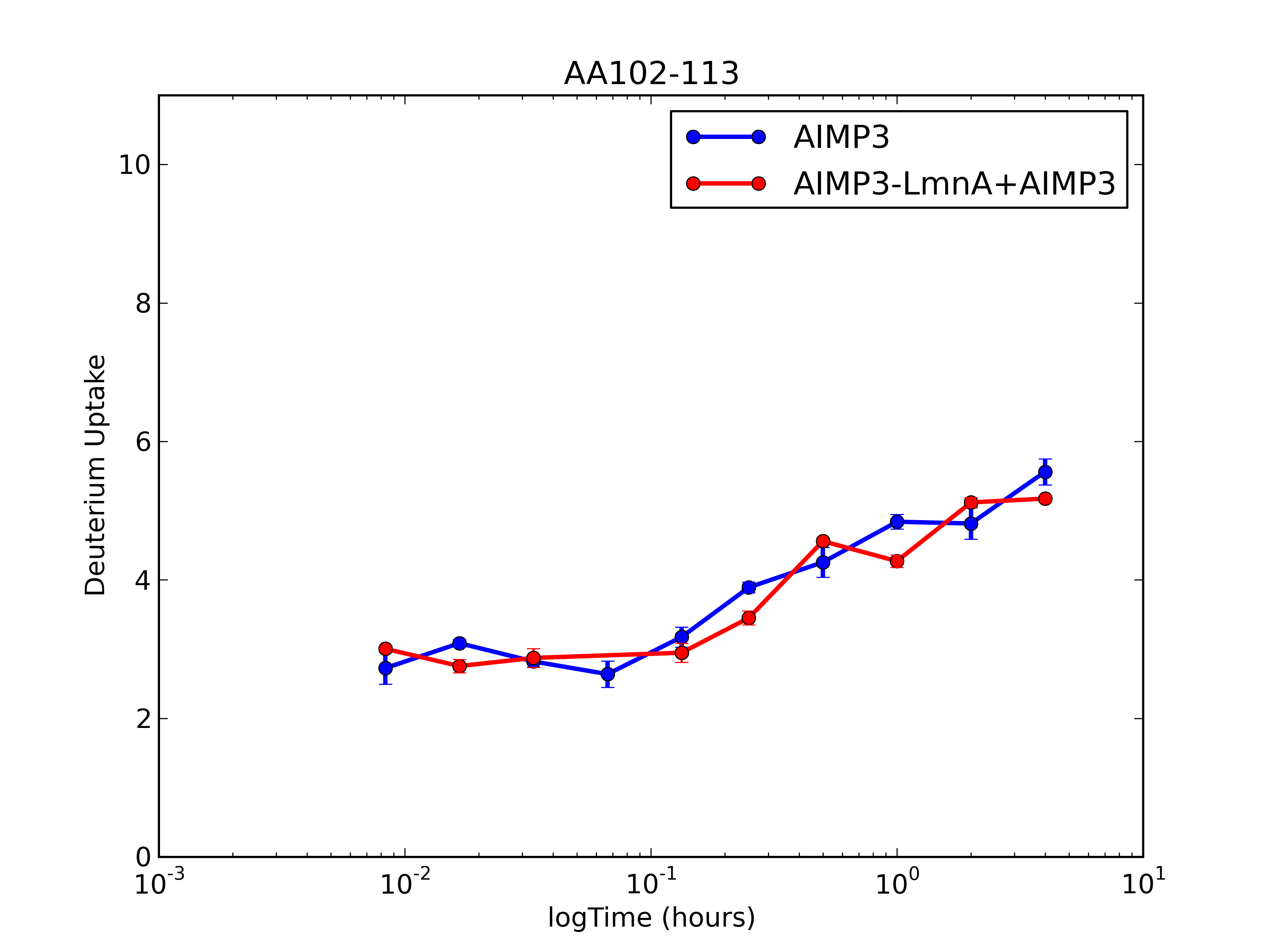

Supplement: S2 File — (ZIP) [file pone.0181869.s004.zip › logfigure-LmnA-scale/AA102-113_charge_3_mz407.5.csv.csv.png]

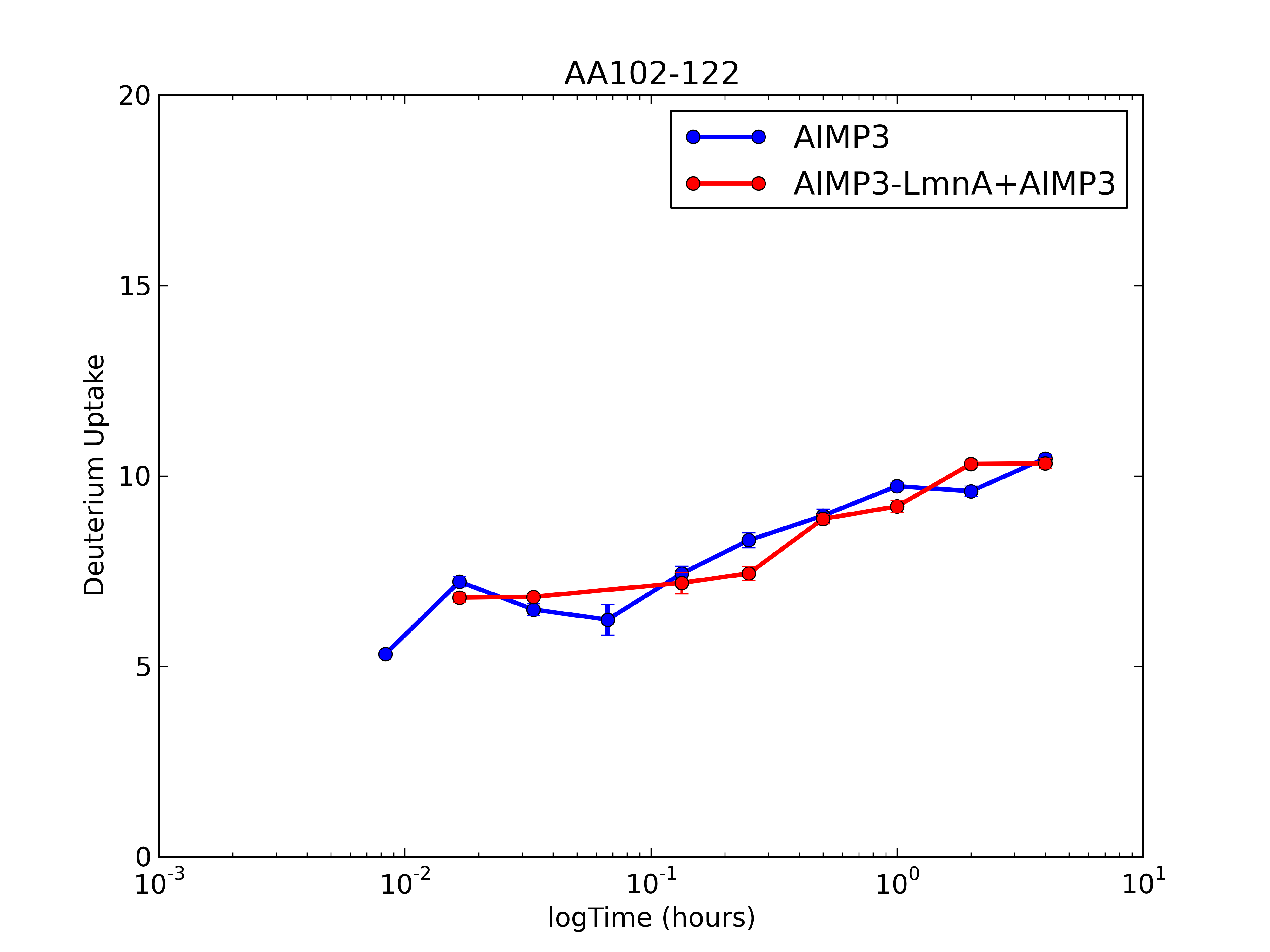

Supplement: S2 File — (ZIP) [file pone.0181869.s004.zip › logfigure-LmnA-scale/AA102-122_charge_3_mz725.7.csv.csv.png]

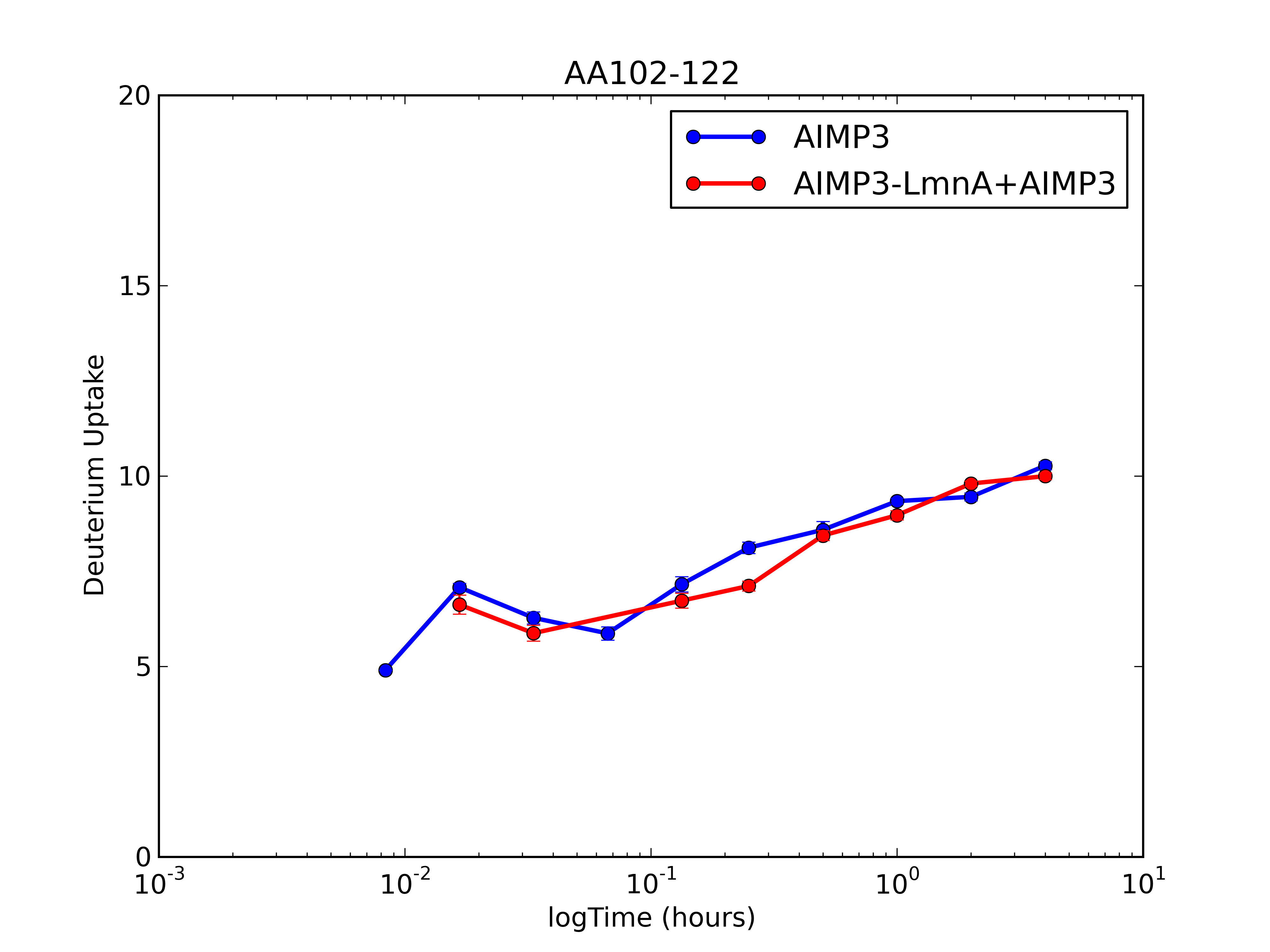

Supplement: S2 File — (ZIP) [file pone.0181869.s004.zip › logfigure-LmnA-scale/AA102-122_charge_4_mz544.5.csv.csv.png]
