# Supplementary figures and images for: Mapping the contact surfaces in the Lamin A:AIMP3 complex by hydrogen/deuterium exchange FT-ICR mass spectrometry (part 3 of 3)
Source: PLoS One. 2017 Aug 10;12(8):e0181869. doi: 10.1371/journal.pone.0181869 (PMC5552228; doi:10.1371/journal.pone.0181869)

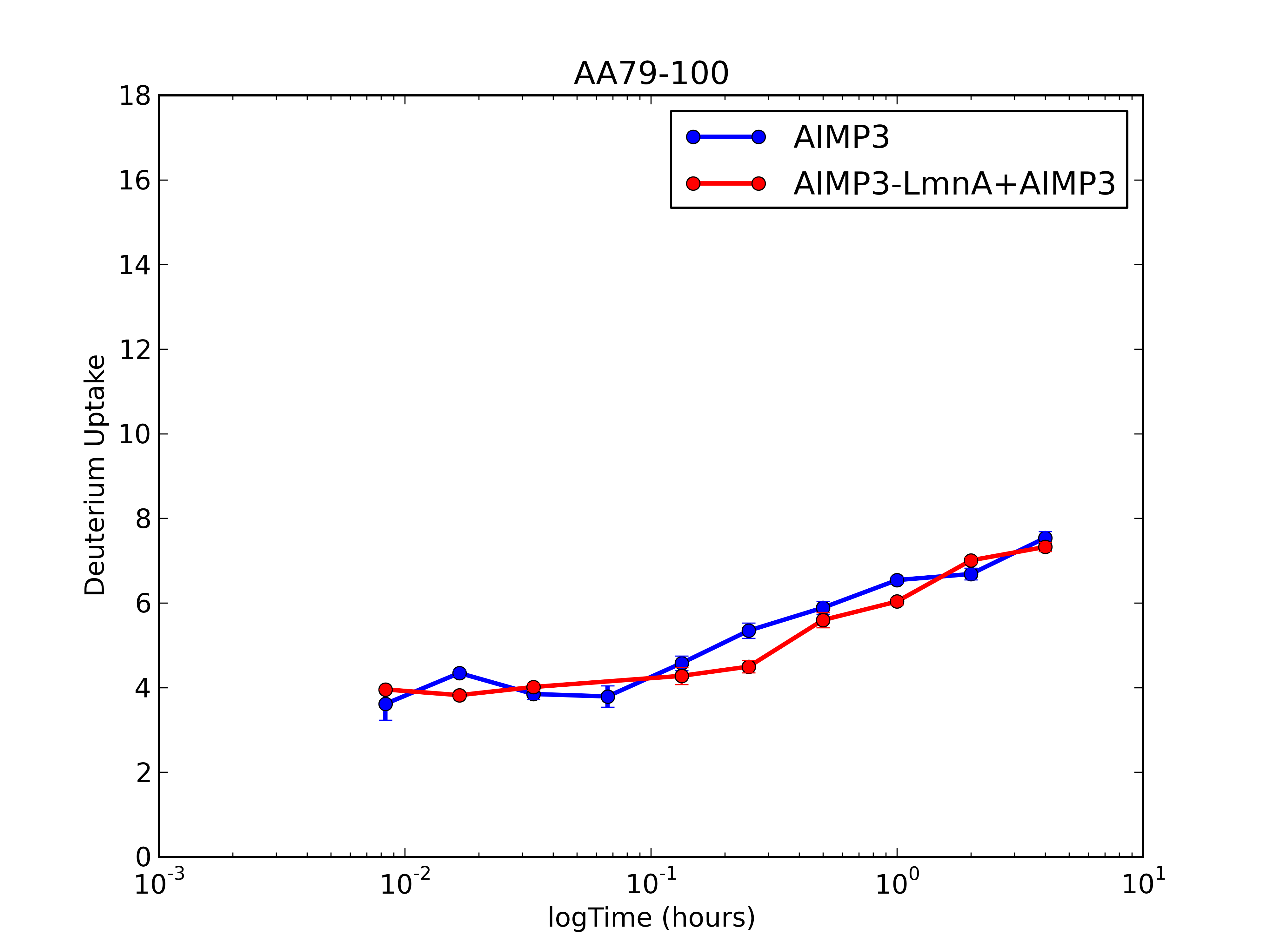

Supplement: S2 File — (ZIP) [file pone.0181869.s004.zip › logfigure-LmnA-scale/AA79-100_charge_3_mz784.4.csv.csv.png]

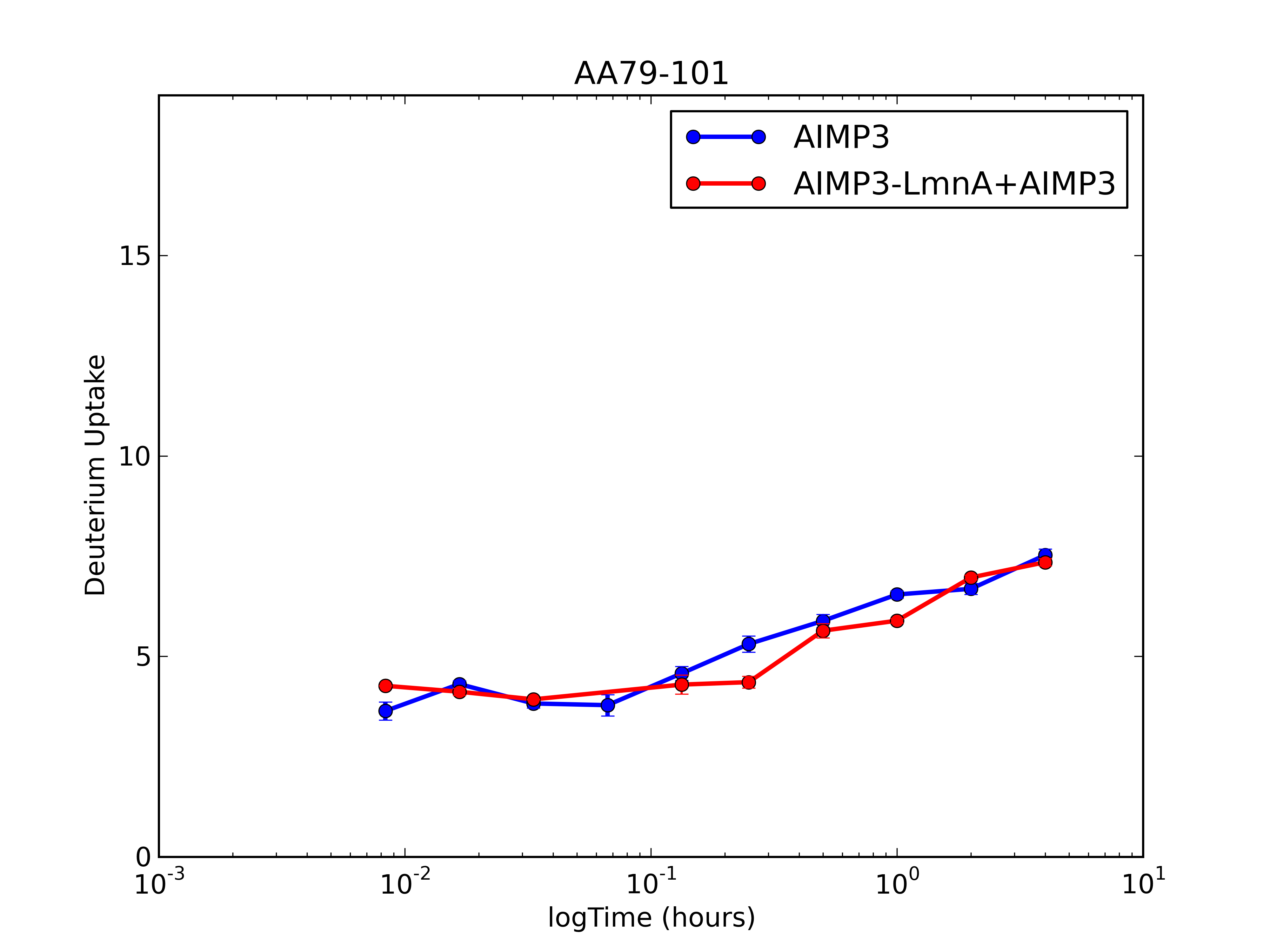

Supplement: S2 File — (ZIP) [file pone.0181869.s004.zip › logfigure-LmnA-scale/AA79-101_charge_3_mz822.1.csv.csv.png]

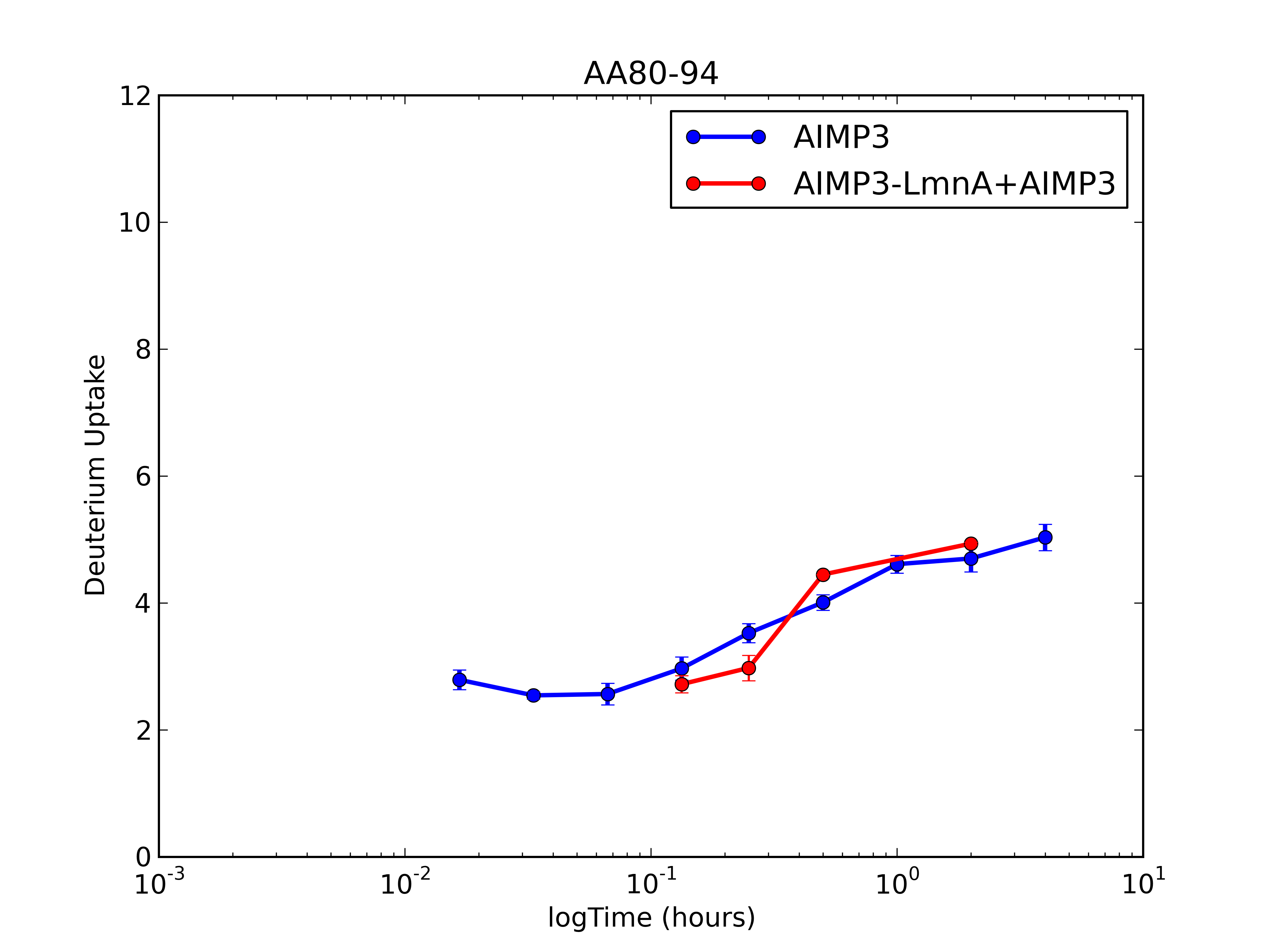

Supplement: S2 File — (ZIP) [file pone.0181869.s004.zip › logfigure-LmnA-scale/AA80-94_charge_3_mz548.6.csv.csv.png]

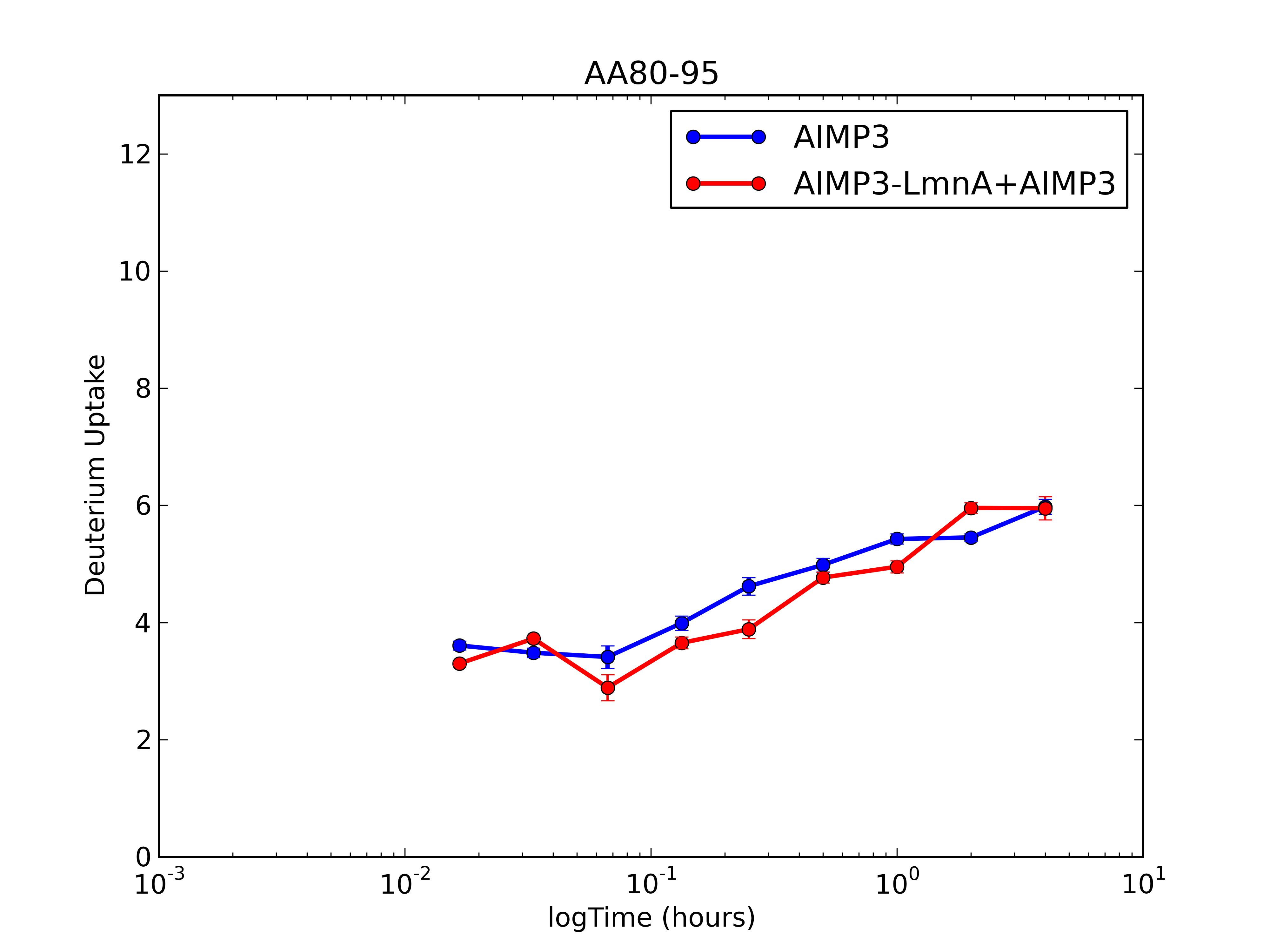

Supplement: S2 File — (ZIP) [file pone.0181869.s004.zip › logfigure-LmnA-scale/AA80-95_charge_3_mz567.6.csv.csv.png]

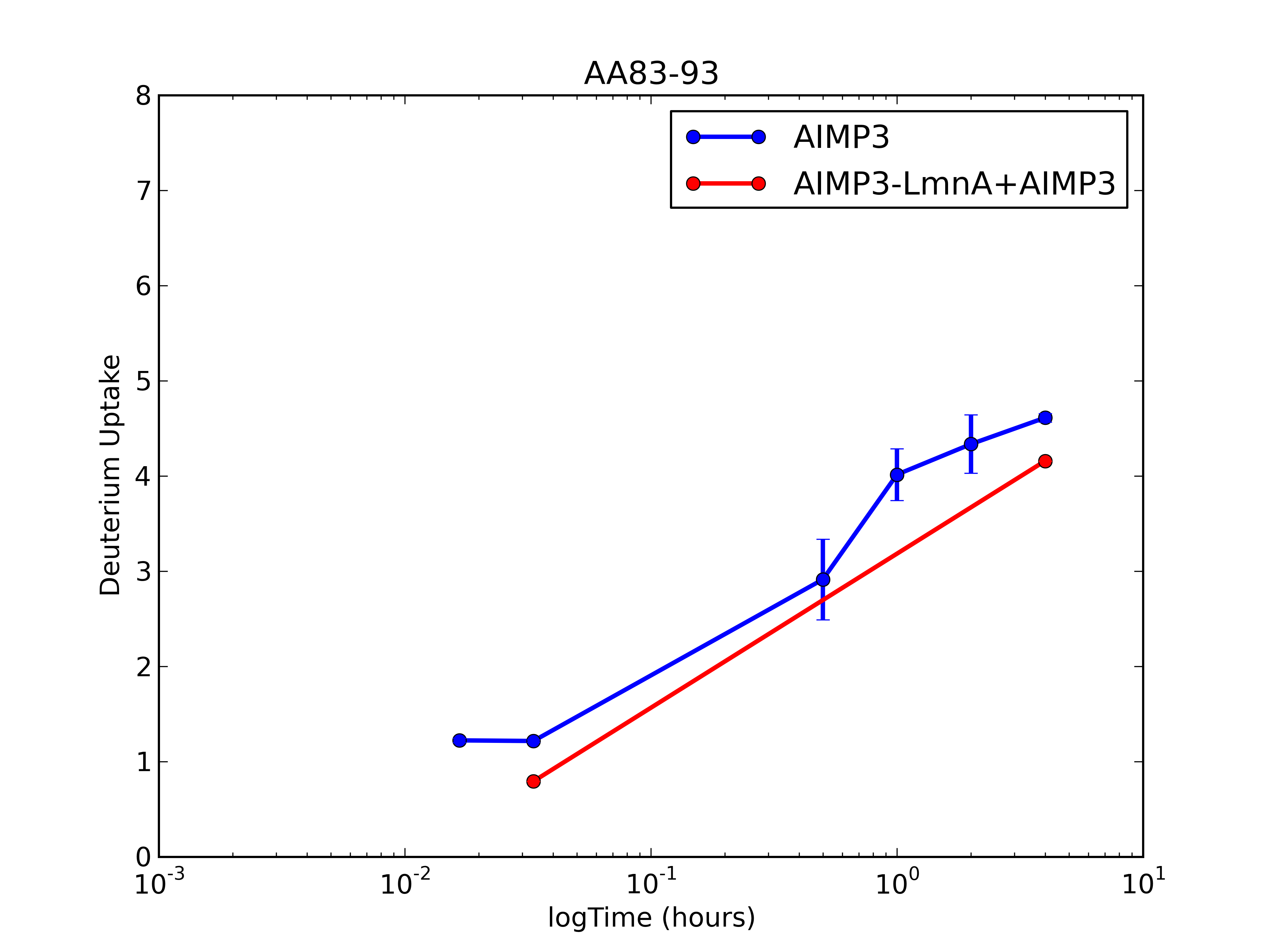

Supplement: S2 File — (ZIP) [file pone.0181869.s004.zip › logfigure-LmnA-scale/AA83-93_charge_1_mz1145.5.csv.csv.png]

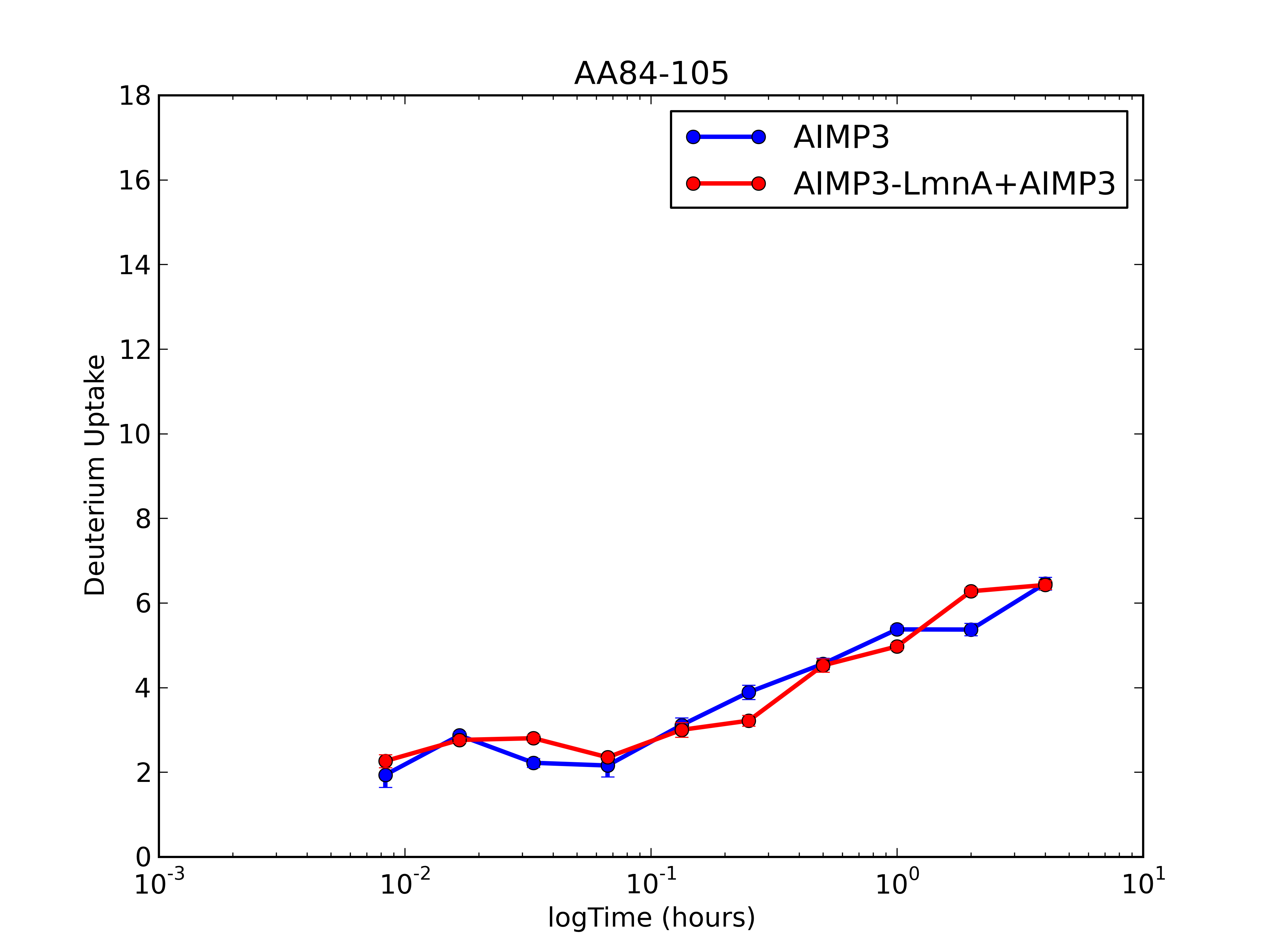

Supplement: S2 File — (ZIP) [file pone.0181869.s004.zip › logfigure-LmnA-scale/AA84-105_charge_4_mz582.5.csv.csv.png]

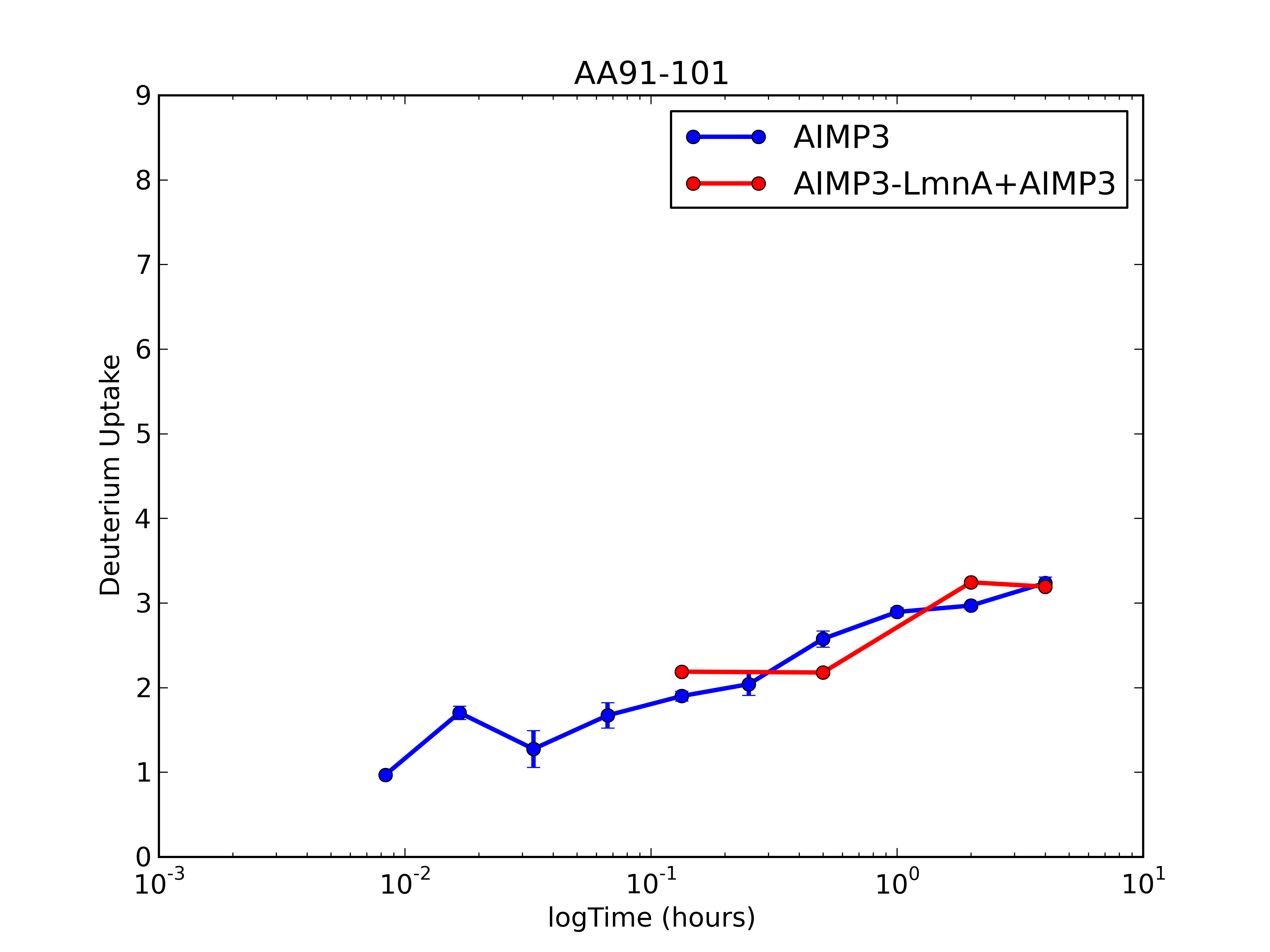

Supplement: S2 File — (ZIP) [file pone.0181869.s004.zip › logfigure-LmnA-scale/AA91-101_charge_2_mz608.3.csv.csv.png]
