# Supplementary figures and images for: Mapping the contact surfaces in the Lamin A:AIMP3 complex by hydrogen/deuterium exchange FT-ICR mass spectrometry (part 2 of 3)
Source: PLoS One. 2017 Aug 10;12(8):e0181869. doi: 10.1371/journal.pone.0181869 (PMC5552228; doi:10.1371/journal.pone.0181869)

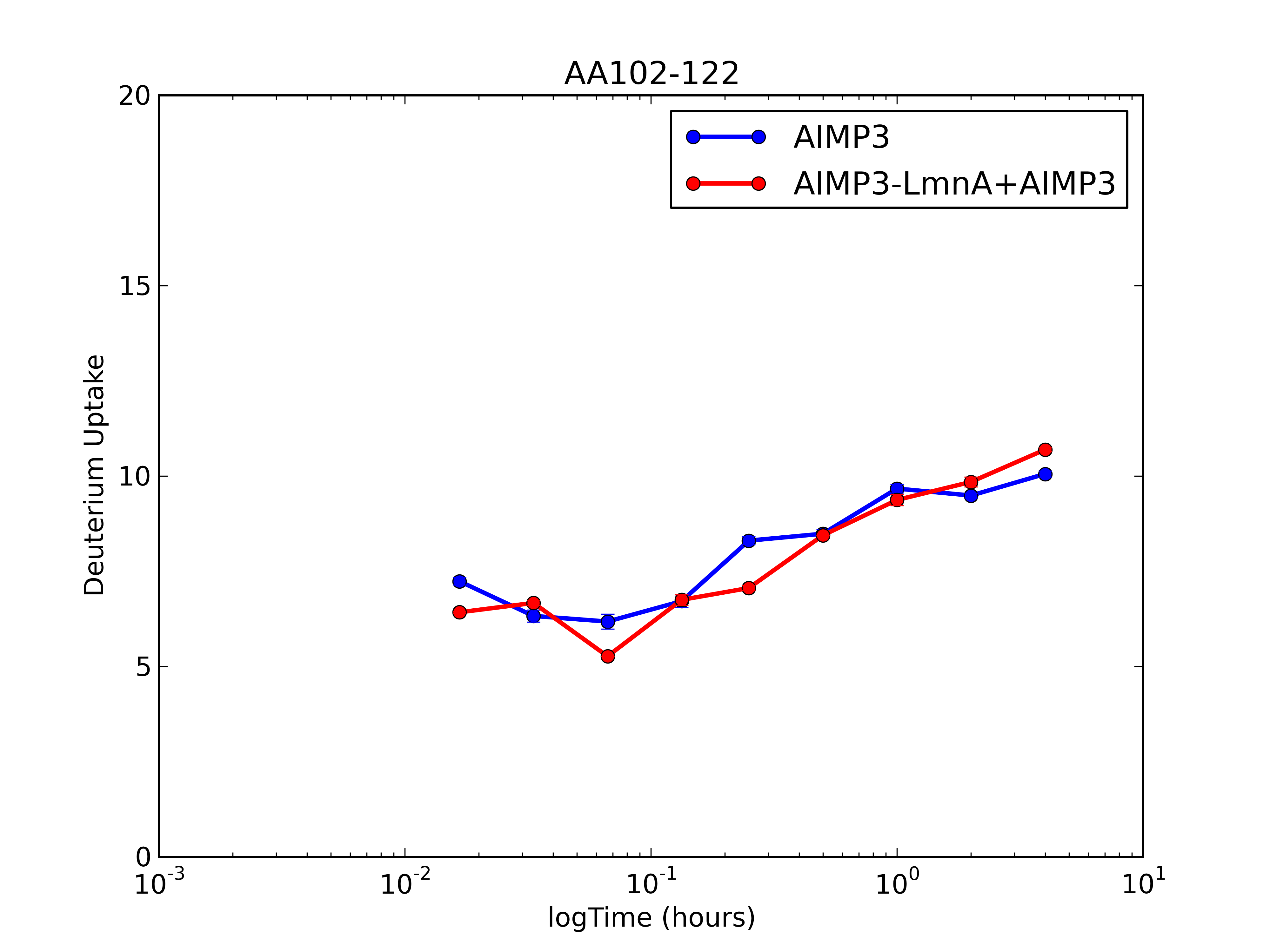

Supplement: S2 File — (ZIP) [file pone.0181869.s004.zip › logfigure-LmnA-scale/AA102-122_charge_5_mz435.8.csv.csv.png]

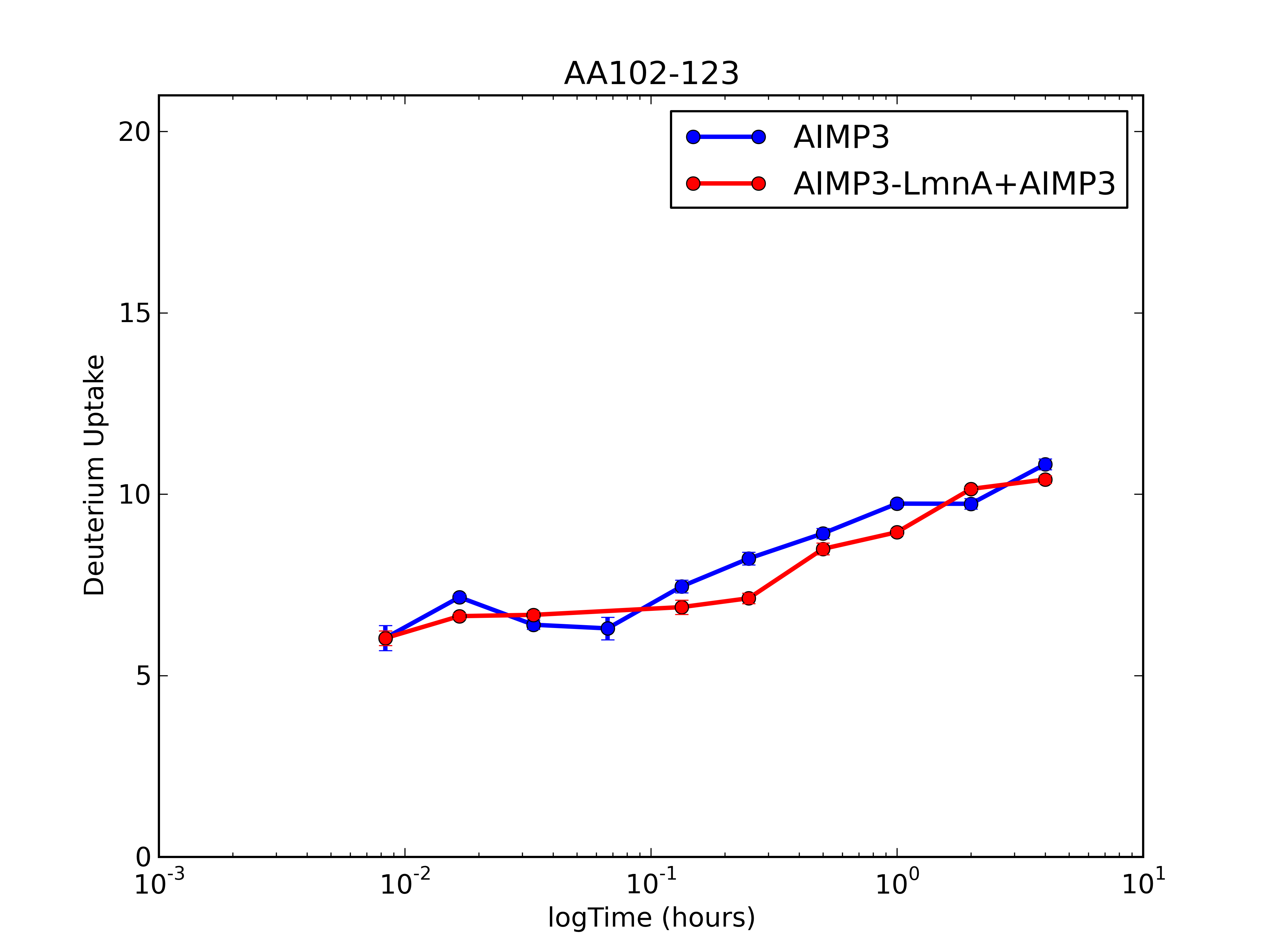

Supplement: S2 File — (ZIP) [file pone.0181869.s004.zip › logfigure-LmnA-scale/AA102-123_charge_4_mz581.3.csv.csv.png]

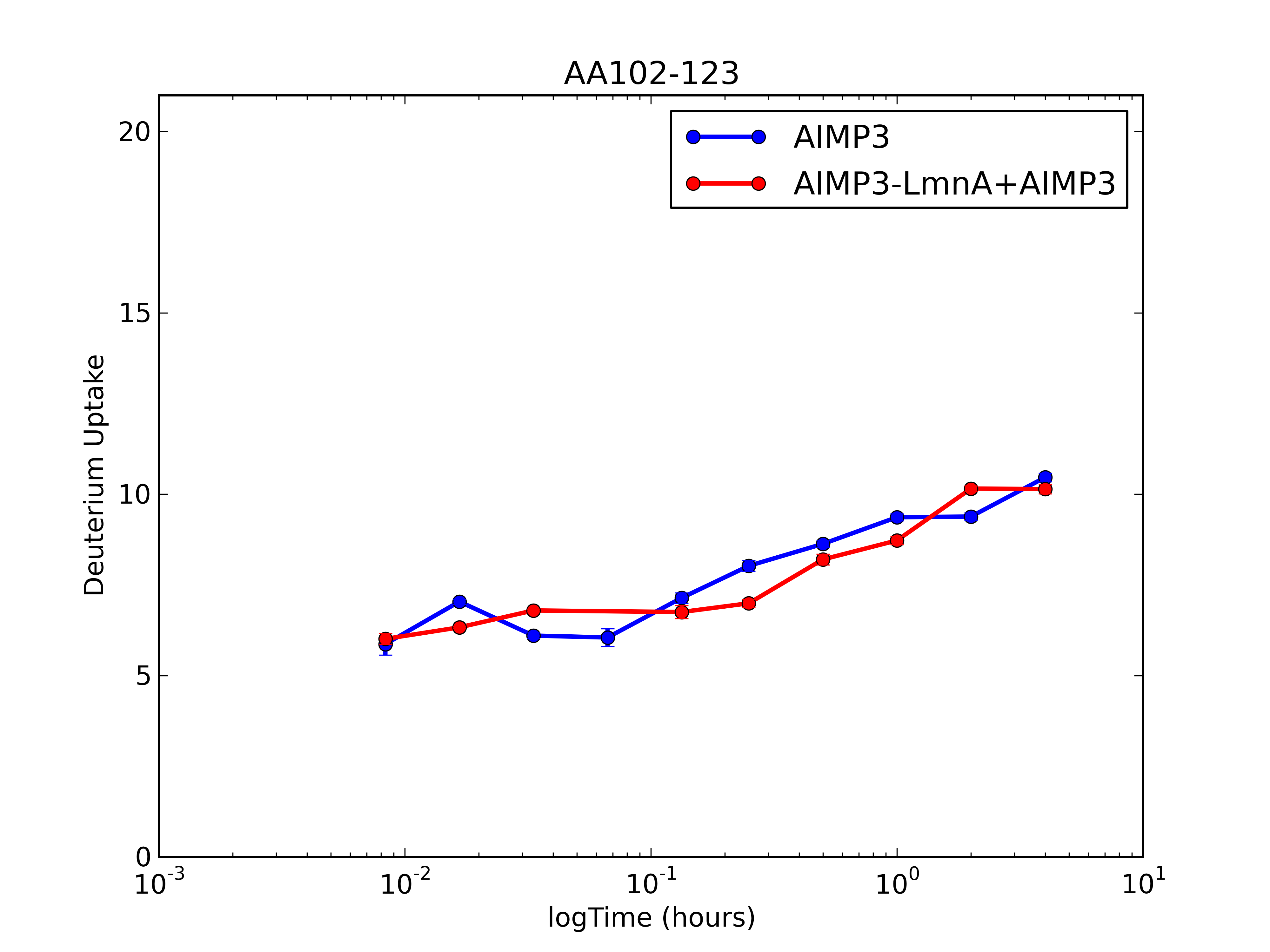

Supplement: S2 File — (ZIP) [file pone.0181869.s004.zip › logfigure-LmnA-scale/AA102-123_charge_5_mz465.2.csv.csv.png]

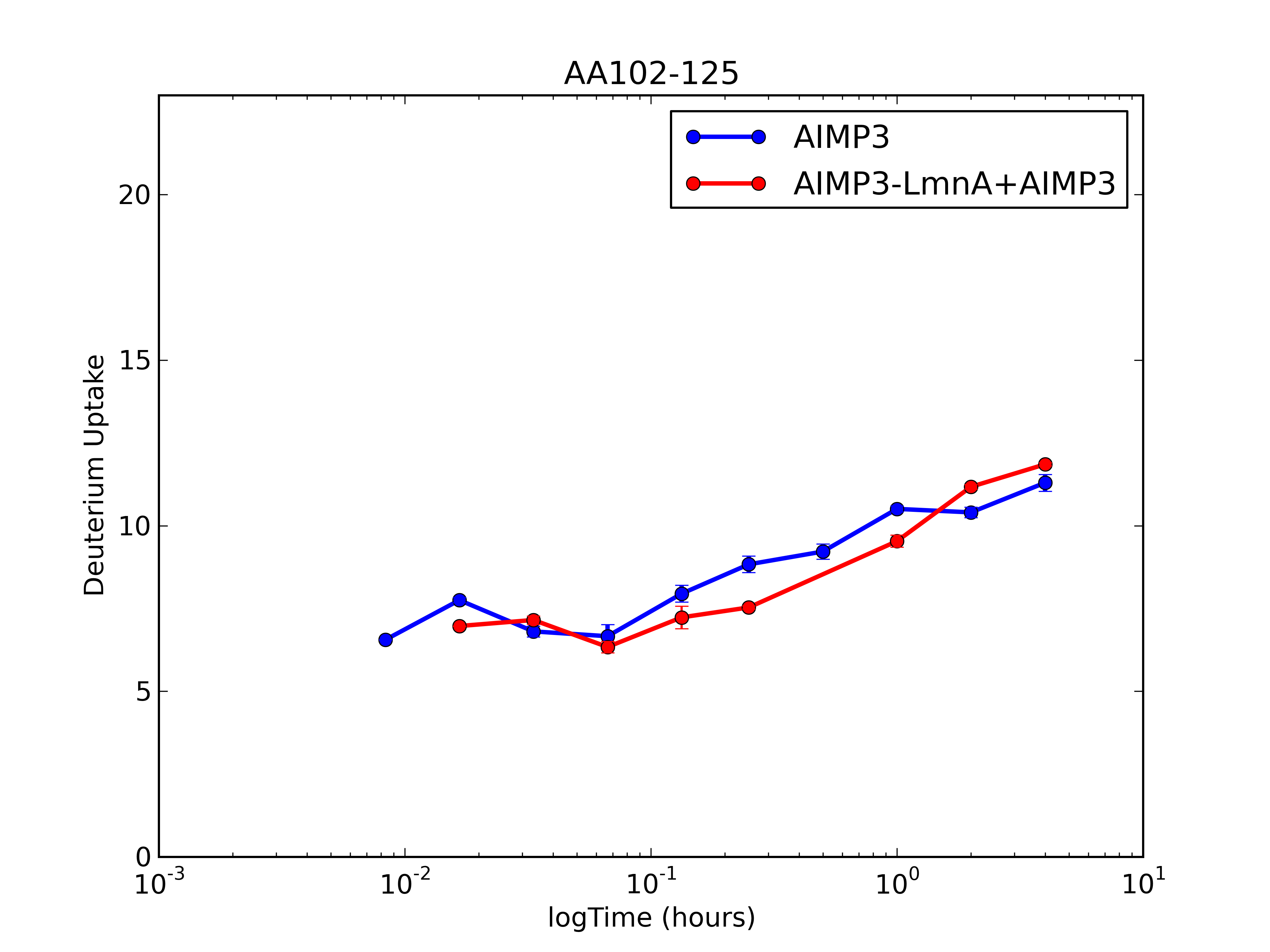

Supplement: S2 File — (ZIP) [file pone.0181869.s004.zip › logfigure-LmnA-scale/AA102-125_charge_3_mz850.8.csv.csv.png]

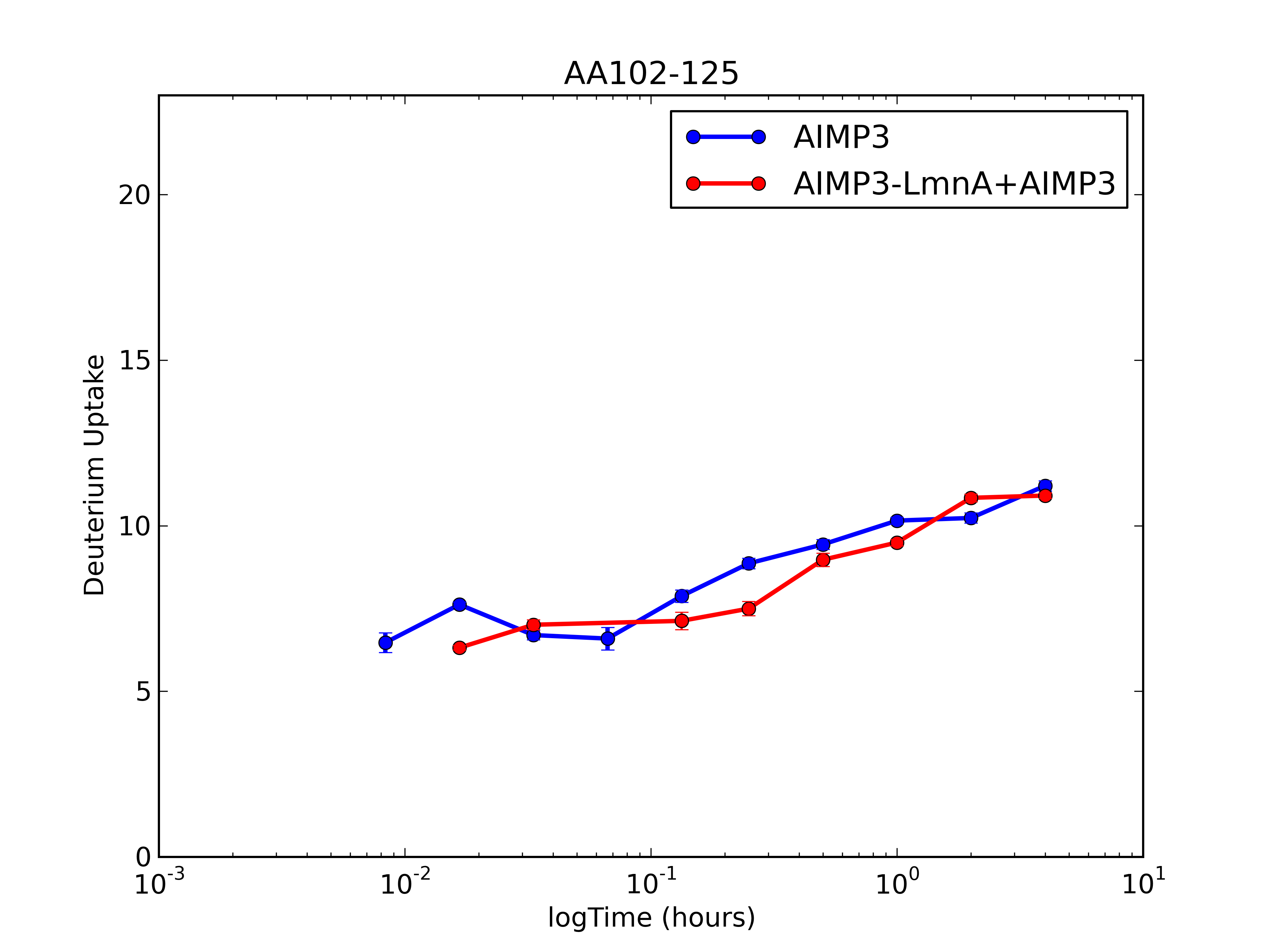

Supplement: S2 File — (ZIP) [file pone.0181869.s004.zip › logfigure-LmnA-scale/AA102-125_charge_4_mz638.3.csv.csv.png]

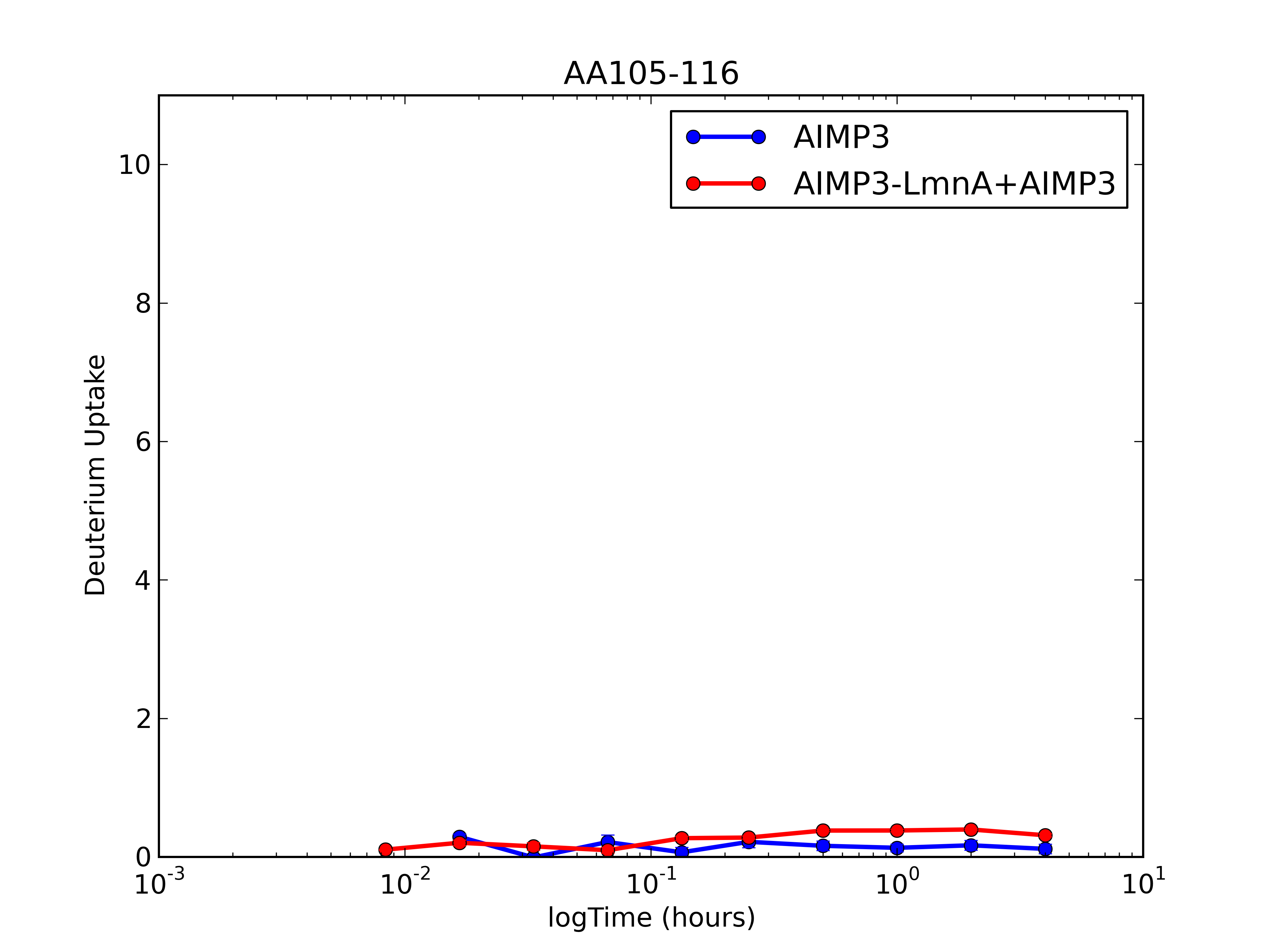

Supplement: S2 File — (ZIP) [file pone.0181869.s004.zip › logfigure-LmnA-scale/AA105-116_charge_2_mz551.8.csv.csv.png]

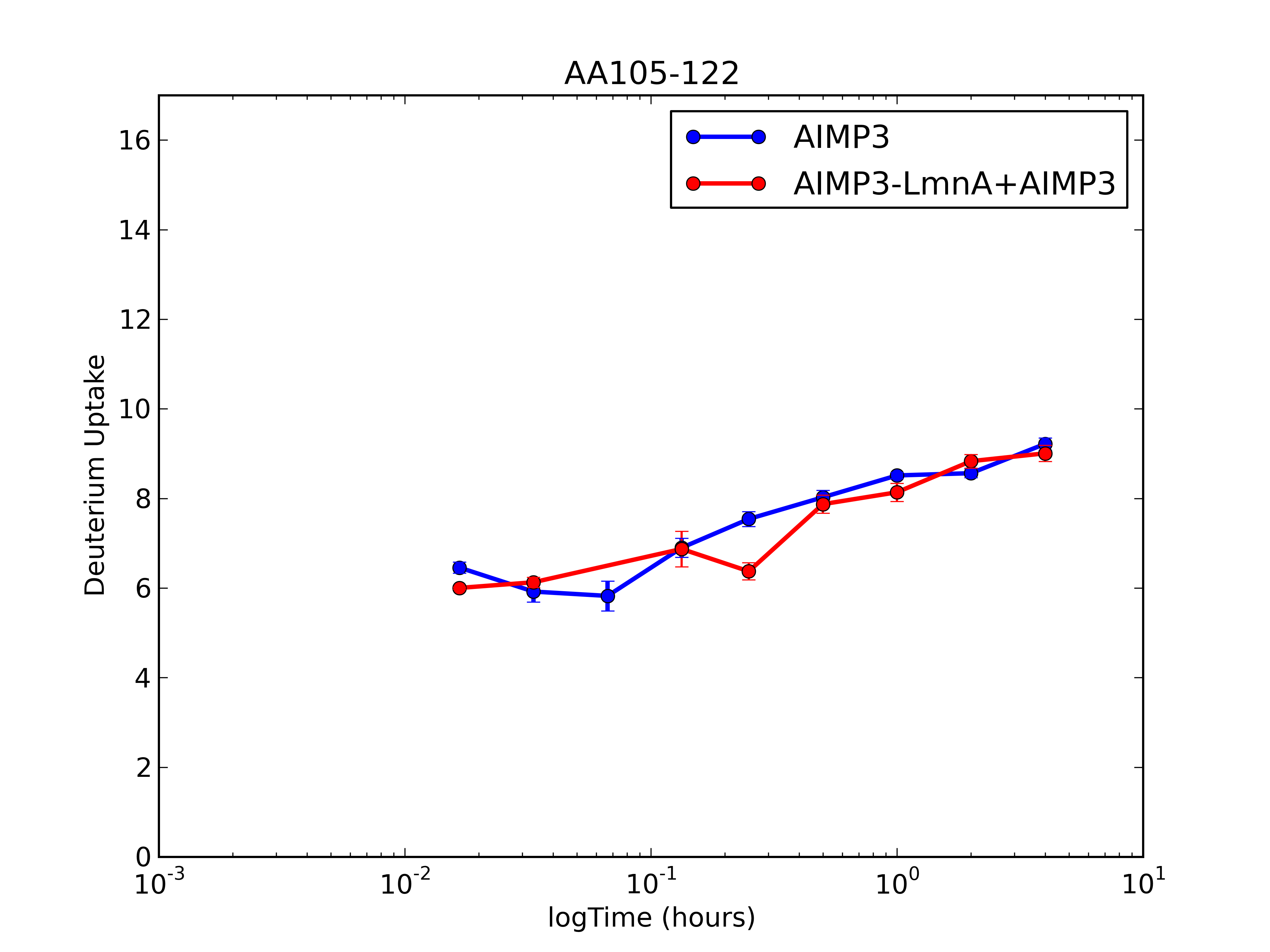

Supplement: S2 File — (ZIP) [file pone.0181869.s004.zip › logfigure-LmnA-scale/AA105-122_charge_3_mz596.0.csv.csv.png]

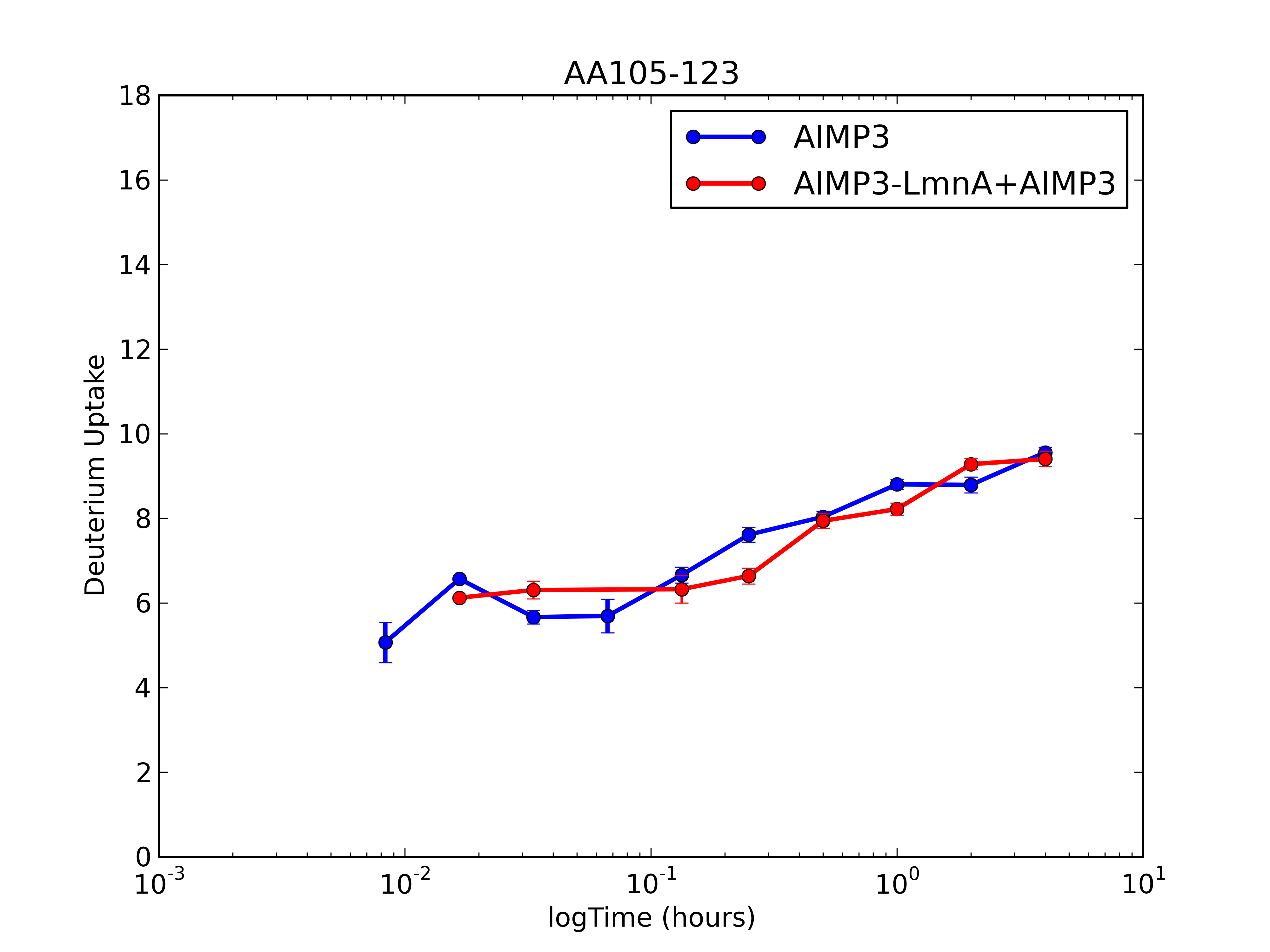

Supplement: S2 File — (ZIP) [file pone.0181869.s004.zip › logfigure-LmnA-scale/AA105-123_charge_3_mz645.0.csv.csv.png]

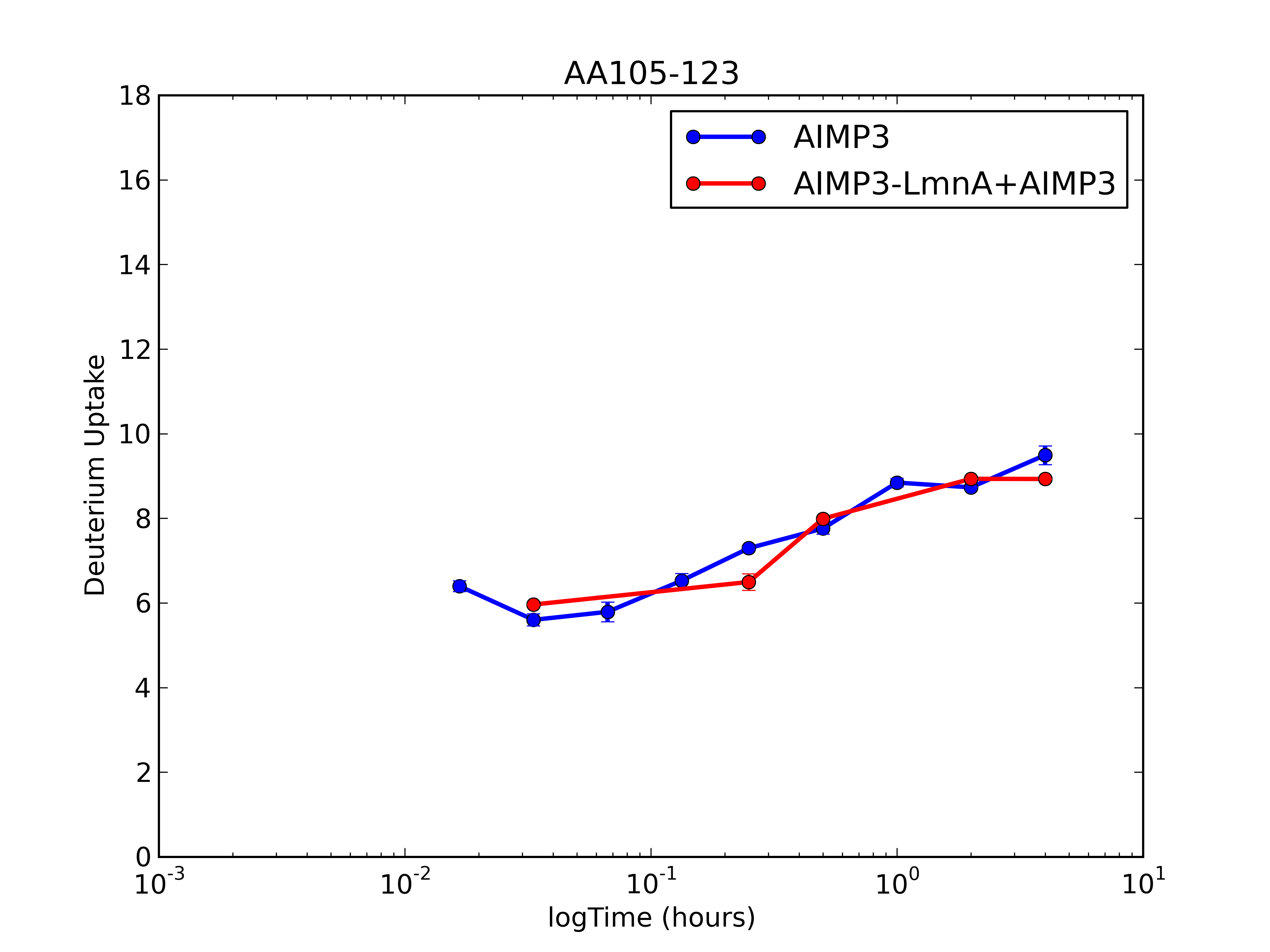

Supplement: S2 File — (ZIP) [file pone.0181869.s004.zip › logfigure-LmnA-scale/AA105-123_charge_4_mz484.0.csv.csv.png]

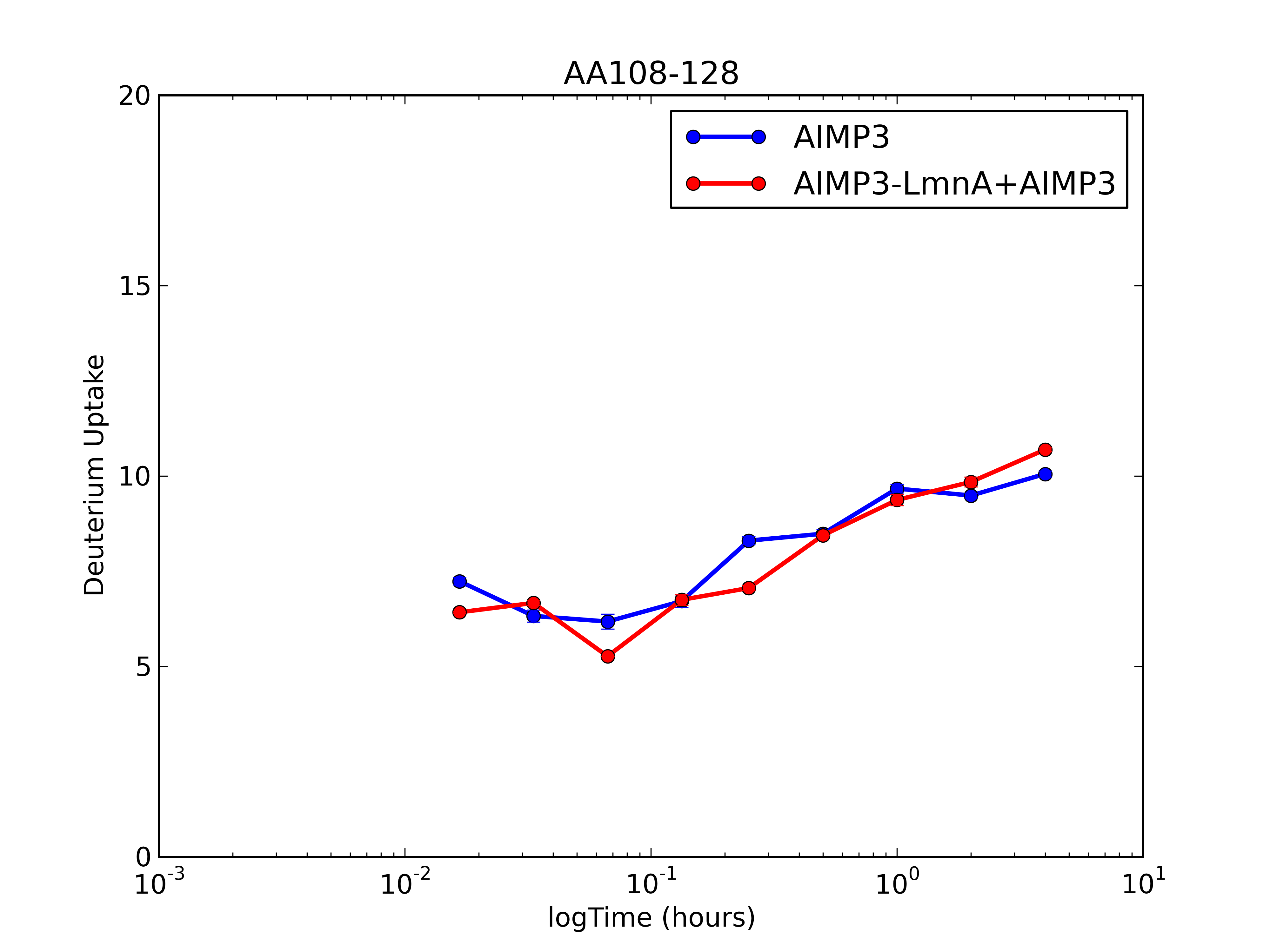

Supplement: S2 File — (ZIP) [file pone.0181869.s004.zip › logfigure-LmnA-scale/AA108-128_charge_5_mz435.6.csv.csv.png]

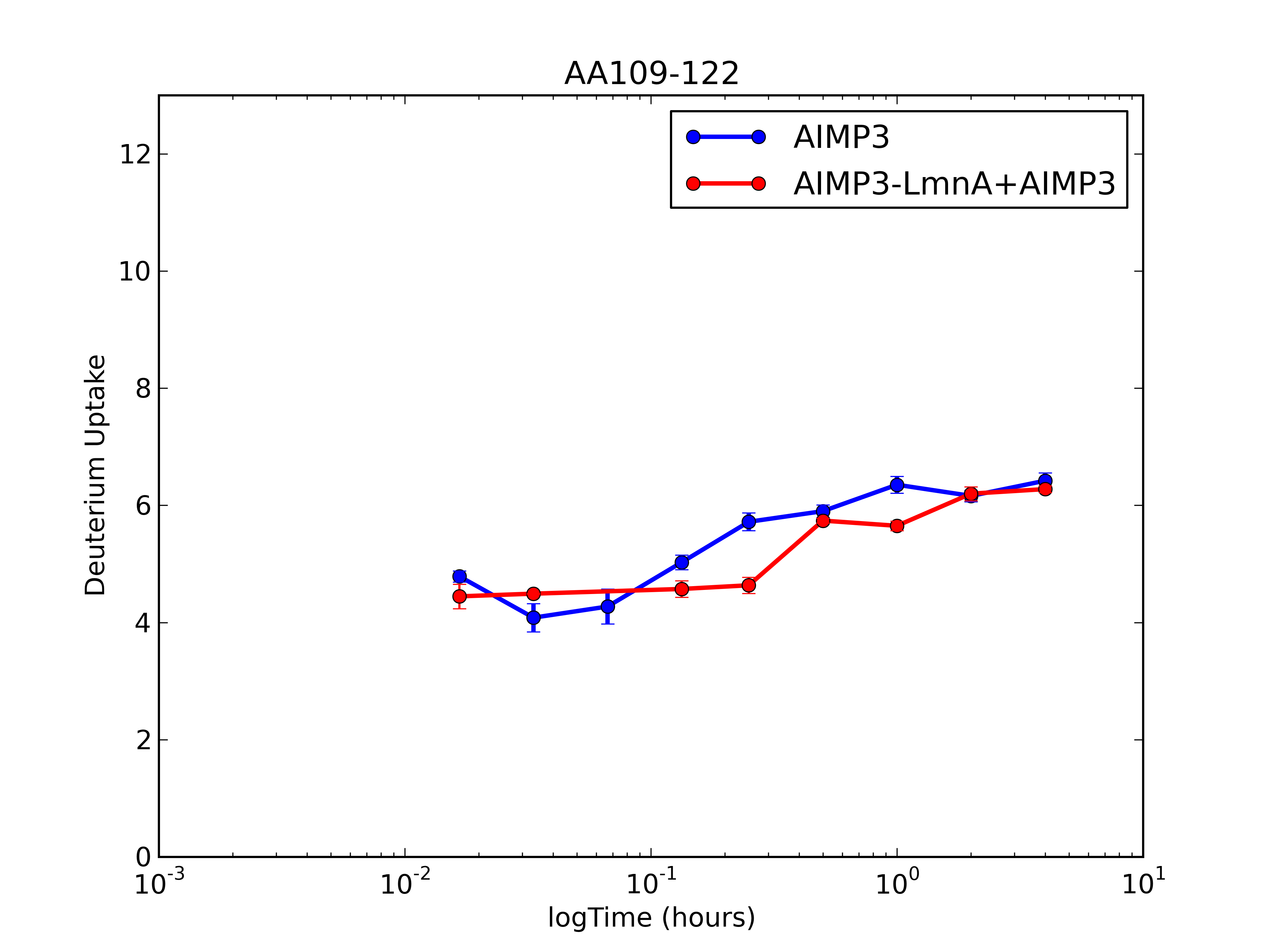

Supplement: S2 File — (ZIP) [file pone.0181869.s004.zip › logfigure-LmnA-scale/AA109-122_charge_3_mz477.2.csv.csv.png]

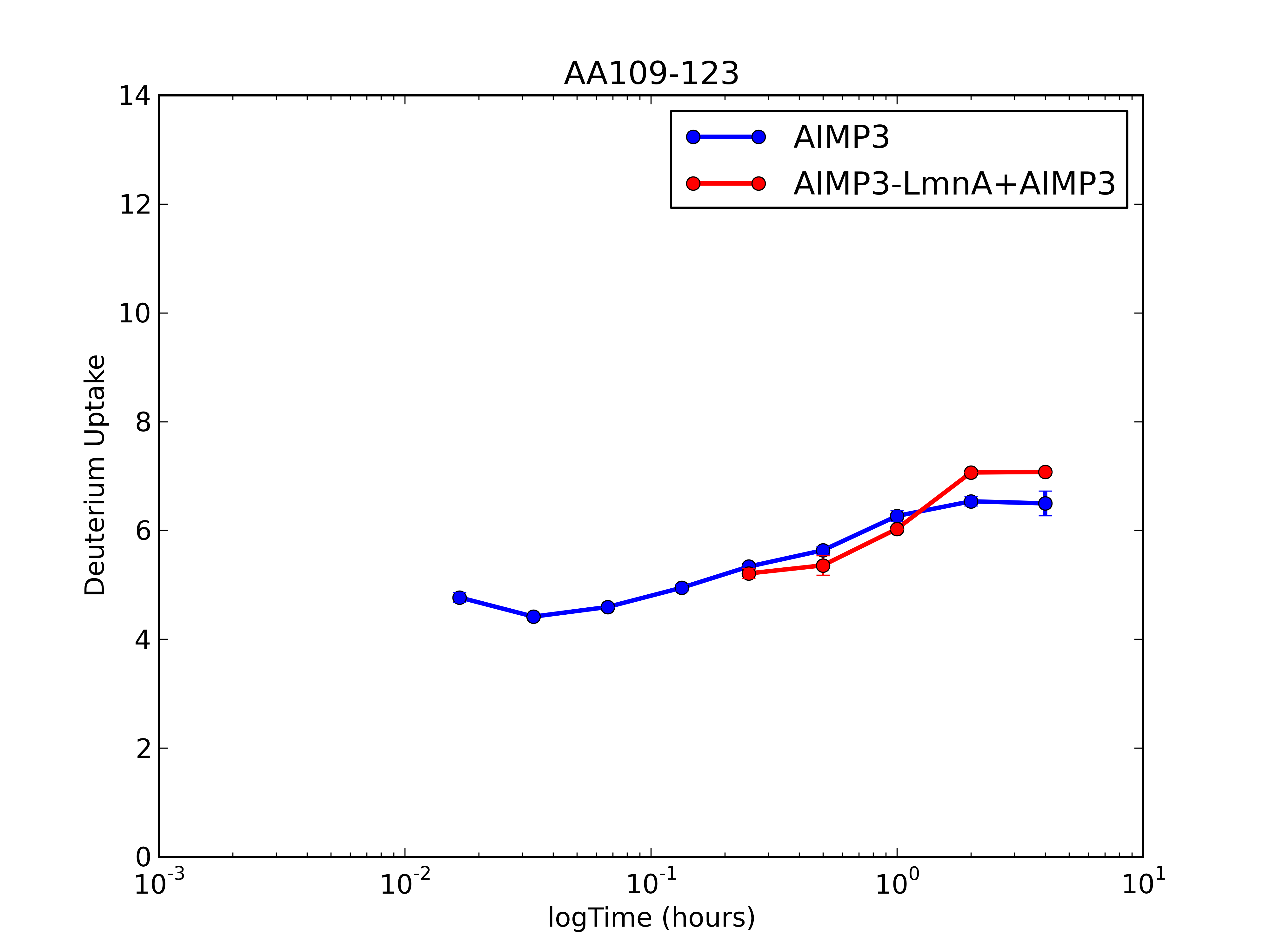

Supplement: S2 File — (ZIP) [file pone.0181869.s004.zip › logfigure-LmnA-scale/AA109-123_charge_3_mz526.3.csv.csv.png]

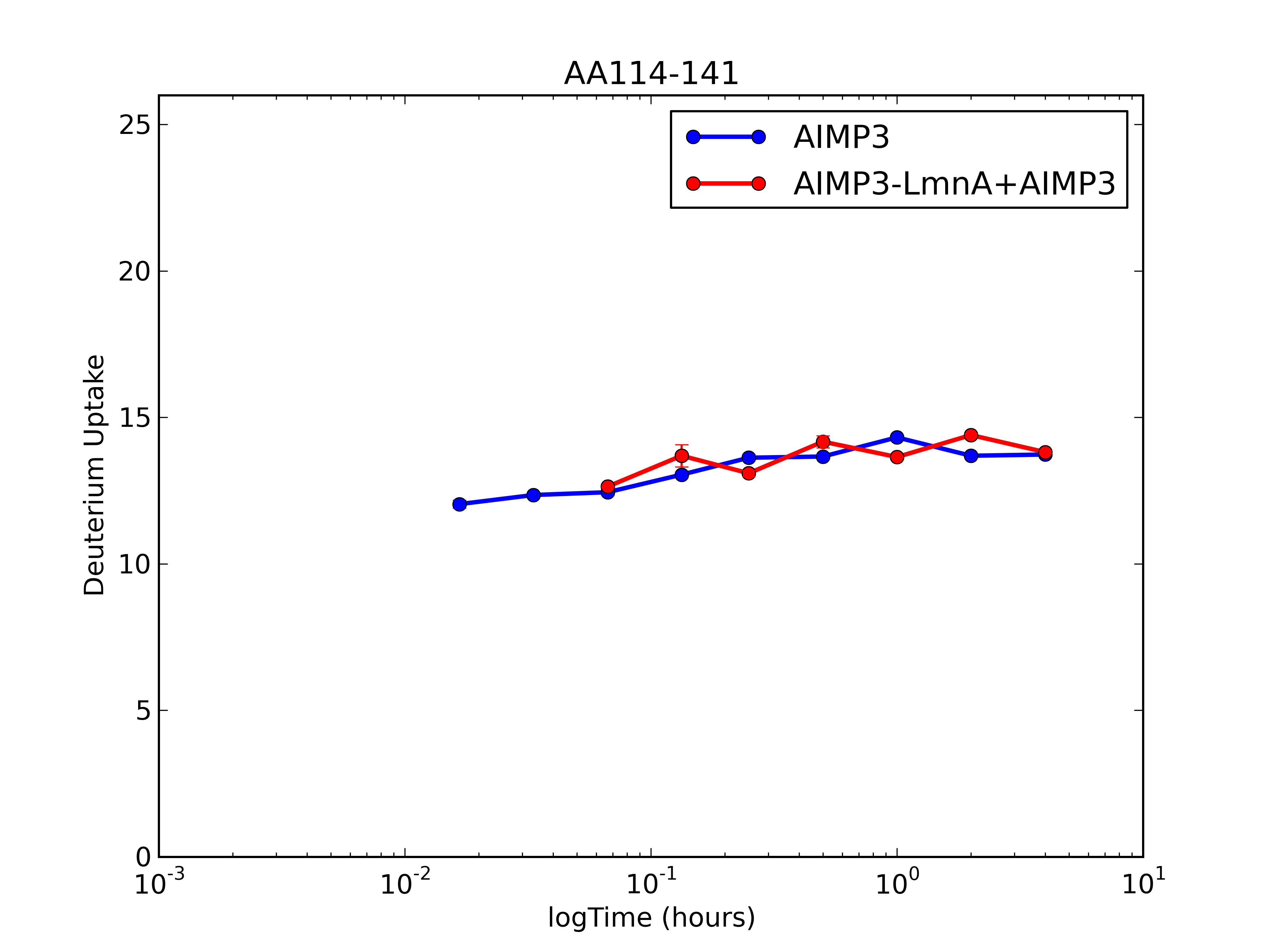

Supplement: S2 File — (ZIP) [file pone.0181869.s004.zip › logfigure-LmnA-scale/AA114-141_charge_4_mz708.8.csv.csv.png]

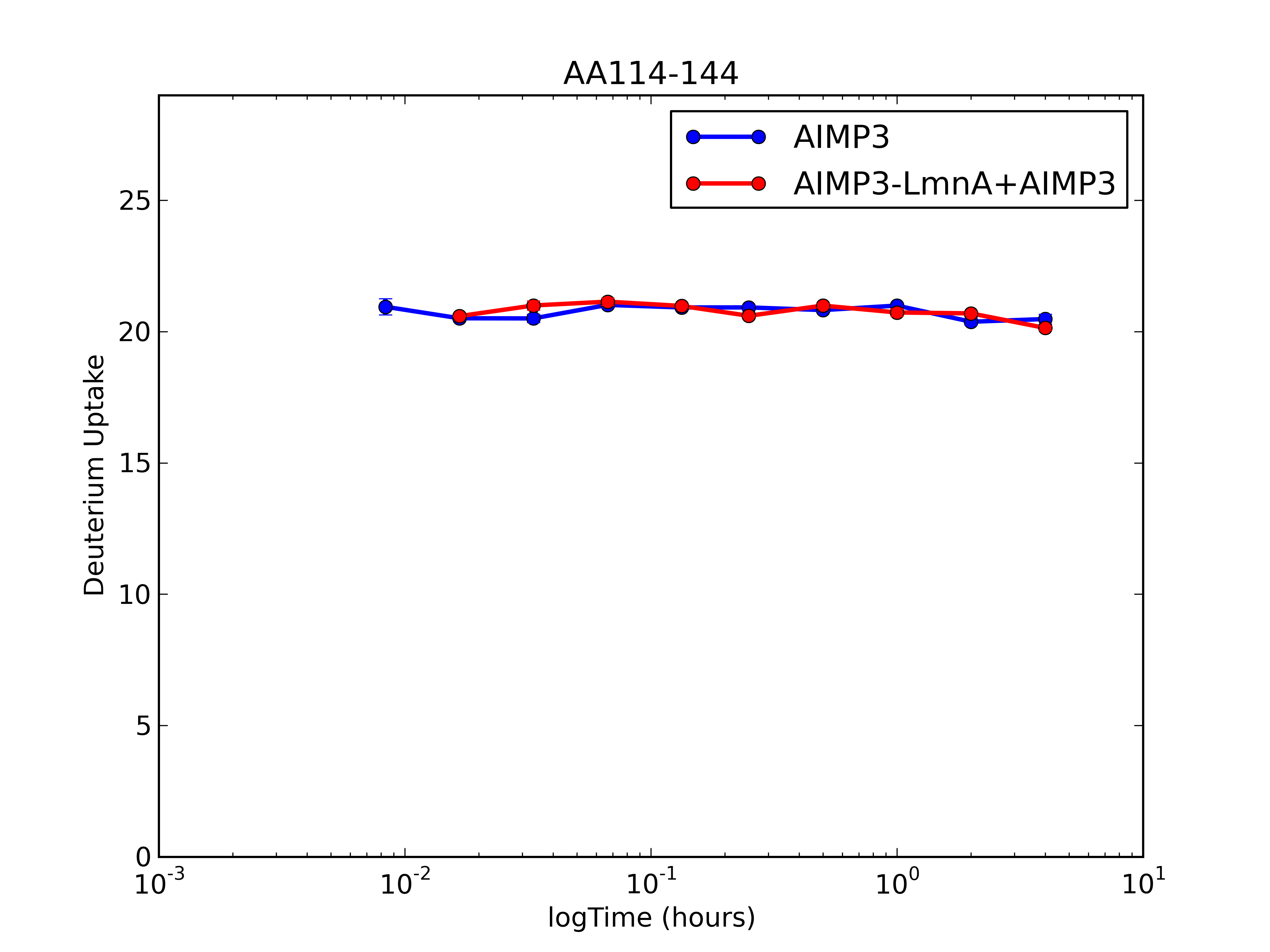

Supplement: S2 File — (ZIP) [file pone.0181869.s004.zip › logfigure-LmnA-scale/AA114-144_charge_3_mz1074.8.csv.csv.png]

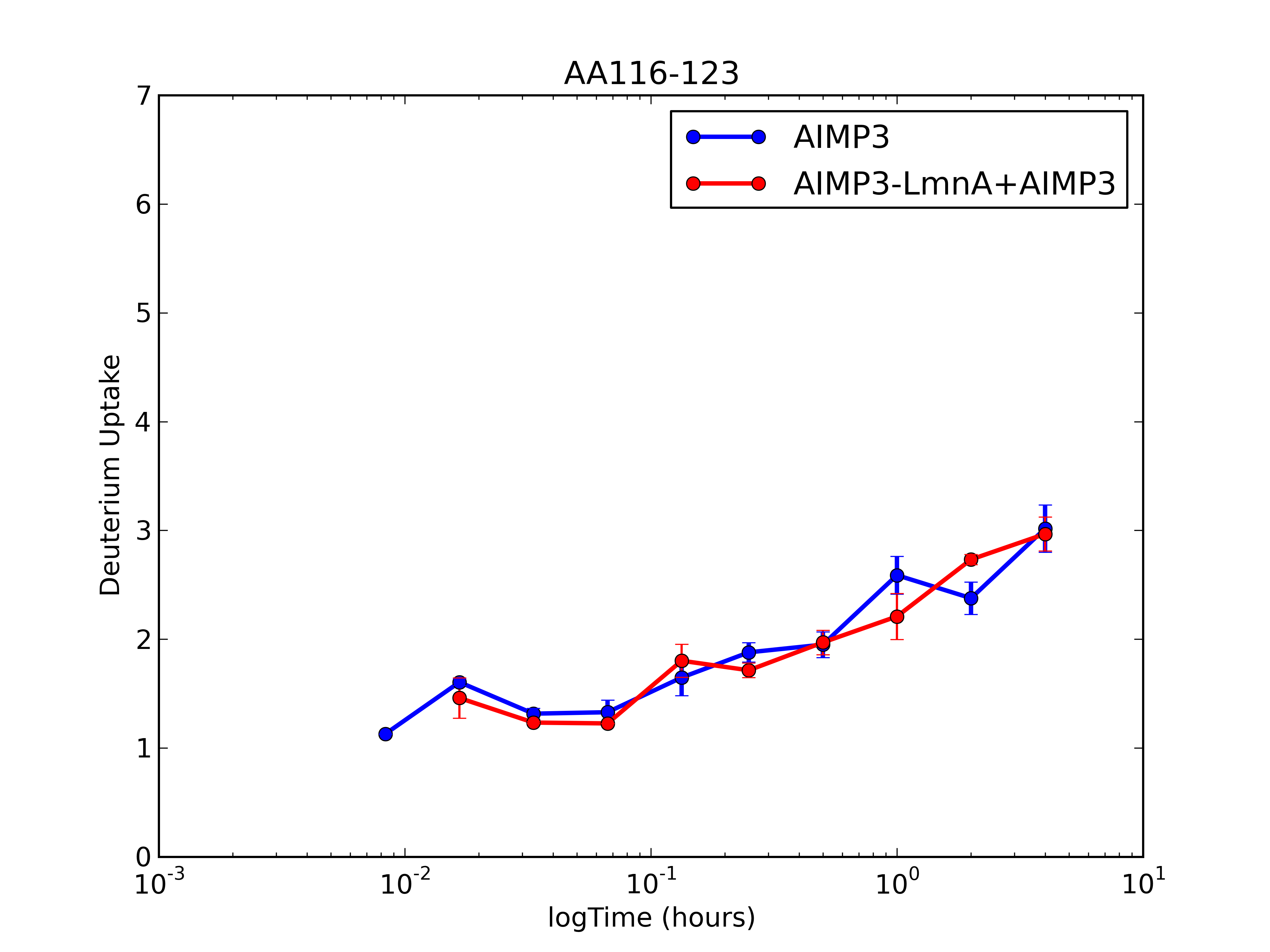

Supplement: S2 File — (ZIP) [file pone.0181869.s004.zip › logfigure-LmnA-scale/AA116-123_charge_2_mz468.7.csv.csv.png]

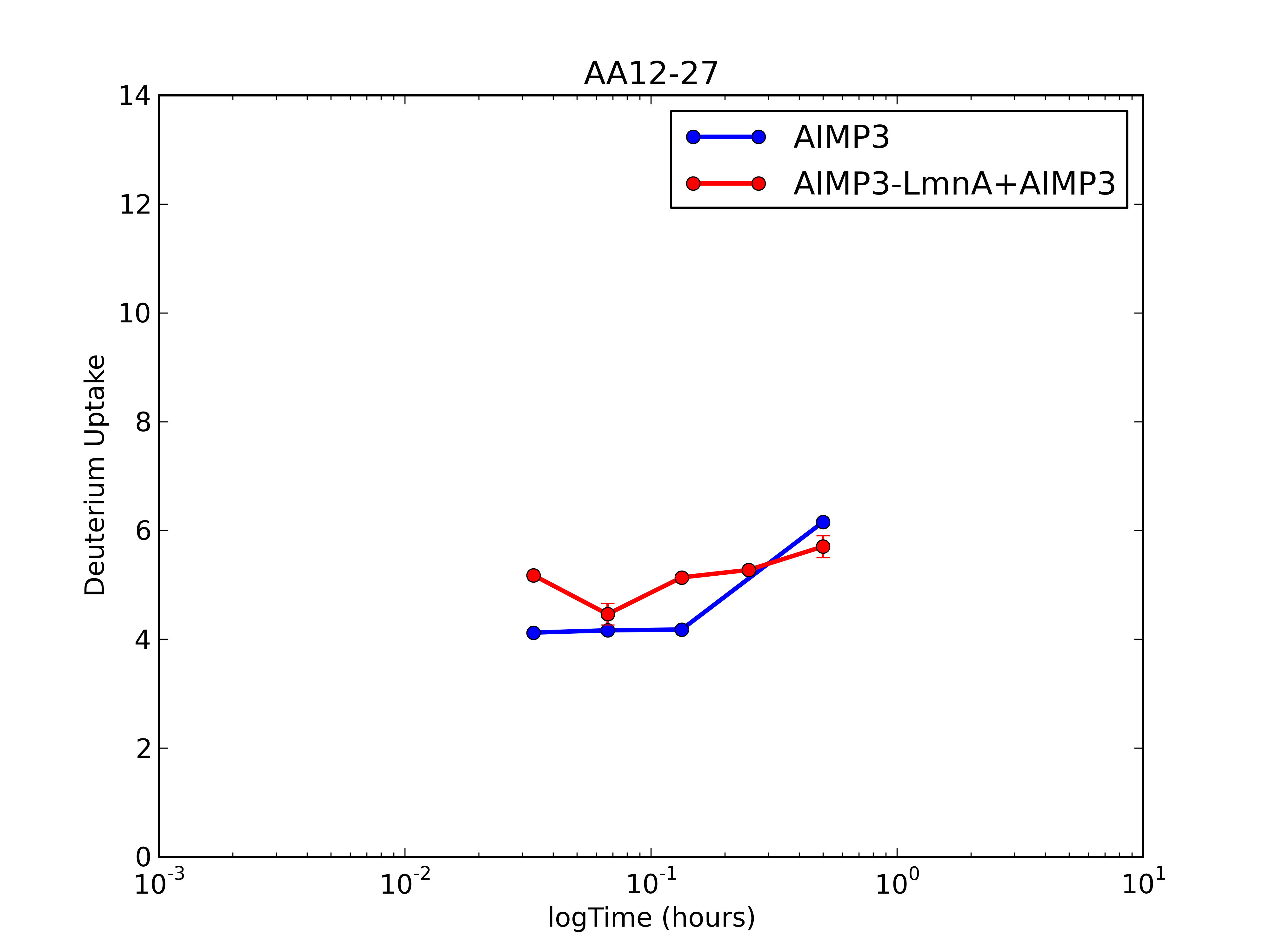

Supplement: S2 File — (ZIP) [file pone.0181869.s004.zip › logfigure-LmnA-scale/AA12-27_charge_3_mz618.9.csv.csv.png]

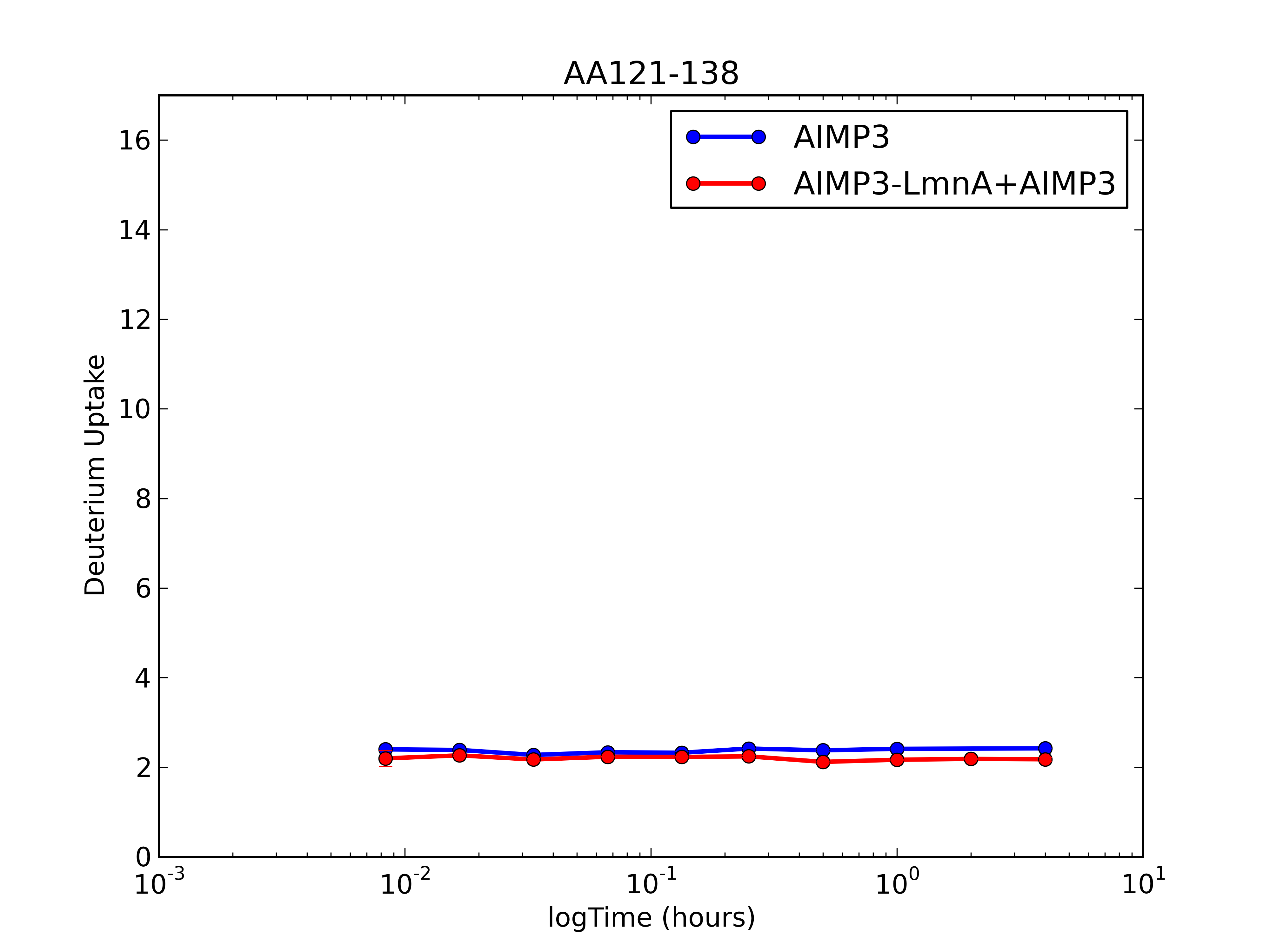

Supplement: S2 File — (ZIP) [file pone.0181869.s004.zip › logfigure-LmnA-scale/AA121-138_charge_4_mz460.2.csv.csv.png]

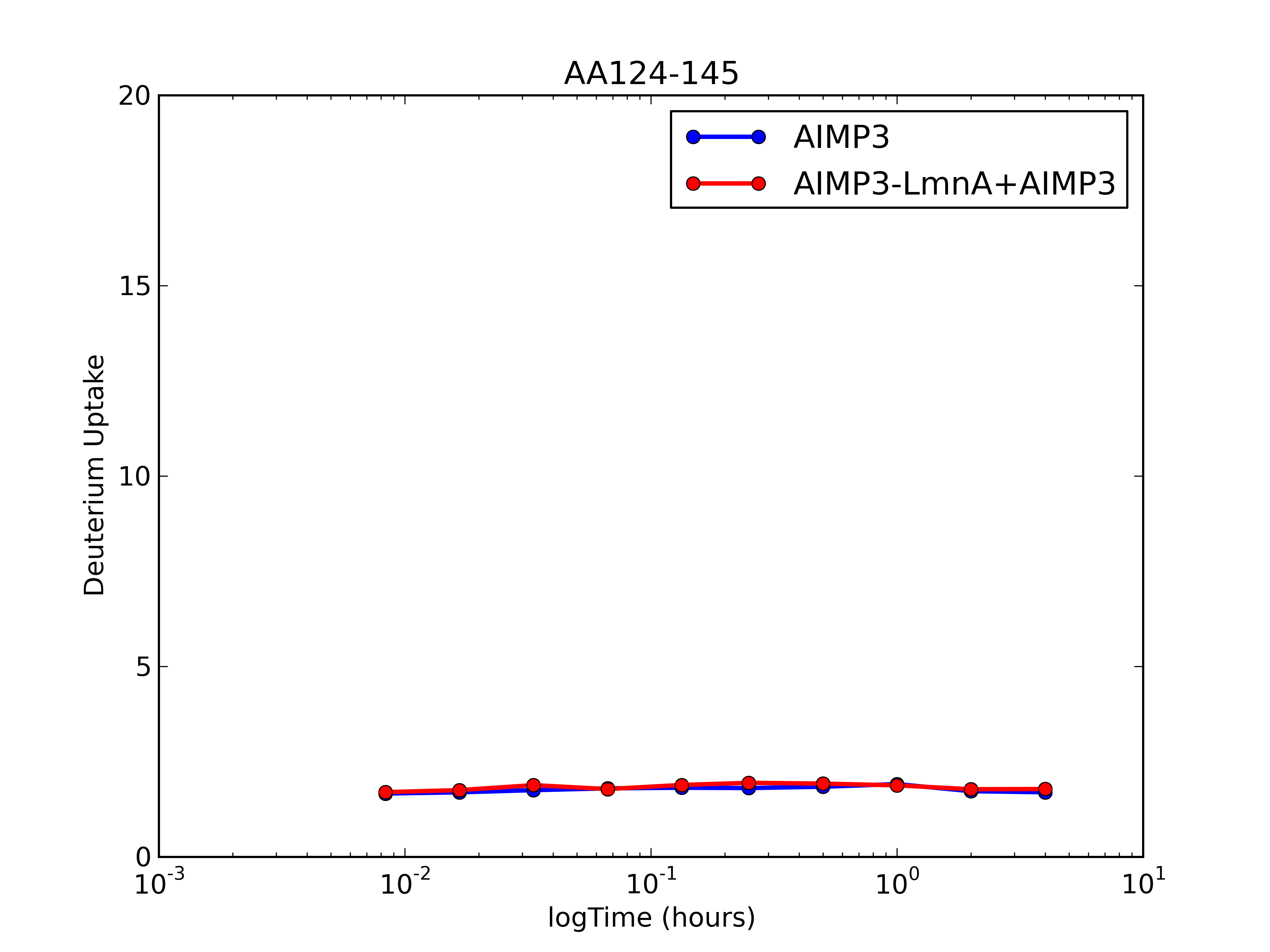

Supplement: S2 File — (ZIP) [file pone.0181869.s004.zip › logfigure-LmnA-scale/AA124-145_charge_3_mz759.6.csv.csv.png]

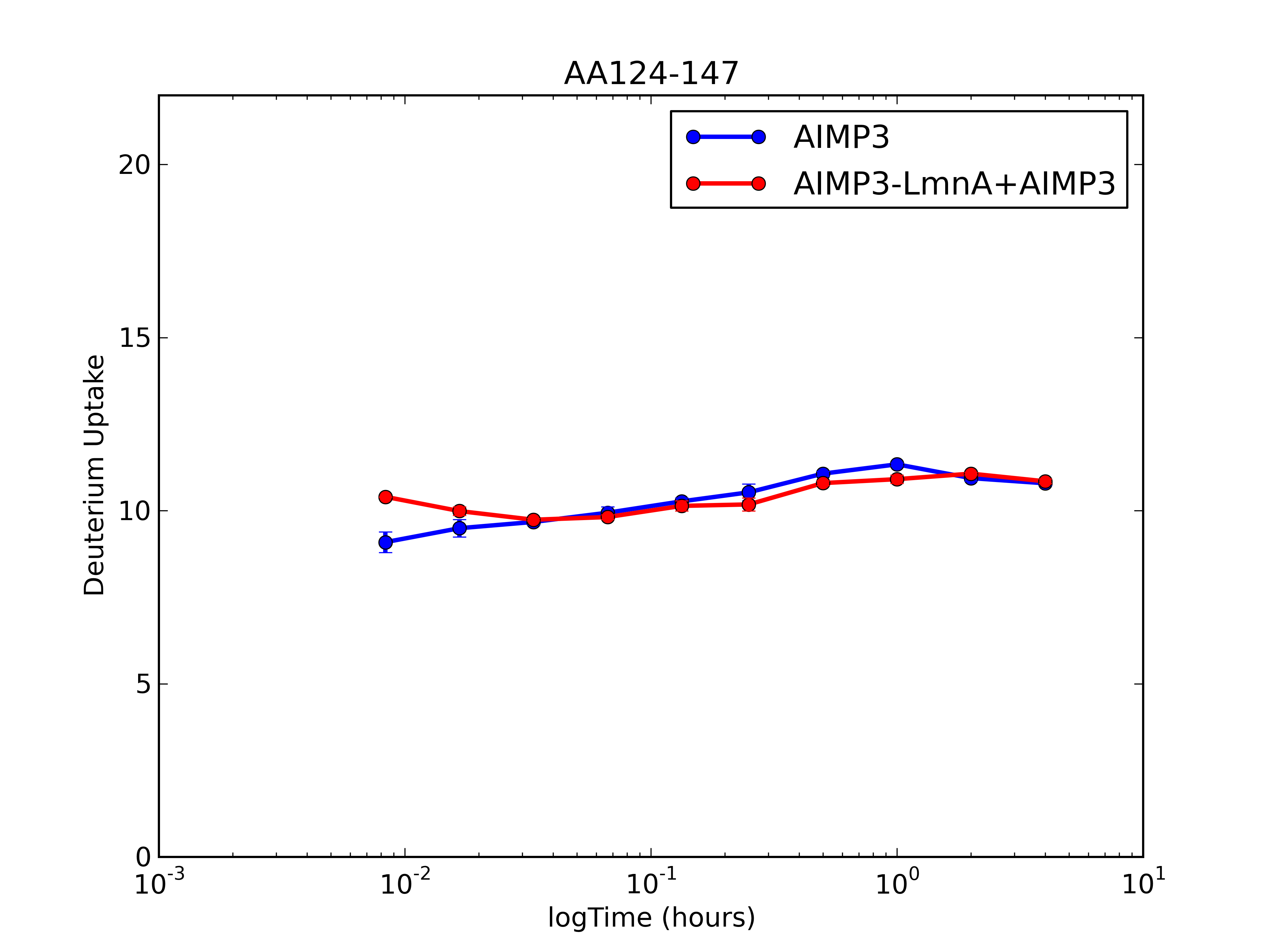

Supplement: S2 File — (ZIP) [file pone.0181869.s004.zip › logfigure-LmnA-scale/AA124-147_charge_3_mz840.7.csv.csv.png]

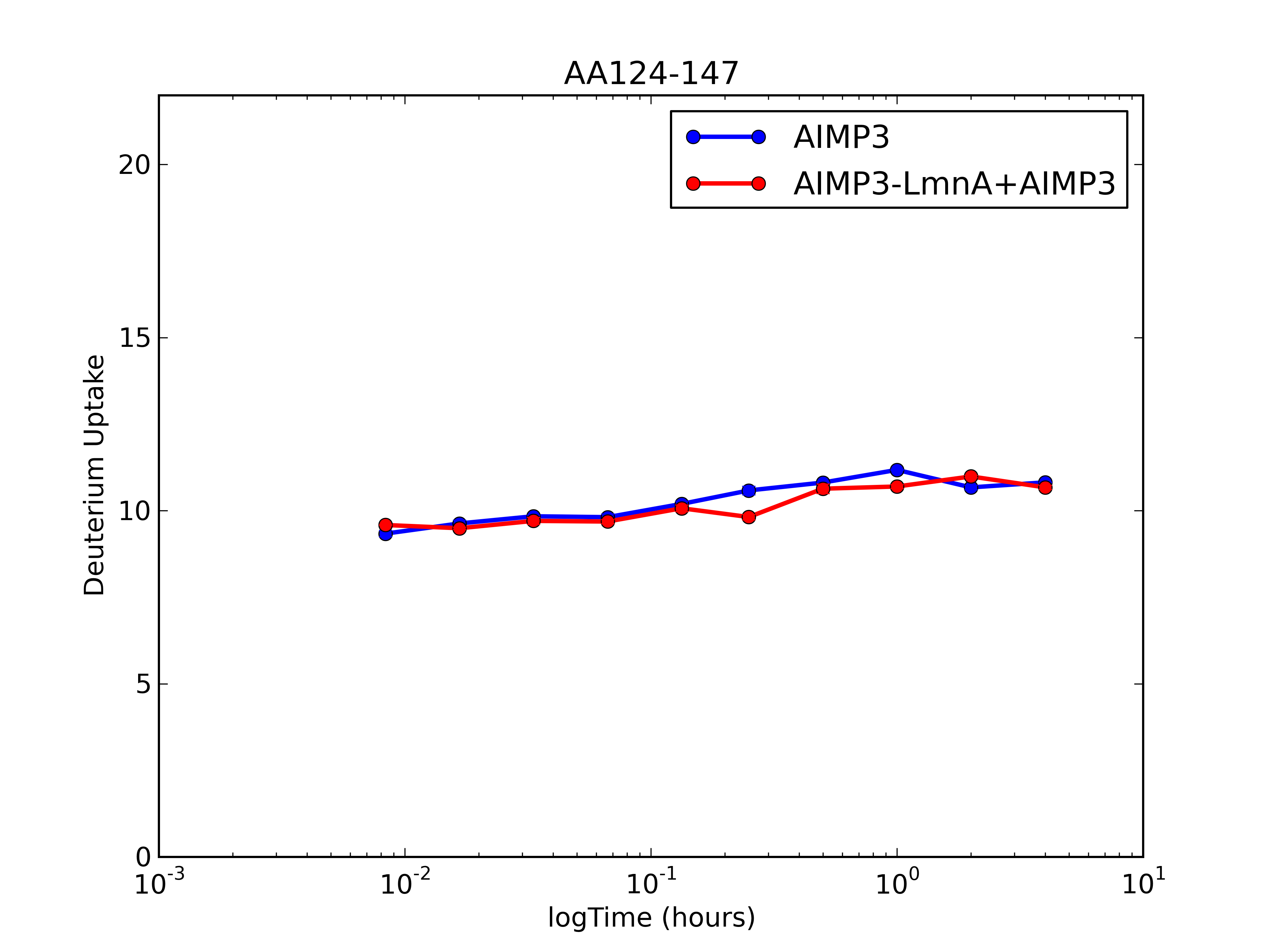

Supplement: S2 File — (ZIP) [file pone.0181869.s004.zip › logfigure-LmnA-scale/AA124-147_charge_4_mz630.7.csv.csv.png]

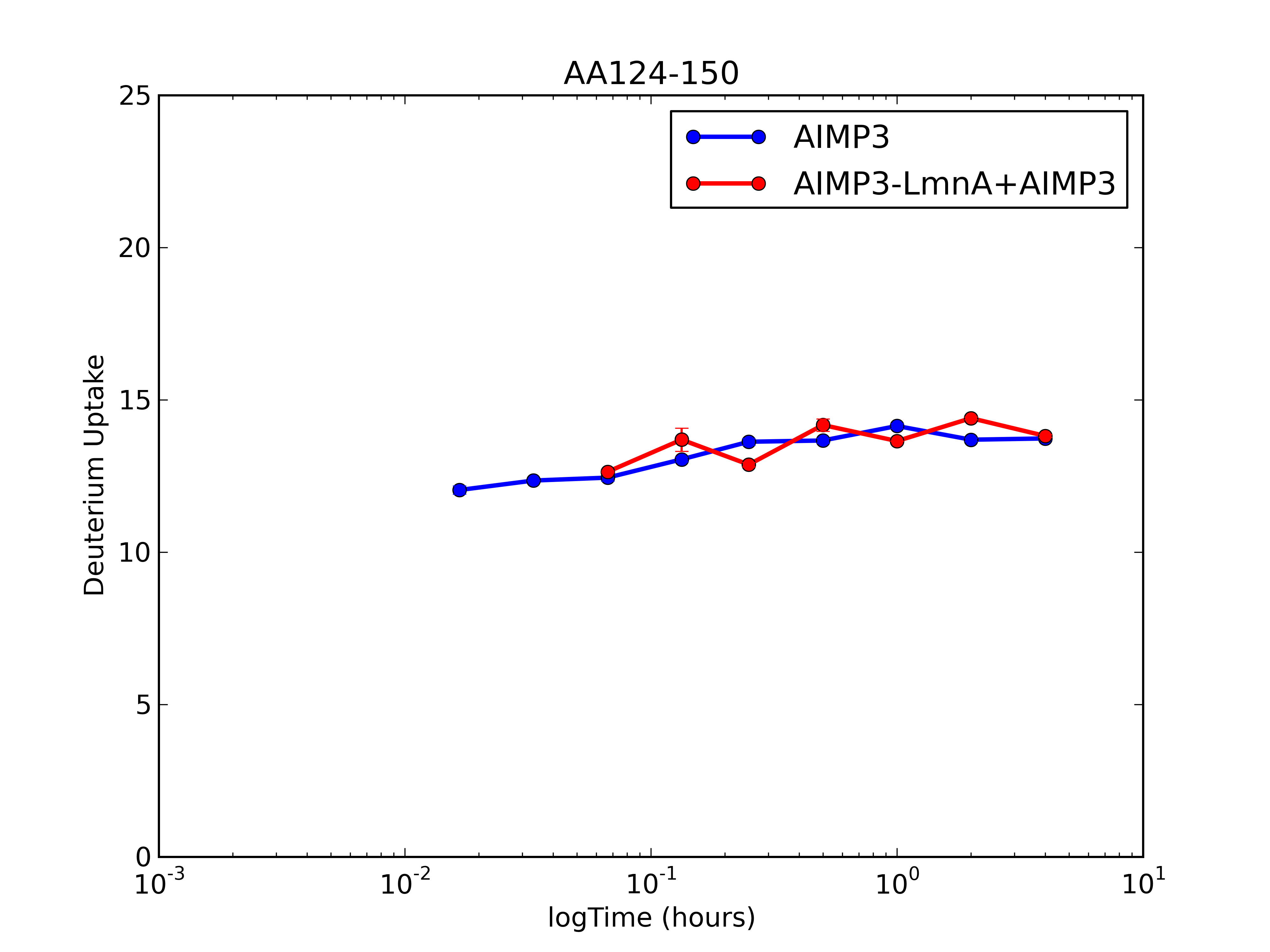

Supplement: S2 File — (ZIP) [file pone.0181869.s004.zip › logfigure-LmnA-scale/AA124-150_charge_4_mz709.0.csv.csv.png]

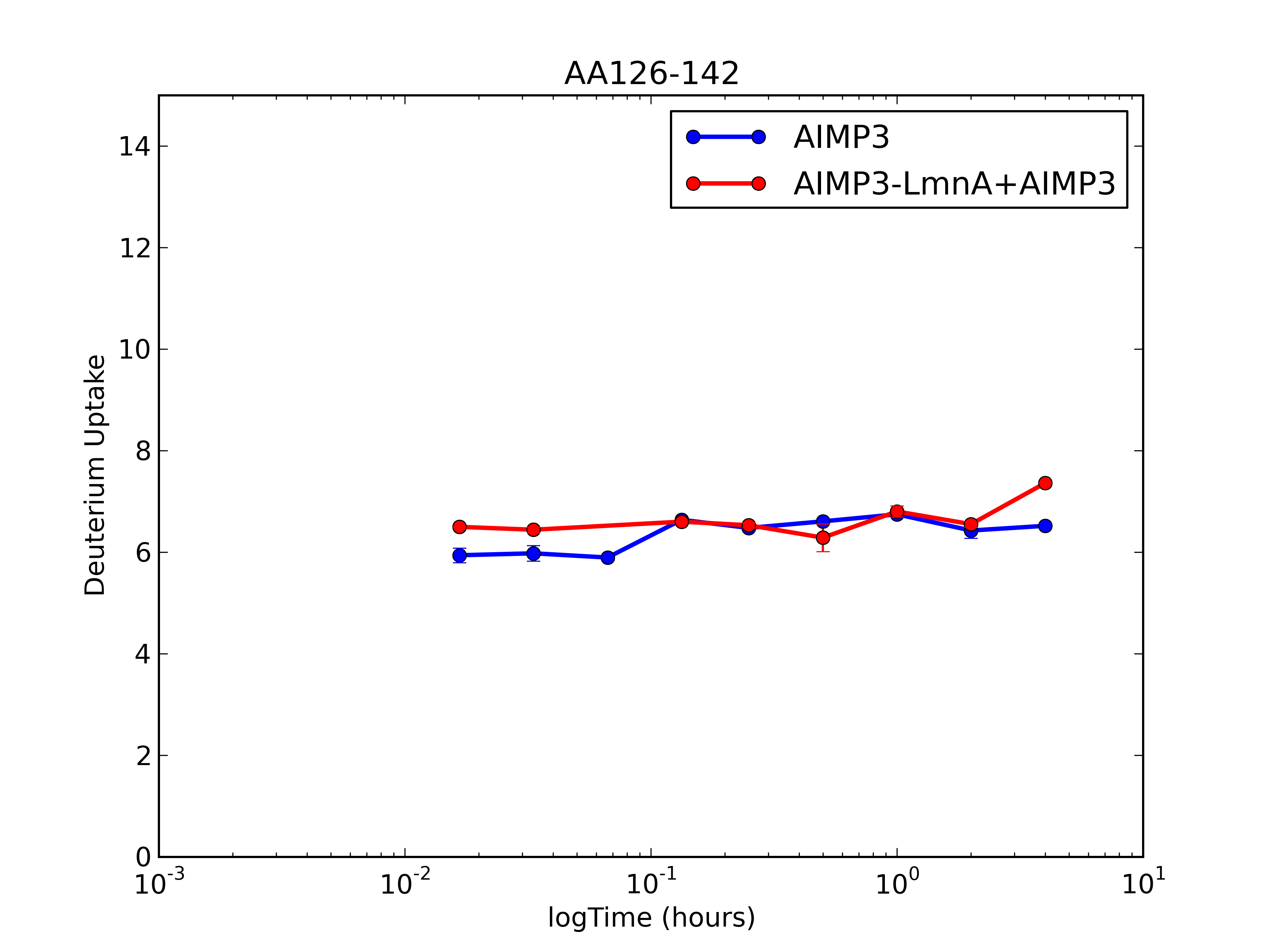

Supplement: S2 File — (ZIP) [file pone.0181869.s004.zip › logfigure-LmnA-scale/AA126-142_charge_2_mz833.3.csv.csv.png]

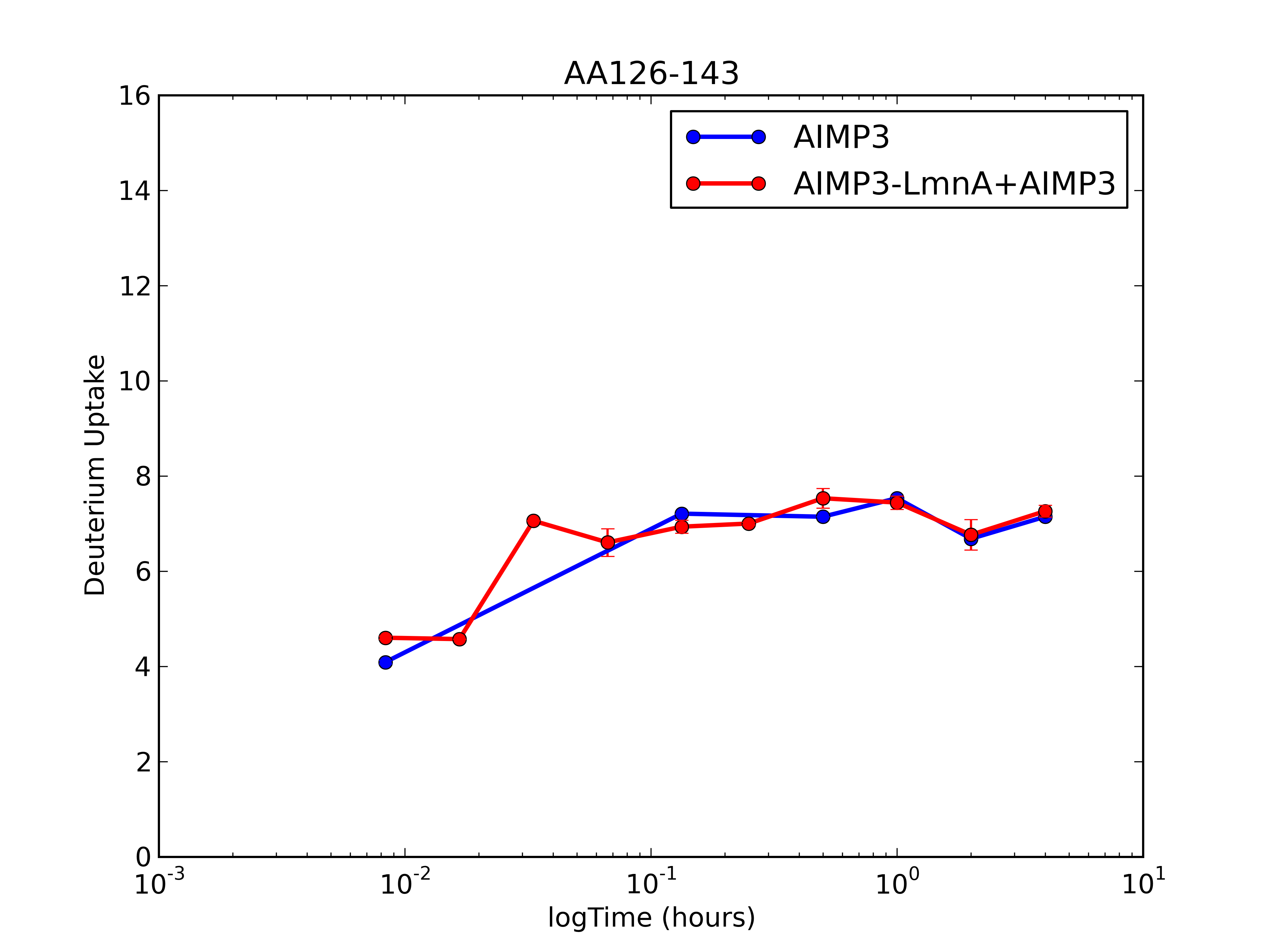

Supplement: S2 File — (ZIP) [file pone.0181869.s004.zip › logfigure-LmnA-scale/AA126-143_charge_2_mz890.3.csv.csv.png]

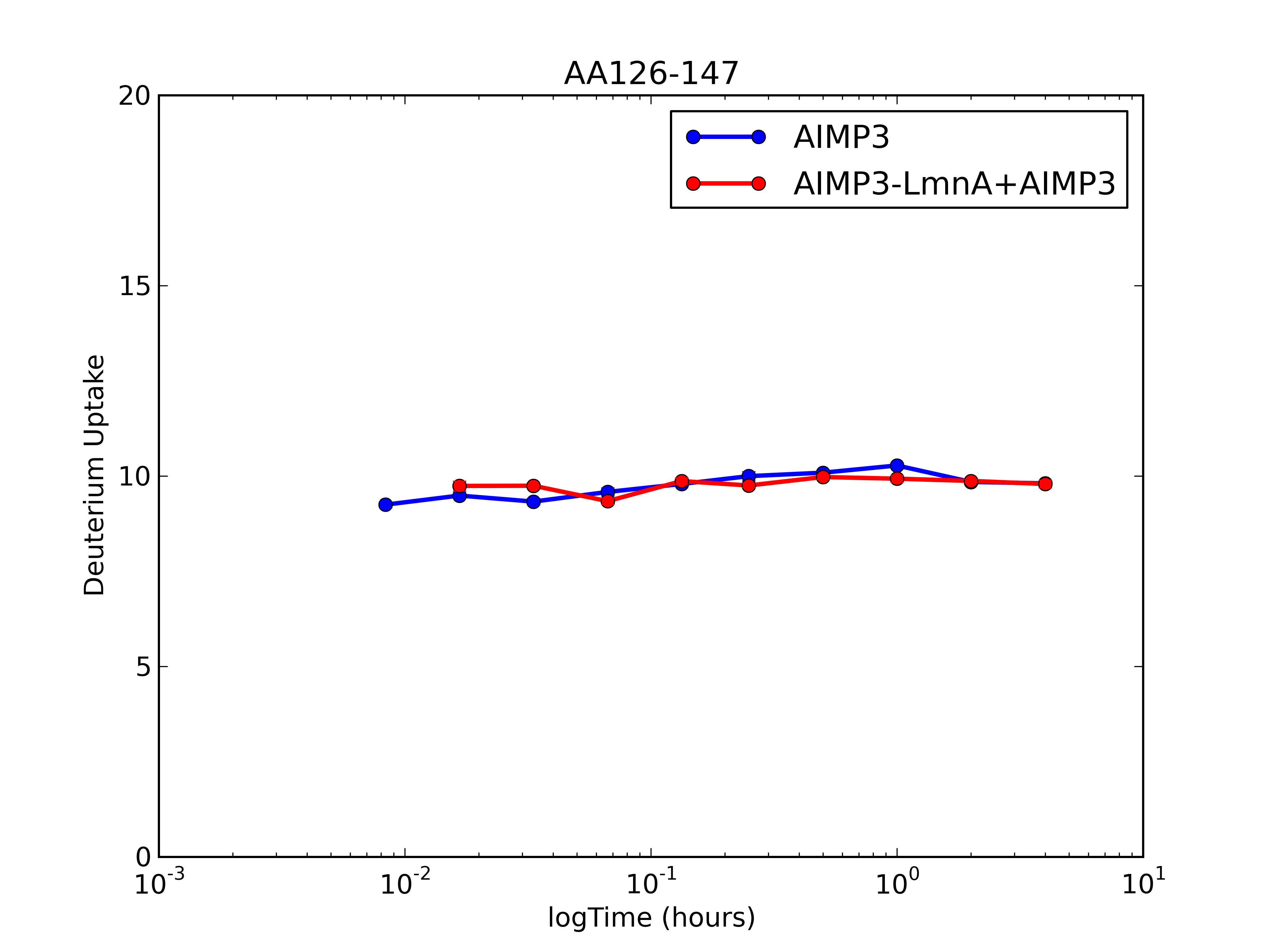

Supplement: S2 File — (ZIP) [file pone.0181869.s004.zip › logfigure-LmnA-scale/AA126-147_charge_3_mz764.6.csv.csv.png]

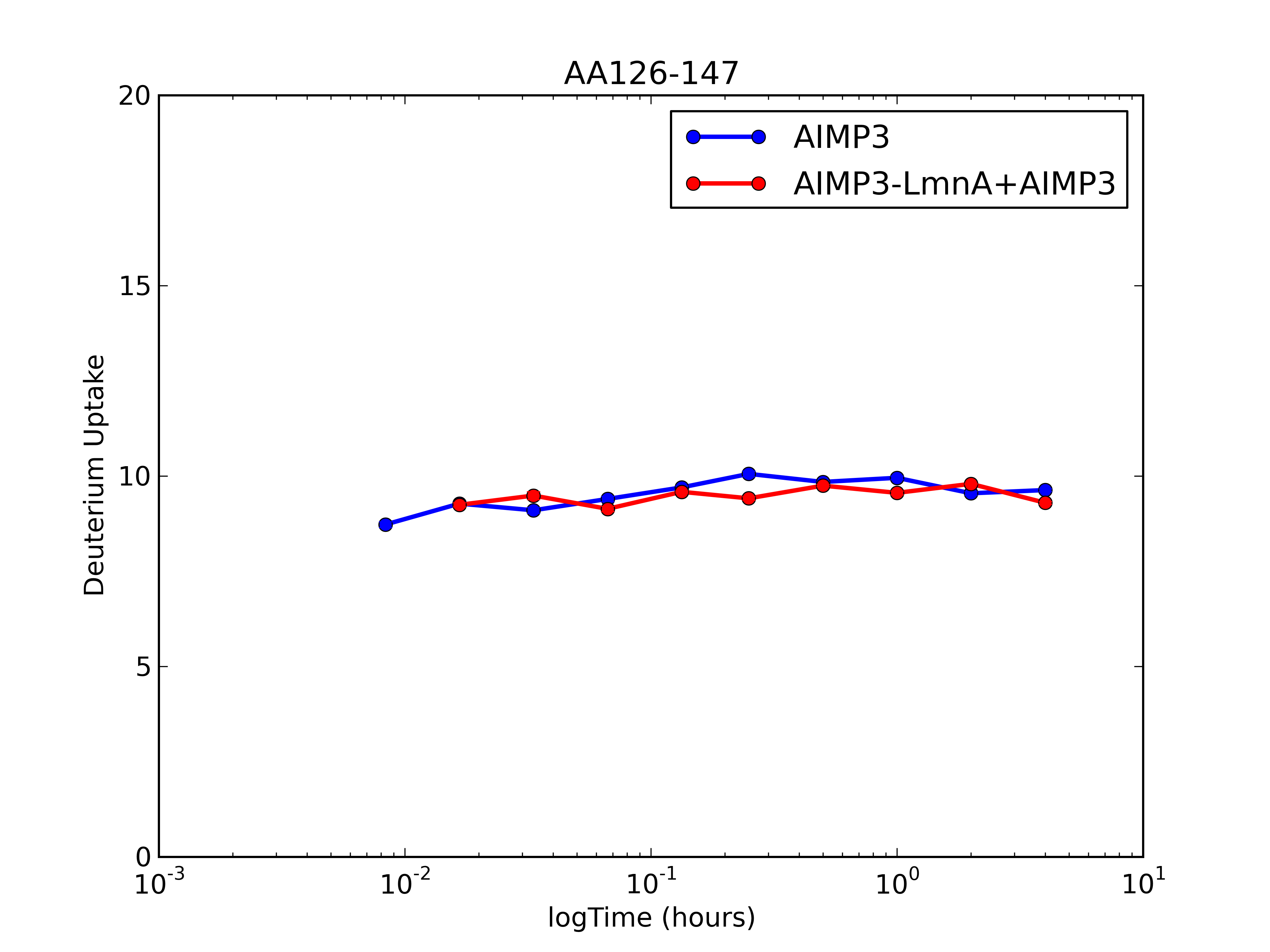

Supplement: S2 File — (ZIP) [file pone.0181869.s004.zip › logfigure-LmnA-scale/AA126-147_charge_4_mz573.7.csv.csv.png]

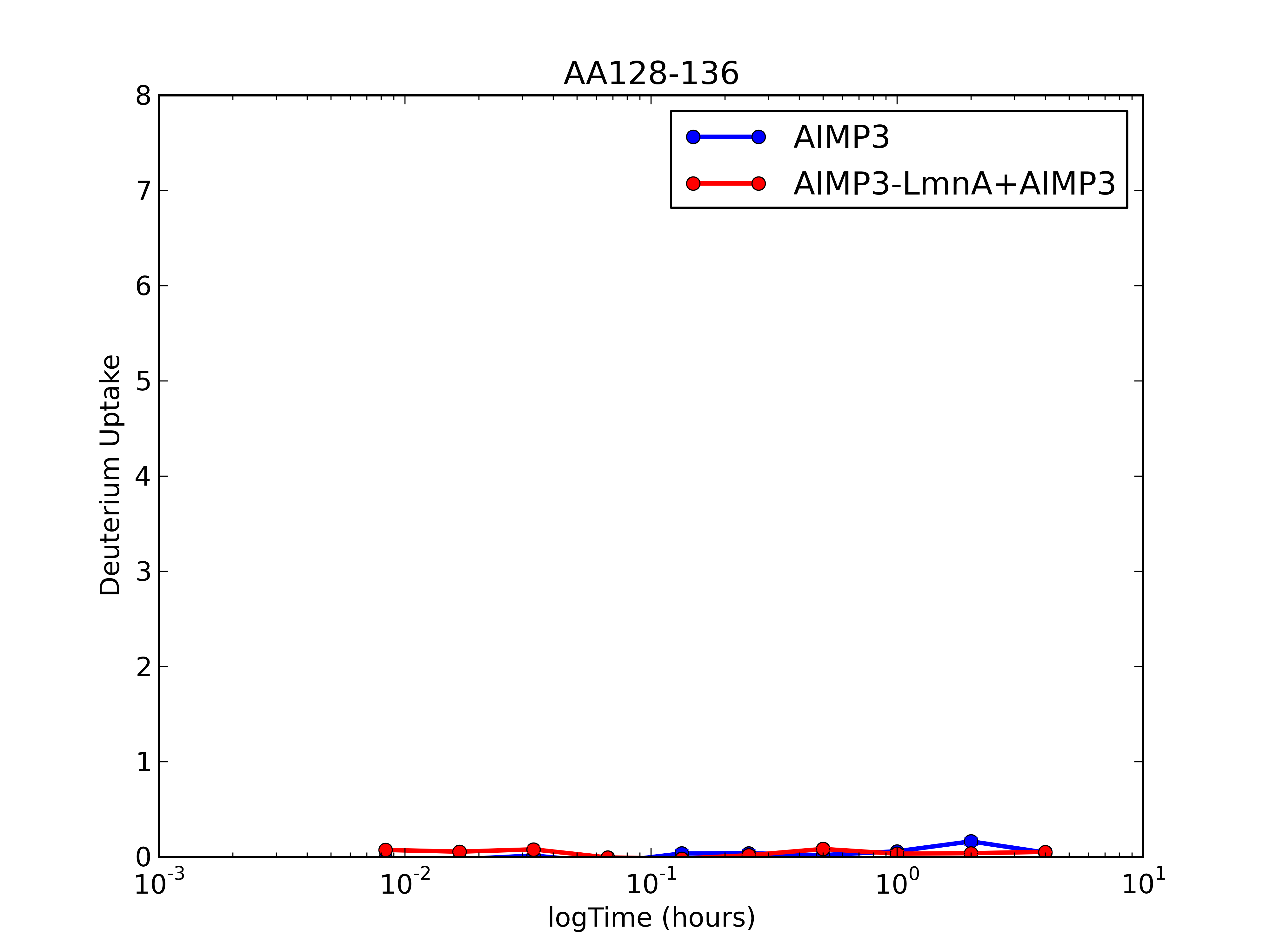

Supplement: S2 File — (ZIP) [file pone.0181869.s004.zip › logfigure-LmnA-scale/AA128-136_charge_2_mz424.6.csv.csv.png]

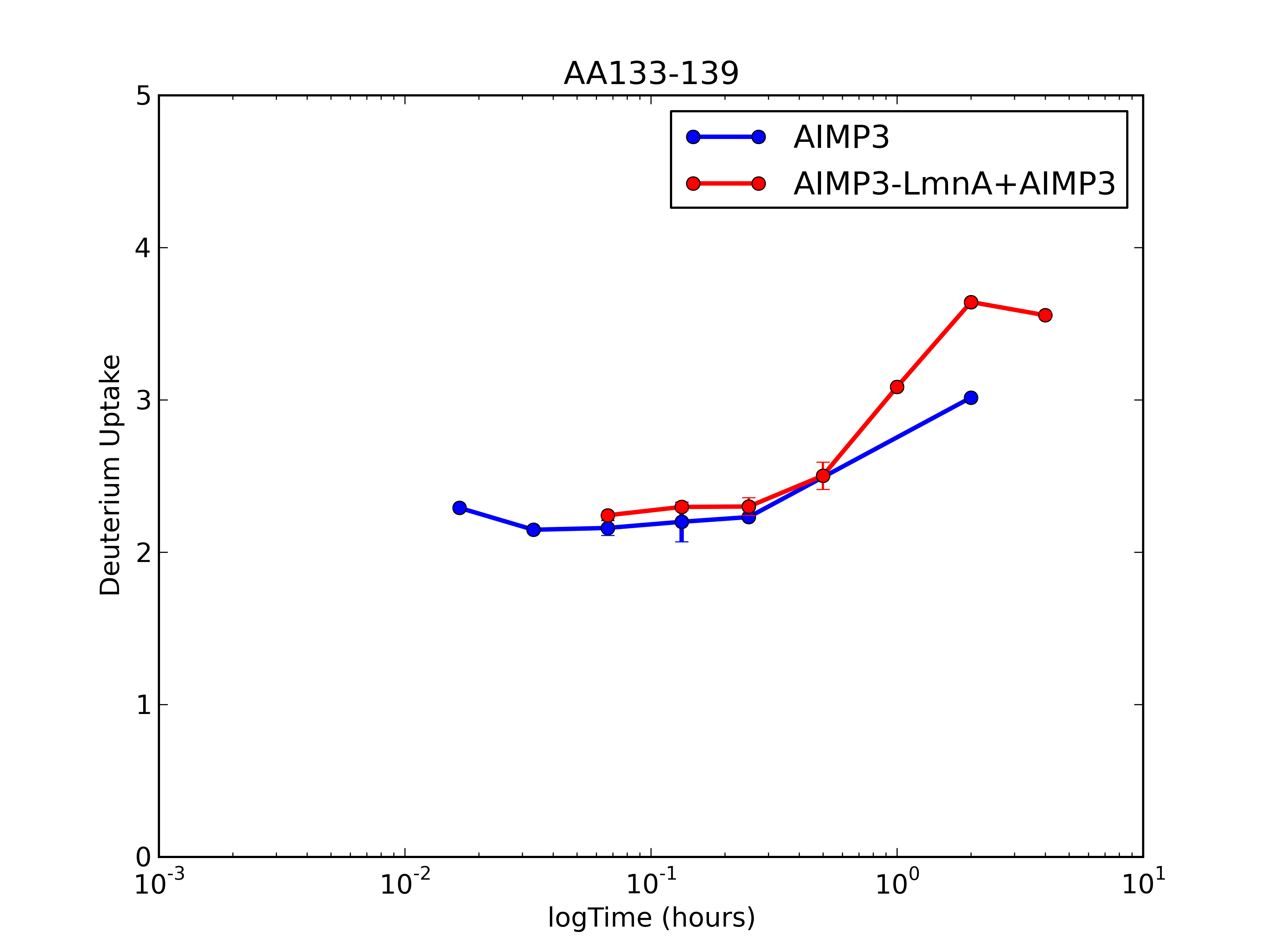

Supplement: S2 File — (ZIP) [file pone.0181869.s004.zip › logfigure-LmnA-scale/AA133-139_charge_1_mz652.2.csv.csv.png]

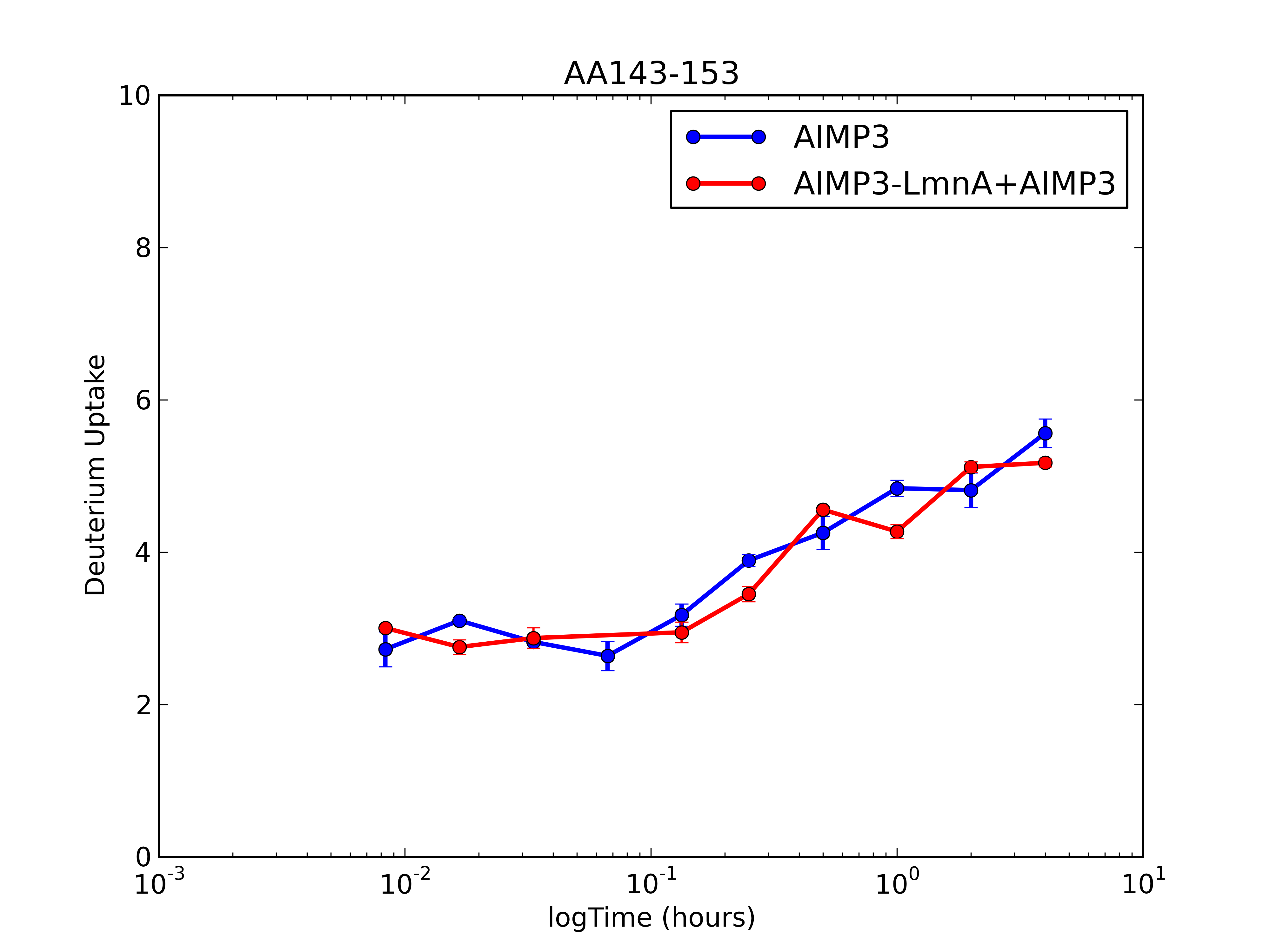

Supplement: S2 File — (ZIP) [file pone.0181869.s004.zip › logfigure-LmnA-scale/AA143-153_charge_3_mz407.2.csv.csv.png]

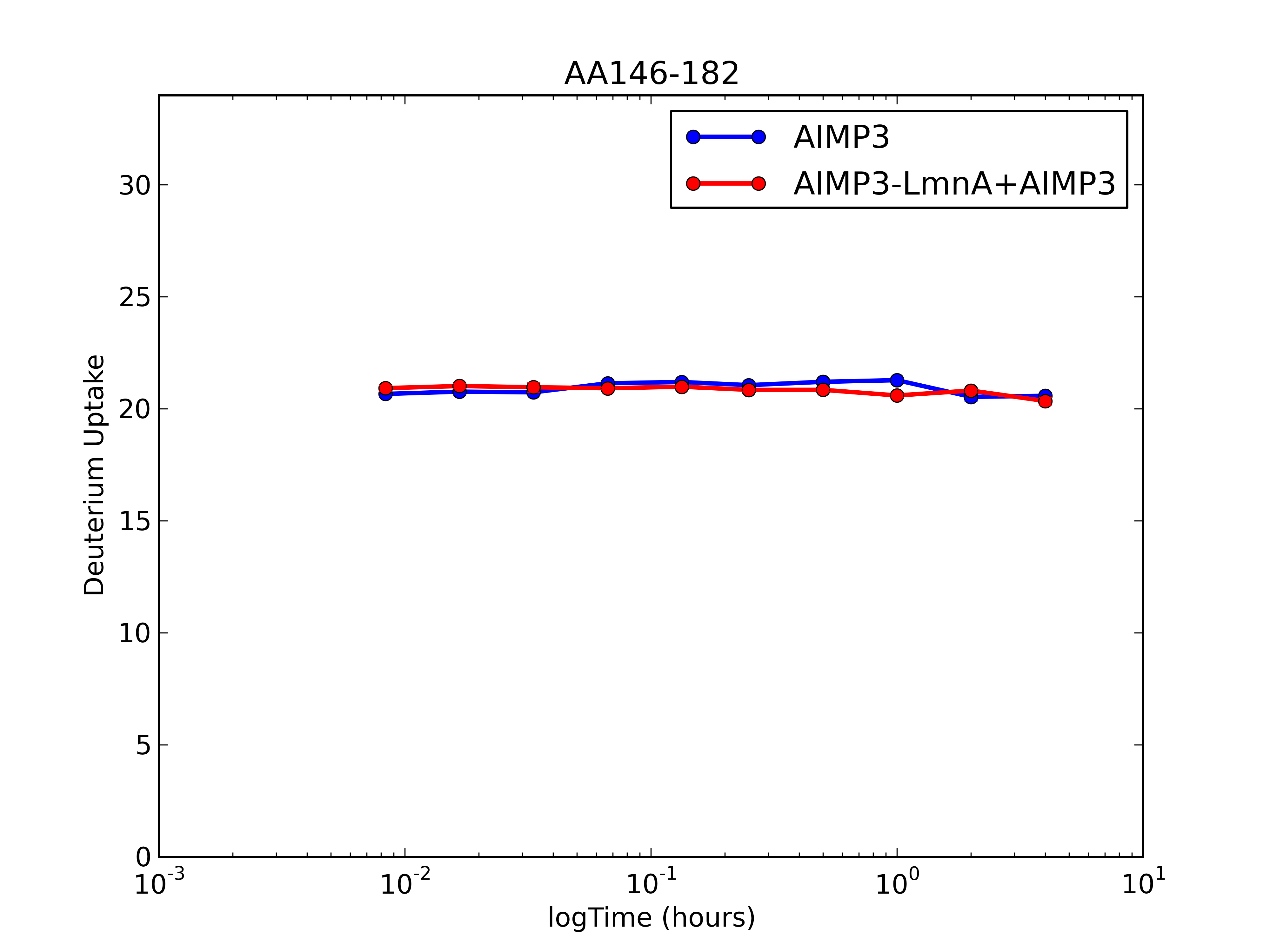

Supplement: S2 File — (ZIP) [file pone.0181869.s004.zip › logfigure-LmnA-scale/AA146-182_charge_3_mz1109.1.csv.csv.png]

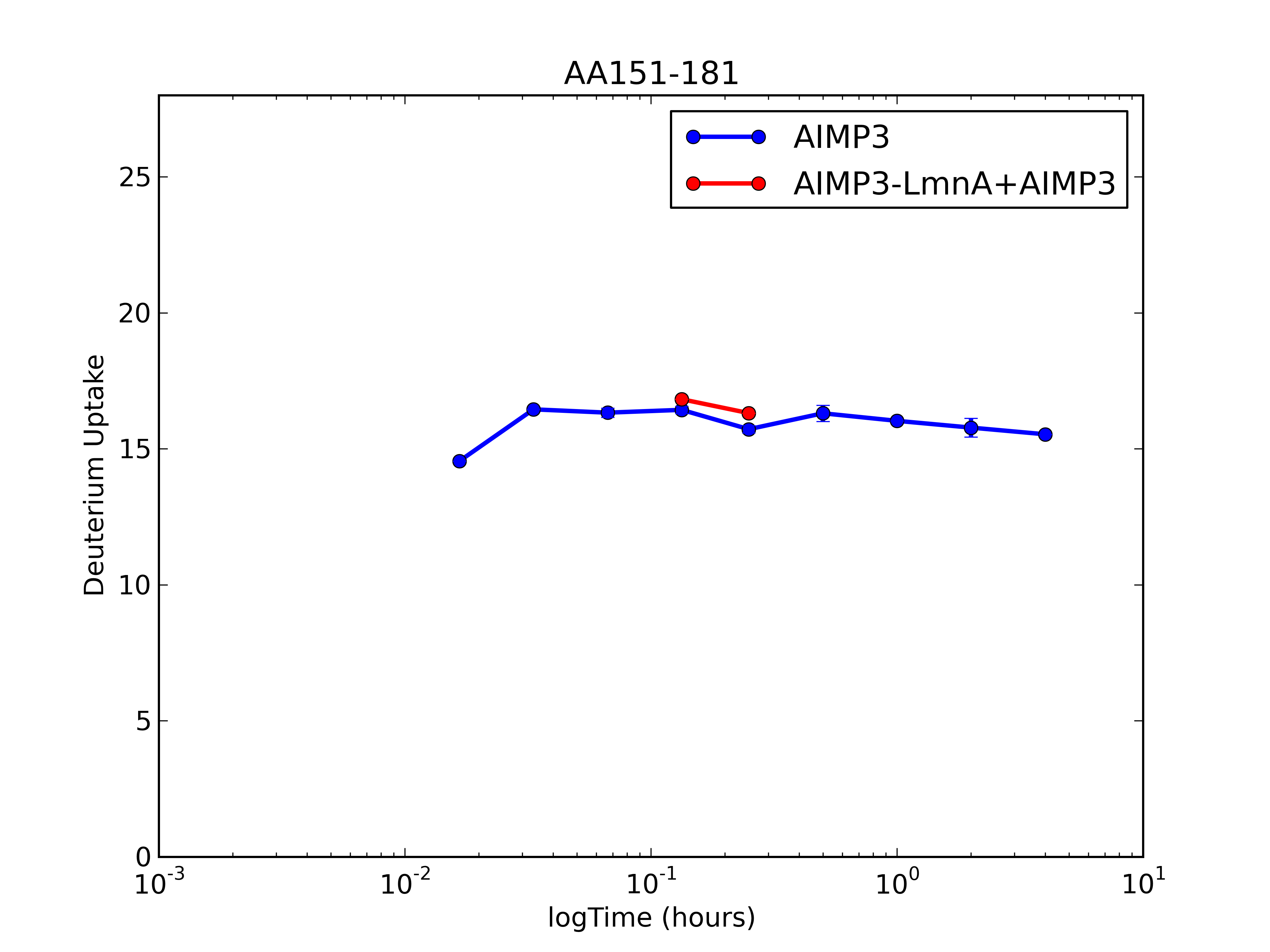

Supplement: S2 File — (ZIP) [file pone.0181869.s004.zip › logfigure-LmnA-scale/AA151-181_charge_3_mz894.7.csv.csv.png]

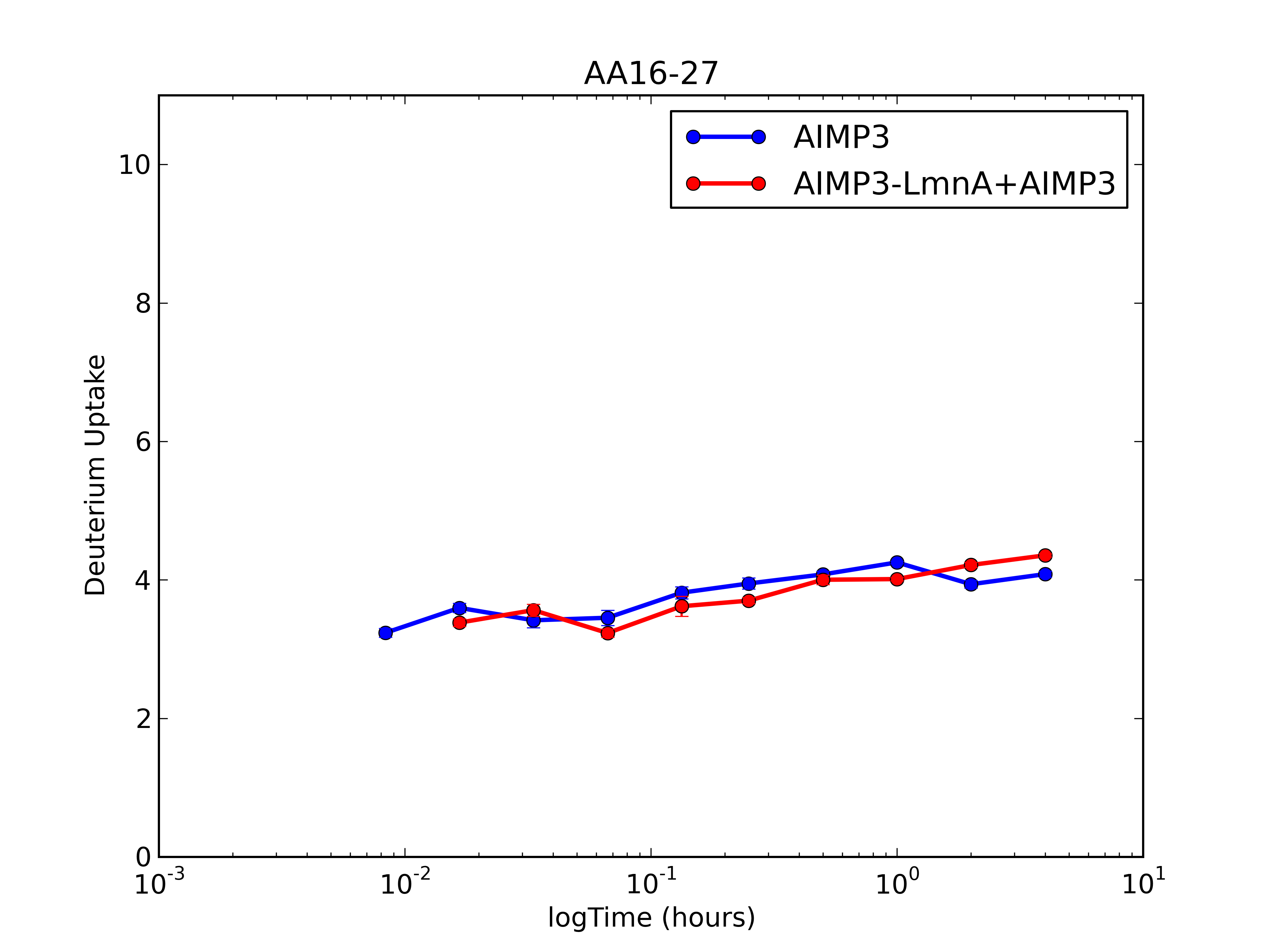

Supplement: S2 File — (ZIP) [file pone.0181869.s004.zip › logfigure-LmnA-scale/AA16-27_charge_3_mz469.2.csv.csv.png]

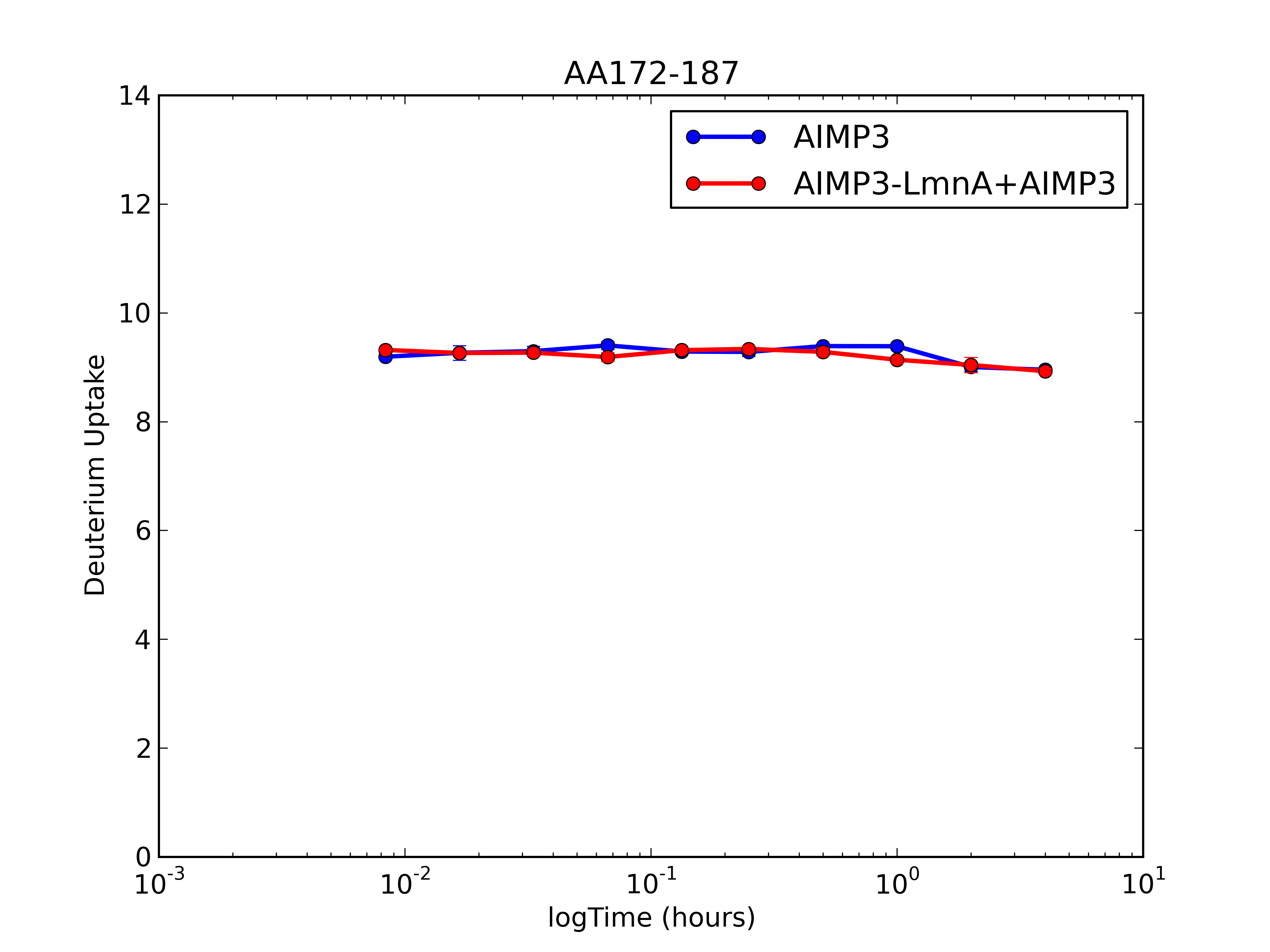

Supplement: S2 File — (ZIP) [file pone.0181869.s004.zip › logfigure-LmnA-scale/AA172-187_charge_2_mz746.8.csv.csv.png]

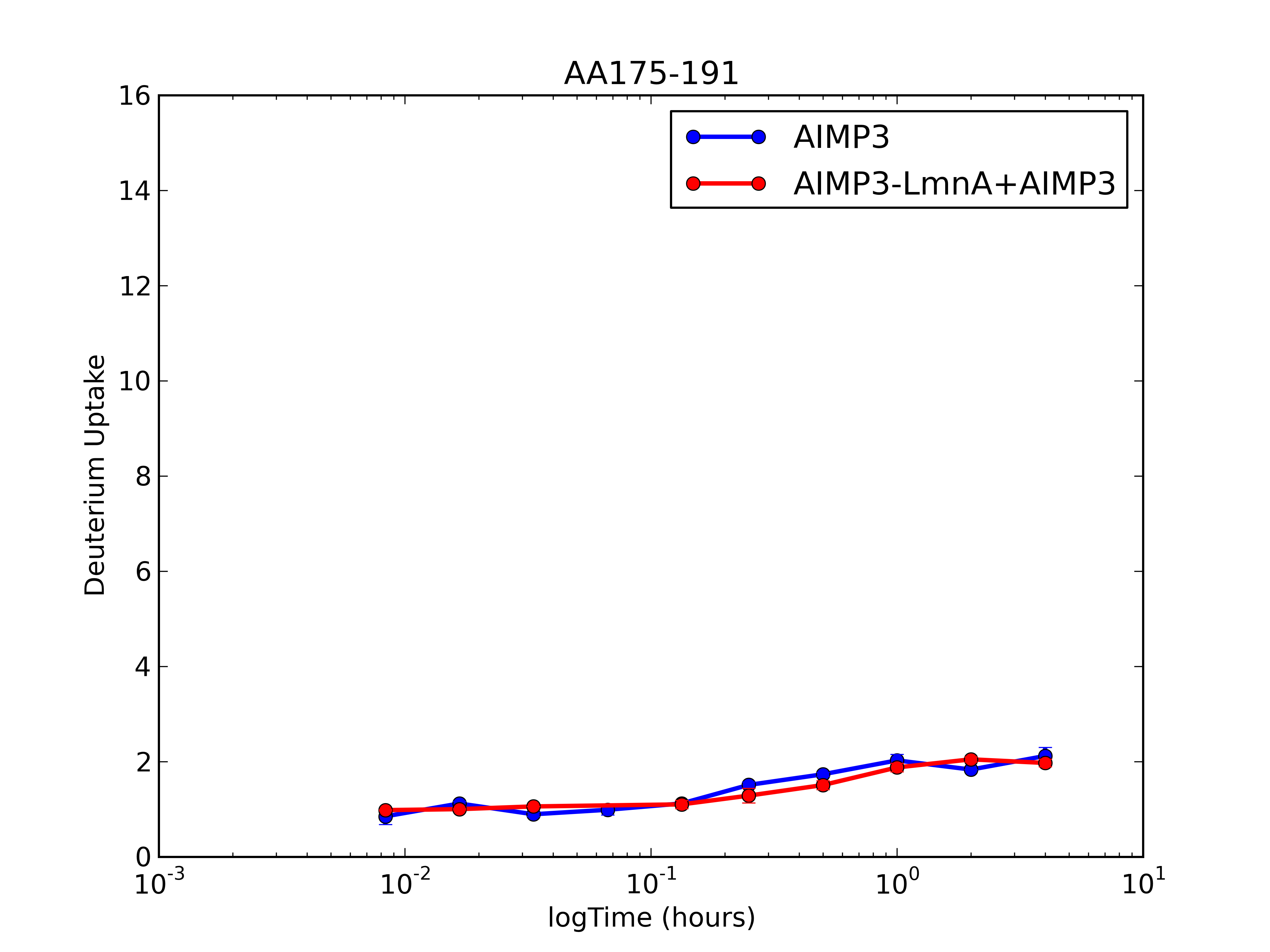

Supplement: S2 File — (ZIP) [file pone.0181869.s004.zip › logfigure-LmnA-scale/AA175-191_charge_2_mz859.9.csv.csv.png]

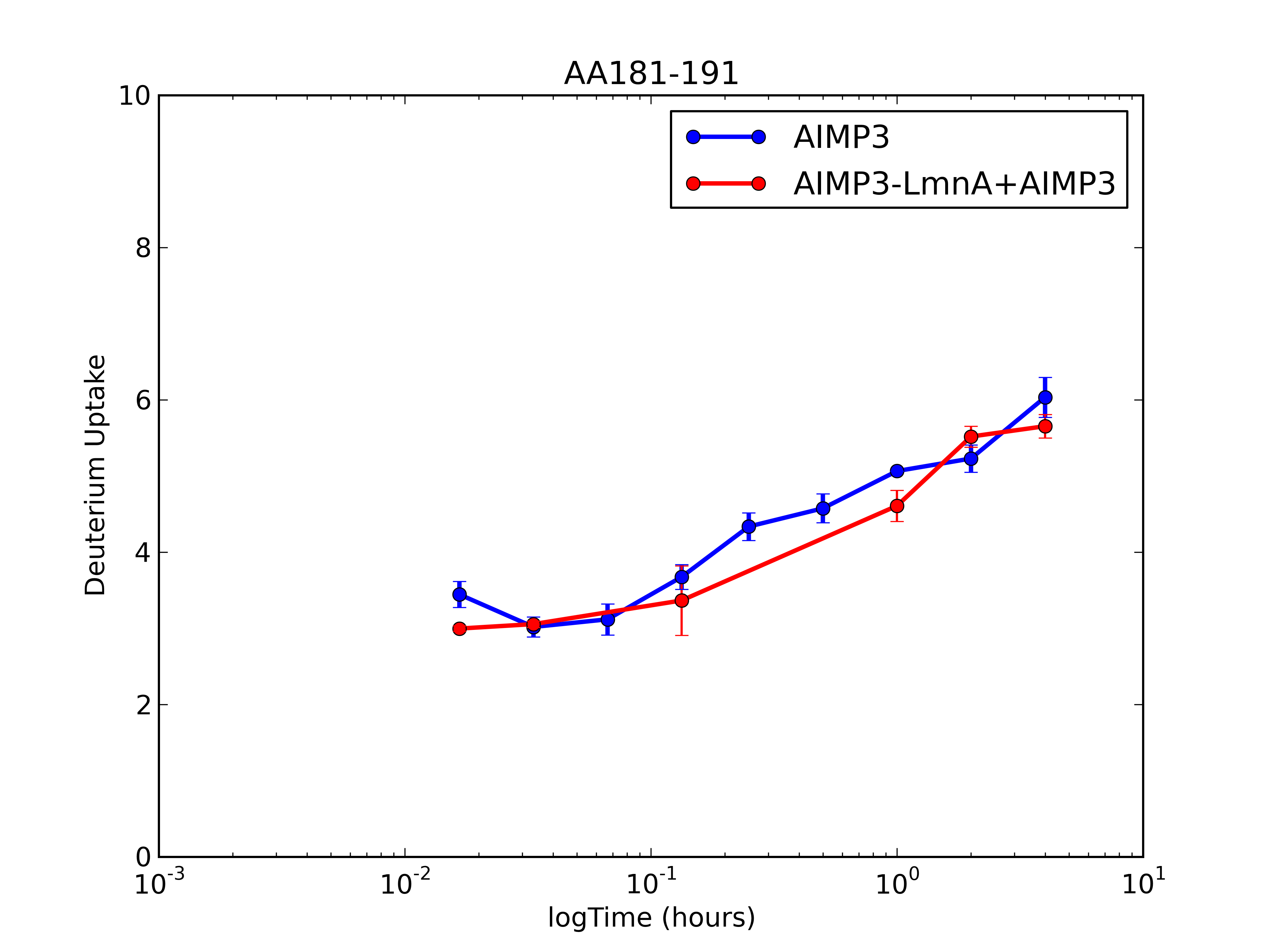

Supplement: S2 File — (ZIP) [file pone.0181869.s004.zip › logfigure-LmnA-scale/AA181-191_charge_2_mz621.8.csv.csv.png]

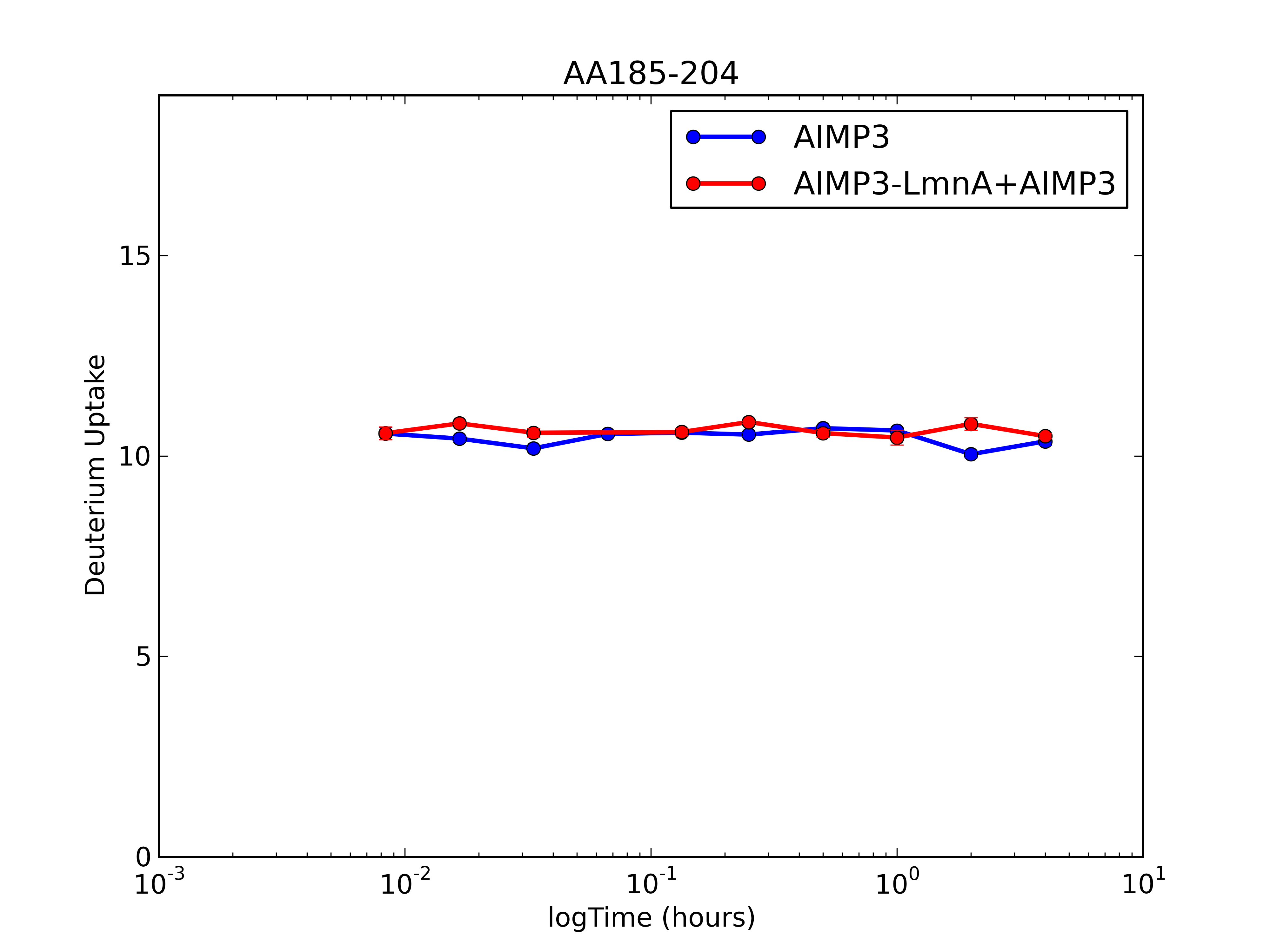

Supplement: S2 File — (ZIP) [file pone.0181869.s004.zip › logfigure-LmnA-scale/AA185-204_charge_3_mz658.3.csv.csv.png]

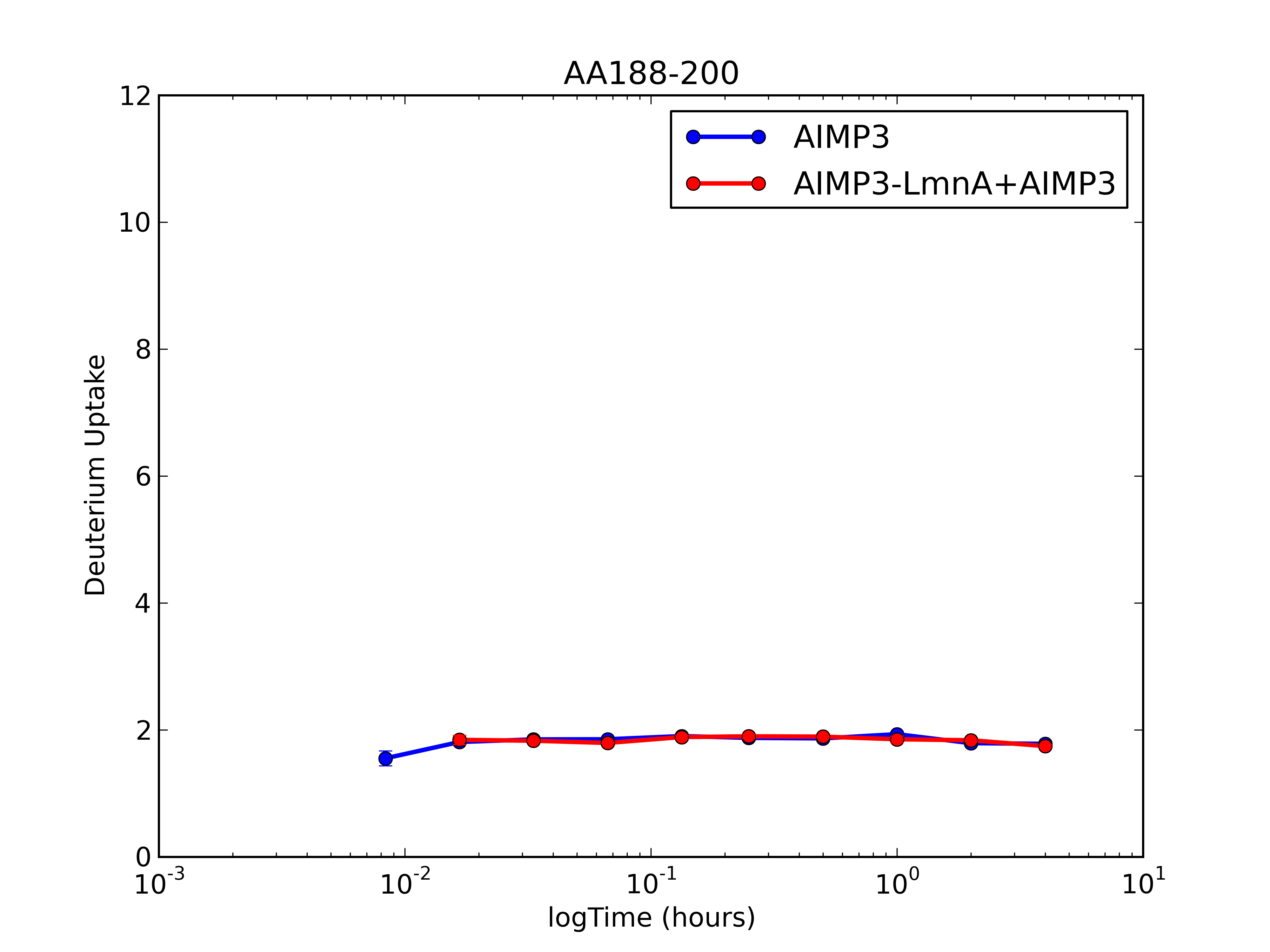

Supplement: S2 File — (ZIP) [file pone.0181869.s004.zip › logfigure-LmnA-scale/AA188-200_charge_2_mz609.2.csv.csv.png]

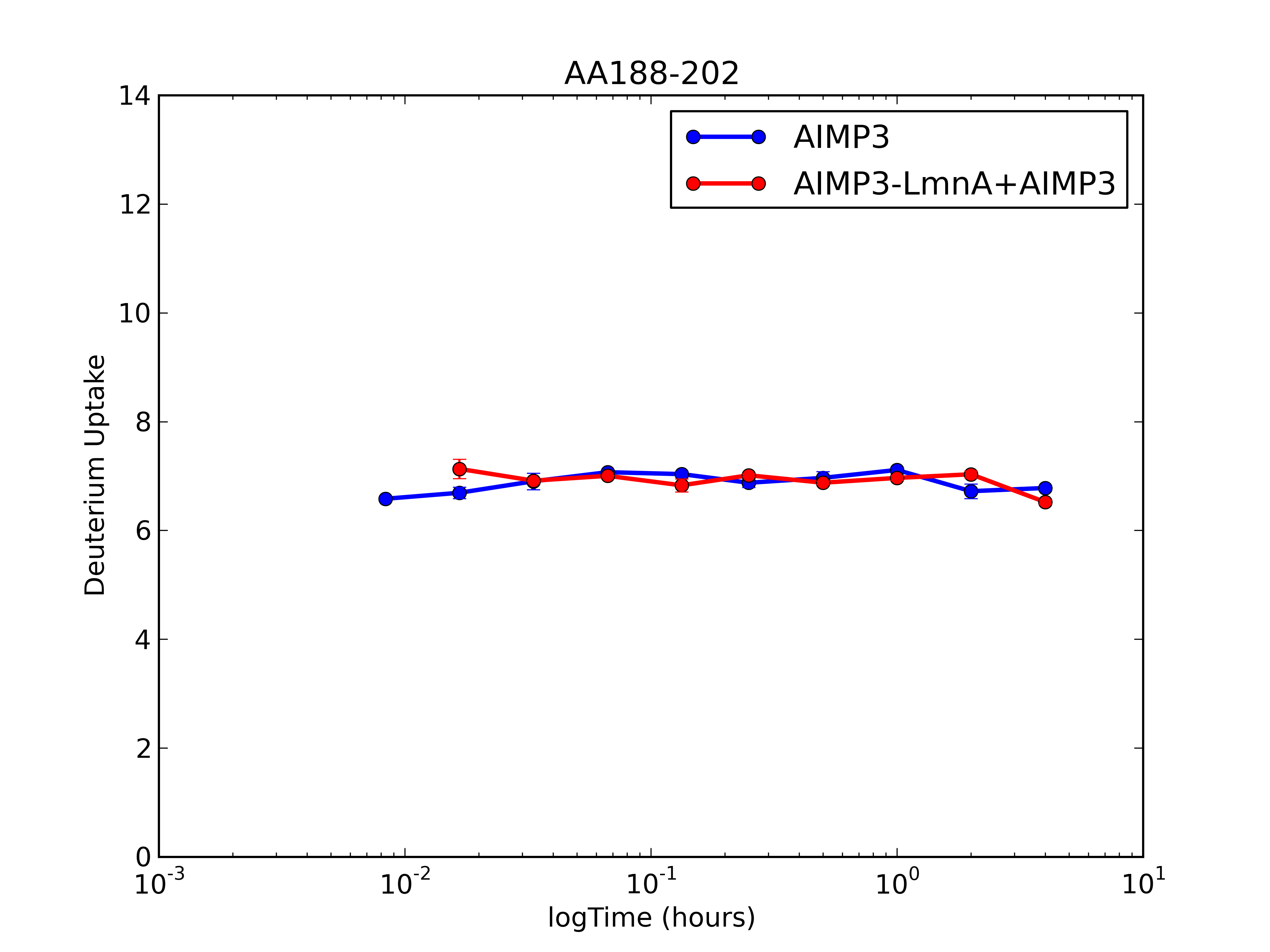

Supplement: S2 File — (ZIP) [file pone.0181869.s004.zip › logfigure-LmnA-scale/AA188-202_charge_2_mz695.3.csv.csv.png]

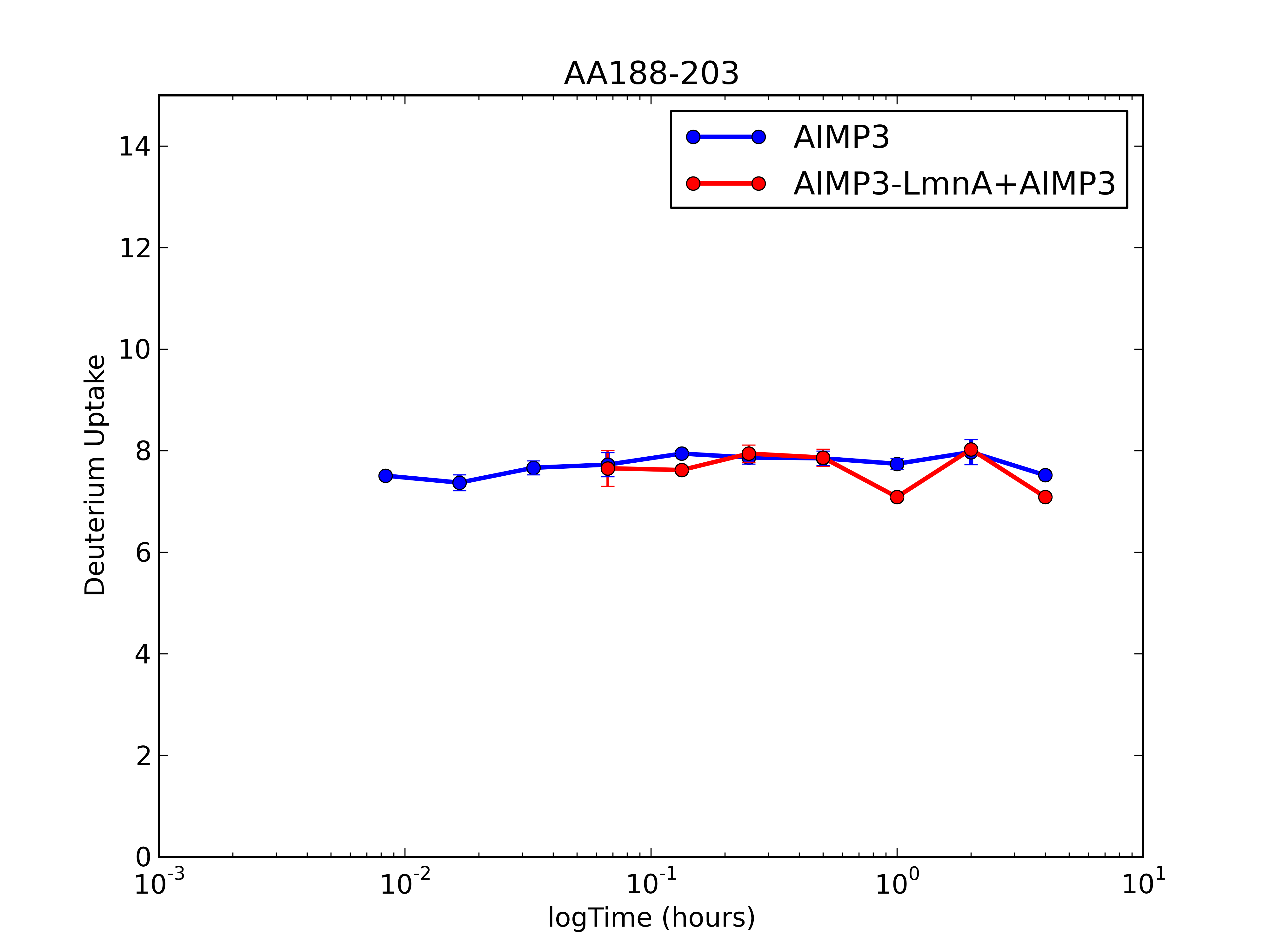

Supplement: S2 File — (ZIP) [file pone.0181869.s004.zip › logfigure-LmnA-scale/AA188-203_charge_2_mz752.3.csv.csv.png]

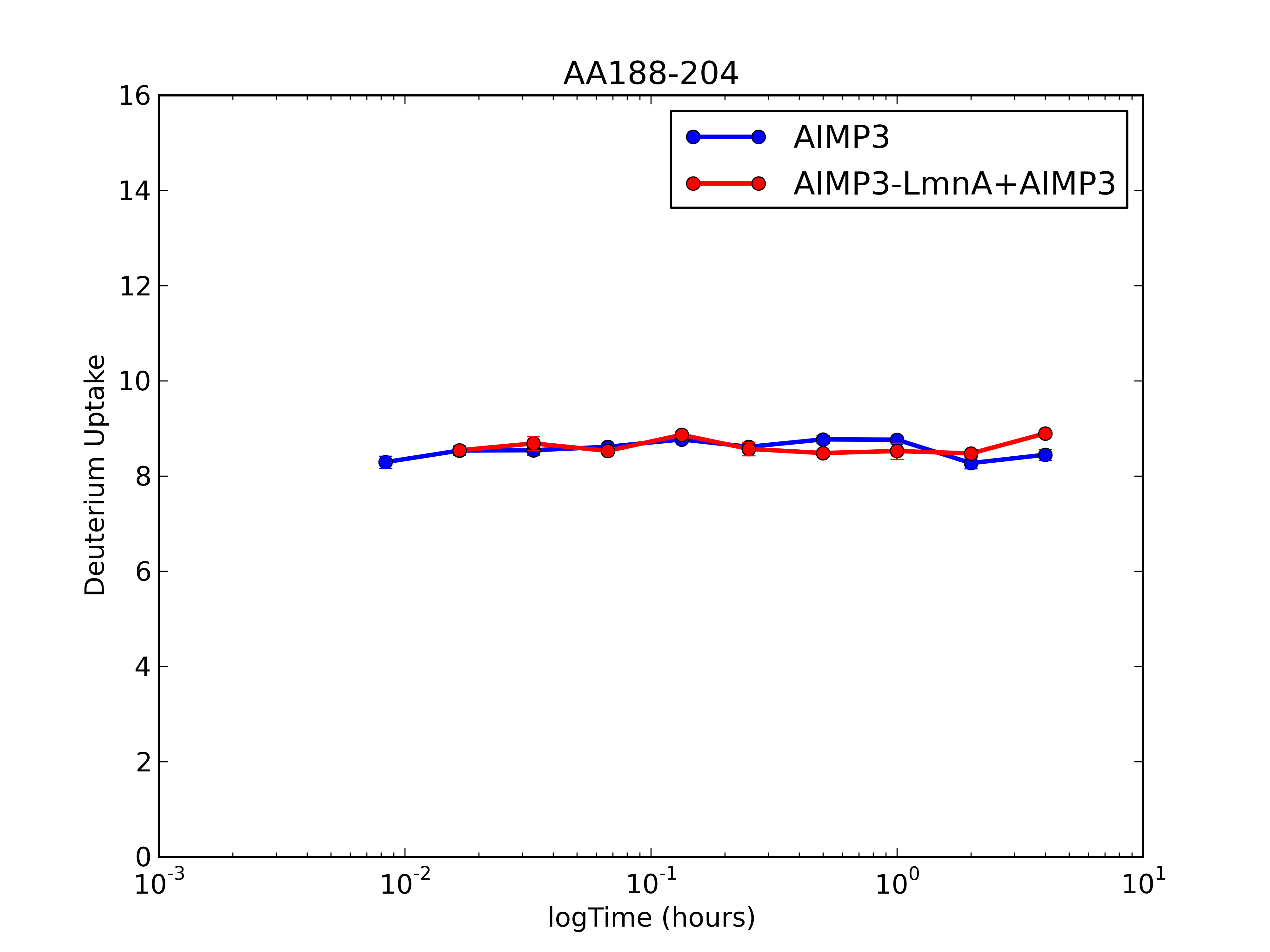

Supplement: S2 File — (ZIP) [file pone.0181869.s004.zip › logfigure-LmnA-scale/AA188-204_charge_2_mz808.8.csv.csv.png]

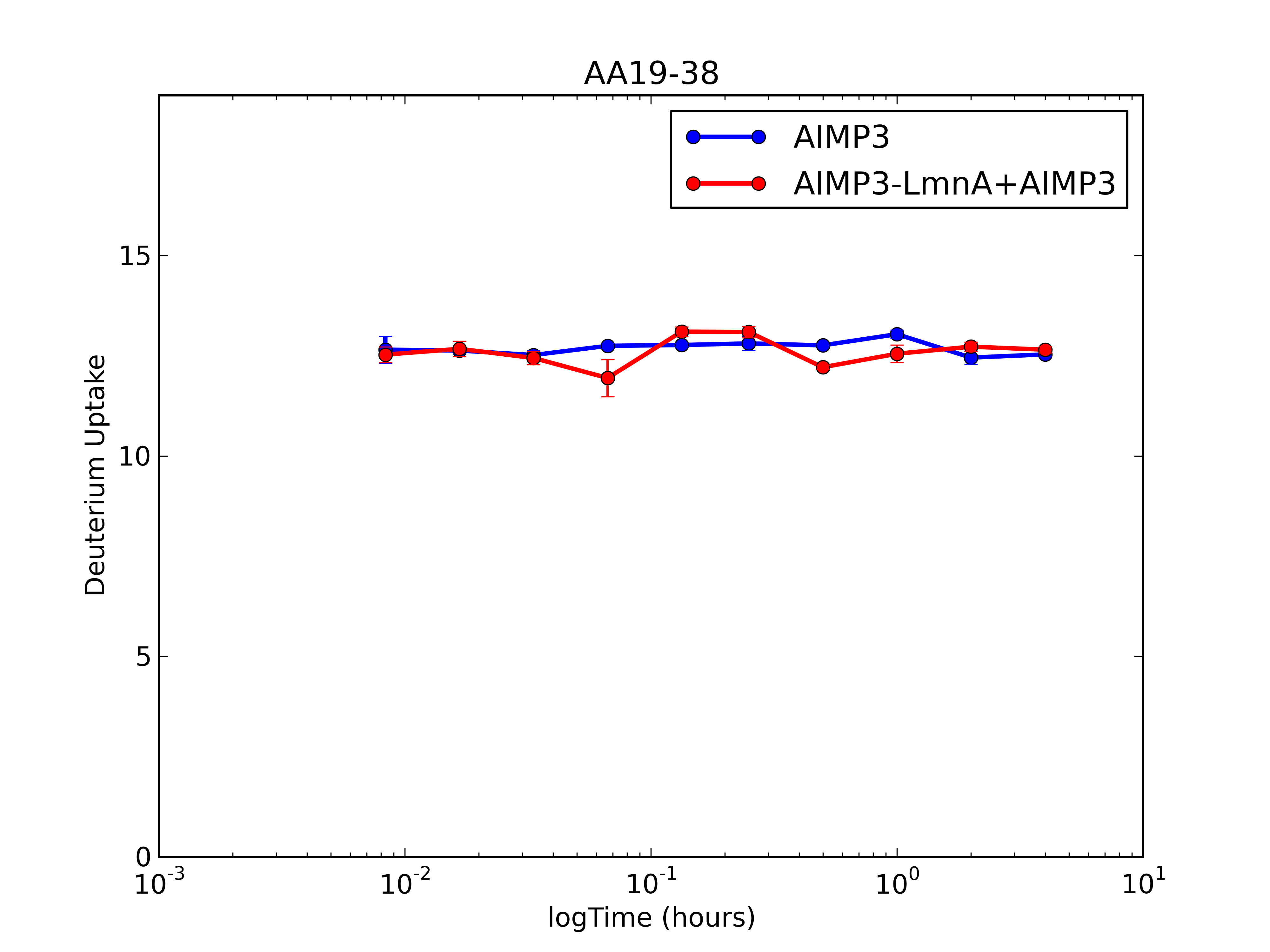

Supplement: S2 File — (ZIP) [file pone.0181869.s004.zip › logfigure-LmnA-scale/AA19-38_charge_2_mz1112.0.csv.csv.png]

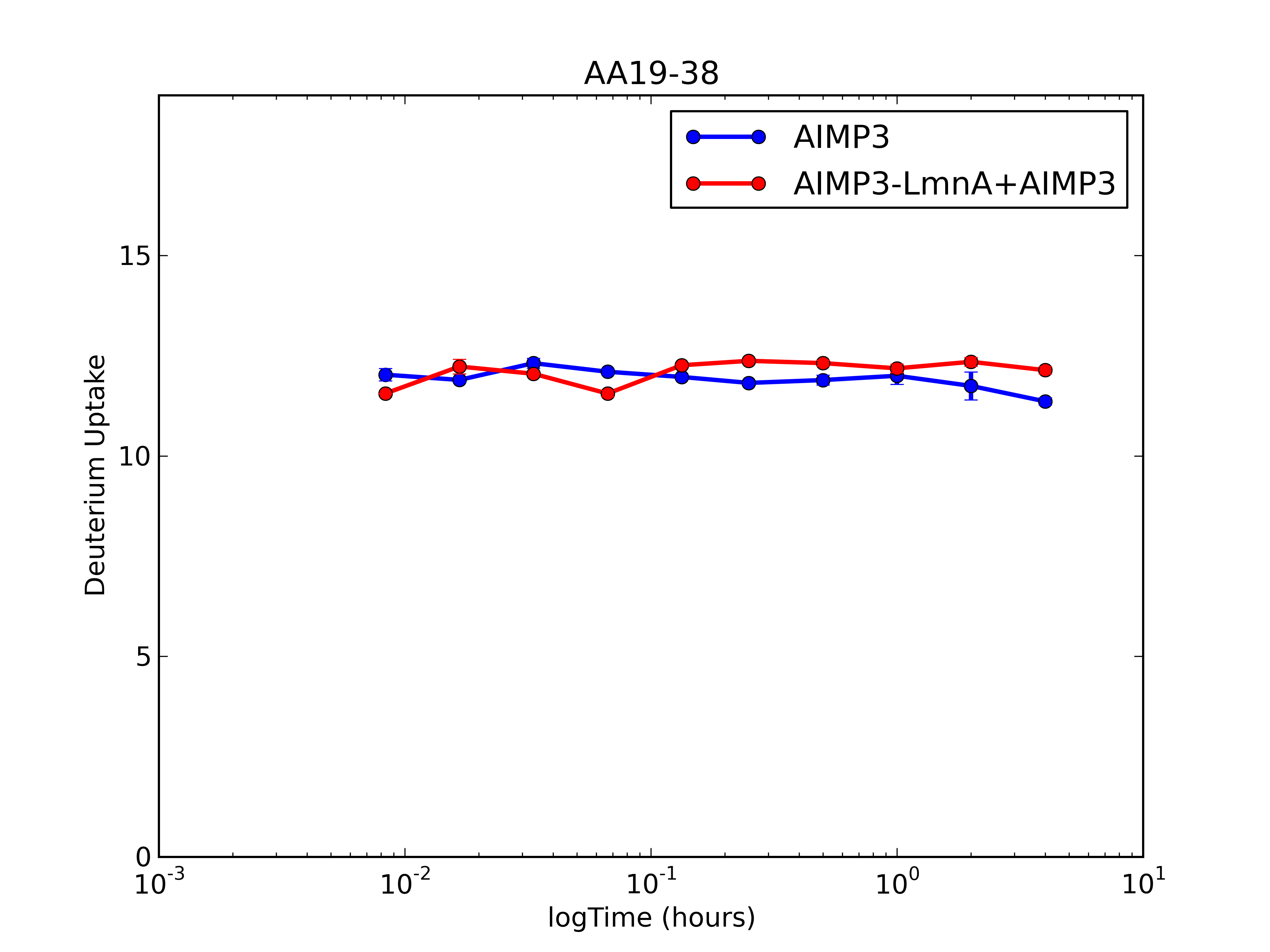

Supplement: S2 File — (ZIP) [file pone.0181869.s004.zip › logfigure-LmnA-scale/AA19-38_charge_3_mz741.6.csv.csv.png]

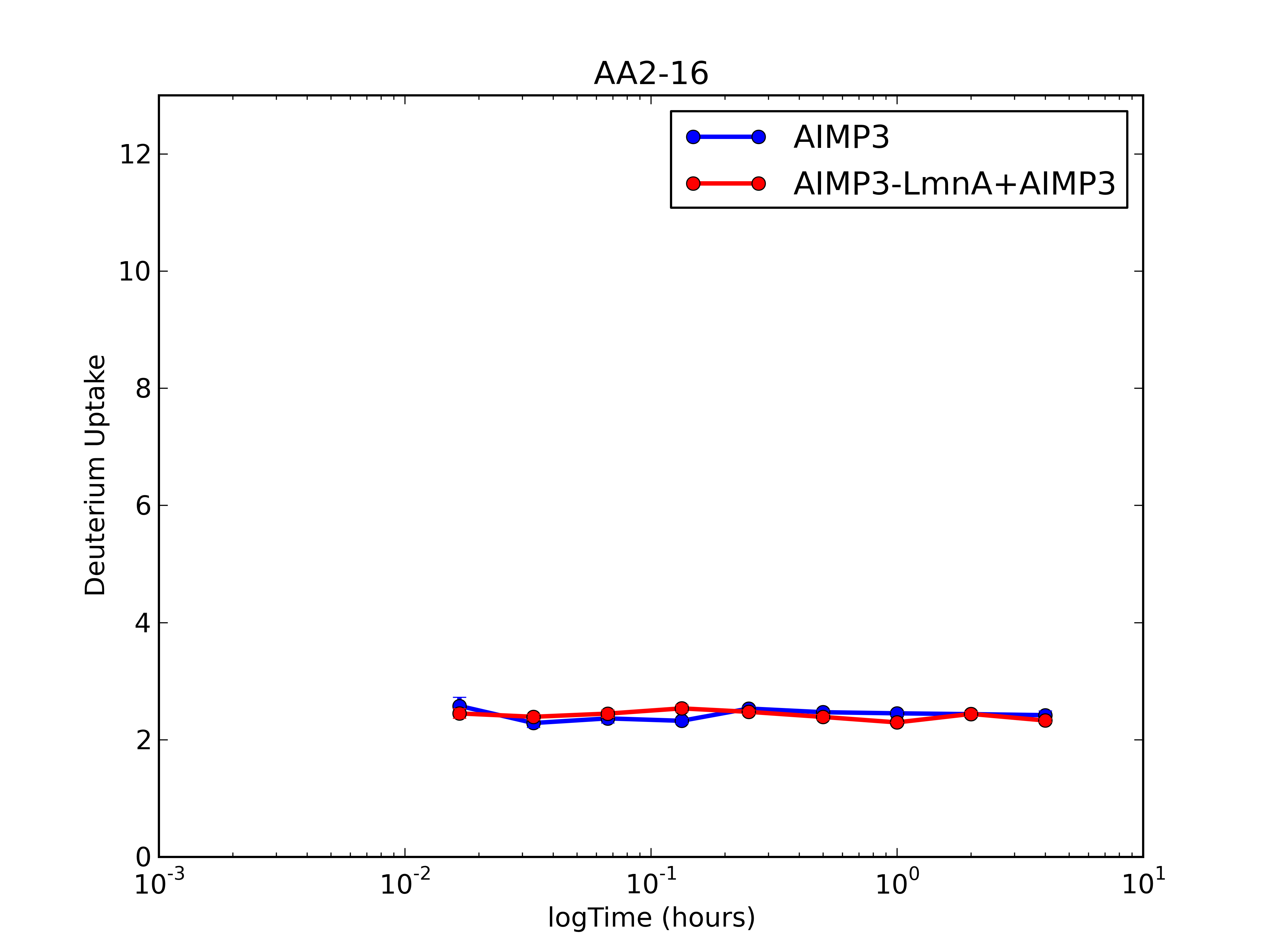

Supplement: S2 File — (ZIP) [file pone.0181869.s004.zip › logfigure-LmnA-scale/AA2-16_charge_3_mz613.6.csv.csv.png]

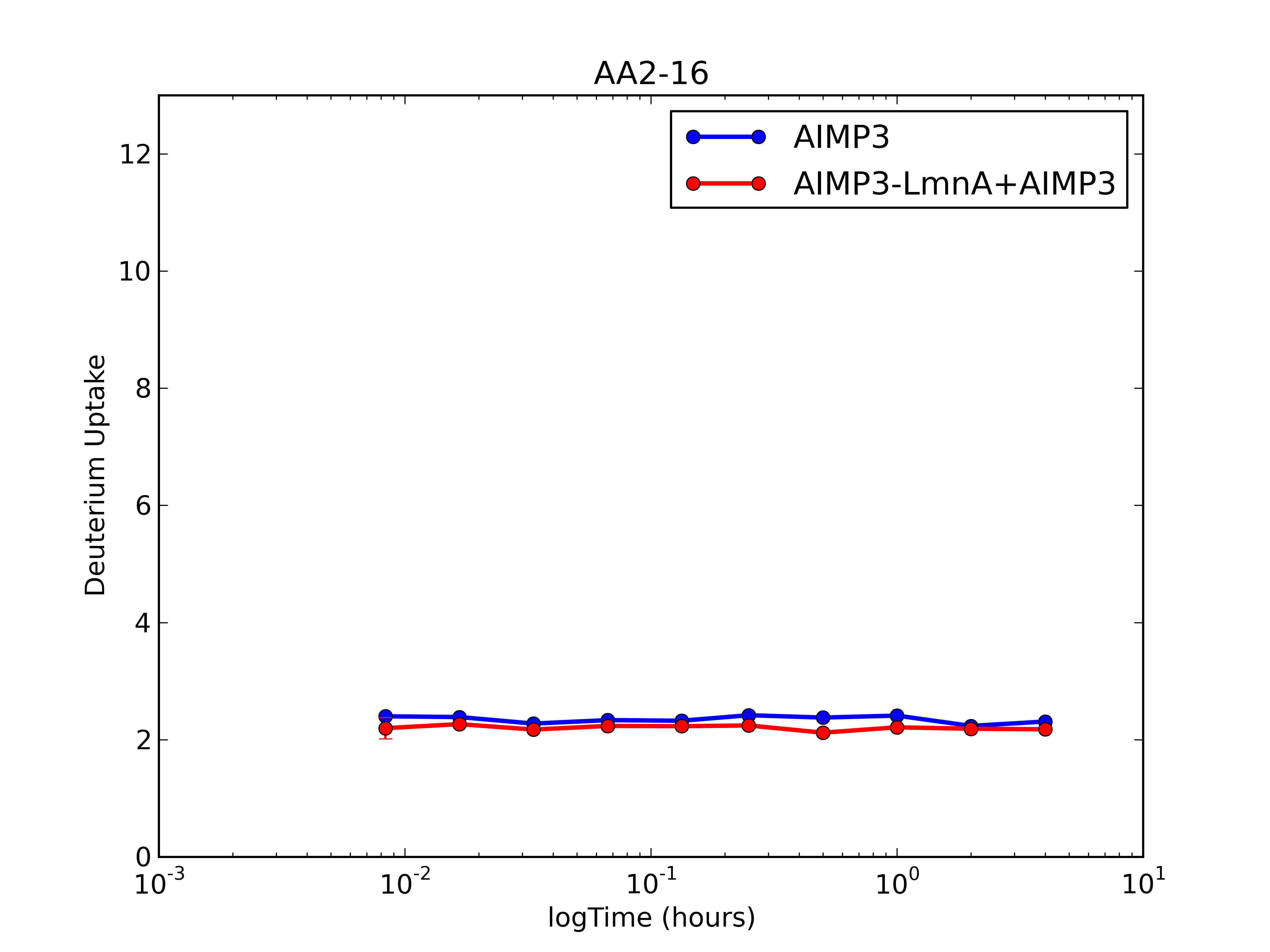

Supplement: S2 File — (ZIP) [file pone.0181869.s004.zip › logfigure-LmnA-scale/AA2-16_charge_4_mz460.4.csv.csv.png]

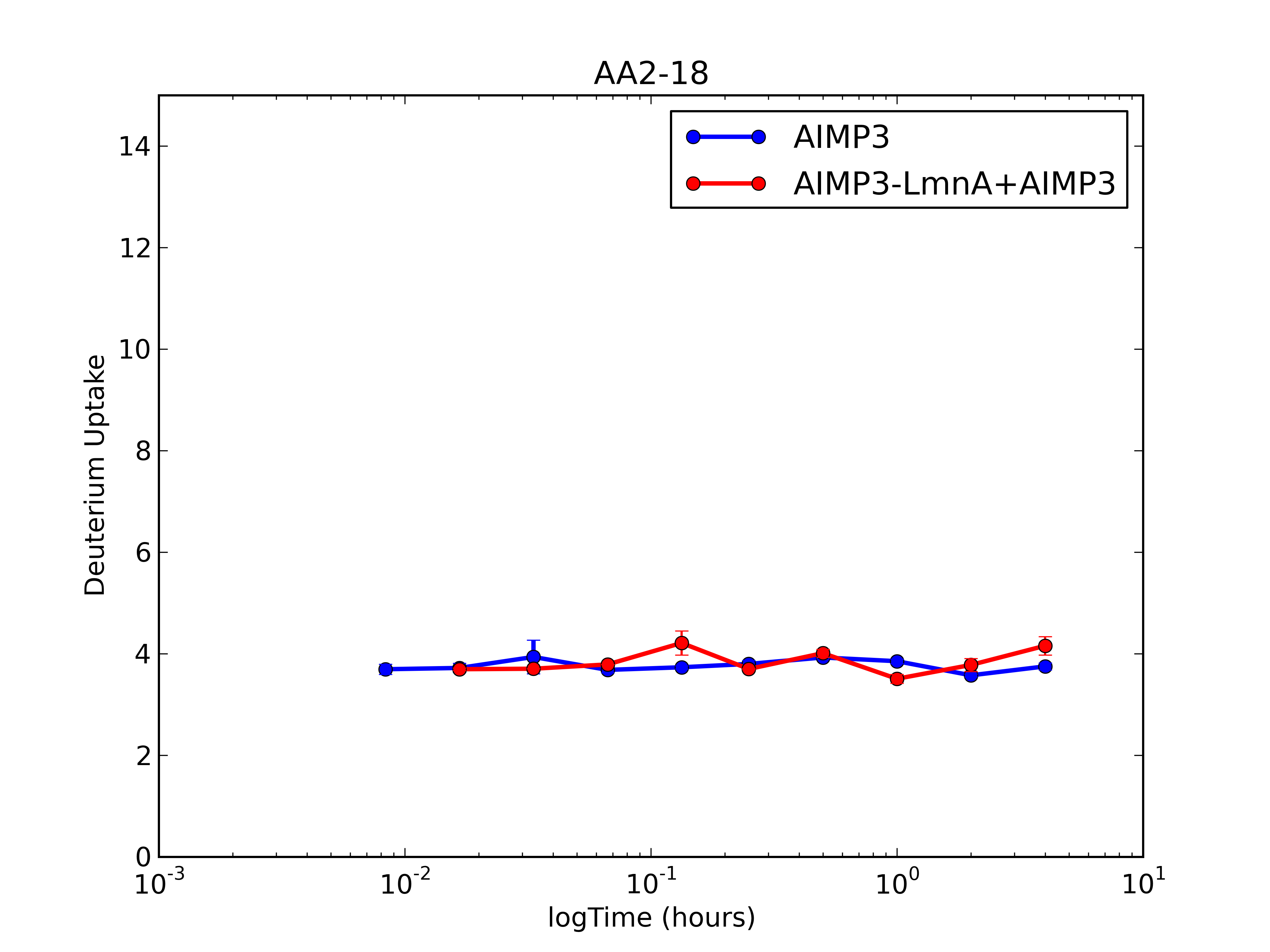

Supplement: S2 File — (ZIP) [file pone.0181869.s004.zip › logfigure-LmnA-scale/AA2-18_charge_3_mz699.3.csv.csv.png]

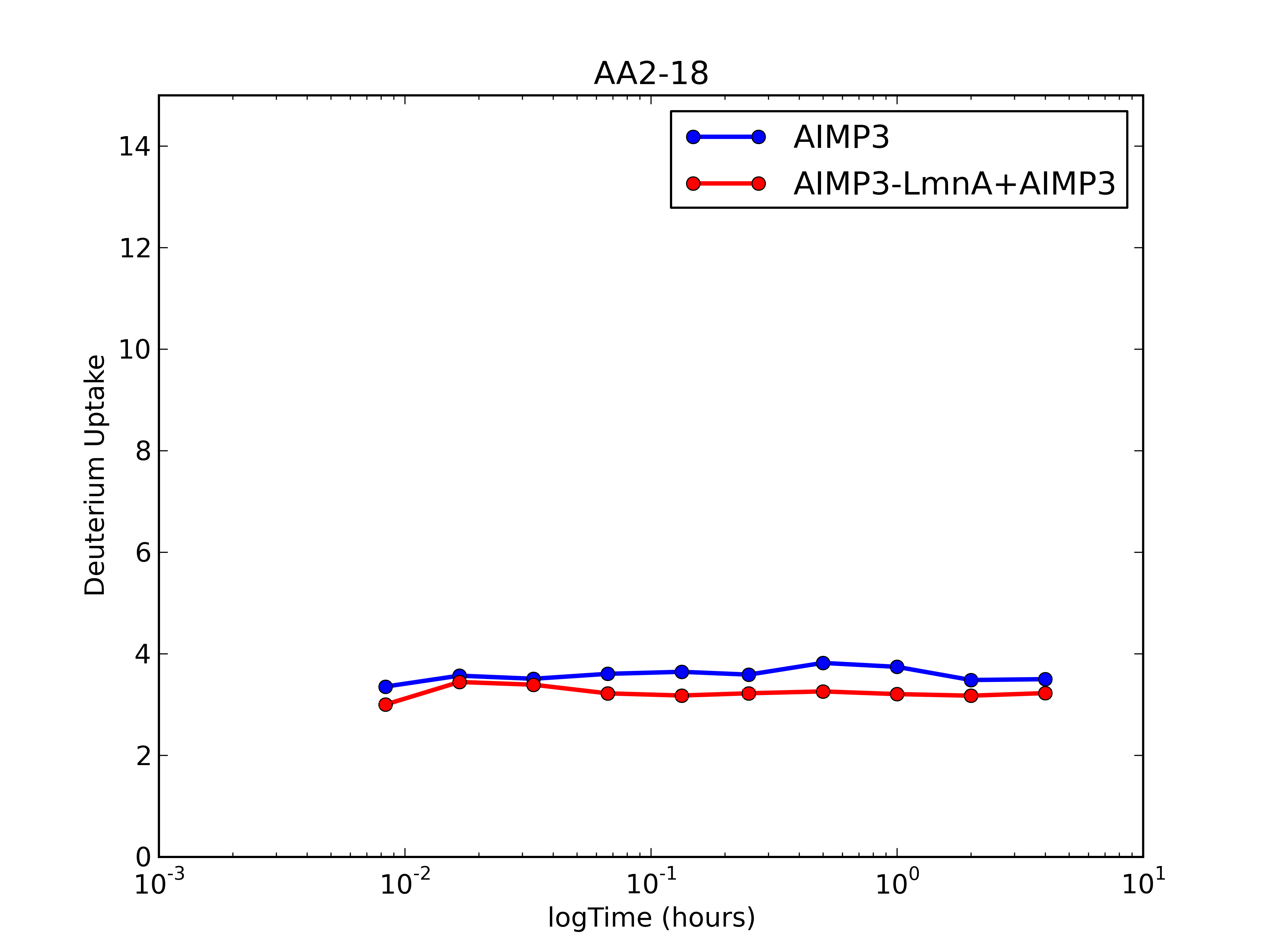

Supplement: S2 File — (ZIP) [file pone.0181869.s004.zip › logfigure-LmnA-scale/AA2-18_charge_4_mz524.7.csv.csv.png]

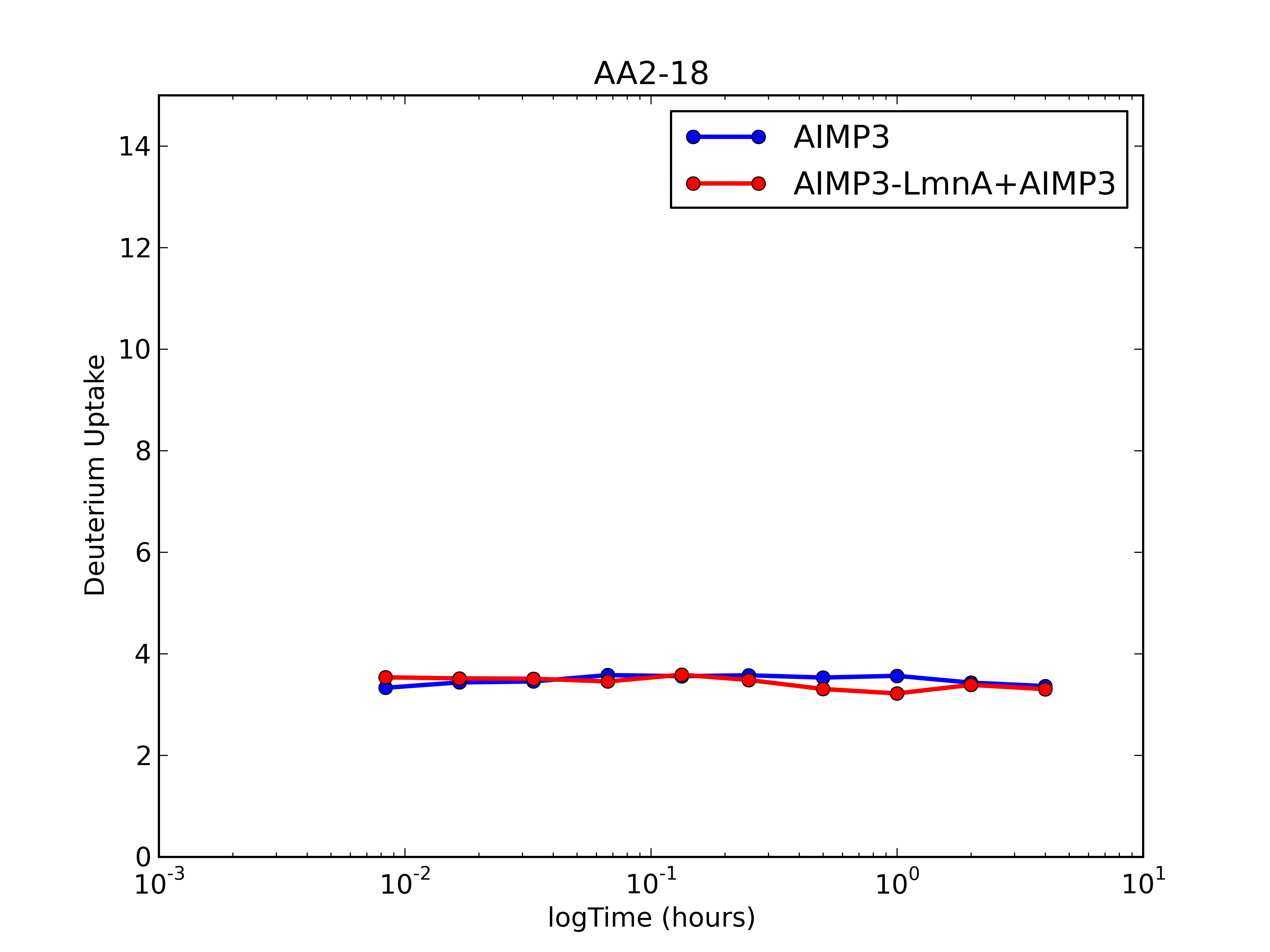

Supplement: S2 File — (ZIP) [file pone.0181869.s004.zip › logfigure-LmnA-scale/AA2-18_charge_5_mz419.9.csv.csv.png]

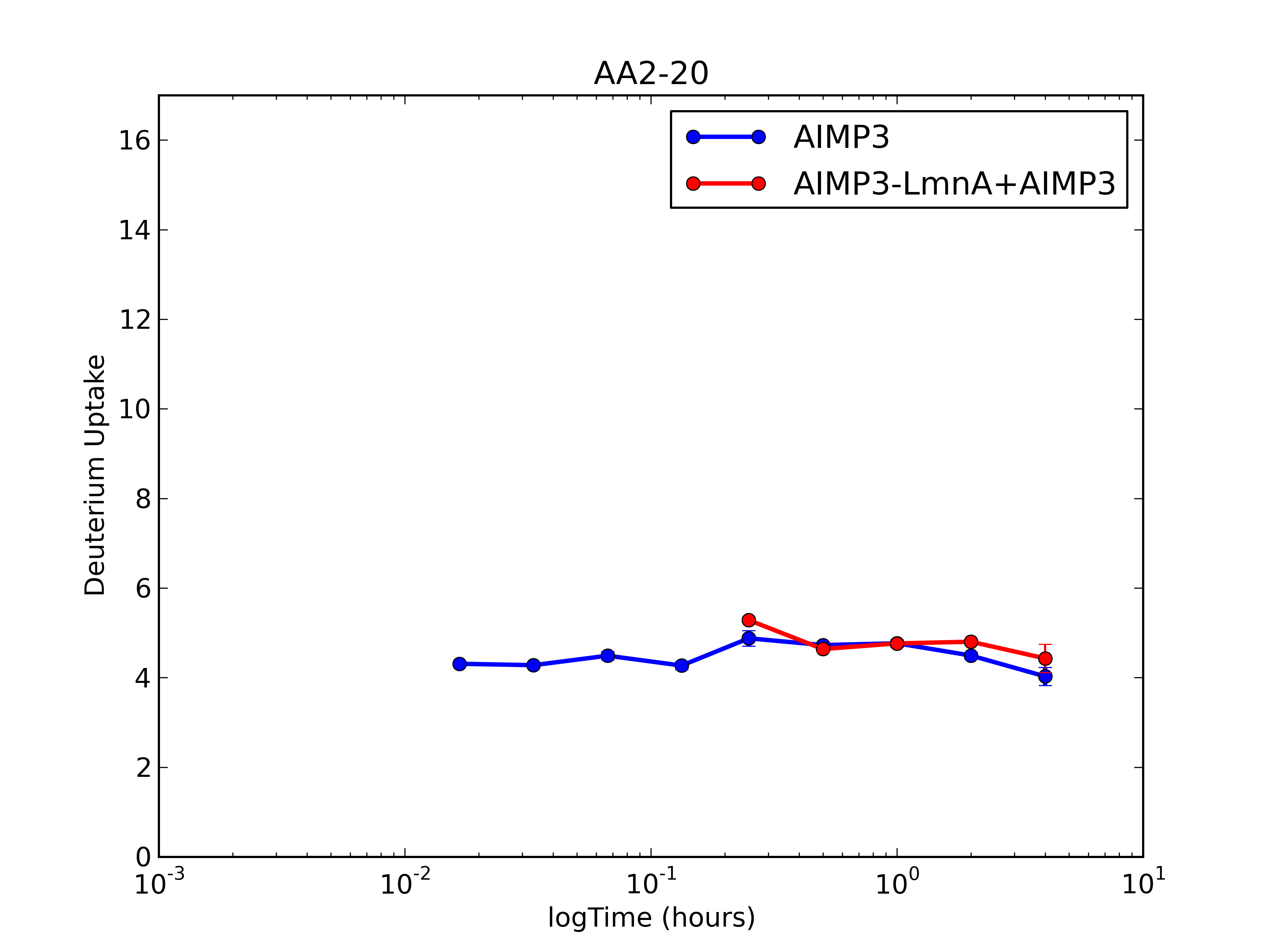

Supplement: S2 File — (ZIP) [file pone.0181869.s004.zip › logfigure-LmnA-scale/AA2-20_charge_3_mz752.0.csv.csv.png]

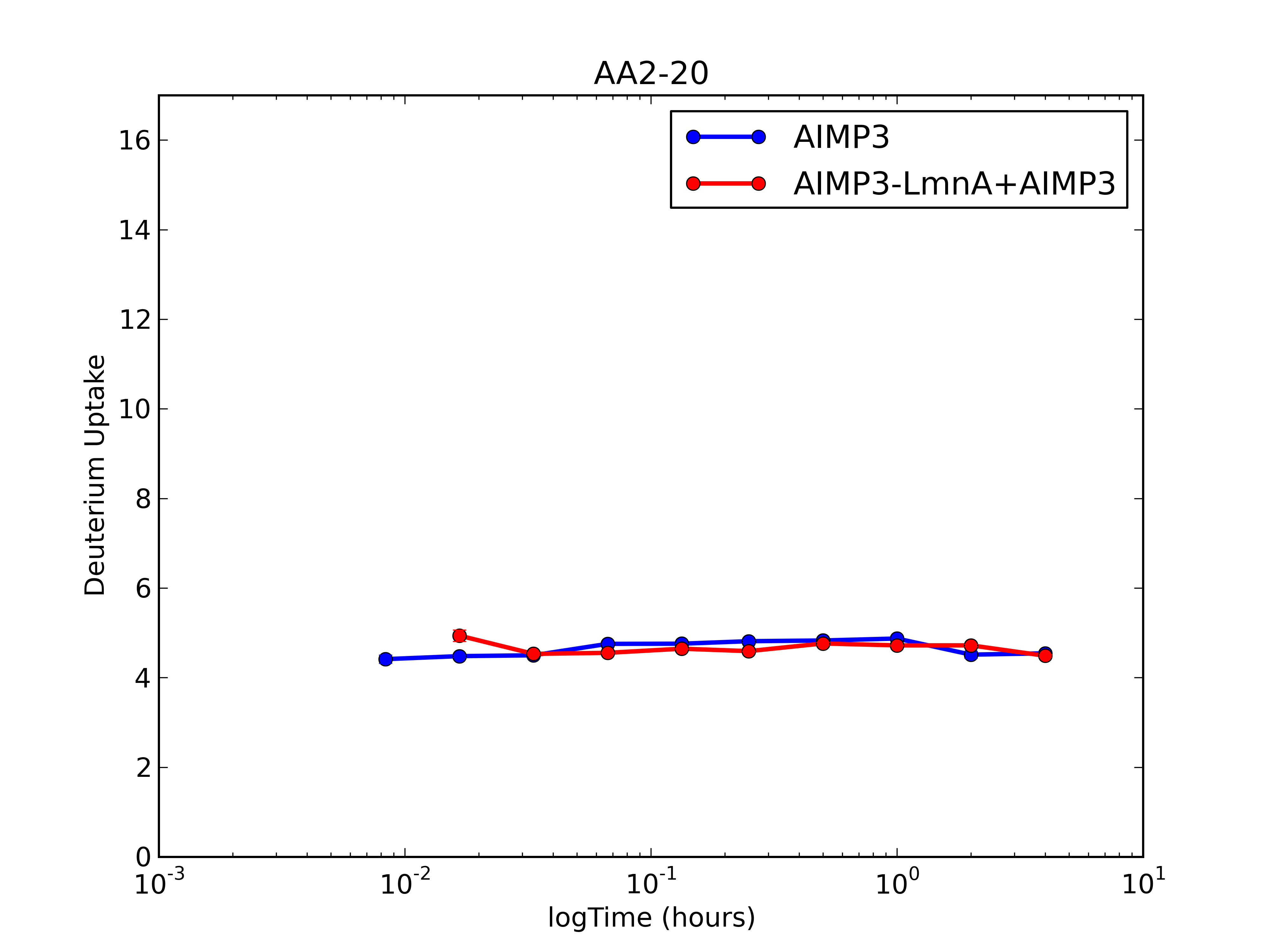

Supplement: S2 File — (ZIP) [file pone.0181869.s004.zip › logfigure-LmnA-scale/AA2-20_charge_4_mz564.2.csv.csv.png]

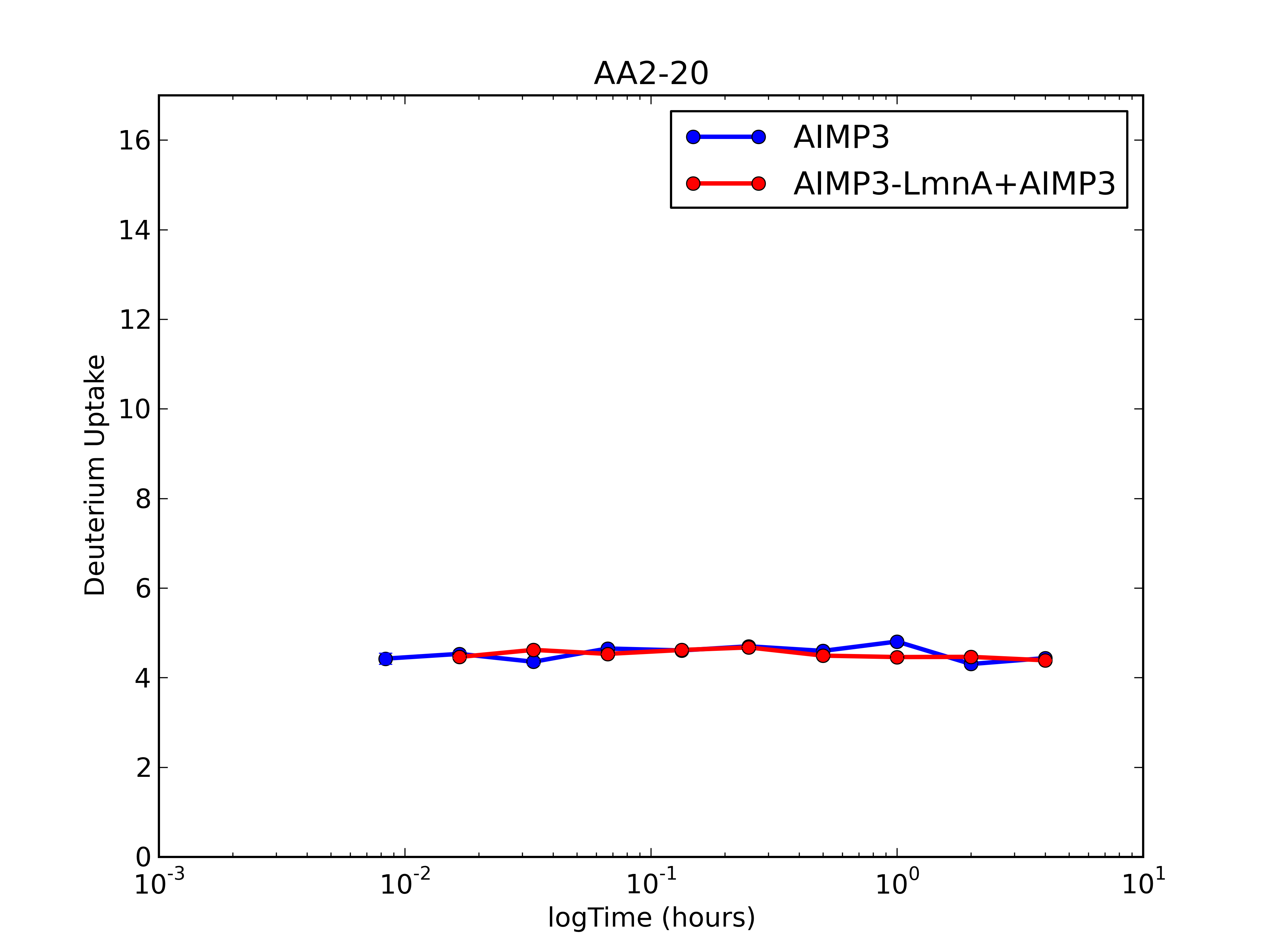

Supplement: S2 File — (ZIP) [file pone.0181869.s004.zip › logfigure-LmnA-scale/AA2-20_charge_5_mz451.6.csv.csv.png]

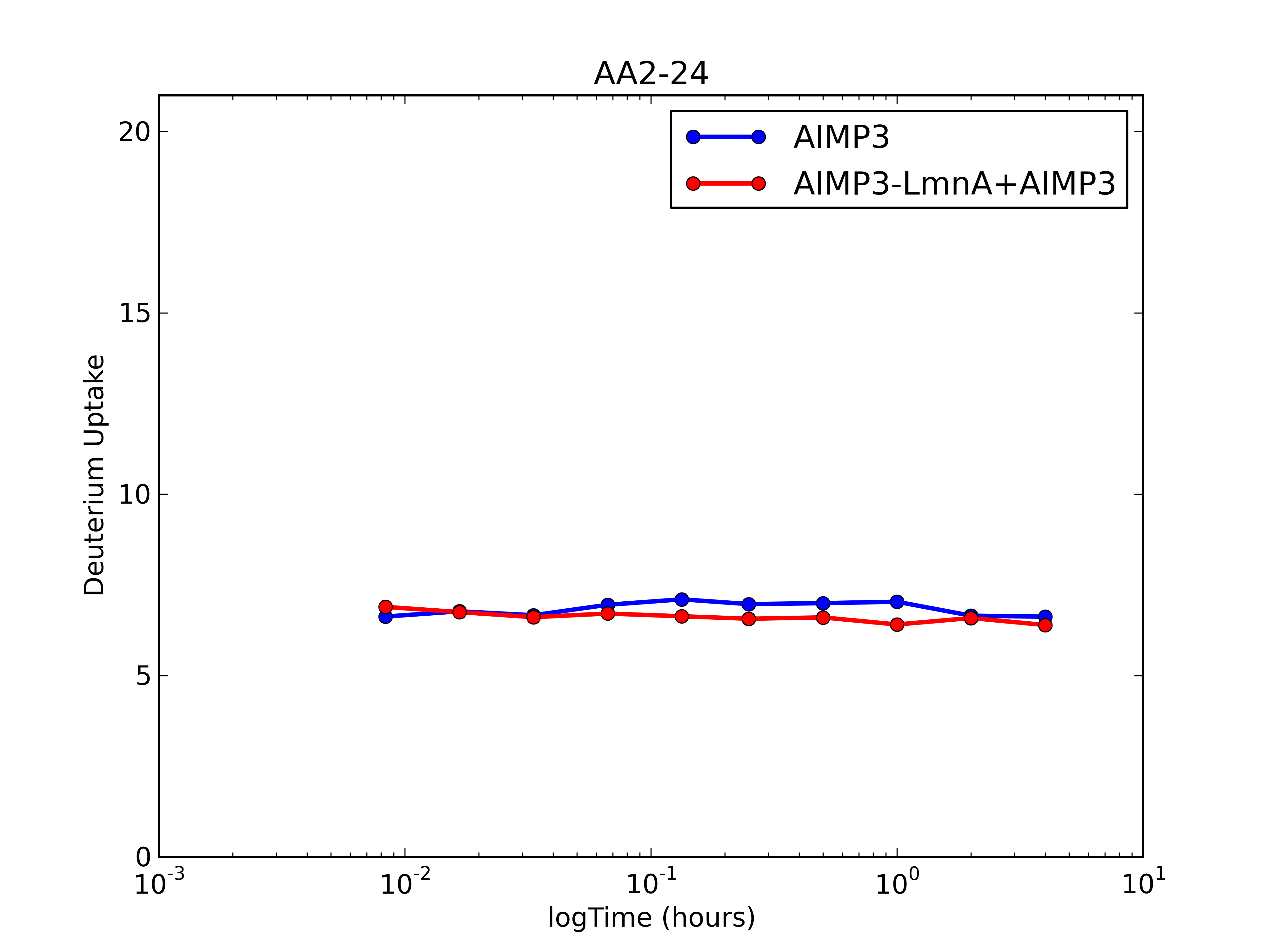

Supplement: S2 File — (ZIP) [file pone.0181869.s004.zip › logfigure-LmnA-scale/AA2-24_charge_4_mz679.5.csv.csv.png]

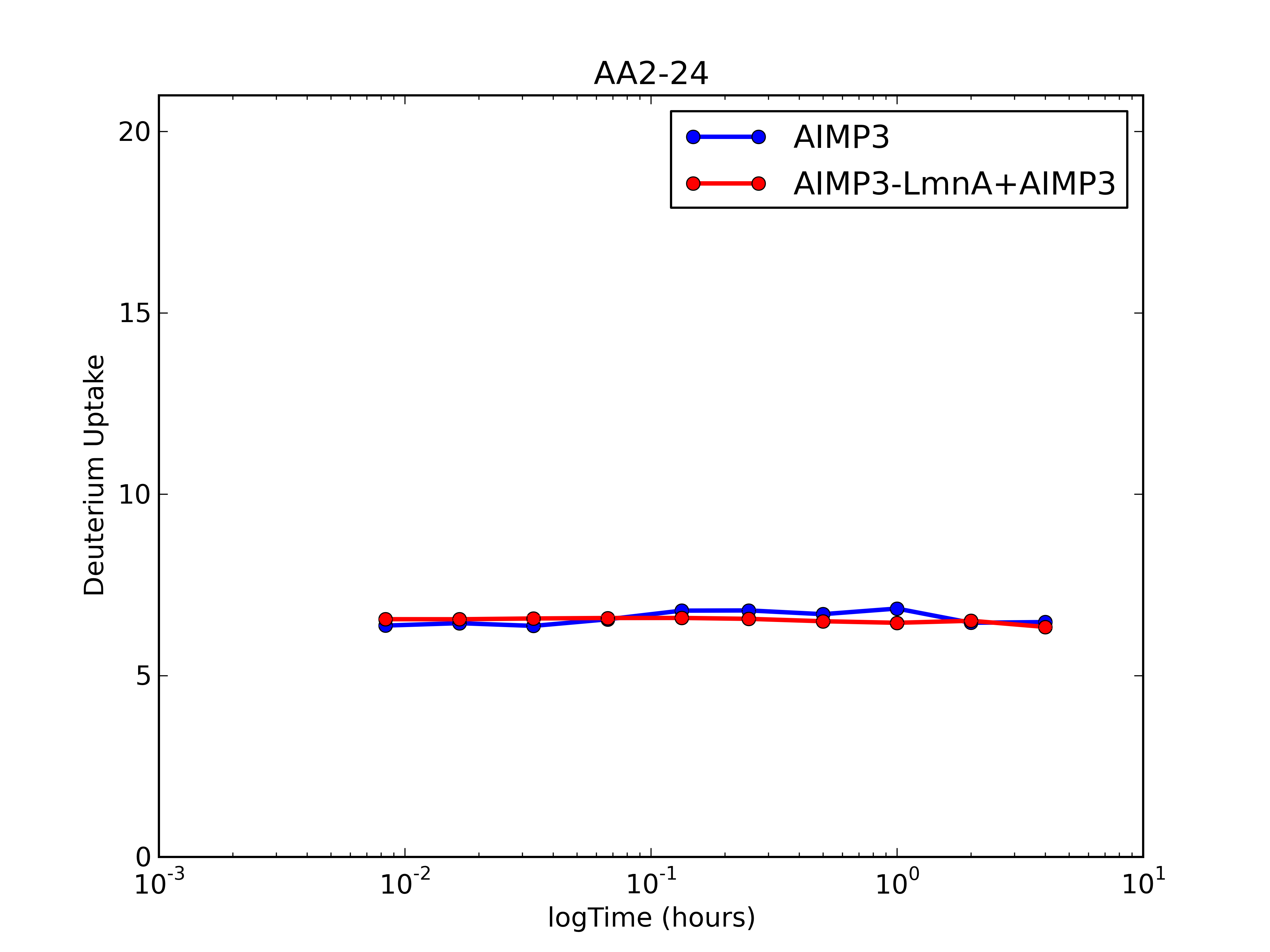

Supplement: S2 File — (ZIP) [file pone.0181869.s004.zip › logfigure-LmnA-scale/AA2-24_charge_5_mz543.8.csv.csv.png]

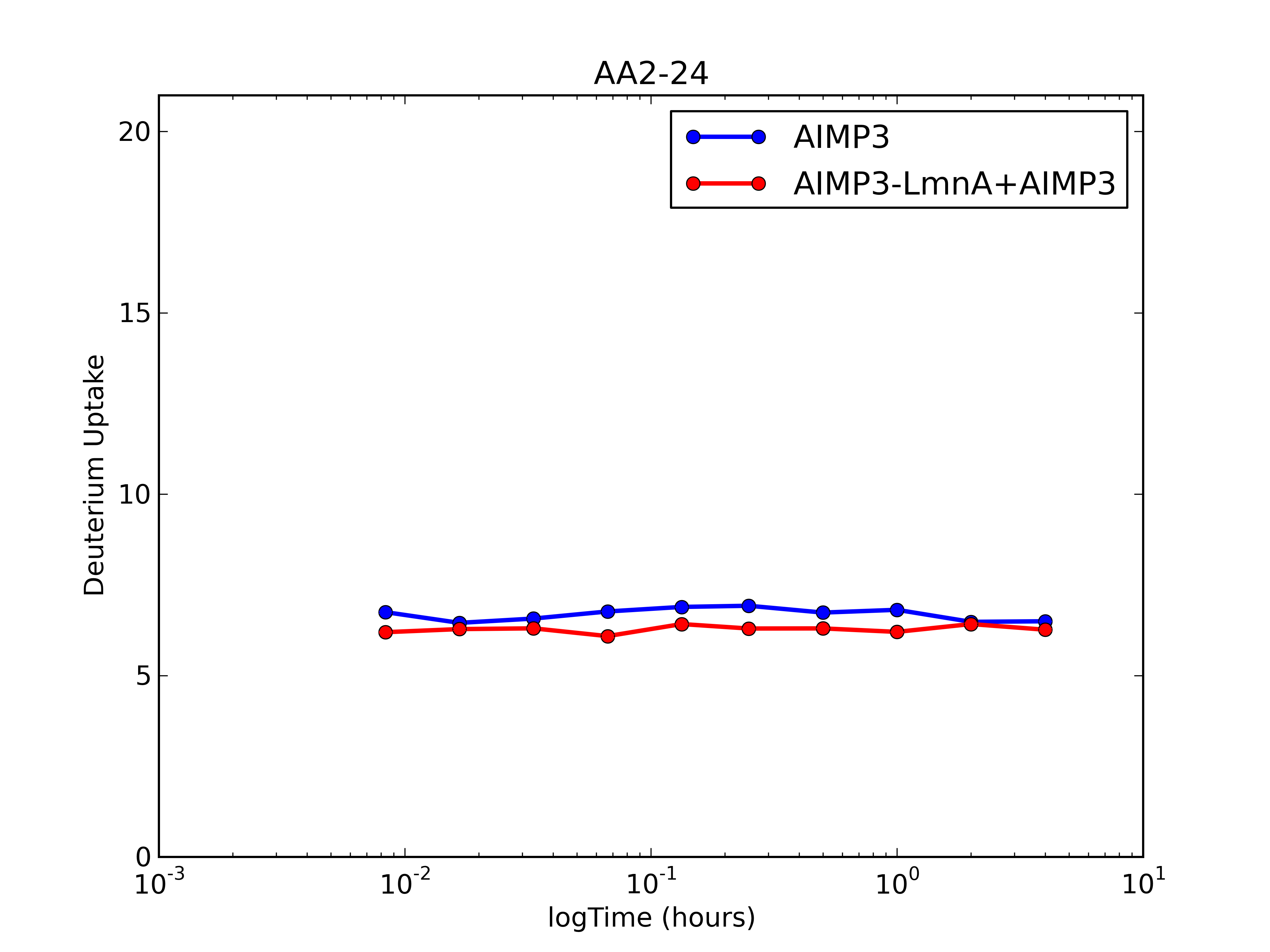

Supplement: S2 File — (ZIP) [file pone.0181869.s004.zip › logfigure-LmnA-scale/AA2-24_charge_6_mz453.3.csv.csv.png]

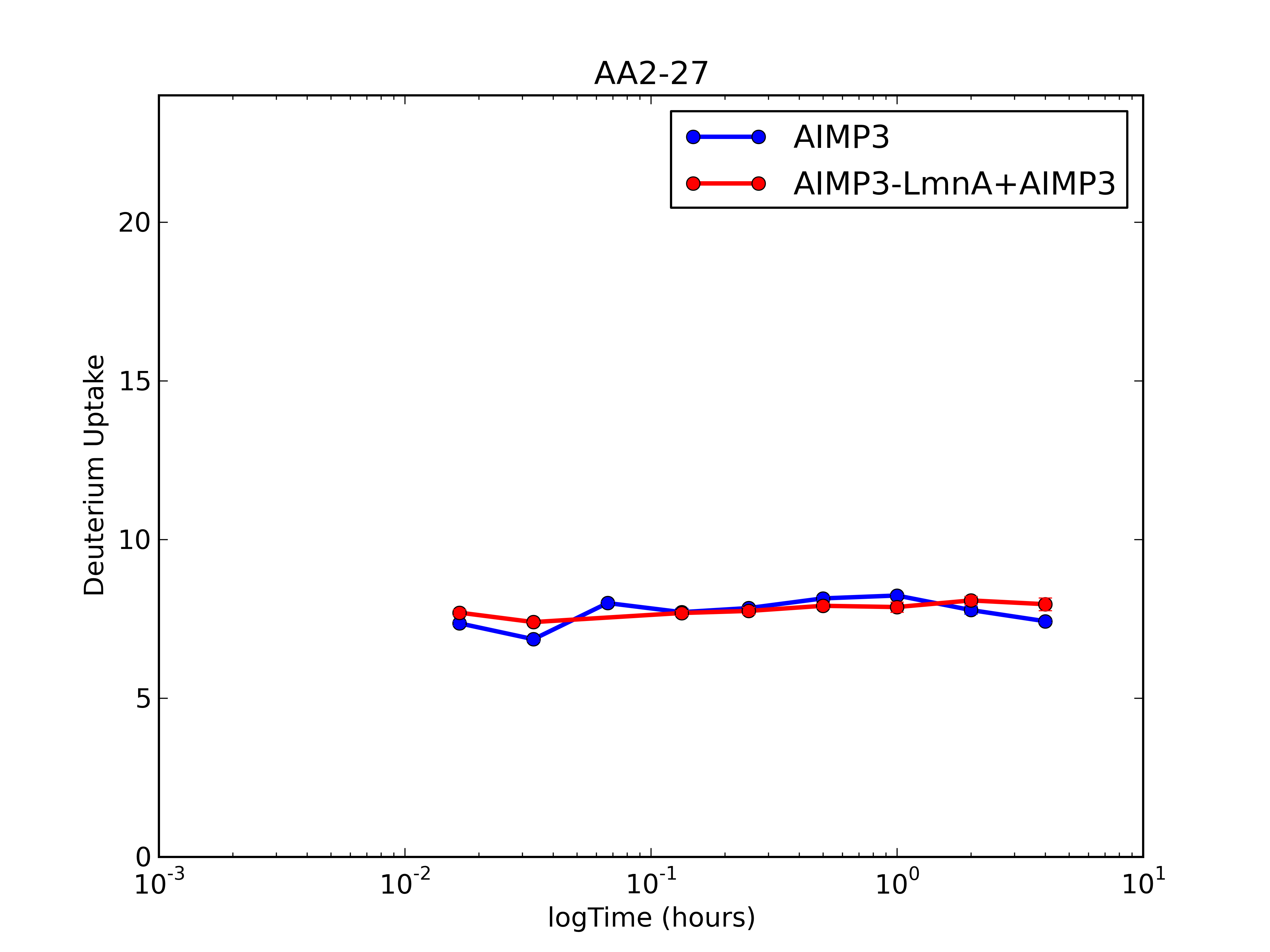

Supplement: S2 File — (ZIP) [file pone.0181869.s004.zip › logfigure-LmnA-scale/AA2-27_charge_4_mz770.3.csv.csv.png]

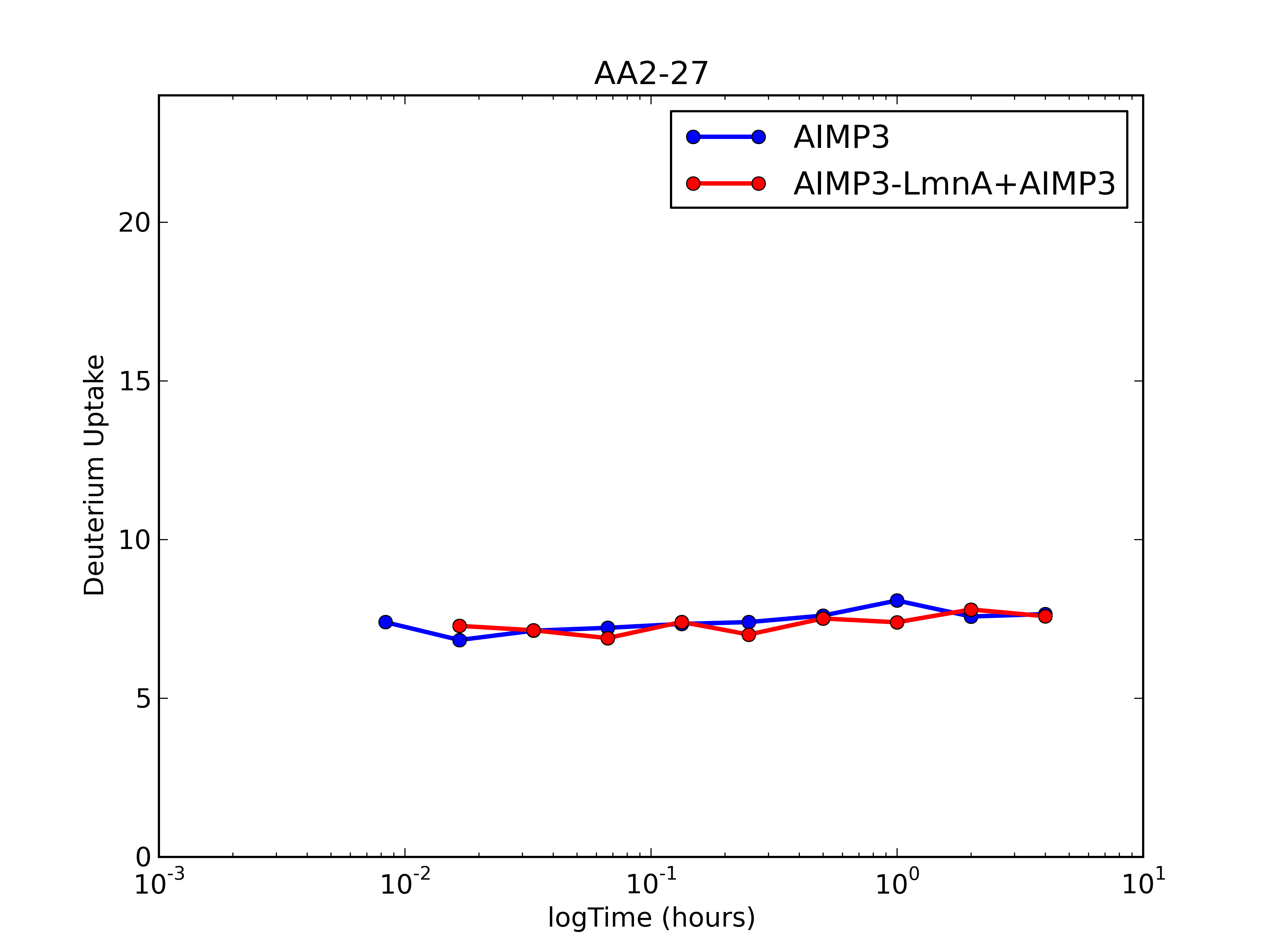

Supplement: S2 File — (ZIP) [file pone.0181869.s004.zip › logfigure-LmnA-scale/AA2-27_charge_5_mz616.4.csv.csv.png]

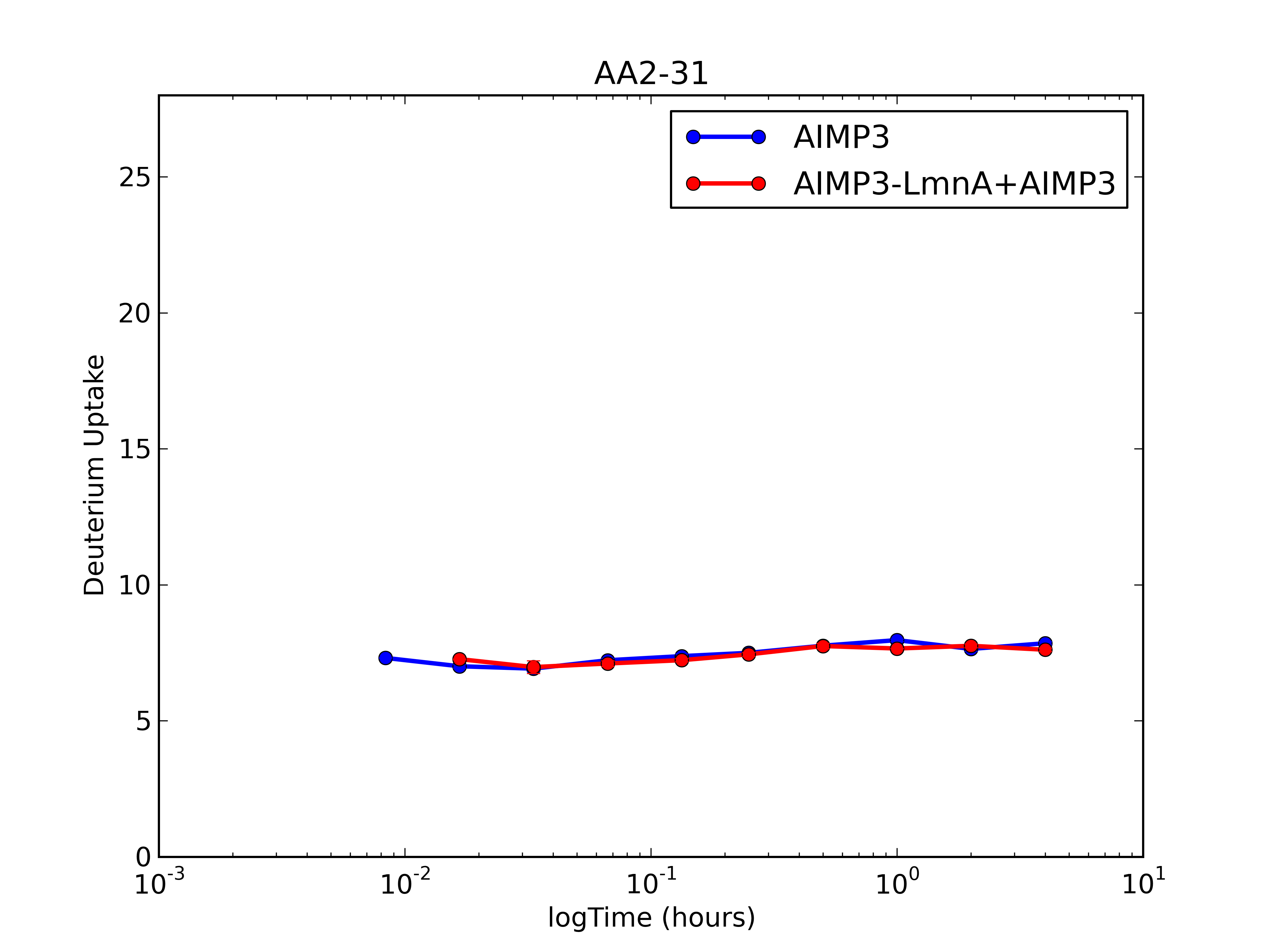

Supplement: S2 File — (ZIP) [file pone.0181869.s004.zip › logfigure-LmnA-scale/AA2-31_charge_4_mz881.4.csv.csv.png]

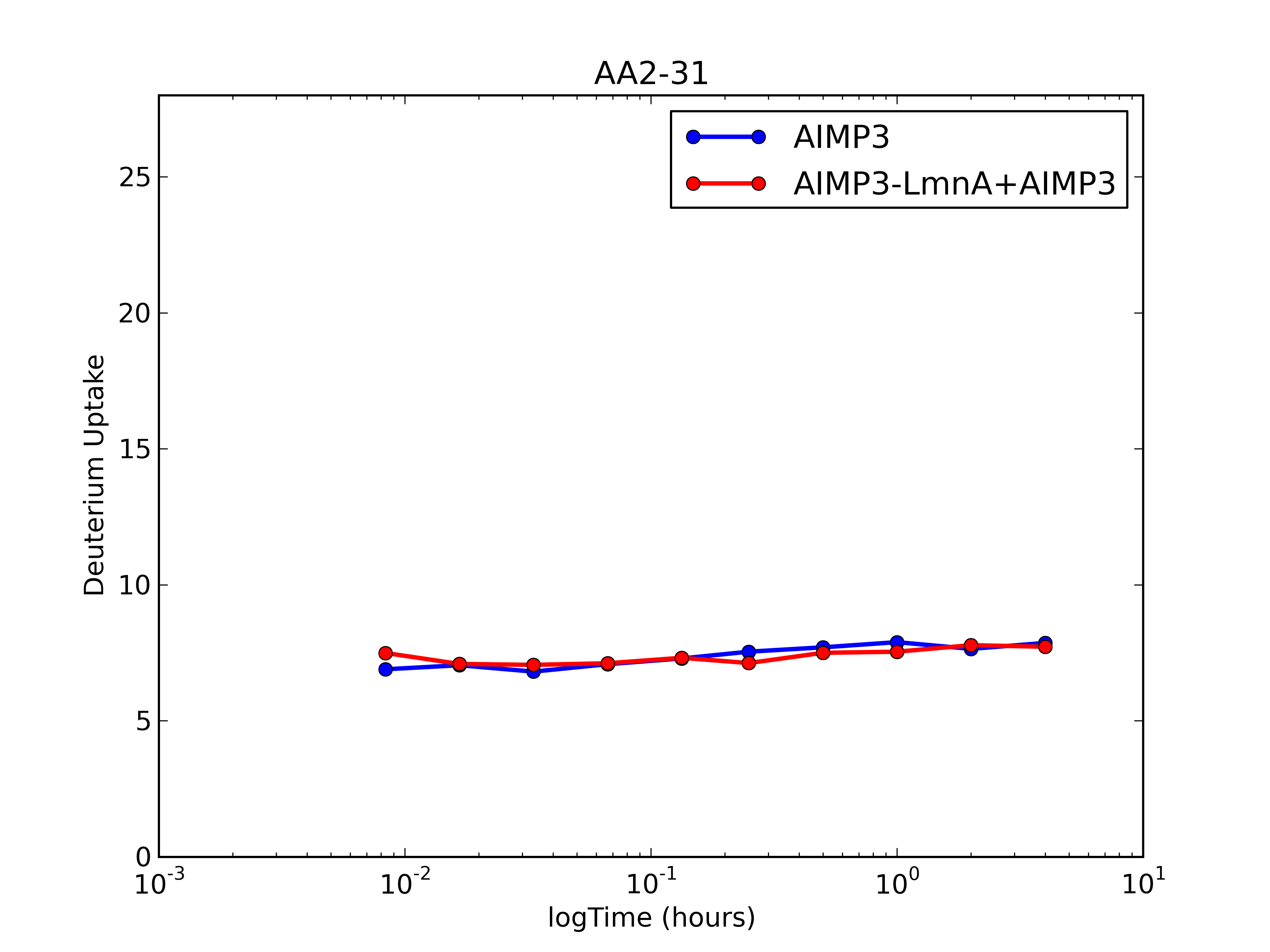

Supplement: S2 File — (ZIP) [file pone.0181869.s004.zip › logfigure-LmnA-scale/AA2-31_charge_5_mz705.3.csv.csv.png]

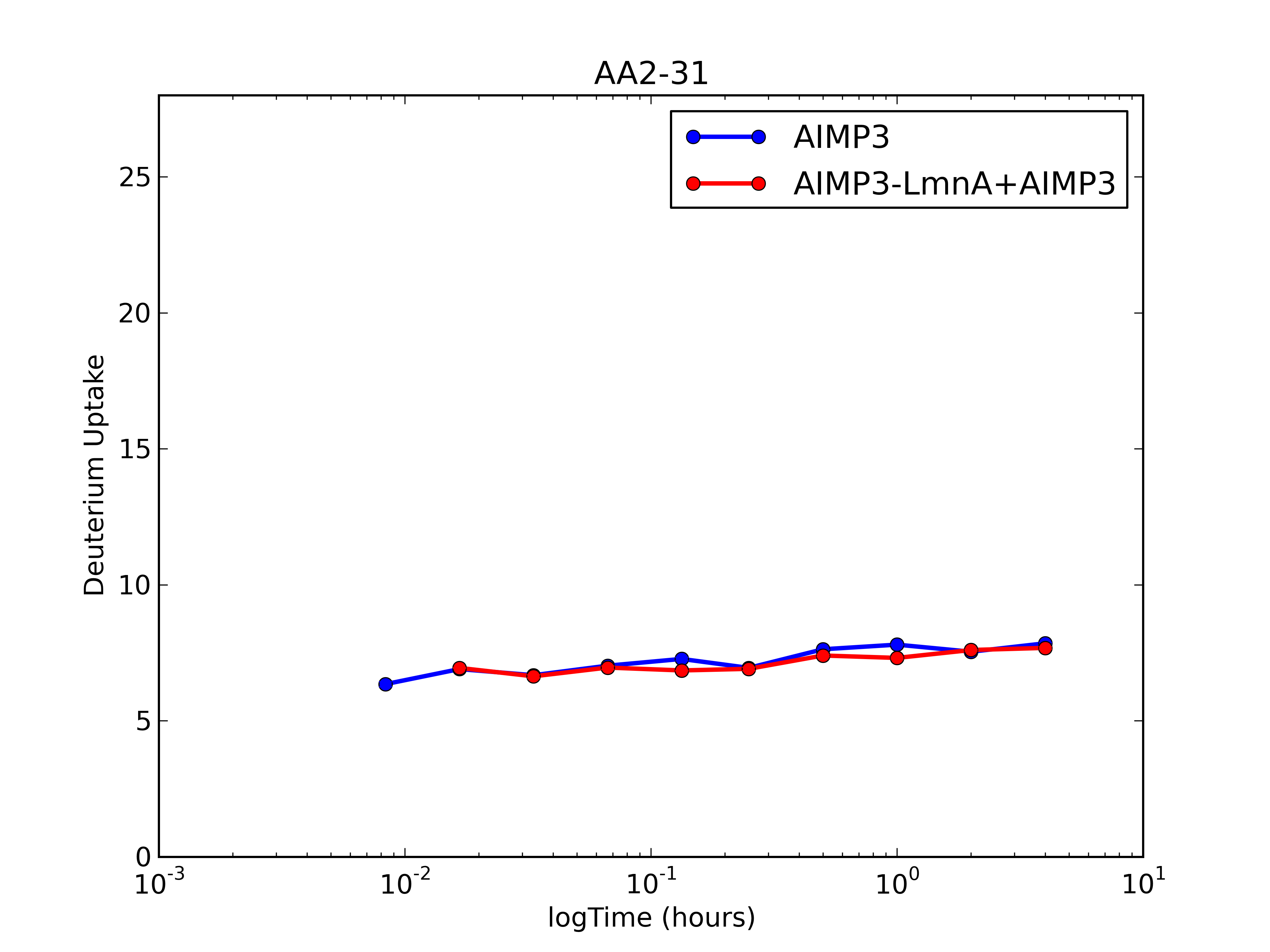

Supplement: S2 File — (ZIP) [file pone.0181869.s004.zip › logfigure-LmnA-scale/AA2-31_charge_6_mz587.9.csv.csv.png]

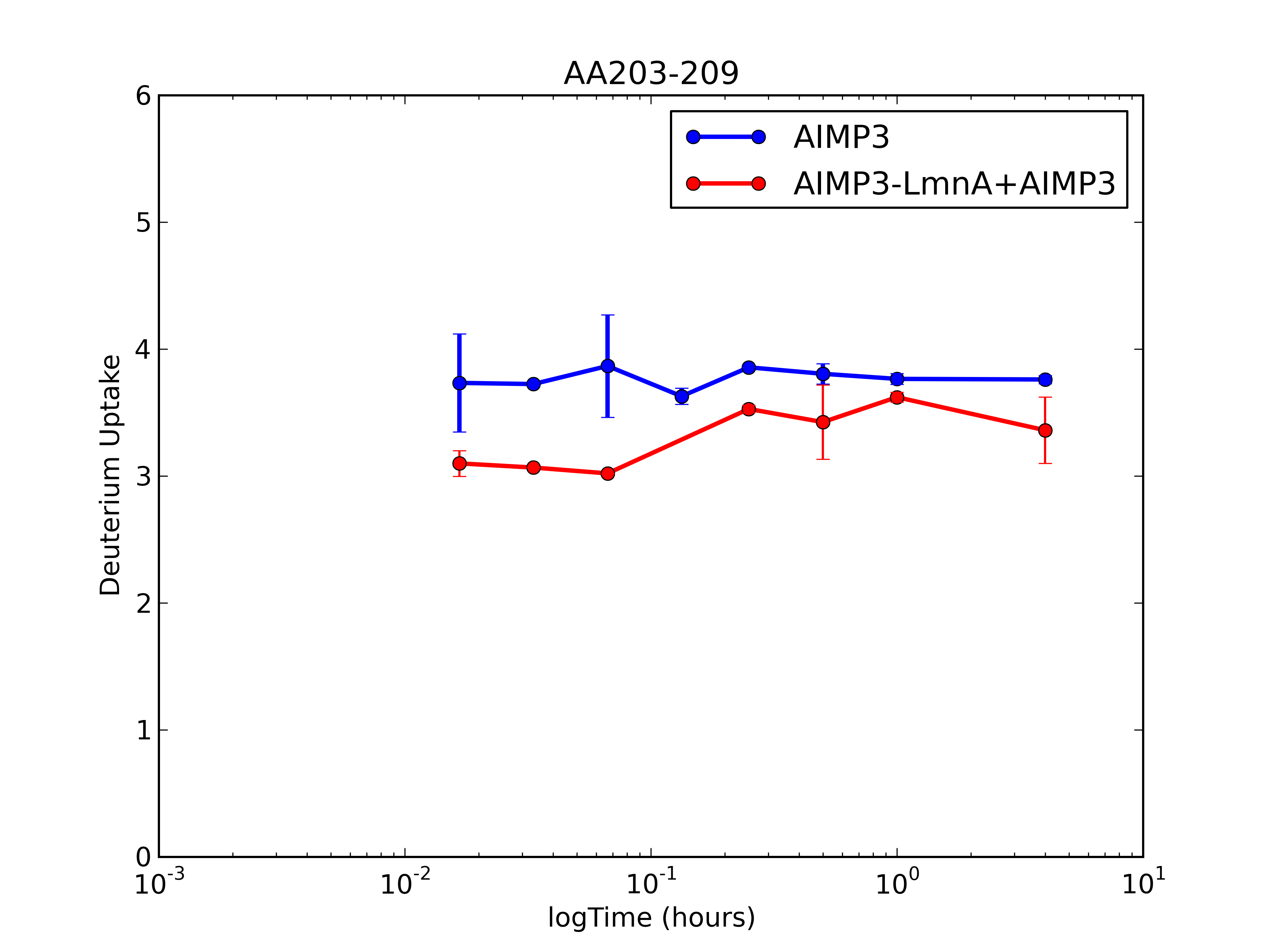

Supplement: S2 File — (ZIP) [file pone.0181869.s004.zip › logfigure-LmnA-scale/AA203-209_charge_1_mz852.4.csv.csv.png]

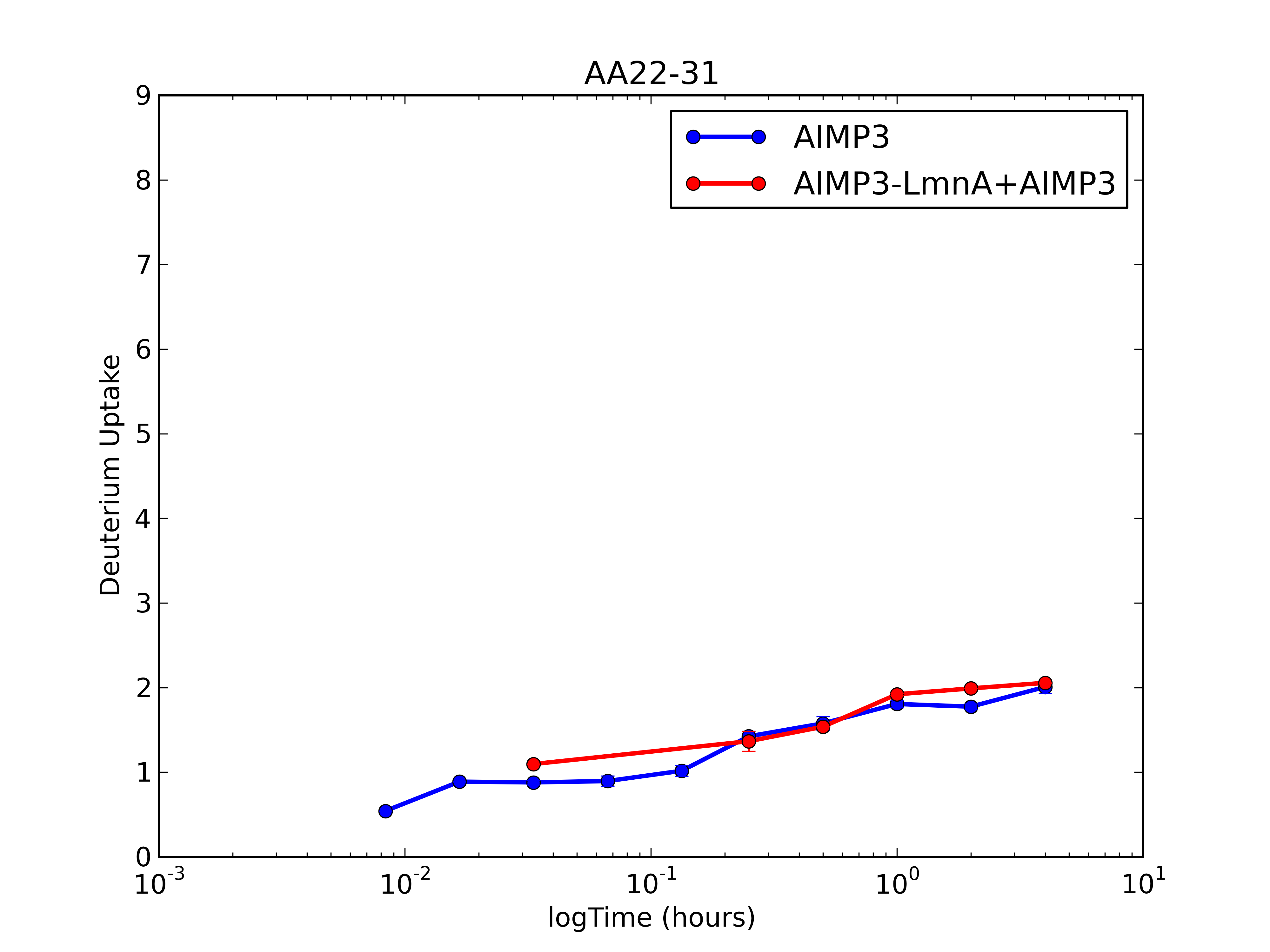

Supplement: S2 File — (ZIP) [file pone.0181869.s004.zip › logfigure-LmnA-scale/AA22-31_charge_2_mz578.7.csv.csv.png]

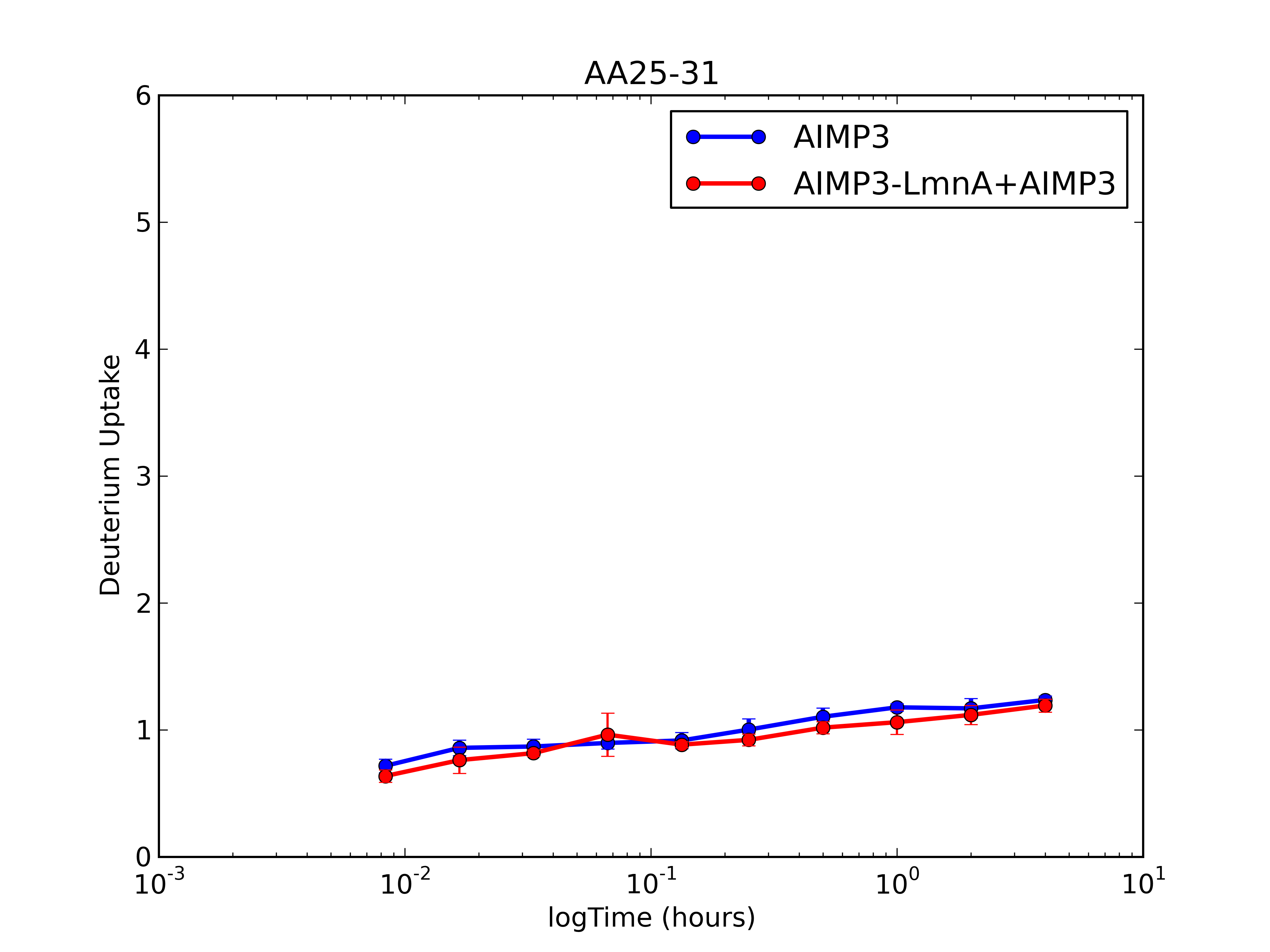

Supplement: S2 File — (ZIP) [file pone.0181869.s004.zip › logfigure-LmnA-scale/AA25-31_charge_1_mz826.4.csv.csv.png]

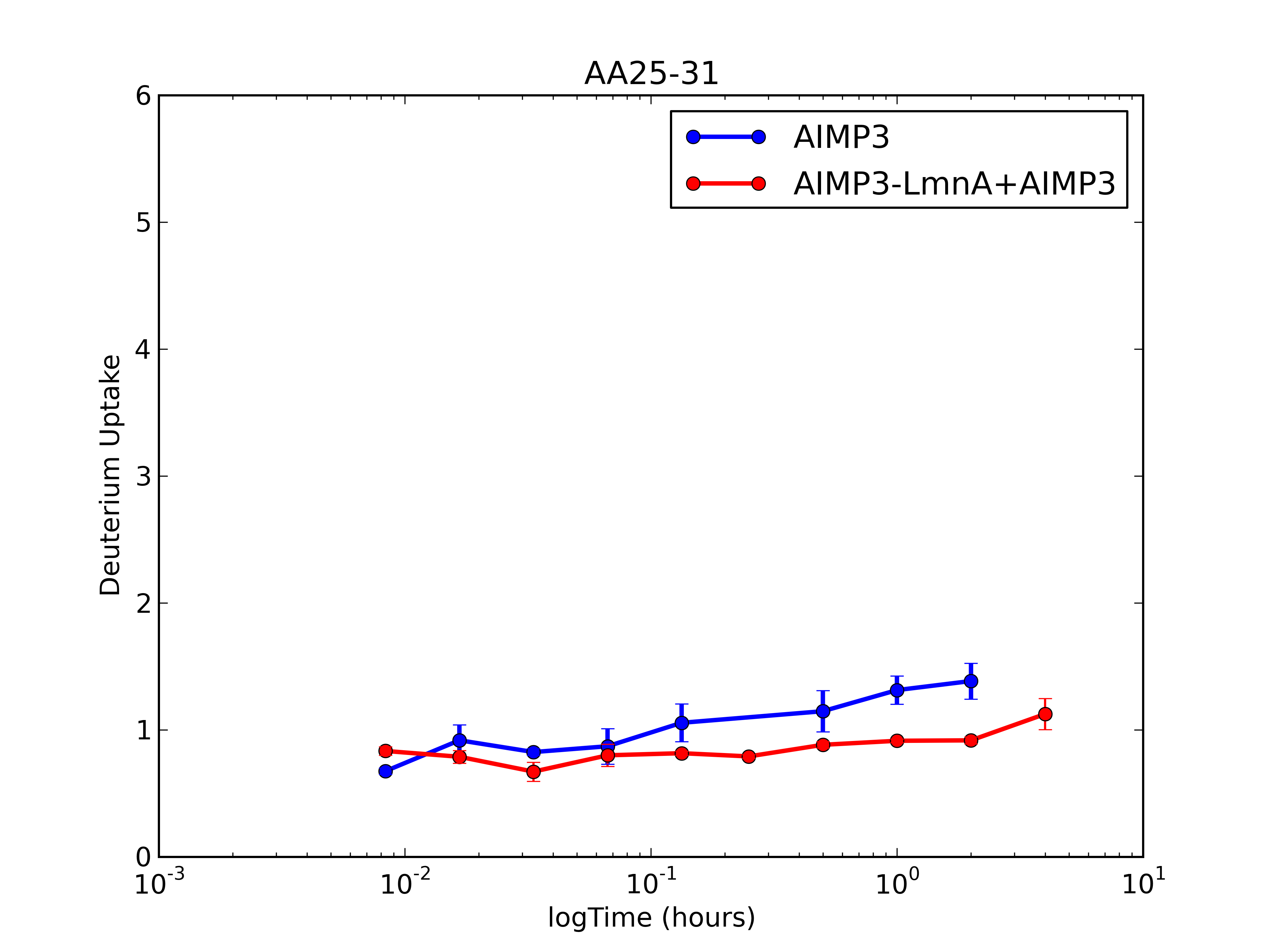

Supplement: S2 File — (ZIP) [file pone.0181869.s004.zip › logfigure-LmnA-scale/AA25-31_charge_2_mz413.7.csv.csv.png]

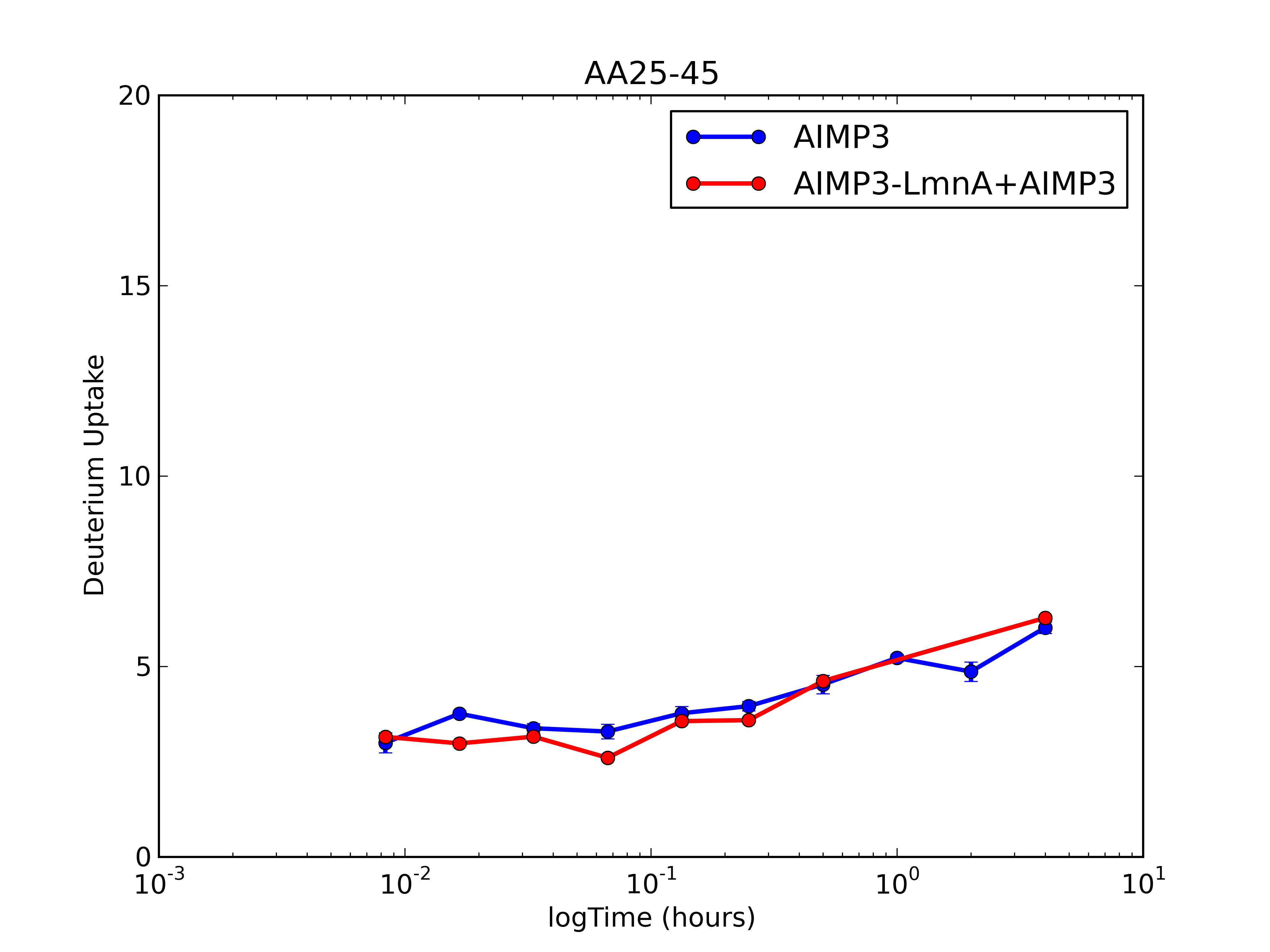

Supplement: S2 File — (ZIP) [file pone.0181869.s004.zip › logfigure-LmnA-scale/AA25-45_charge_3_mz758.0.csv.csv.png]

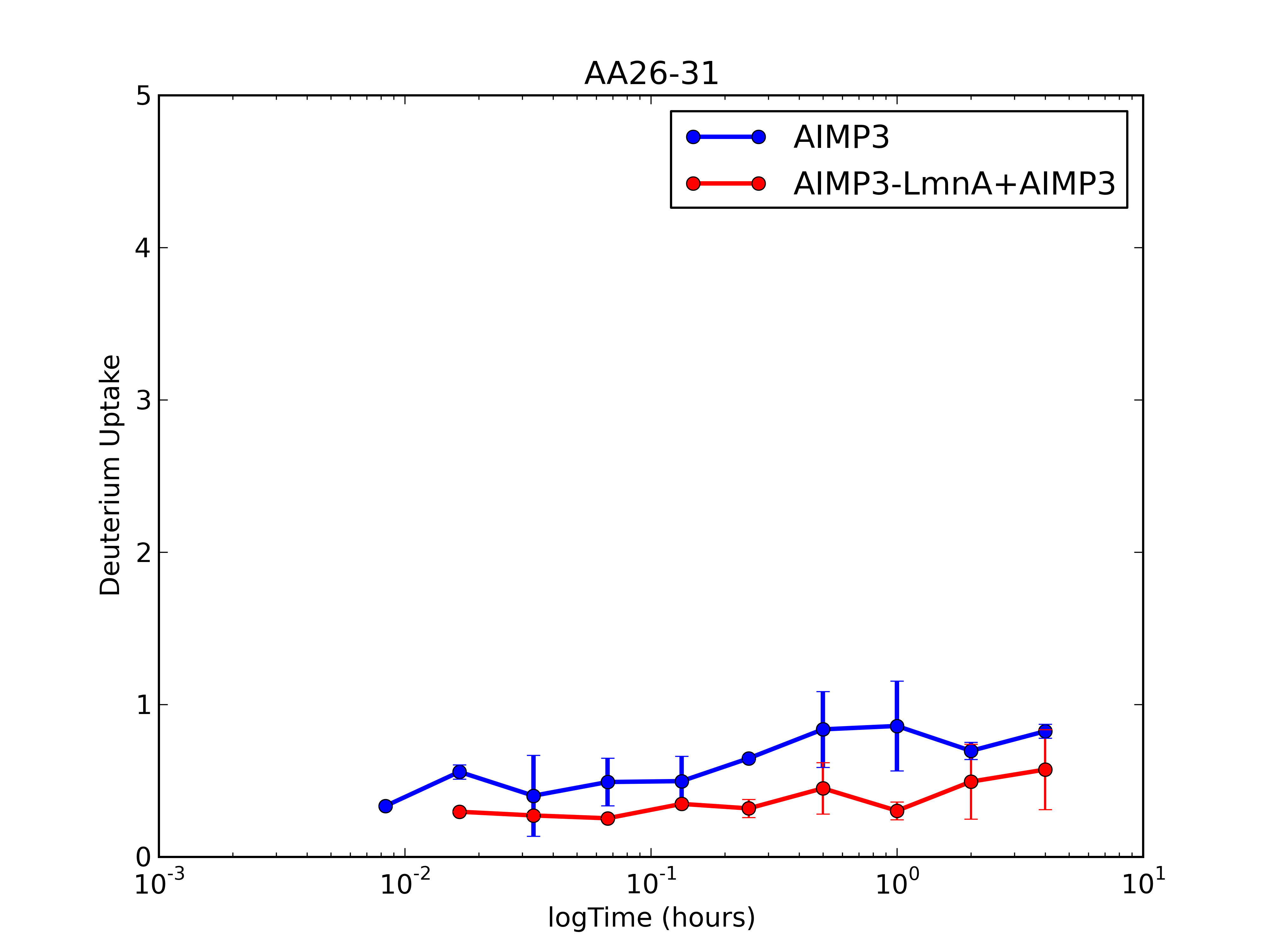

Supplement: S2 File — (ZIP) [file pone.0181869.s004.zip › logfigure-LmnA-scale/AA26-31_charge_1_mz713.3.csv.csv.png]

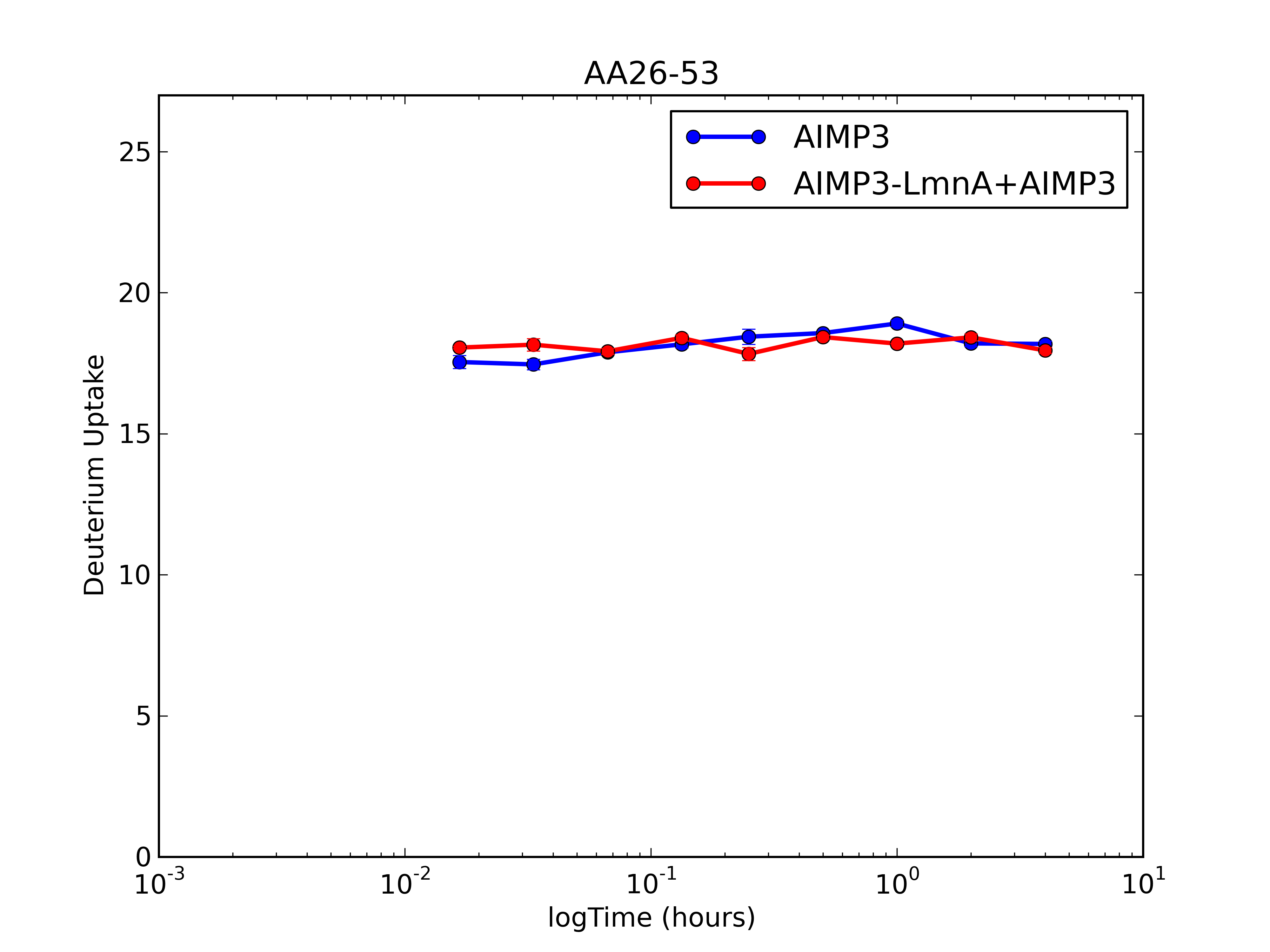

Supplement: S2 File — (ZIP) [file pone.0181869.s004.zip › logfigure-LmnA-scale/AA26-53_charge_3_mz1065.8.csv.csv.png]

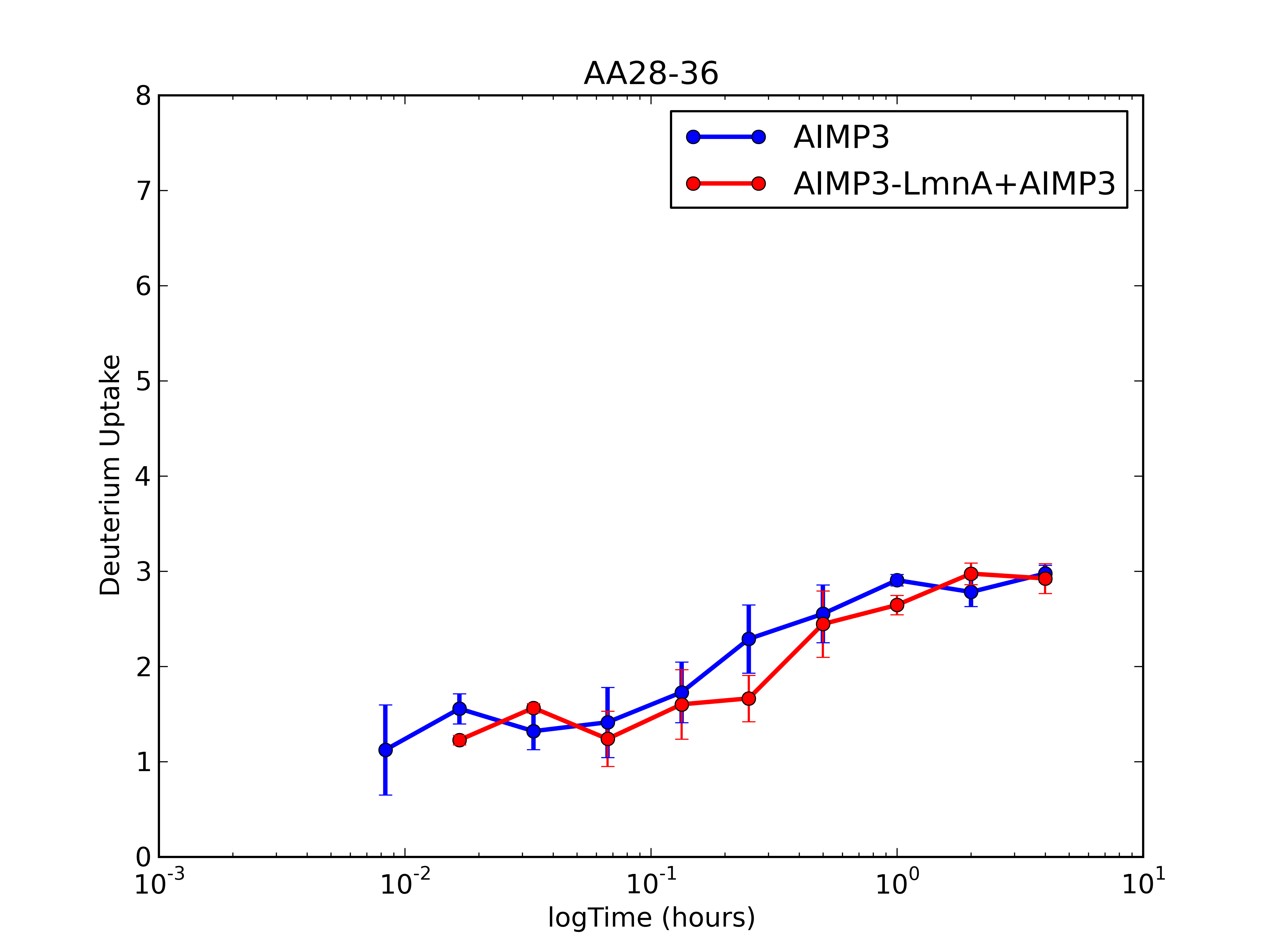

Supplement: S2 File — (ZIP) [file pone.0181869.s004.zip › logfigure-LmnA-scale/AA28-36_charge_1_mz1028.4.csv.csv.png]

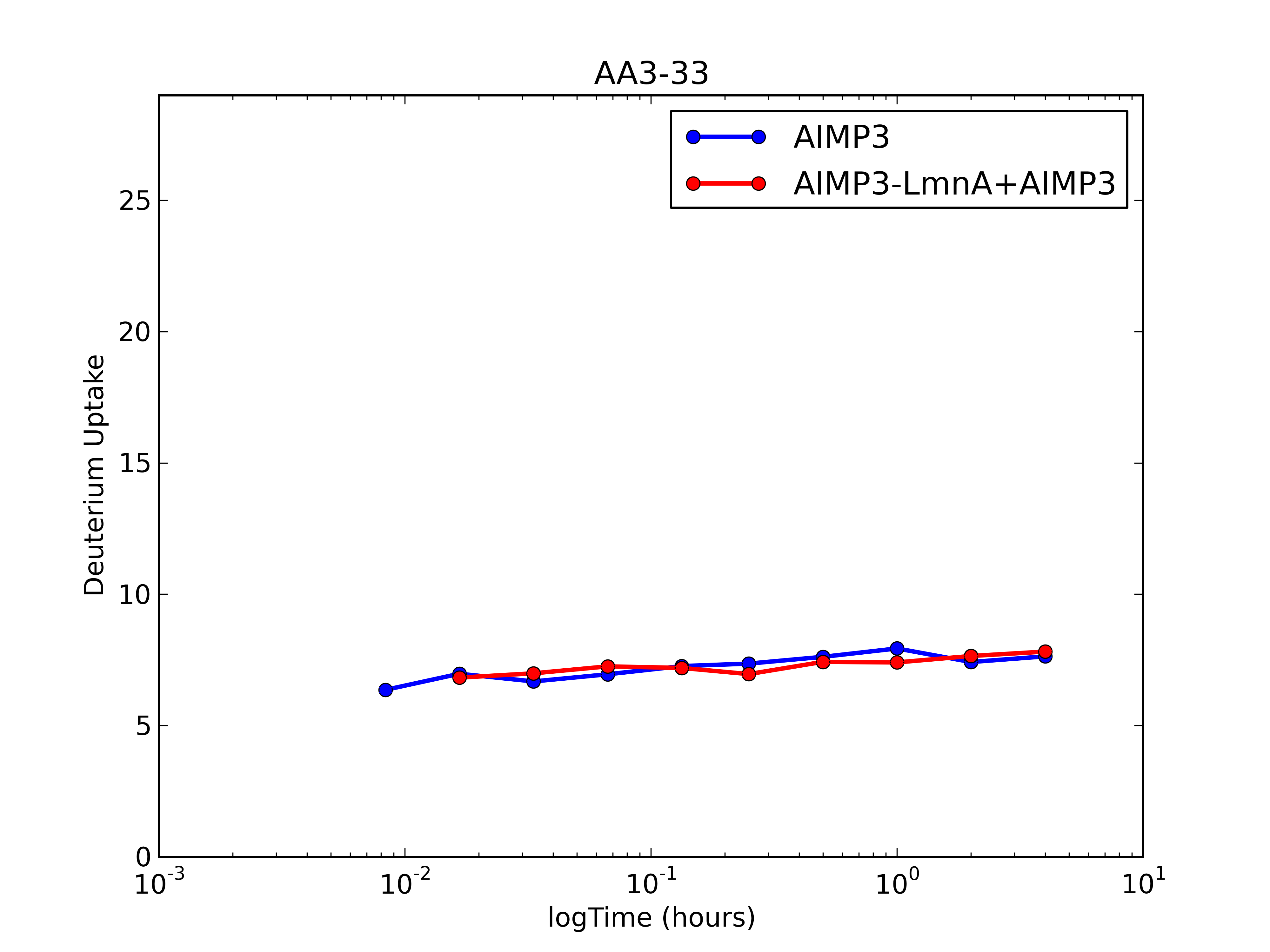

Supplement: S2 File — (ZIP) [file pone.0181869.s004.zip › logfigure-LmnA-scale/AA3-33_charge_6_mz617.4.csv.csv.png]

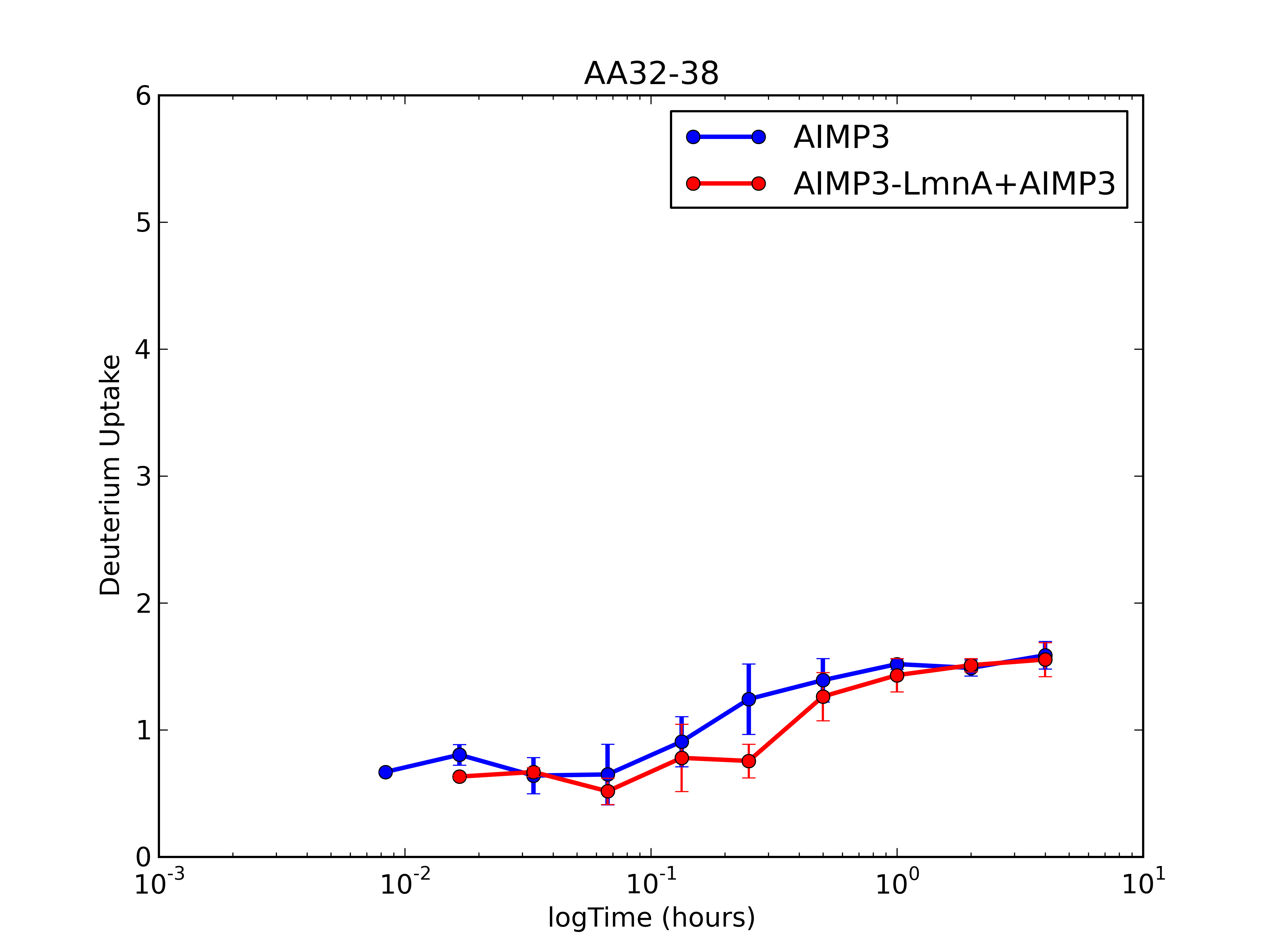

Supplement: S2 File — (ZIP) [file pone.0181869.s004.zip › logfigure-LmnA-scale/AA32-38_charge_1_mz796.3.csv.csv.png]

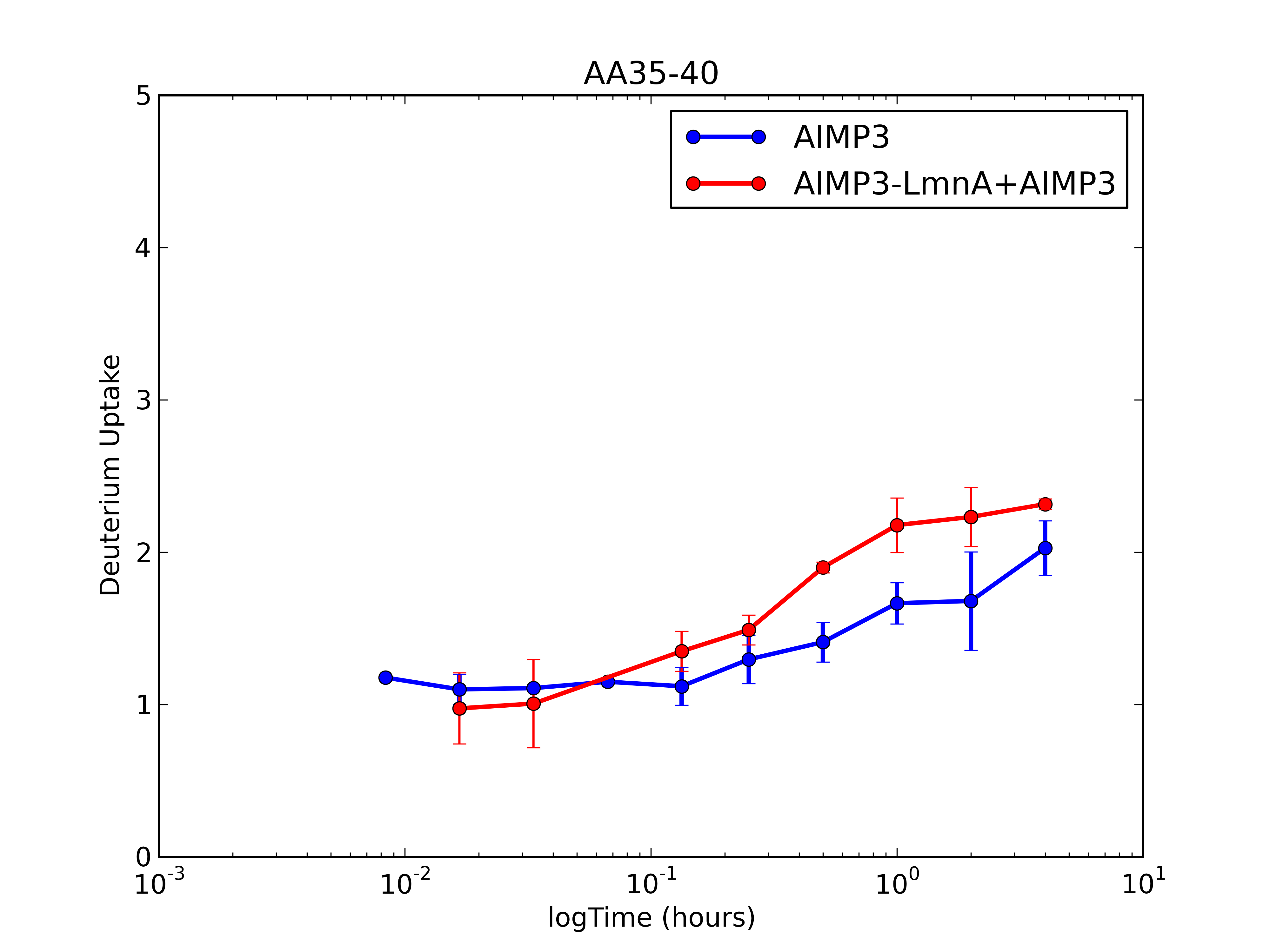

Supplement: S2 File — (ZIP) [file pone.0181869.s004.zip › logfigure-LmnA-scale/AA35-40_charge_1_mz646.3.csv.csv.png]

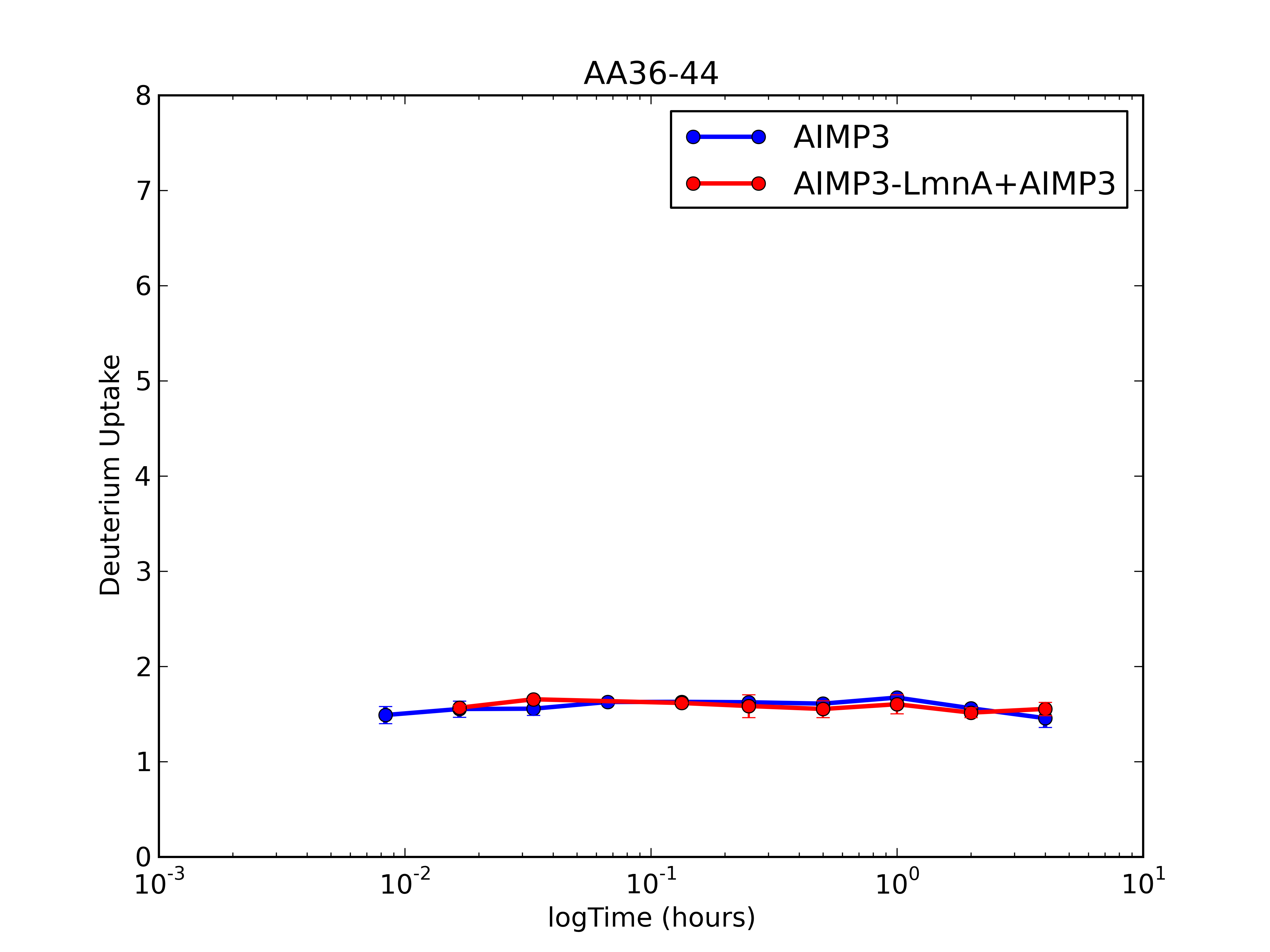

Supplement: S2 File — (ZIP) [file pone.0181869.s004.zip › logfigure-LmnA-scale/AA36-44_charge_2_mz451.2.csv.csv.png]

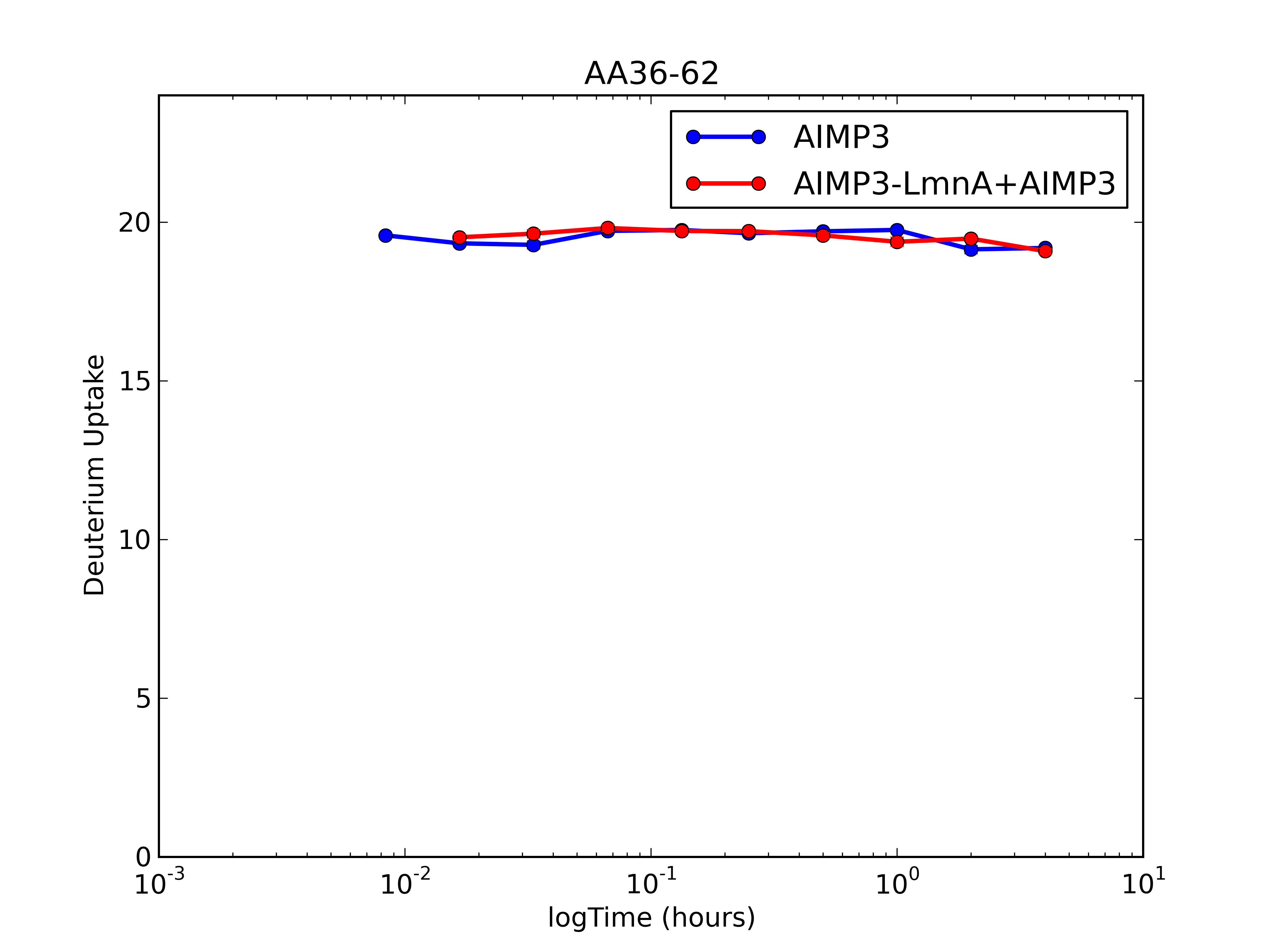

Supplement: S2 File — (ZIP) [file pone.0181869.s004.zip › logfigure-LmnA-scale/AA36-62_charge_3_mz987.8.csv.csv.png]

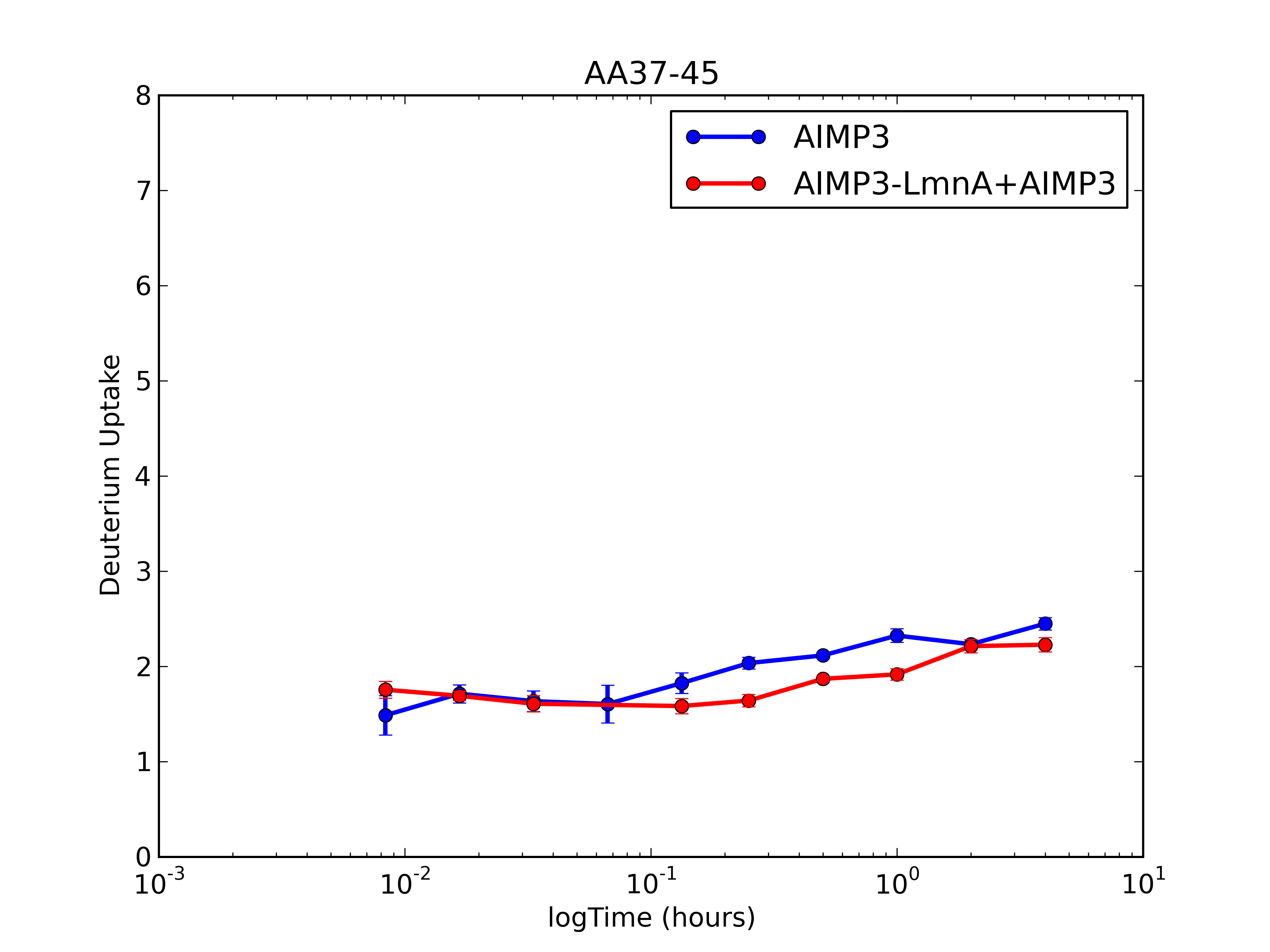

Supplement: S2 File — (ZIP) [file pone.0181869.s004.zip › logfigure-LmnA-scale/AA37-45_charge_2_mz450.2.csv.csv.png]

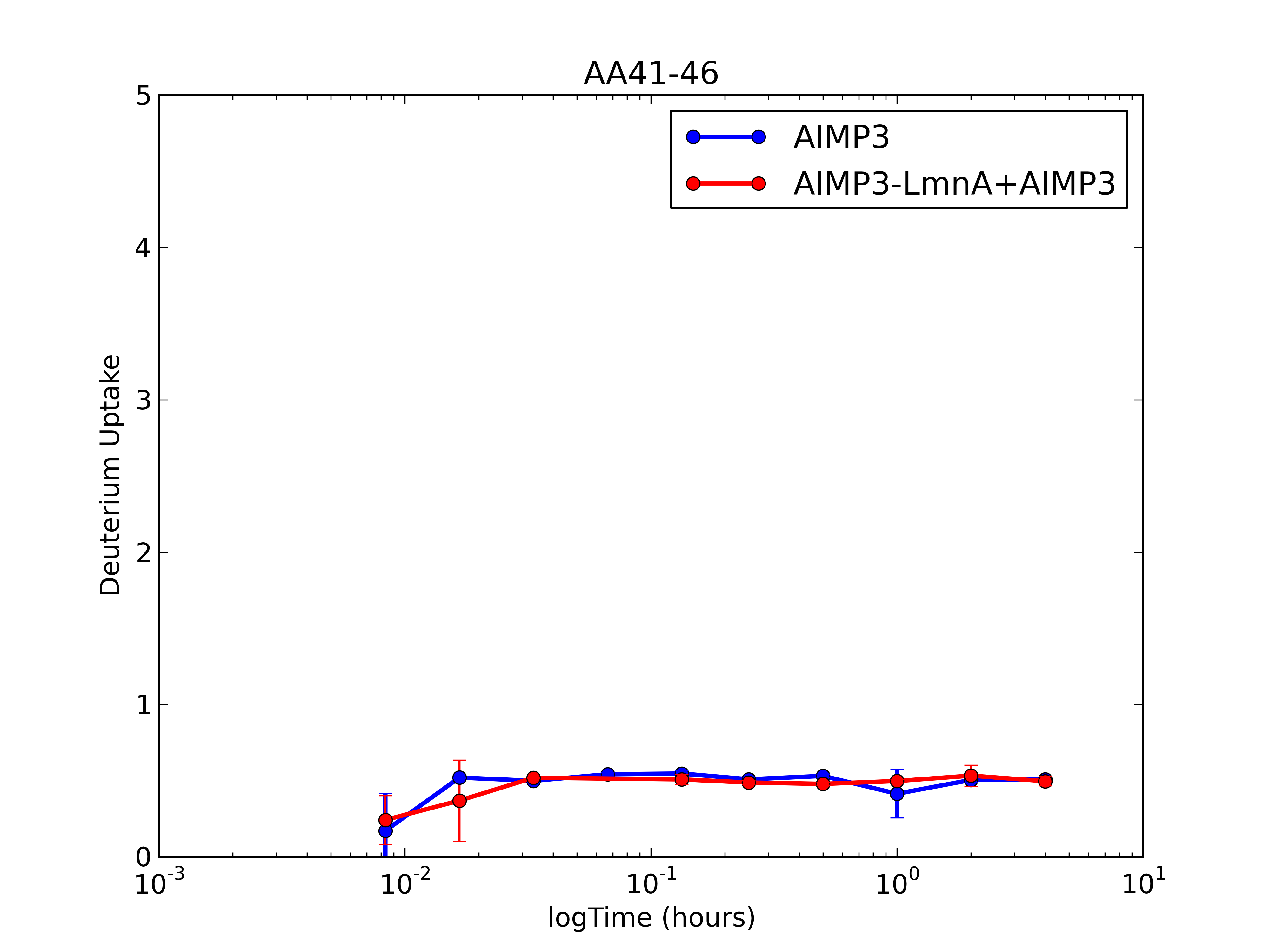

Supplement: S2 File — (ZIP) [file pone.0181869.s004.zip › logfigure-LmnA-scale/AA41-46_charge_1_mz587.3.csv.csv.png]

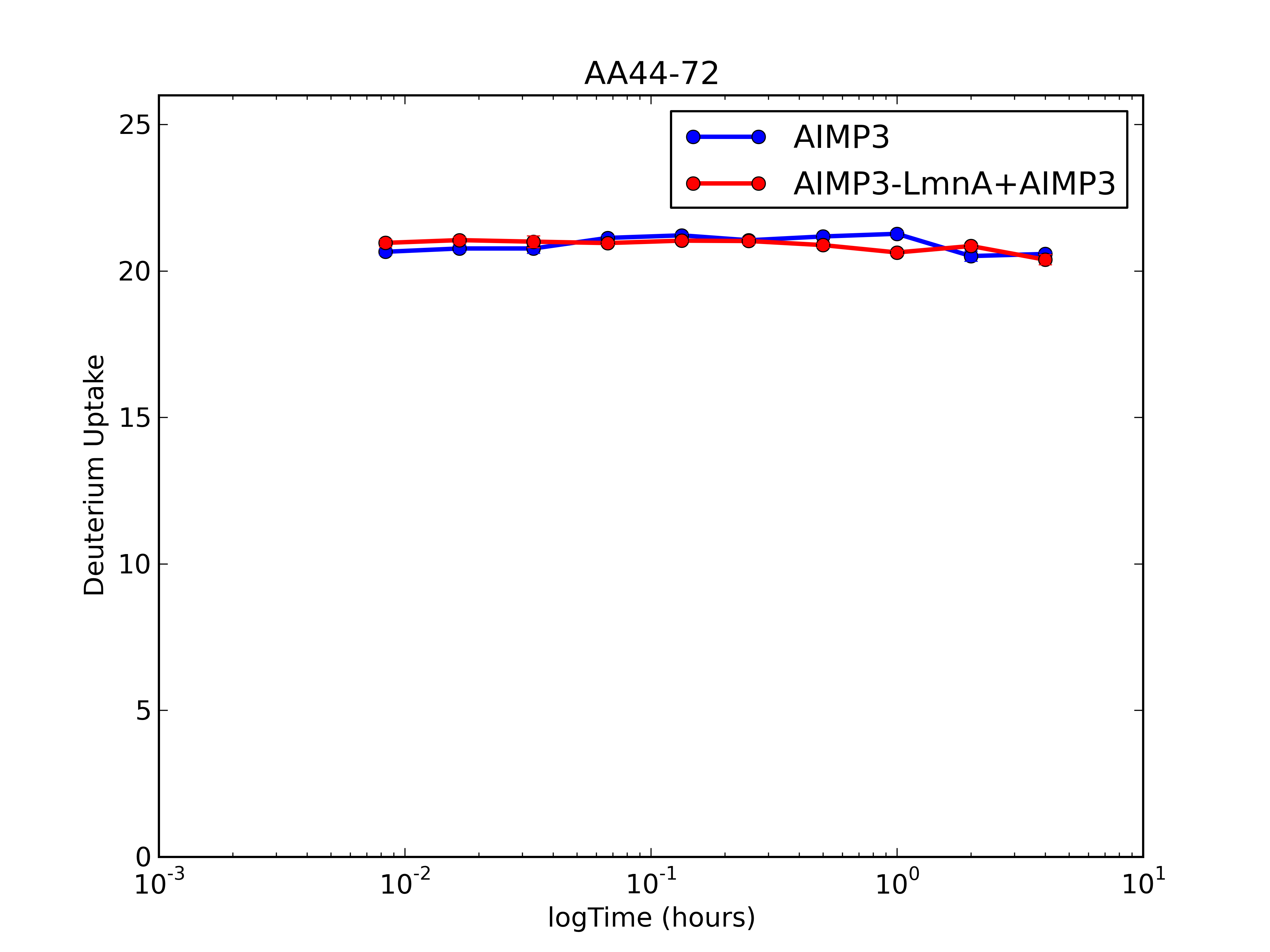

Supplement: S2 File — (ZIP) [file pone.0181869.s004.zip › logfigure-LmnA-scale/AA44-72_charge_3_mz1109.1.csv.csv.png]

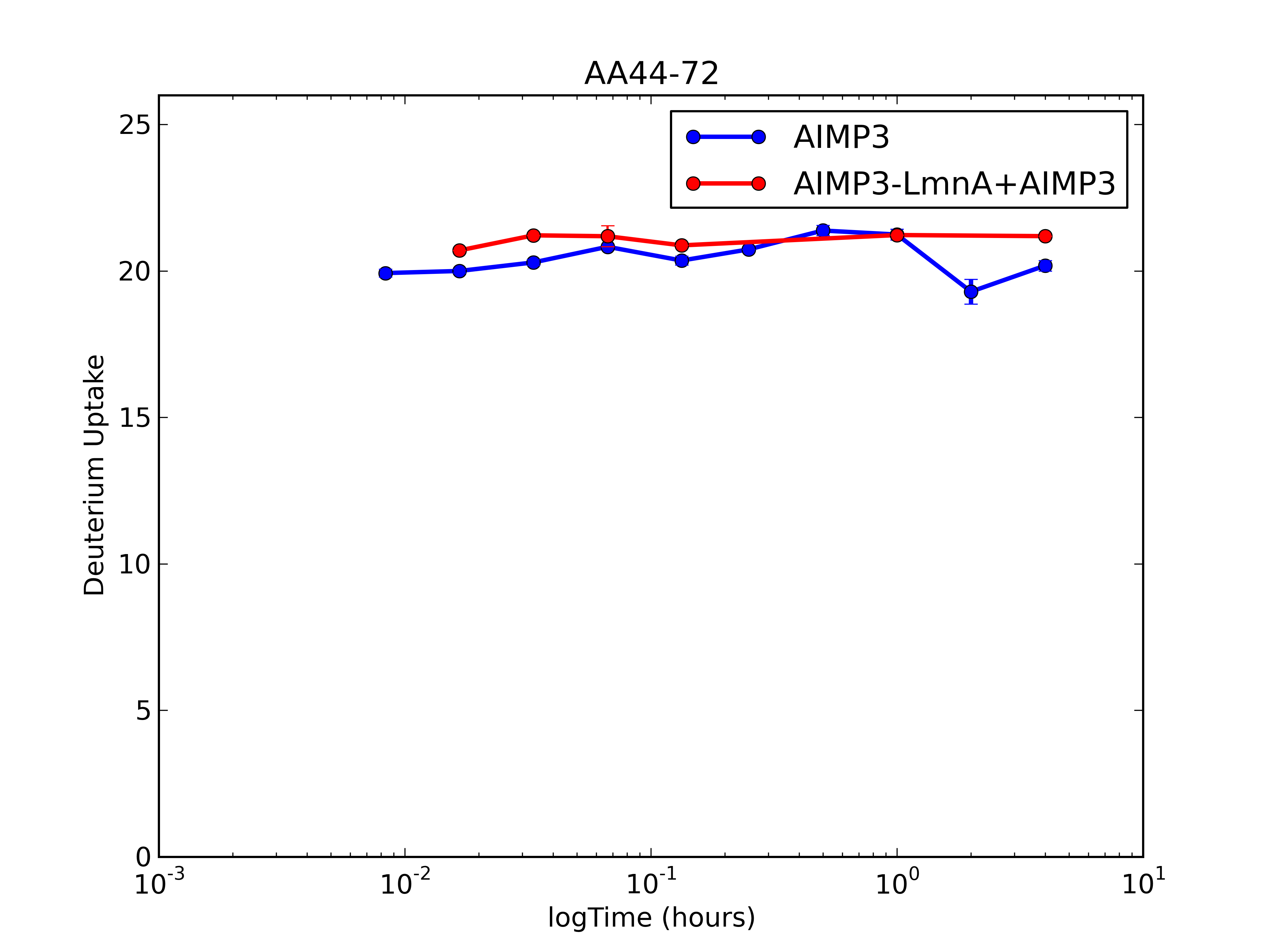

Supplement: S2 File — (ZIP) [file pone.0181869.s004.zip › logfigure-LmnA-scale/AA44-72_charge_4_mz832.1.csv.csv.png]

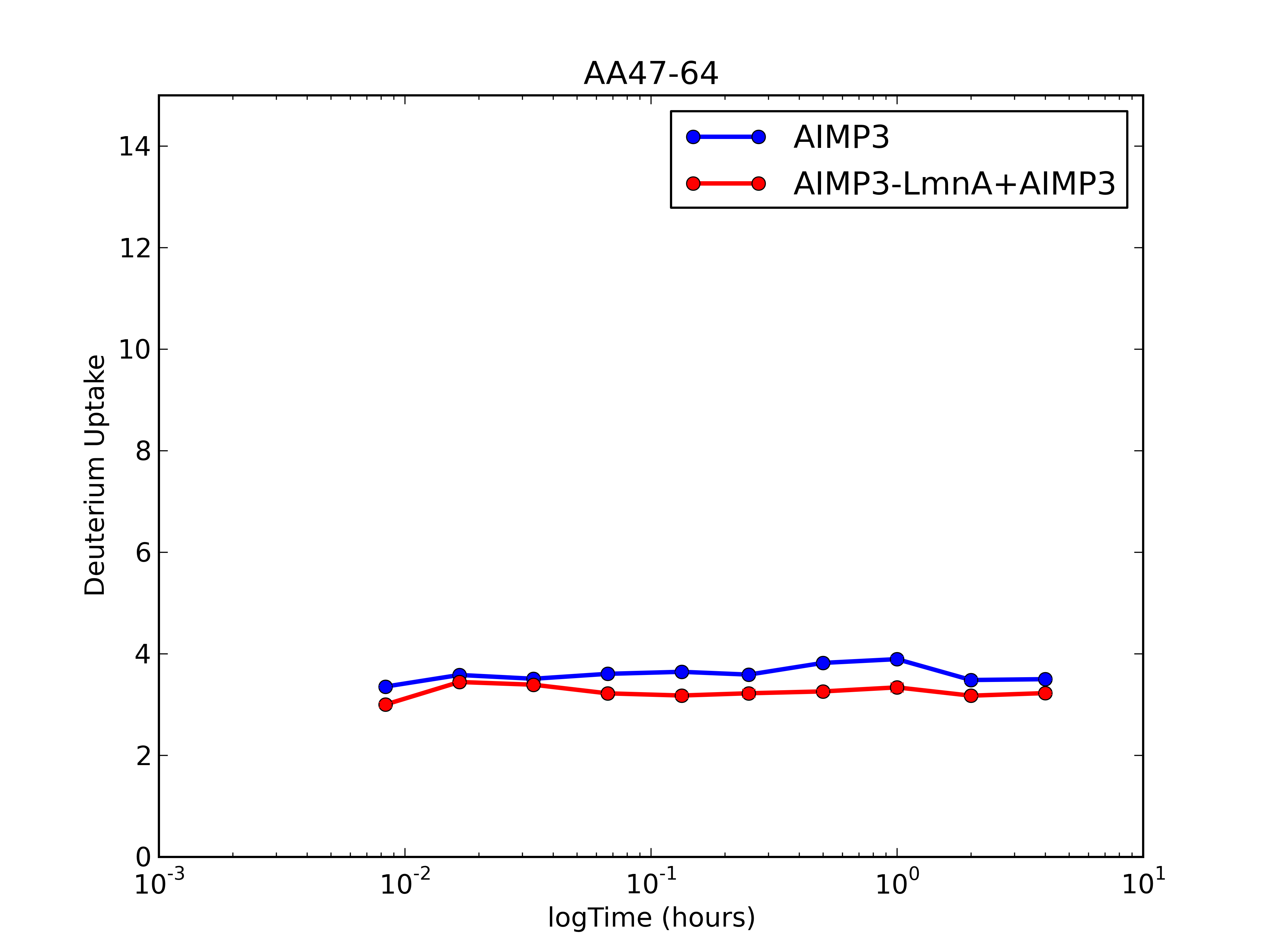

Supplement: S2 File — (ZIP) [file pone.0181869.s004.zip › logfigure-LmnA-scale/AA47-64_charge_4_mz524.4.csv.csv.png]

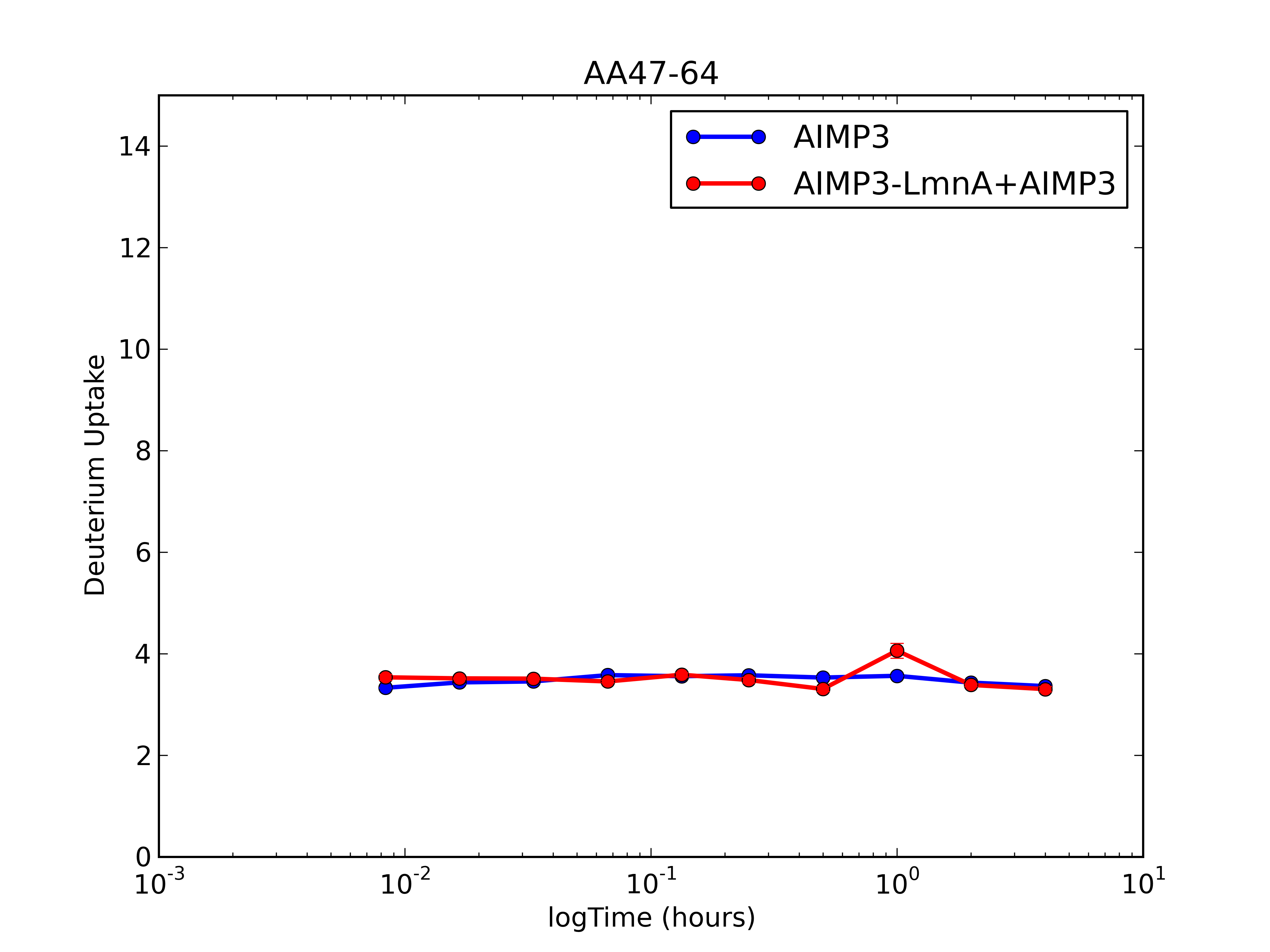

Supplement: S2 File — (ZIP) [file pone.0181869.s004.zip › logfigure-LmnA-scale/AA47-64_charge_5_mz419.7.csv.csv.png]

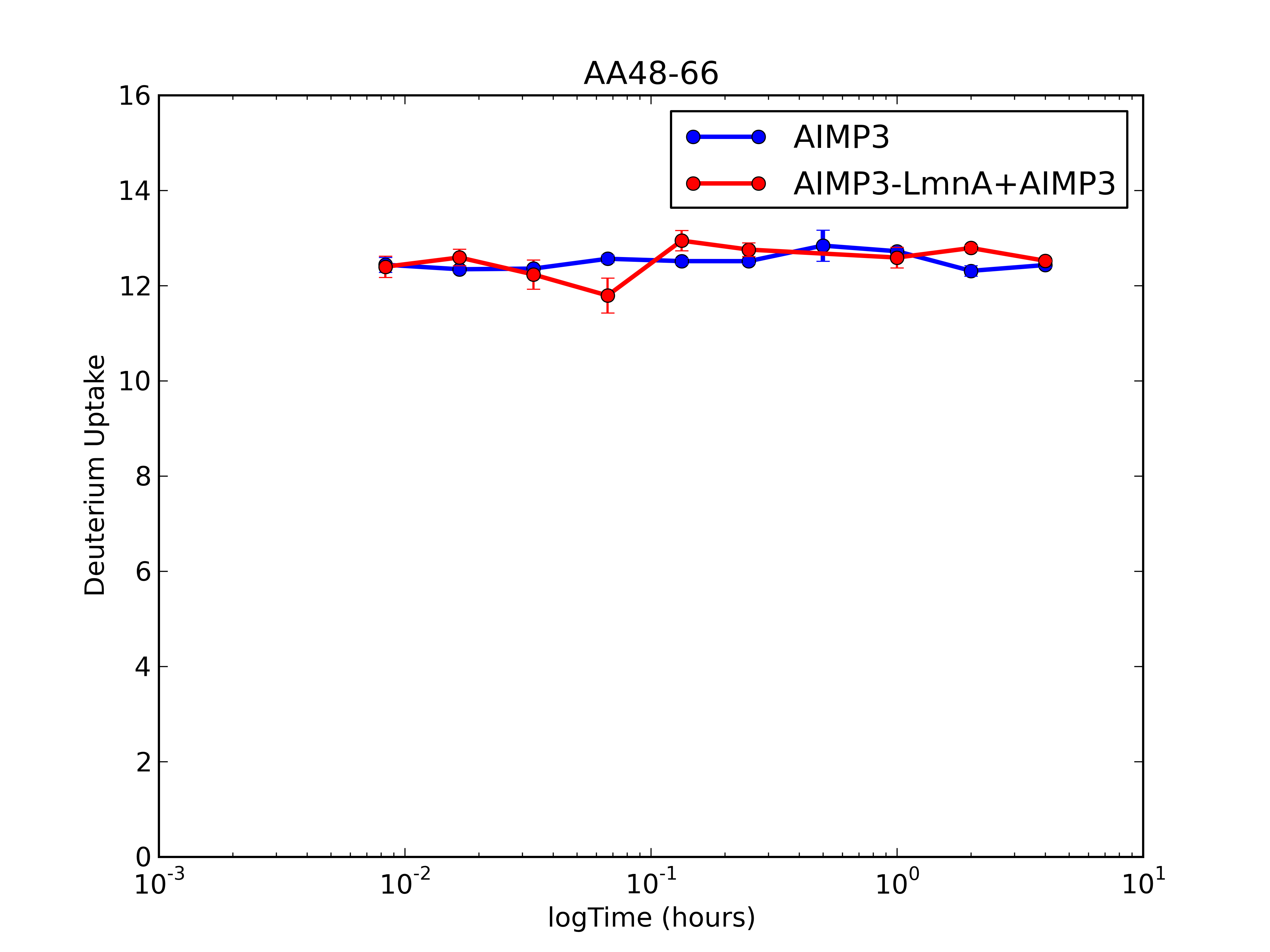

Supplement: S2 File — (ZIP) [file pone.0181869.s004.zip › logfigure-LmnA-scale/AA48-66_charge_2_mz1111.5.csv.csv.png]

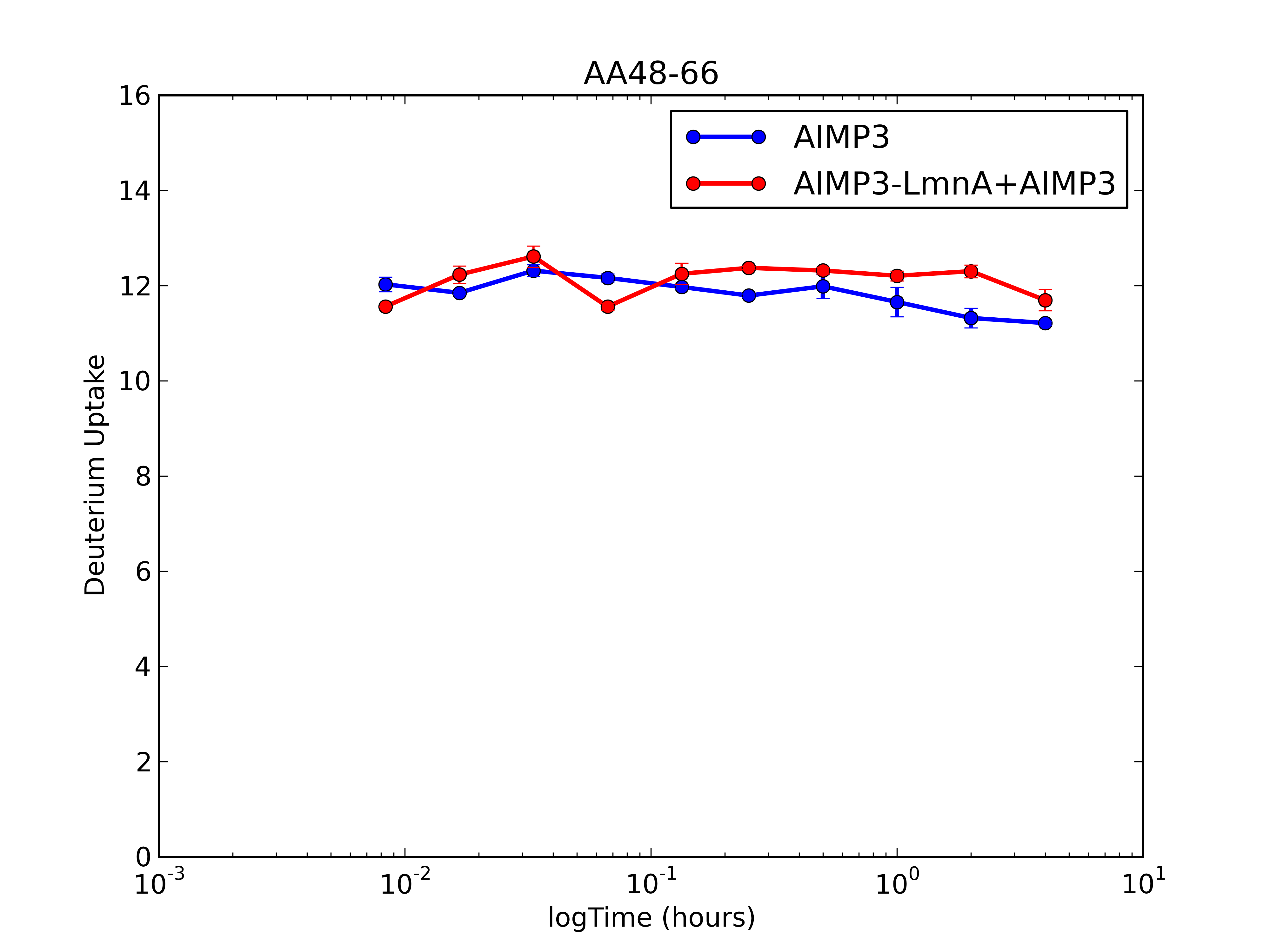

Supplement: S2 File — (ZIP) [file pone.0181869.s004.zip › logfigure-LmnA-scale/AA48-66_charge_3_mz741.3.csv.csv.png]

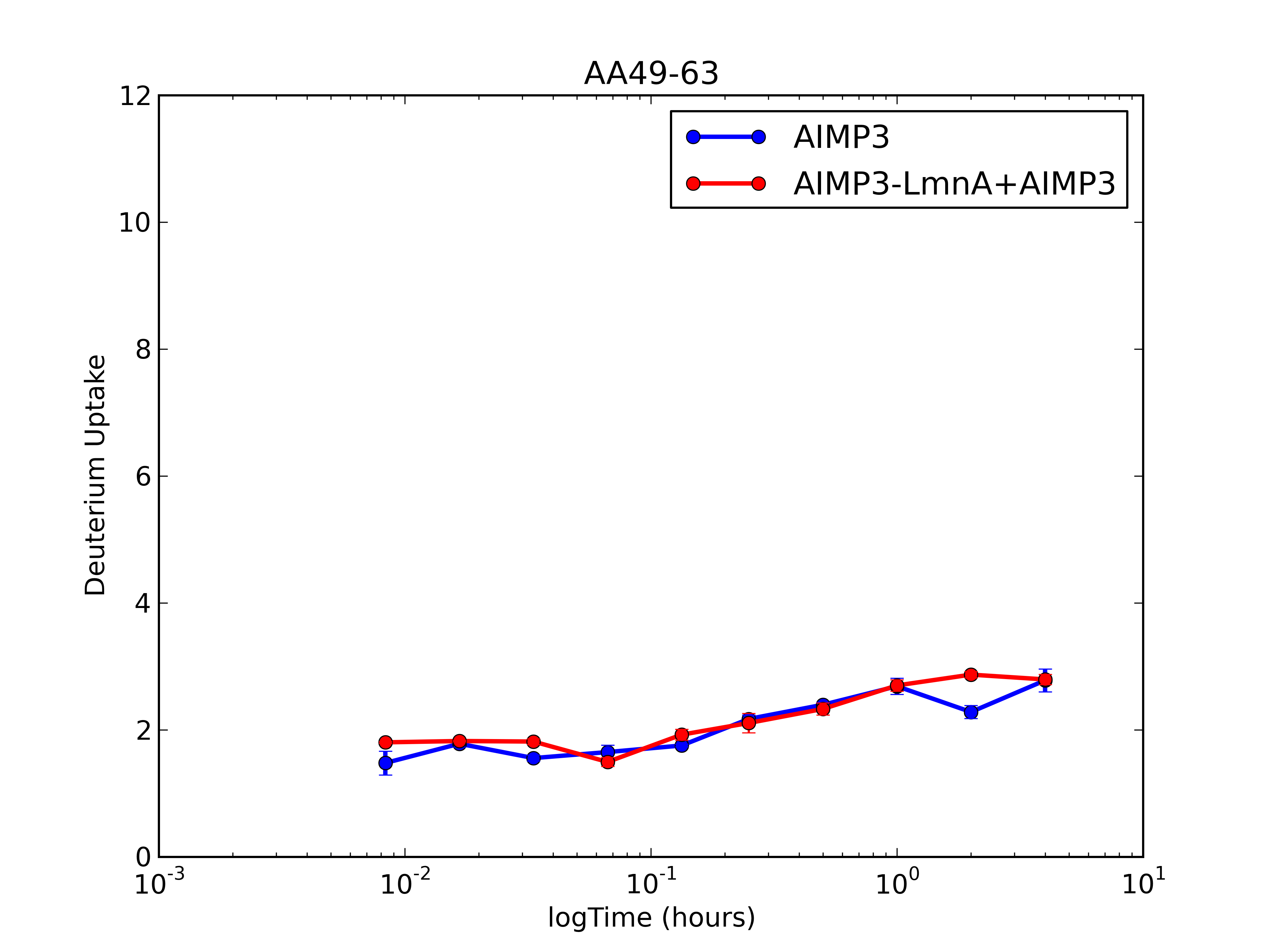

Supplement: S2 File — (ZIP) [file pone.0181869.s004.zip › logfigure-LmnA-scale/AA49-63_charge_2_mz859.4.csv.csv.png]

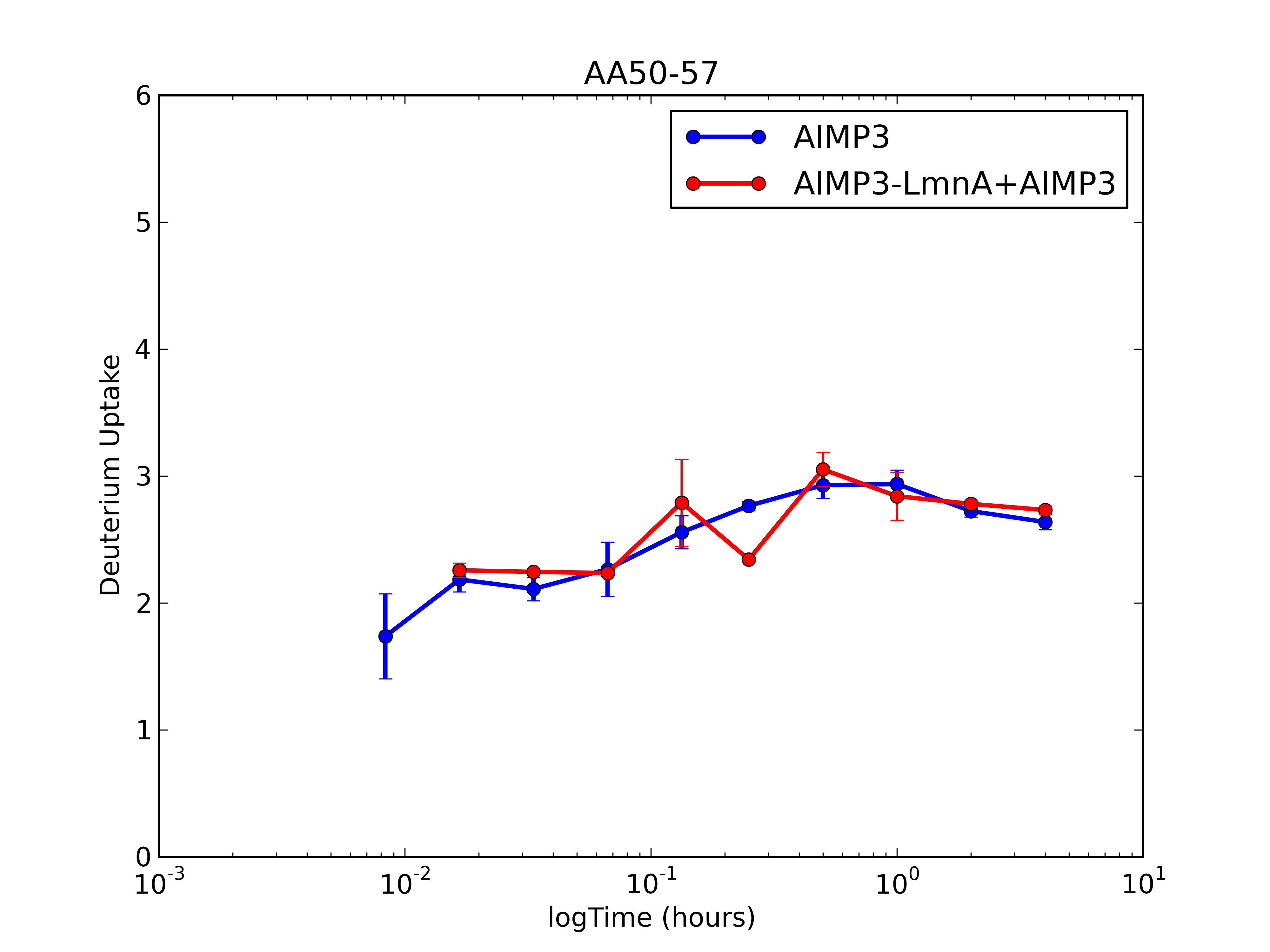

Supplement: S2 File — (ZIP) [file pone.0181869.s004.zip › logfigure-LmnA-scale/AA50-57_charge_2_mz447.1.csv.csv.png]

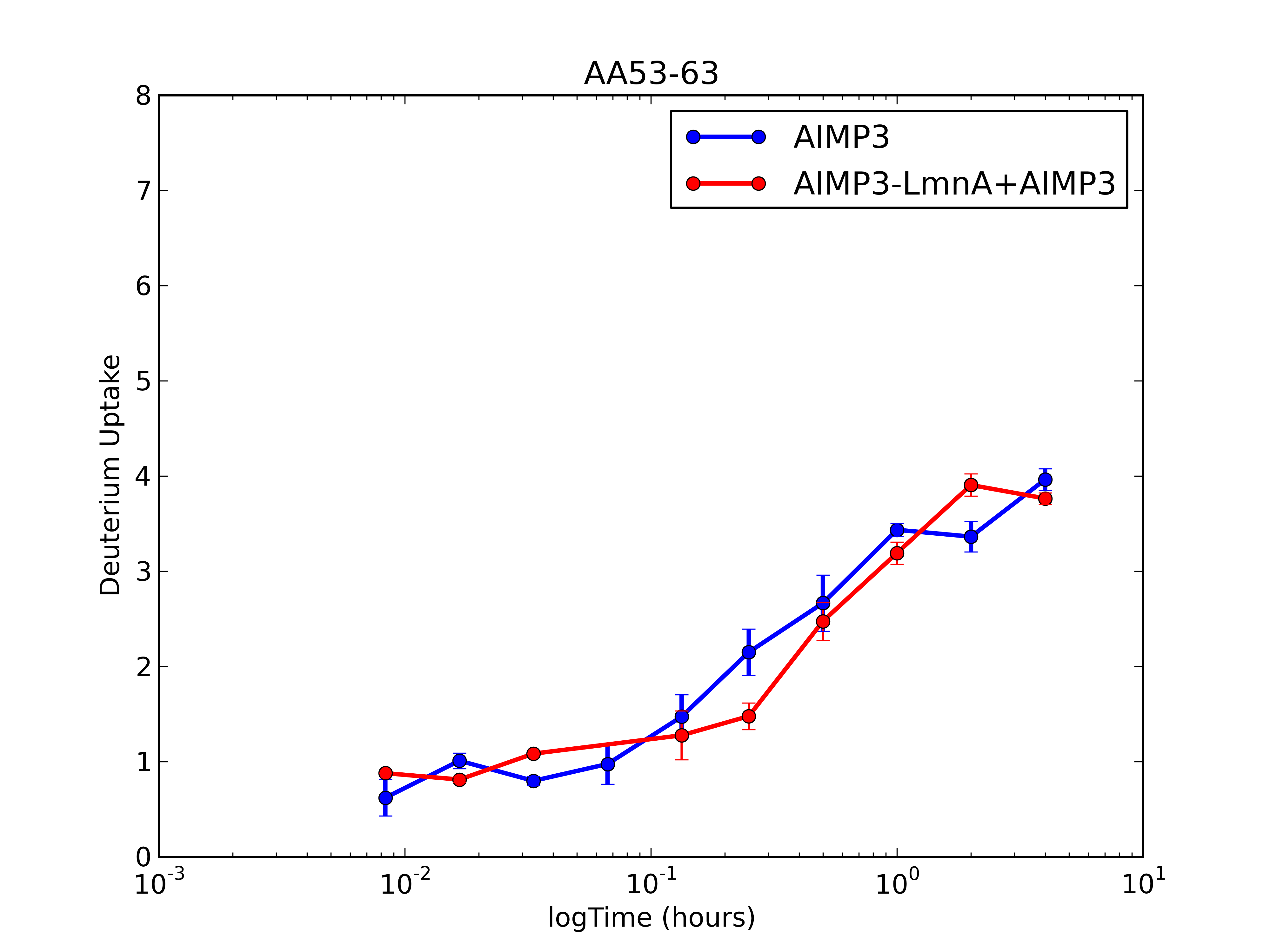

Supplement: S2 File — (ZIP) [file pone.0181869.s004.zip › logfigure-LmnA-scale/AA53-63_charge_2_mz573.2.csv.csv.png]

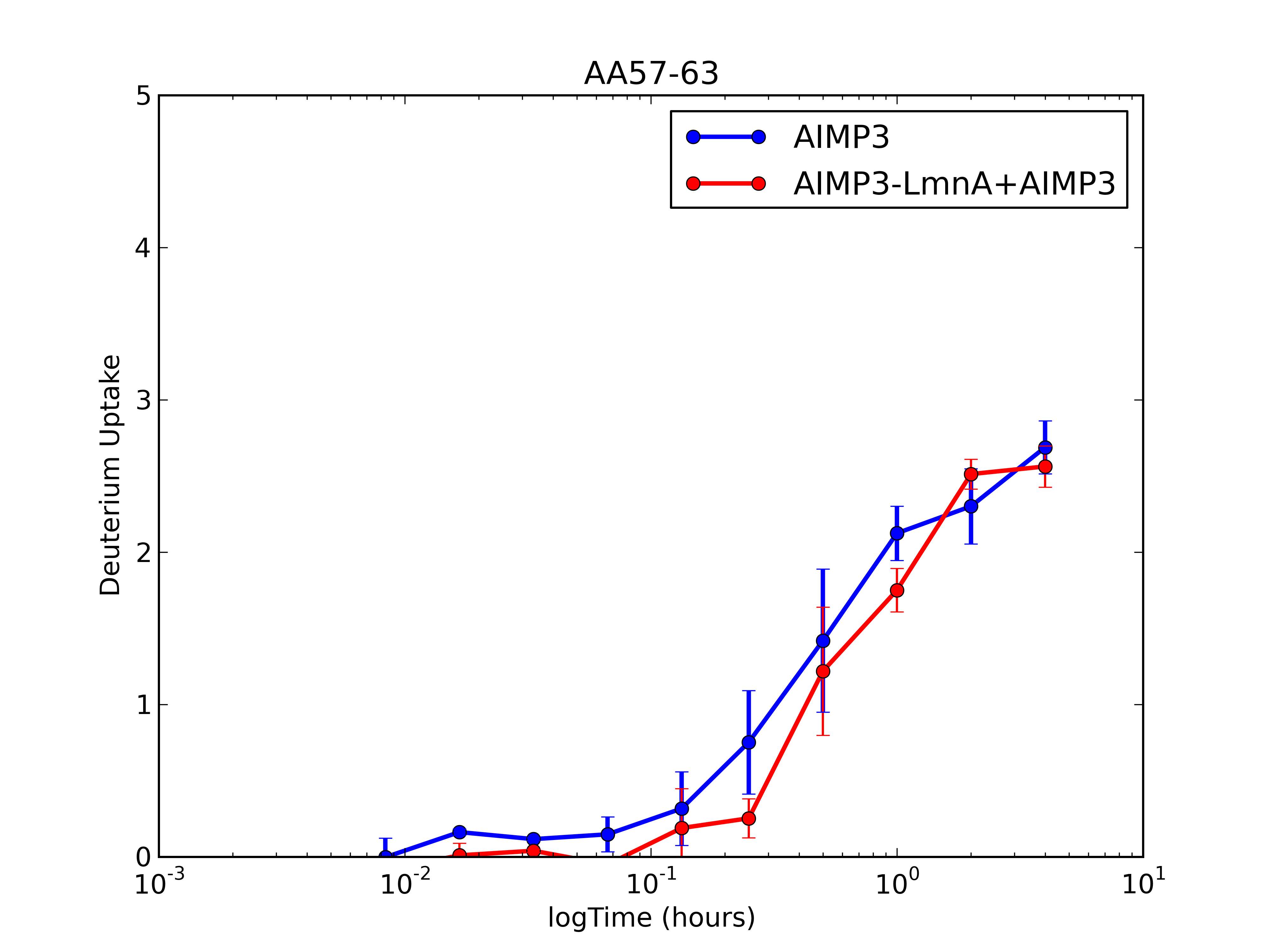

Supplement: S2 File — (ZIP) [file pone.0181869.s004.zip › logfigure-LmnA-scale/AA57-63_charge_1_mz785.4.csv.csv.png]

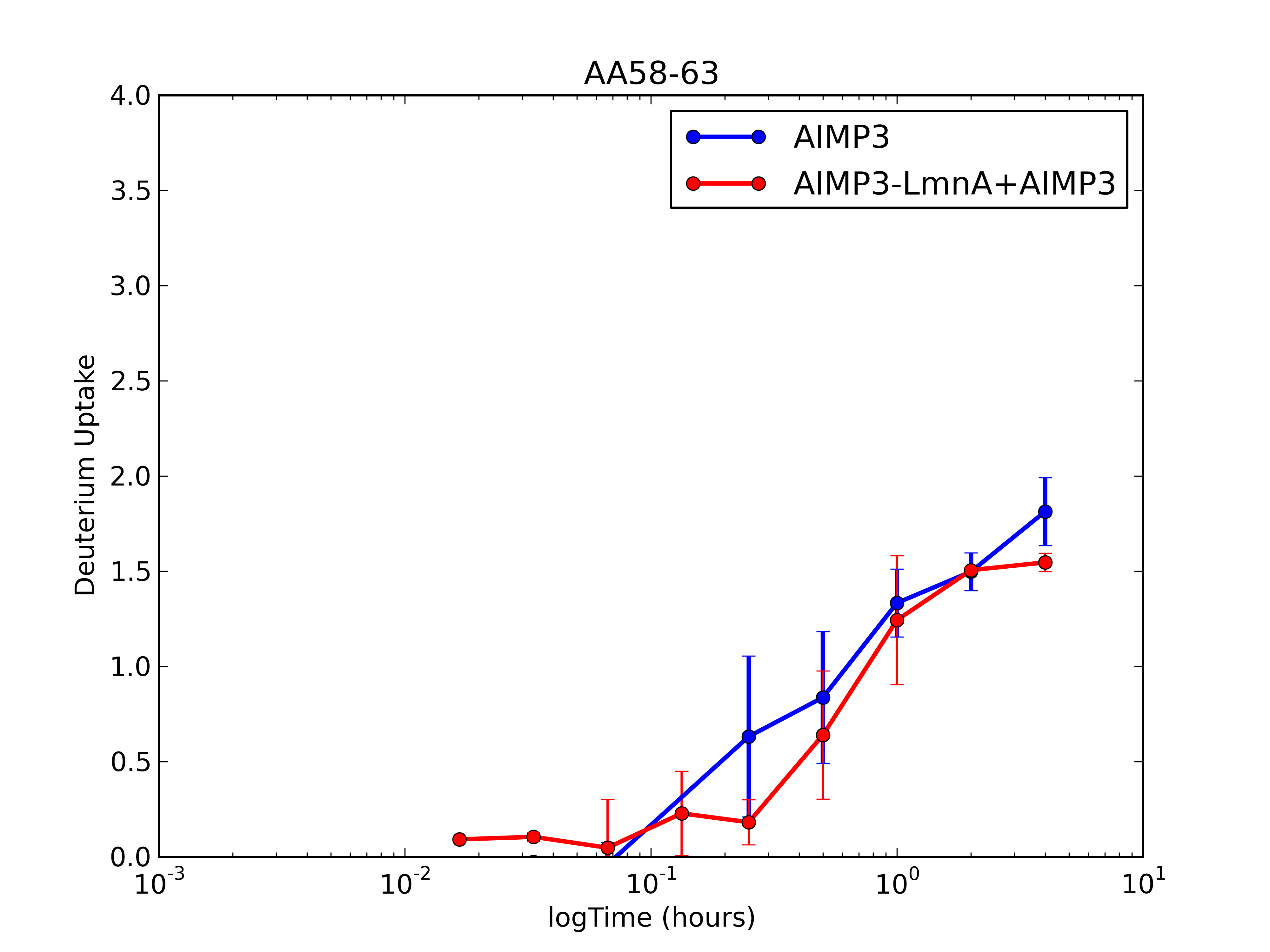

Supplement: S2 File — (ZIP) [file pone.0181869.s004.zip › logfigure-LmnA-scale/AA58-63_charge_1_mz657.4.csv.csv.png]

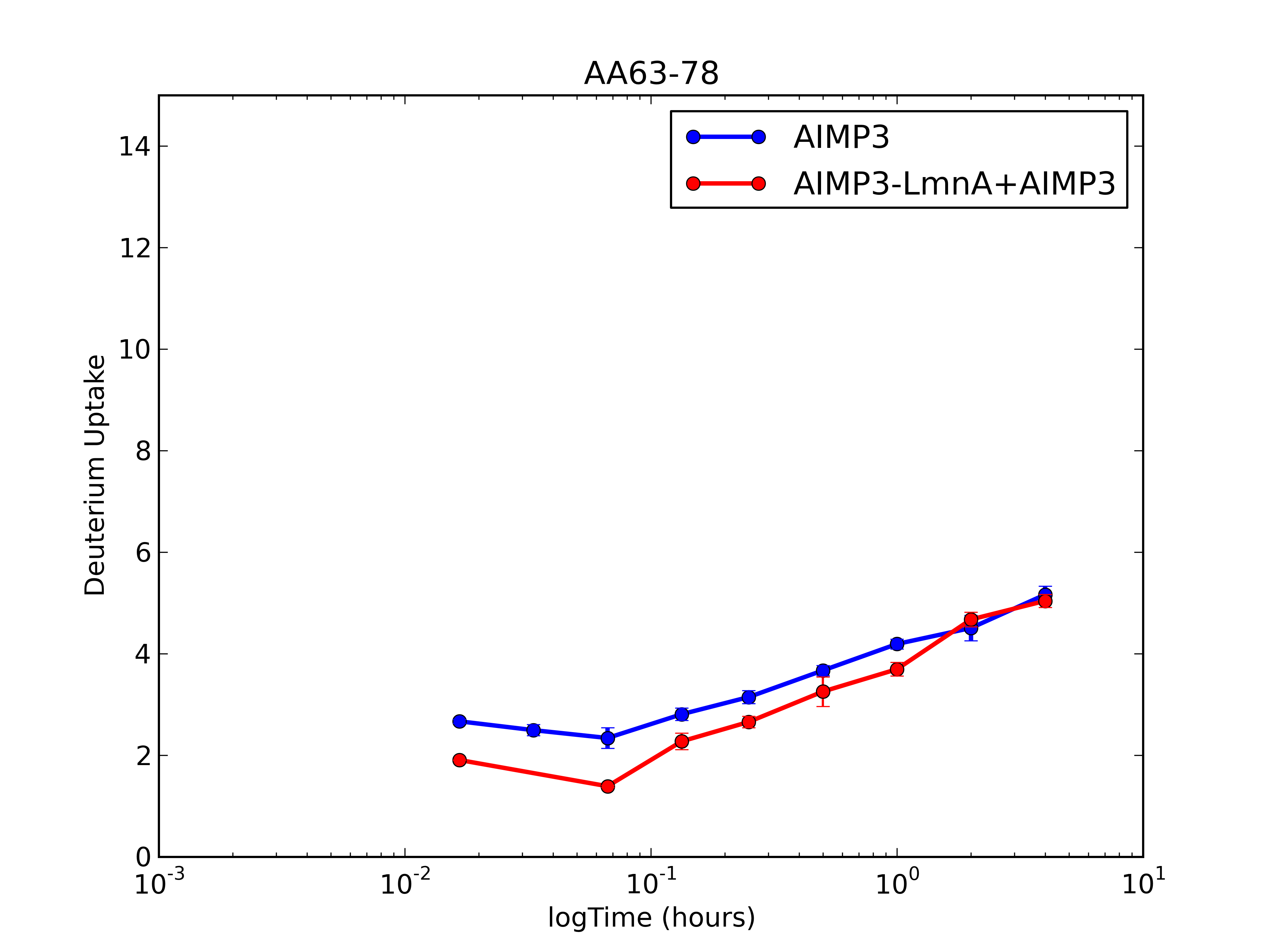

Supplement: S2 File — (ZIP) [file pone.0181869.s004.zip › logfigure-LmnA-scale/AA63-78_charge_2_mz896.9.csv.csv.png]

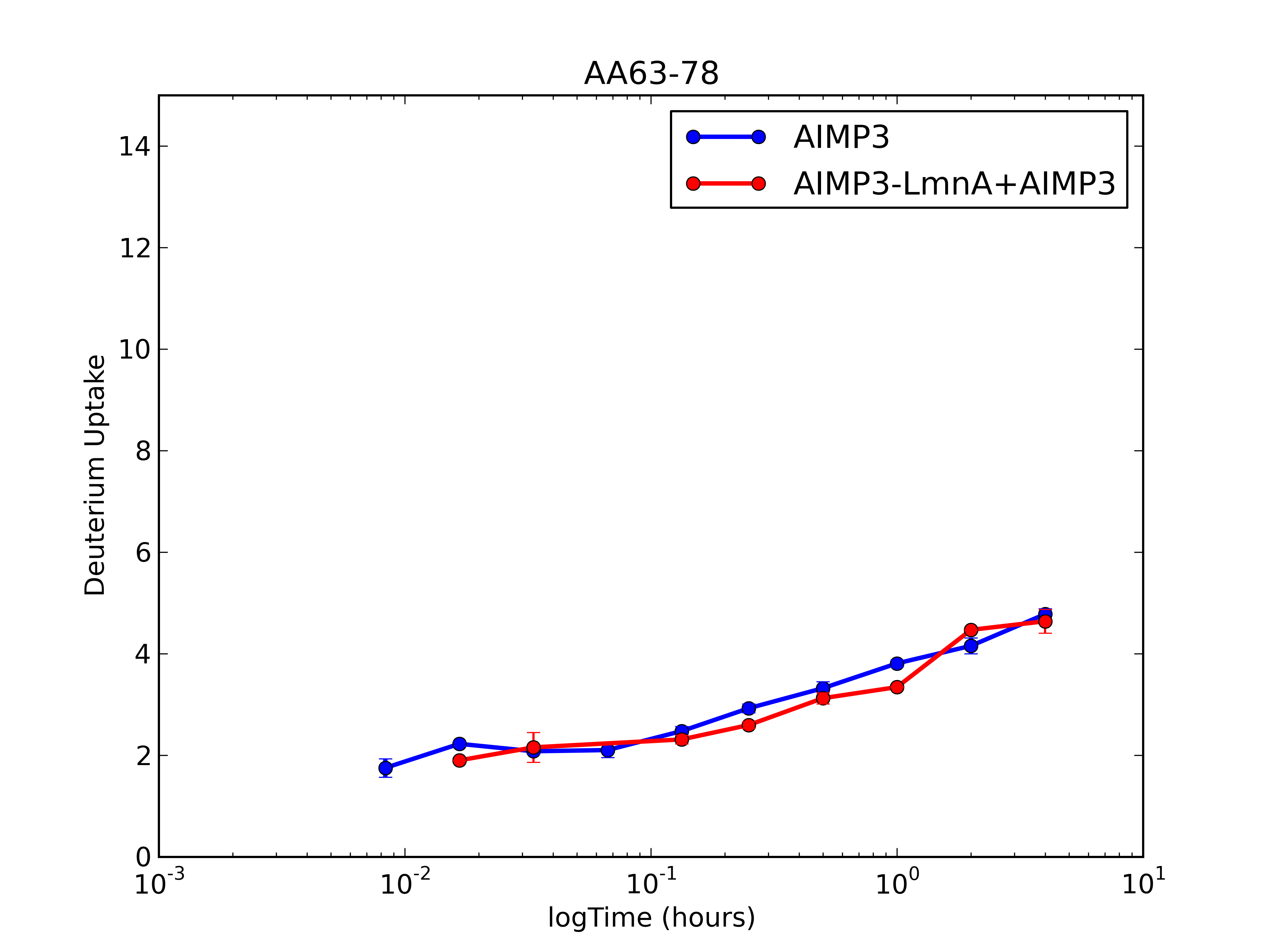

Supplement: S2 File — (ZIP) [file pone.0181869.s004.zip › logfigure-LmnA-scale/AA63-78_charge_3_mz598.3.csv.csv.png]

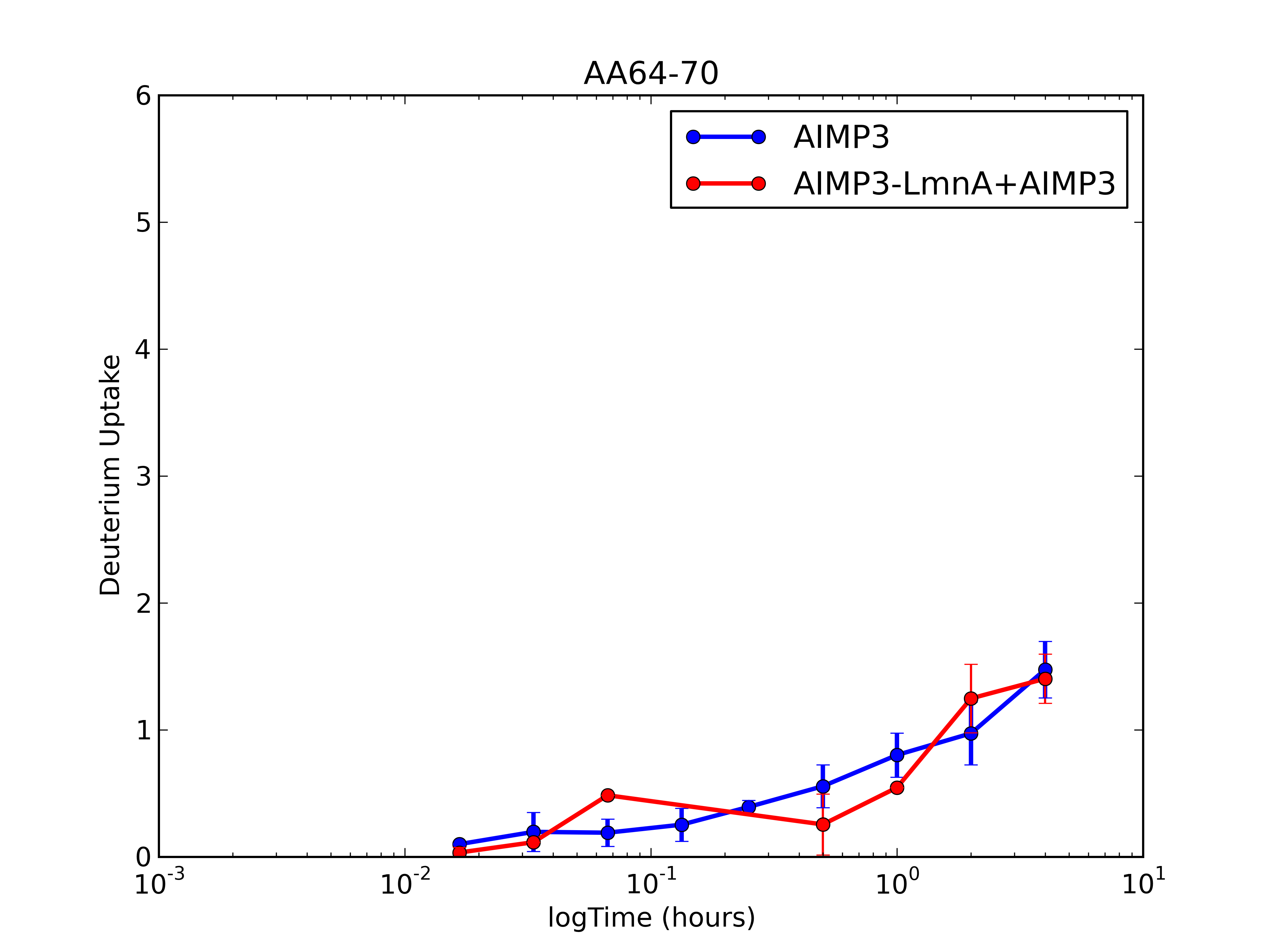

Supplement: S2 File — (ZIP) [file pone.0181869.s004.zip › logfigure-LmnA-scale/AA64-70_charge_1_mz854.3.csv.csv.png]

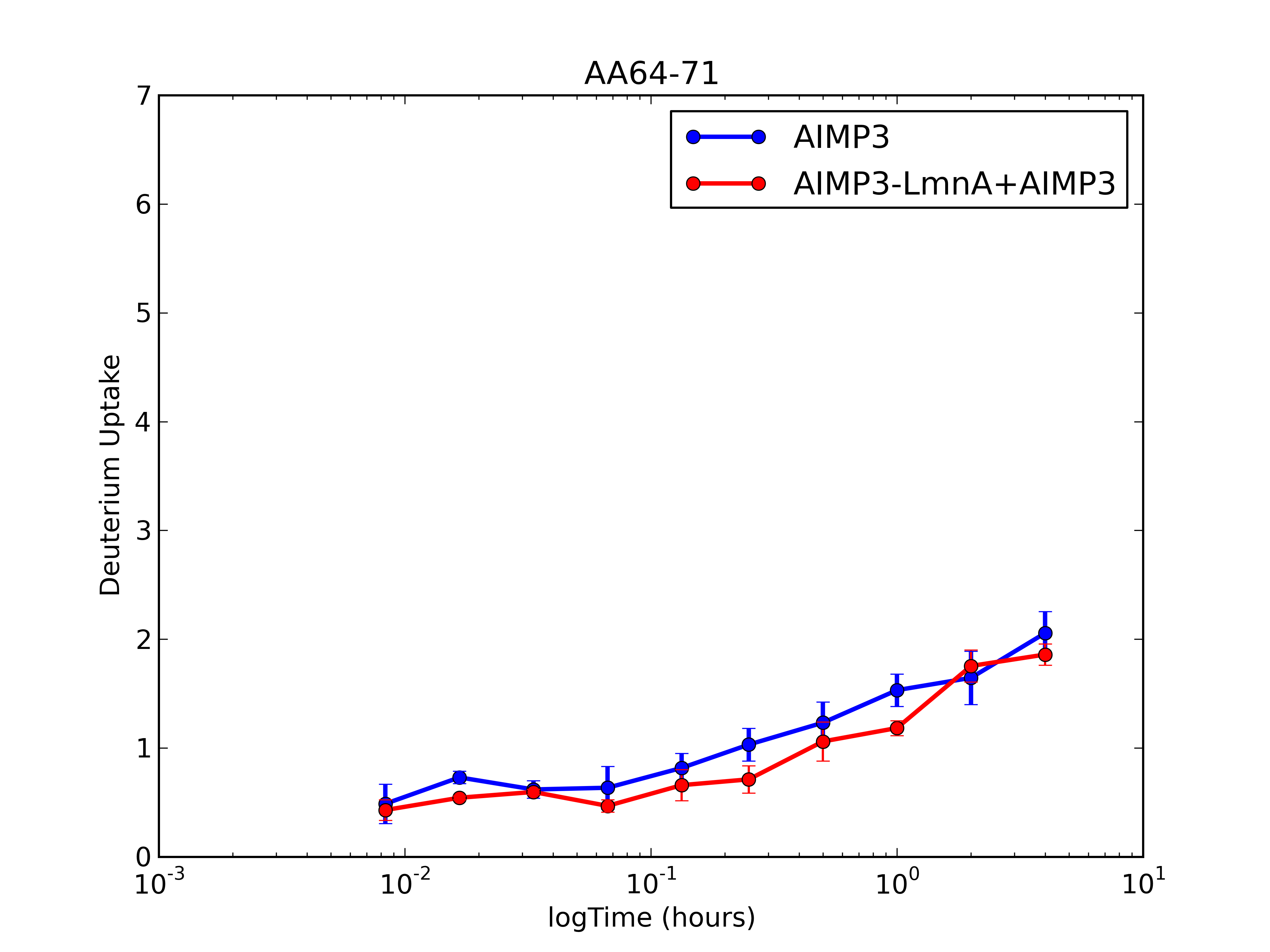

Supplement: S2 File — (ZIP) [file pone.0181869.s004.zip › logfigure-LmnA-scale/AA64-71_charge_1_mz982.3.csv.csv.png]

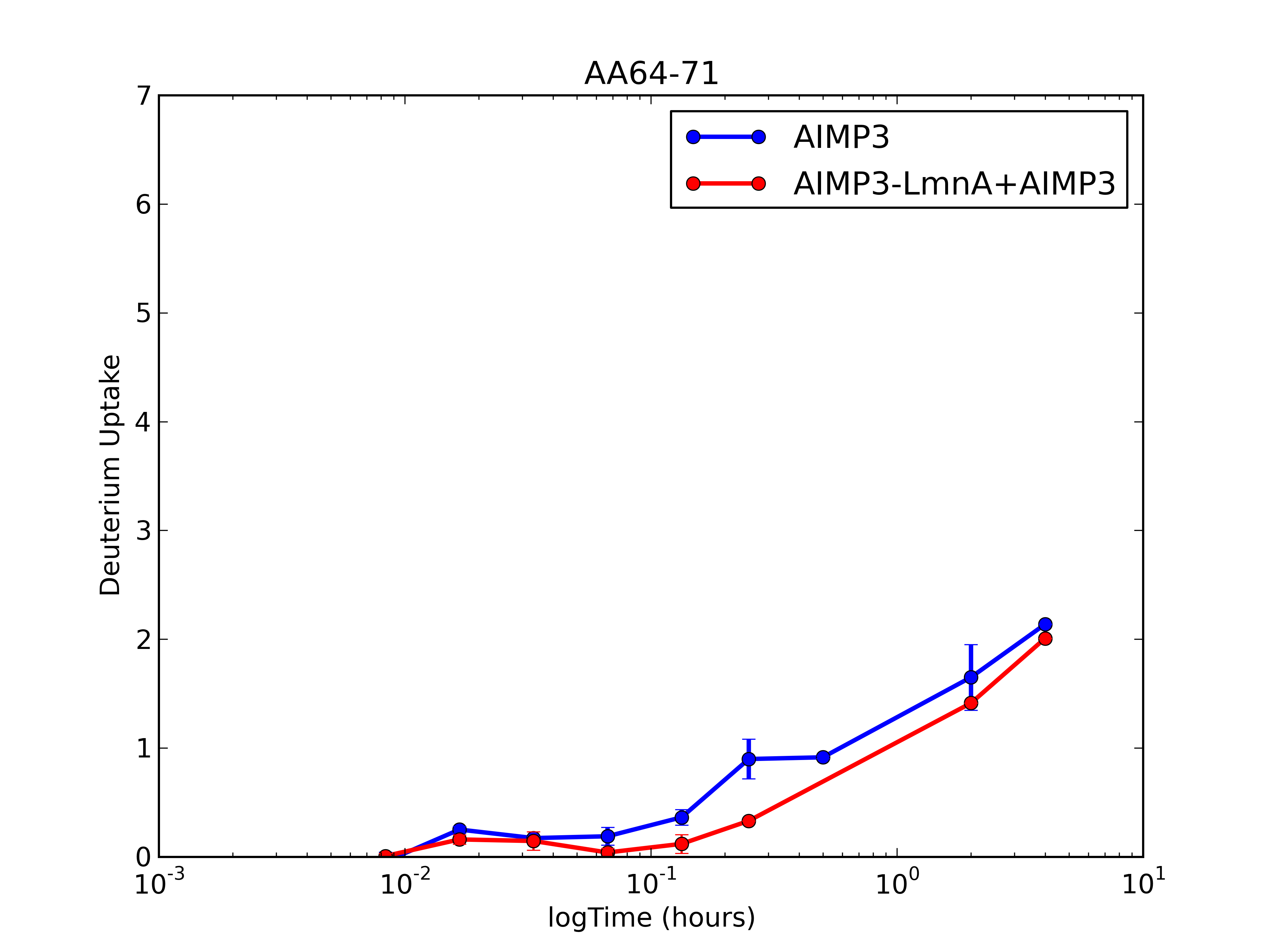

Supplement: S2 File — (ZIP) [file pone.0181869.s004.zip › logfigure-LmnA-scale/AA64-71_charge_2_mz491.7.csv.csv.png]

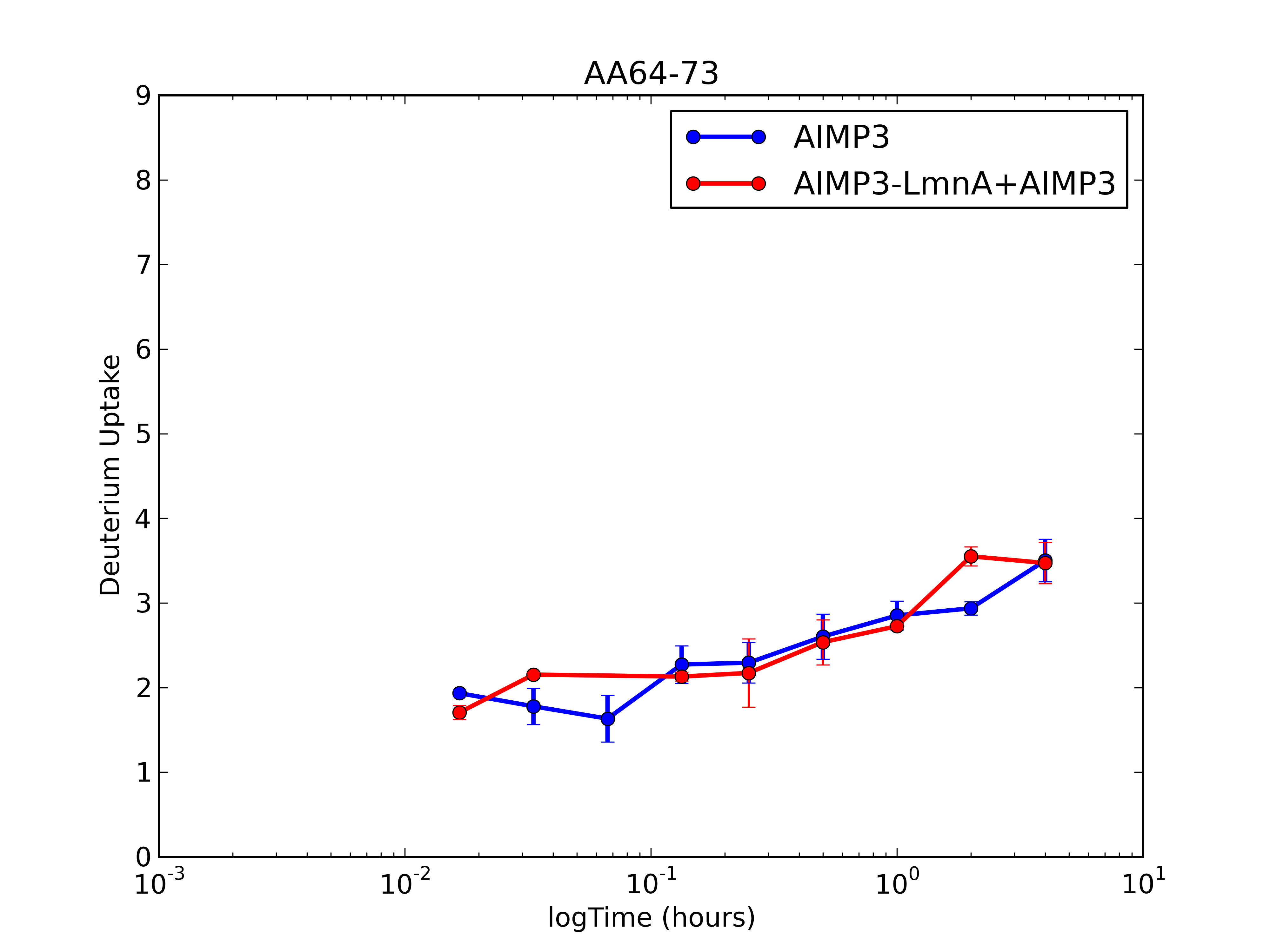

Supplement: S2 File — (ZIP) [file pone.0181869.s004.zip › logfigure-LmnA-scale/AA64-73_charge_1_mz1167.5.csv.csv.png]

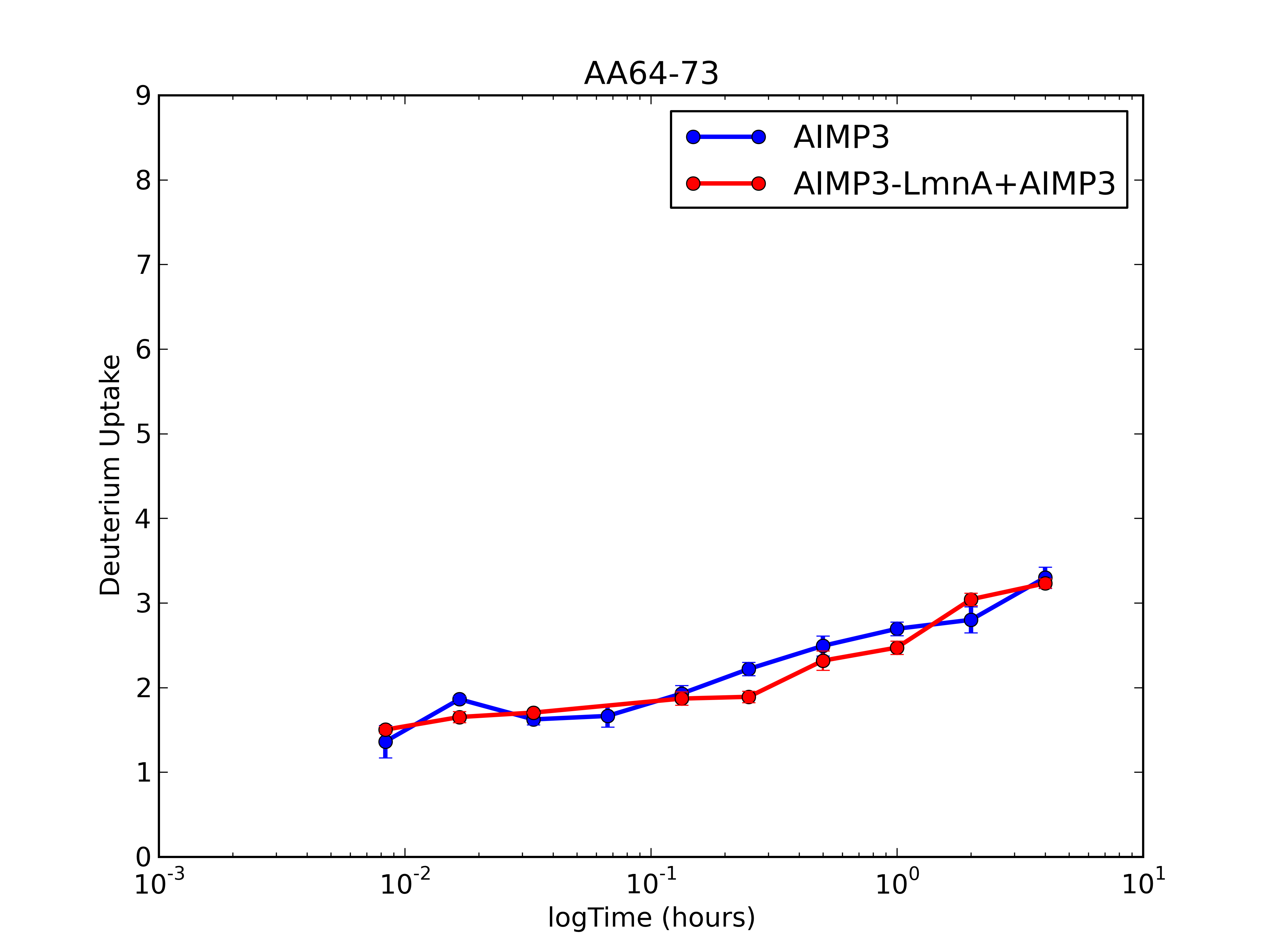

Supplement: S2 File — (ZIP) [file pone.0181869.s004.zip › logfigure-LmnA-scale/AA64-73_charge_2_mz584.2.csv.csv.png]

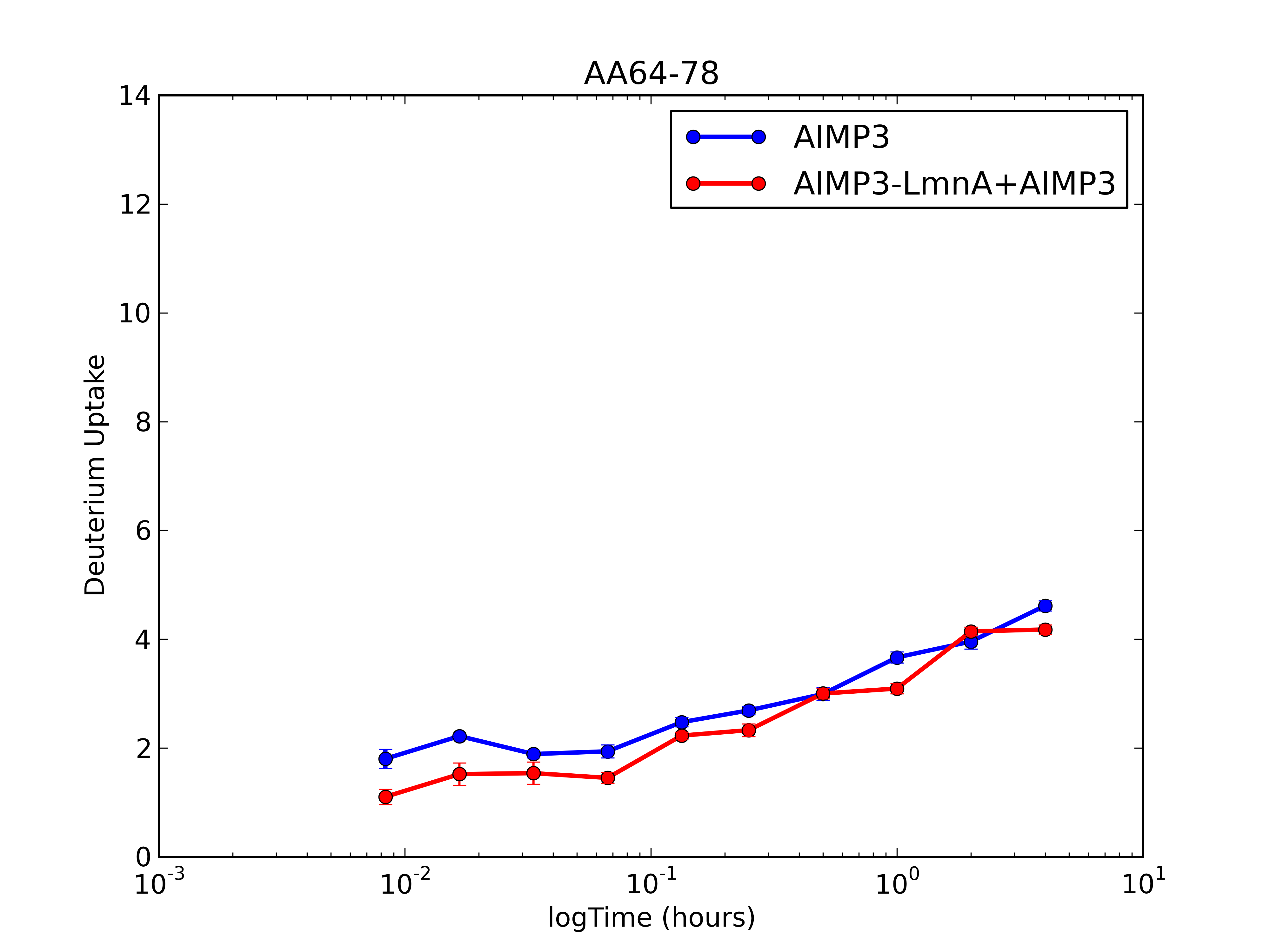

Supplement: S2 File — (ZIP) [file pone.0181869.s004.zip › logfigure-LmnA-scale/AA64-78_charge_3_mz560.6.csv.csv.png]

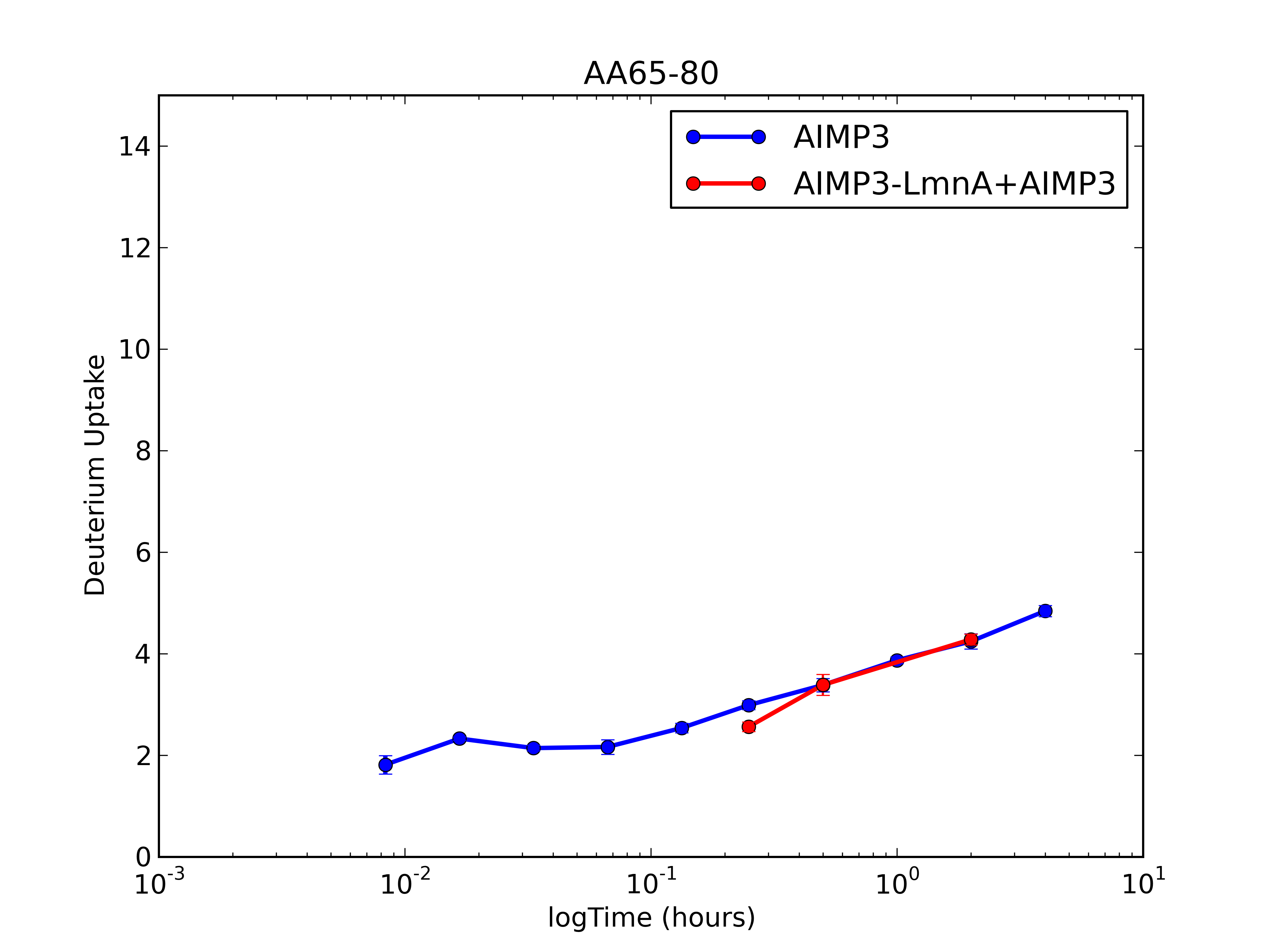

Supplement: S2 File — (ZIP) [file pone.0181869.s004.zip › logfigure-LmnA-scale/AA65-80_charge_3_mz597.9.csv.csv.png]

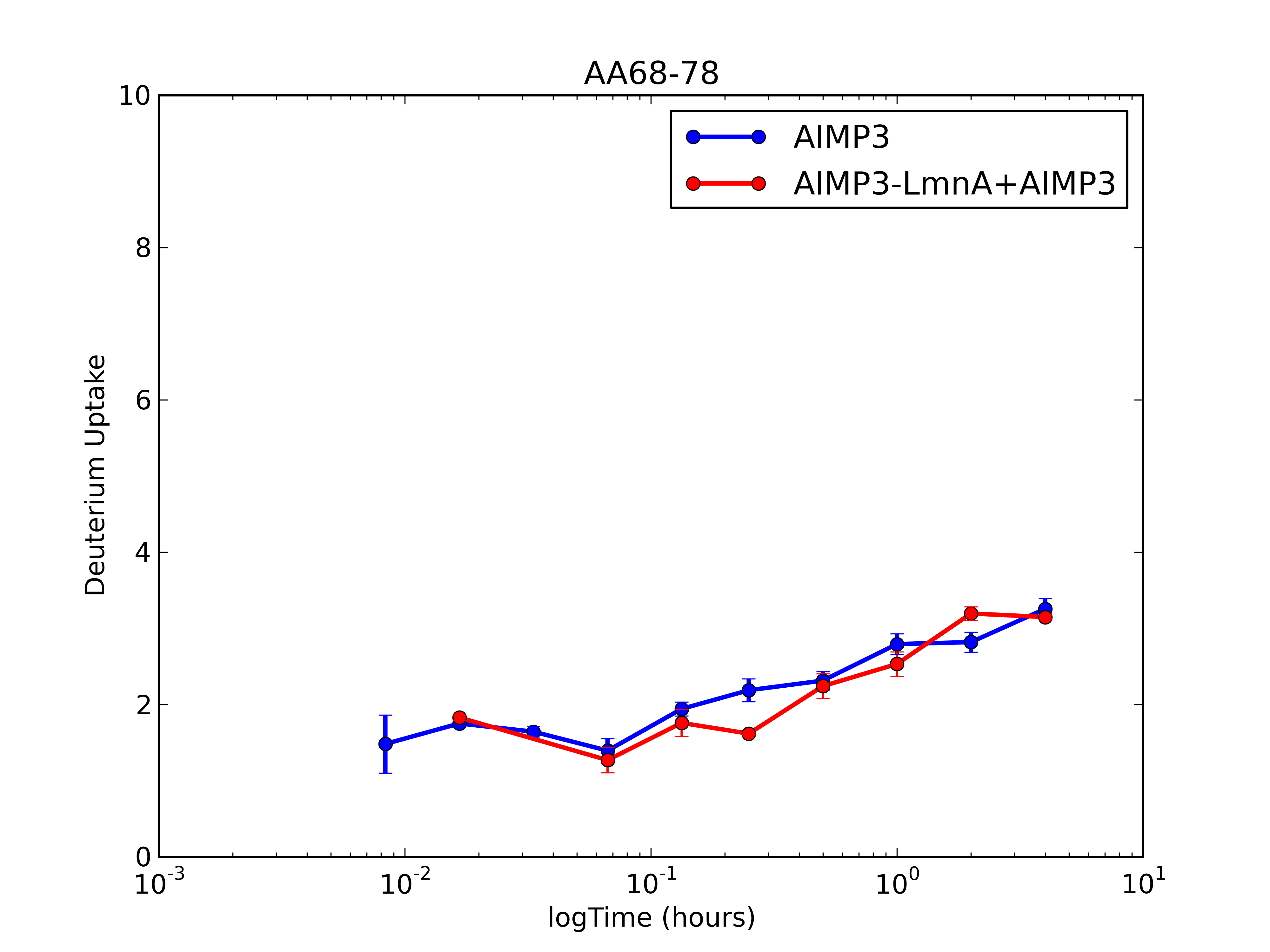

Supplement: S2 File — (ZIP) [file pone.0181869.s004.zip › logfigure-LmnA-scale/AA68-78_charge_2_mz626.3.csv.csv.png]

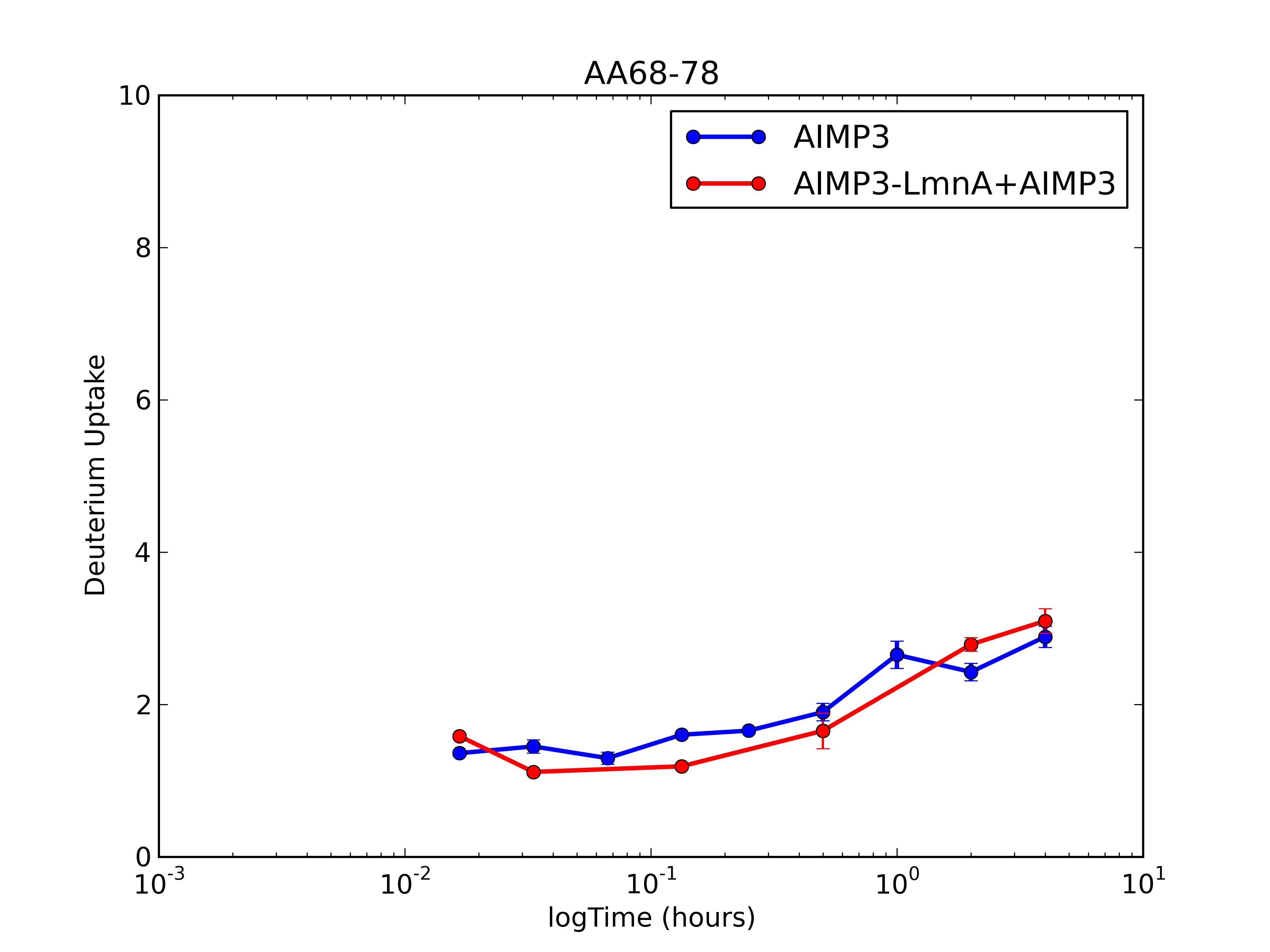

Supplement: S2 File — (ZIP) [file pone.0181869.s004.zip › logfigure-LmnA-scale/AA68-78_charge_3_mz417.8.csv.csv.png]

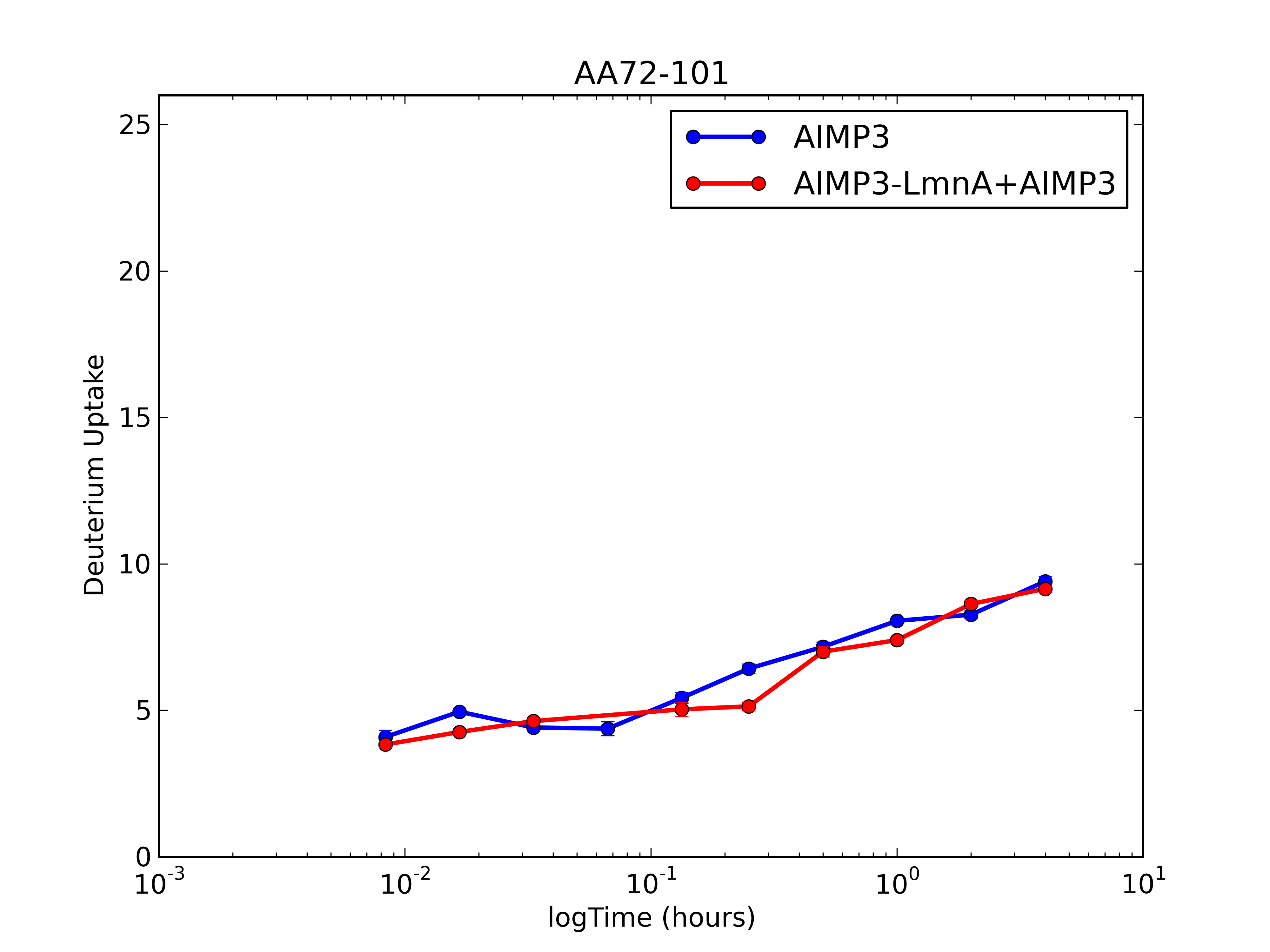

Supplement: S2 File — (ZIP) [file pone.0181869.s004.zip › logfigure-LmnA-scale/AA72-101_charge_4_mz791.2.csv.csv.png]

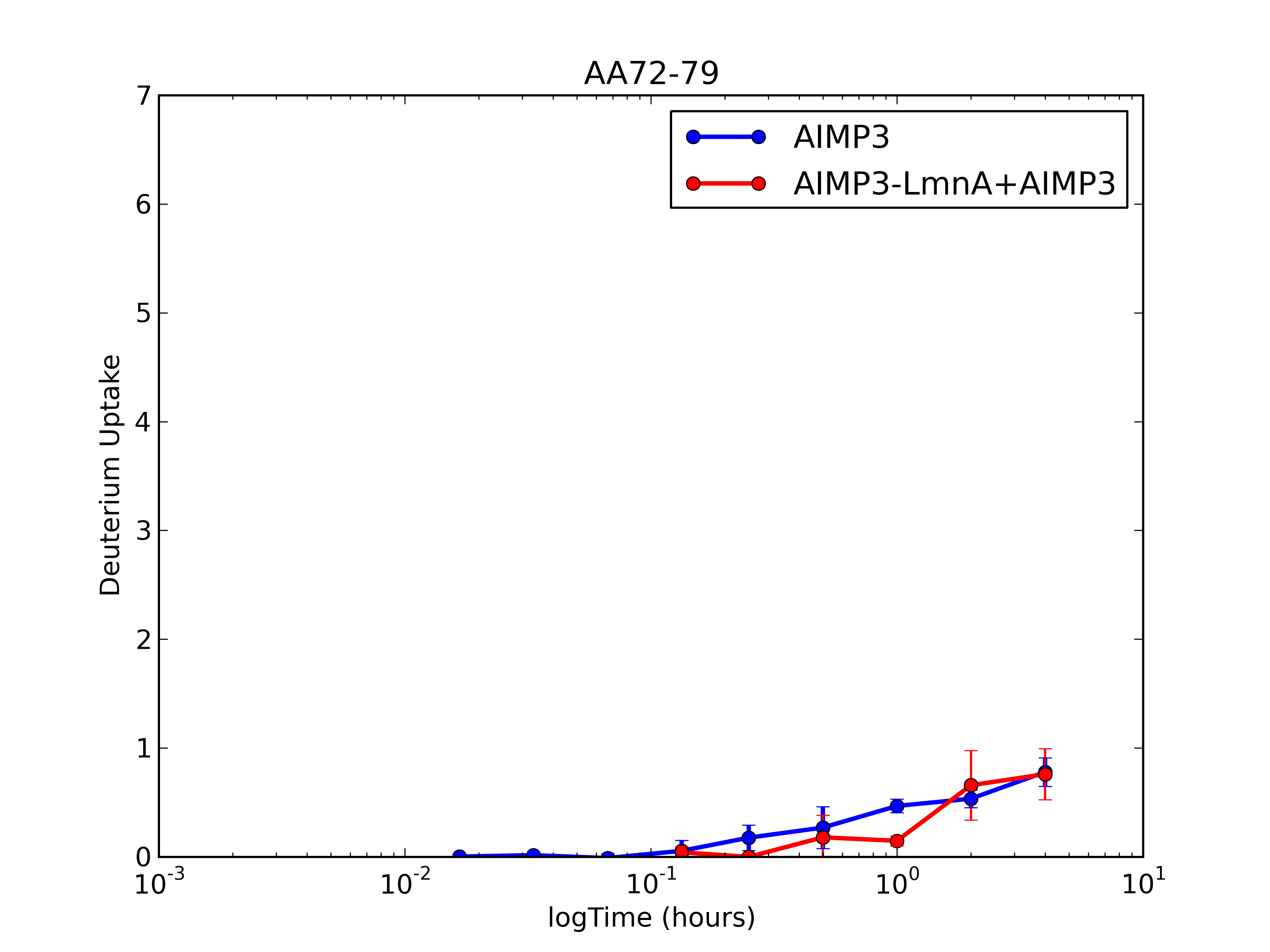

Supplement: S2 File — (ZIP) [file pone.0181869.s004.zip › logfigure-LmnA-scale/AA72-79_charge_1_mz829.5.csv.csv.png]

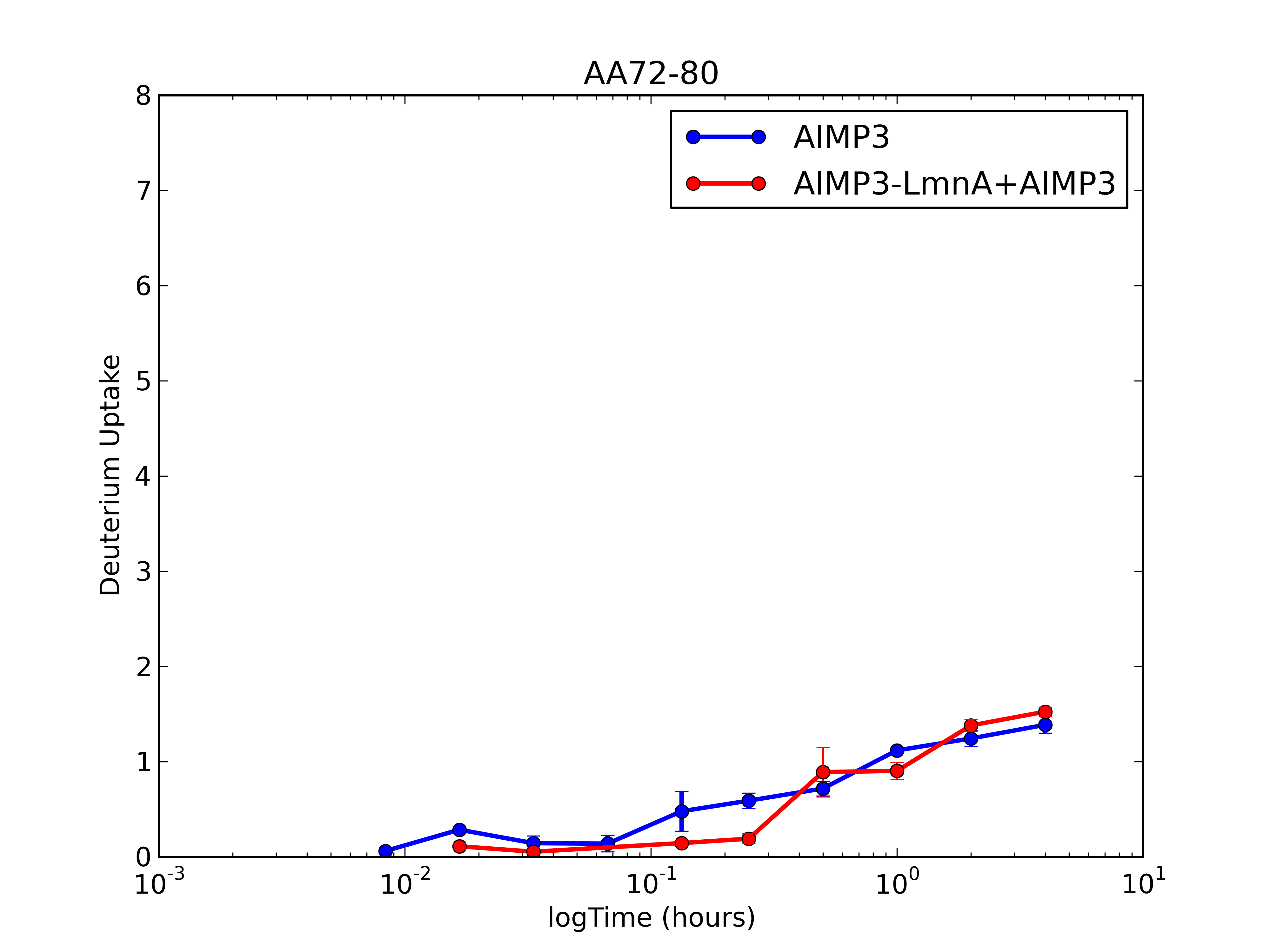

Supplement: S2 File — (ZIP) [file pone.0181869.s004.zip › logfigure-LmnA-scale/AA72-80_charge_2_mz472.3.csv.csv.png]

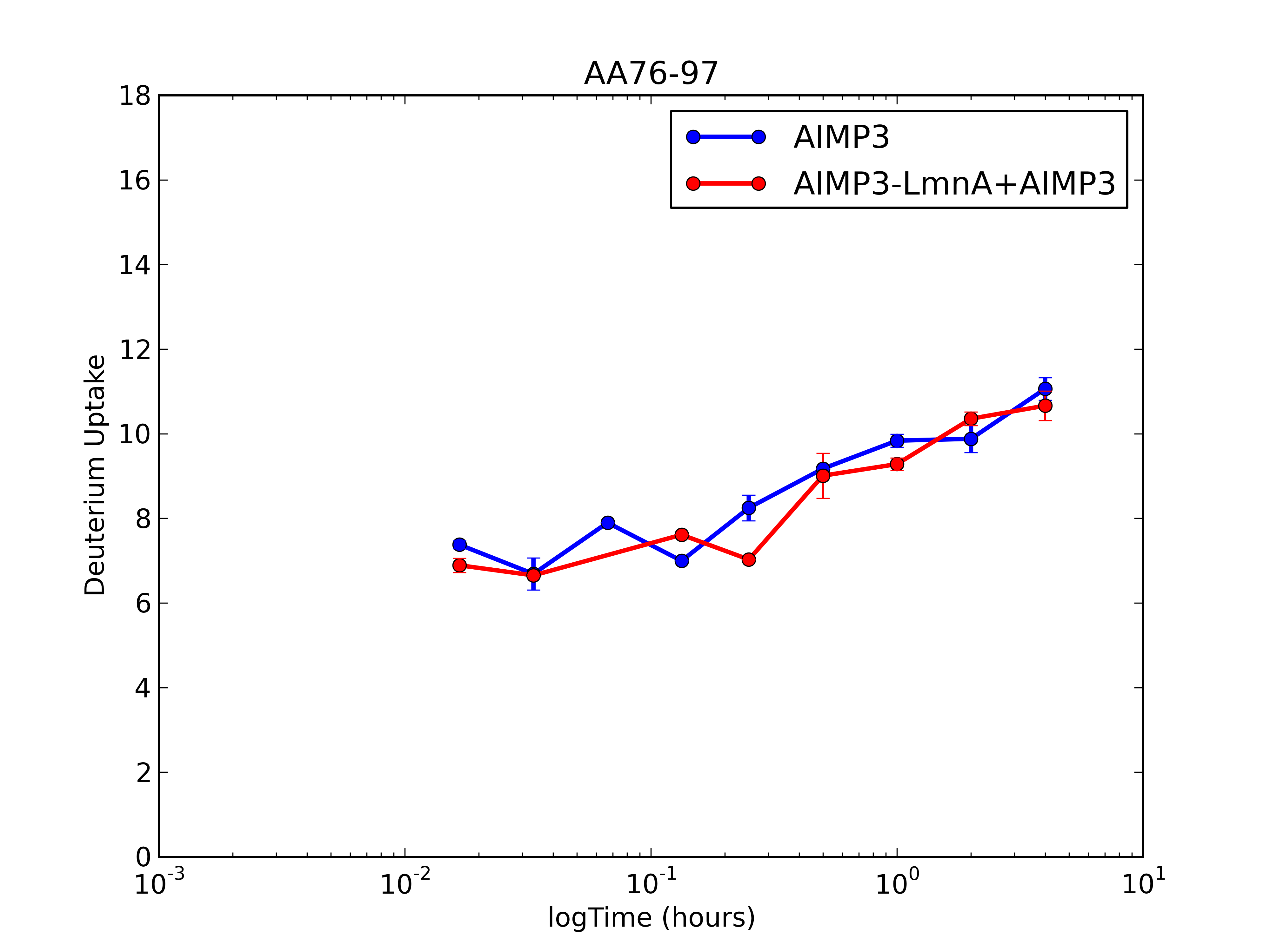

Supplement: S2 File — (ZIP) [file pone.0181869.s004.zip › logfigure-LmnA-scale/AA76-97_charge_2_mz1161.6.csv.csv.png]

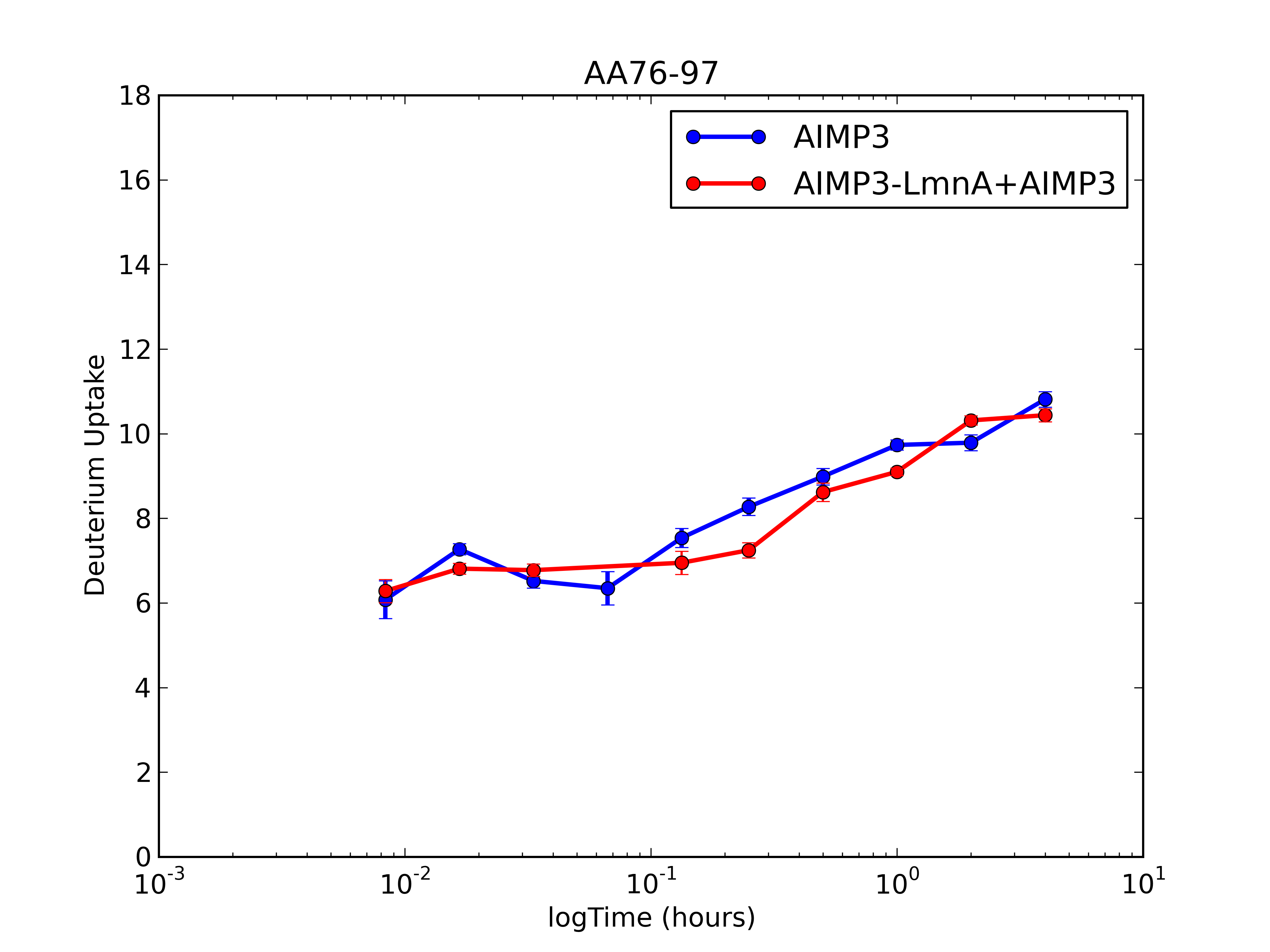

Supplement: S2 File — (ZIP) [file pone.0181869.s004.zip › logfigure-LmnA-scale/AA76-97_charge_3_mz774.7.csv.csv.png]

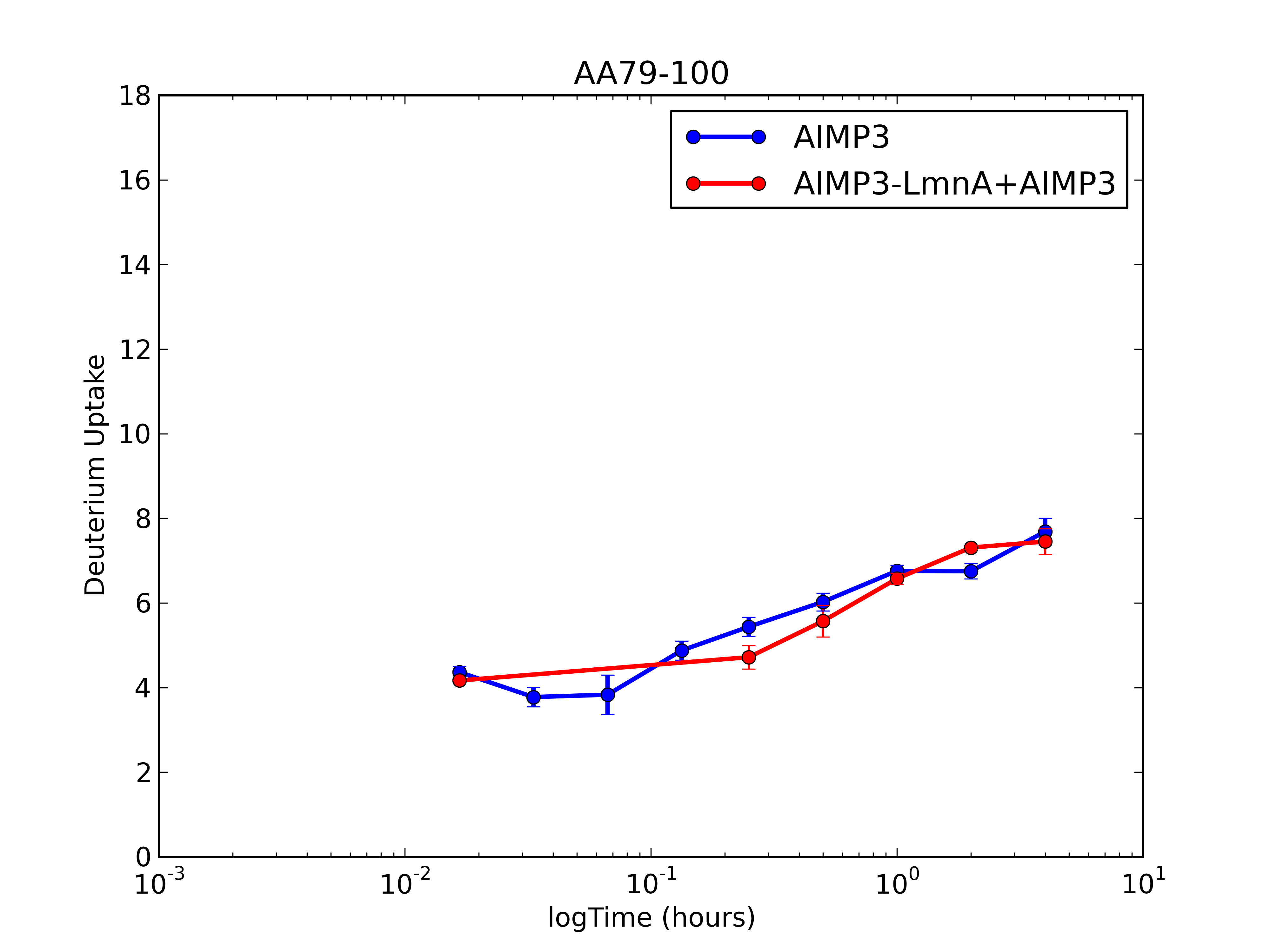

Supplement: S2 File — (ZIP) [file pone.0181869.s004.zip › logfigure-LmnA-scale/AA79-100_charge_2_mz1176.1.csv.csv.png]
